# 1,3-Dibromo-5,5-dimethylhydantoin as a Precatalyst for Activation of Carbonyl Functionality

**DOI:** 10.3390/molecules24142608

**Published:** 2019-07-17

**Authors:** Klara Čebular, Bojan Đ. Božić, Stojan Stavber

**Affiliations:** 1 Department of Physical and Organic Chemistry, Jožef Stefan Institute, Jamova 39, 1000 Ljubljana, Slovenia; 2 Jožef Stefan International Postgraduate School, Jamova 39, 1000 Ljubljana, Slovenia; 3 Institute of Physiology and Biochemistry, Faculty of Biology, University of Belgrade, Studentski trg 16, 11000 Belgrade, Serbia

**Keywords:** esterification, 1,3-dibromo-5,5-dimethylhydantoin, metal-free catalyst, aryl acids, alkyl acids

## Abstract

Activation of carbonyl moiety is one of the most rudimentary approaches in organic synthesis and is crucial for a plethora of industrial-scale condensation reactions. In esterification and aldol condensation, which represent two of the most important reactions, the susceptibility of the carbonyl group to nucleophile attack allows the construction of a variety of useful organic compounds. In this context, there is a constant need for development of and improvement in the methods for addition-elimination reactions via activation of carbonyl functionality. In this paper, an advanced methodology for the direct esterification of carboxylic acids and alcohols, and for aldol condensation of aldehydes using widely available, inexpensive, and metal-free 1,3-dibromo-5,5-dimethylhydantoin under neat reaction conditions is reported. The method is air- and moisture-tolerant, allowing simple synthetic and isolation procedures for both reactions presented in this paper. The reaction pathway for esterification is proposed and a scale-up of certain industrially important derivatives is performed.

## 1. Introduction

**Figure 1:**
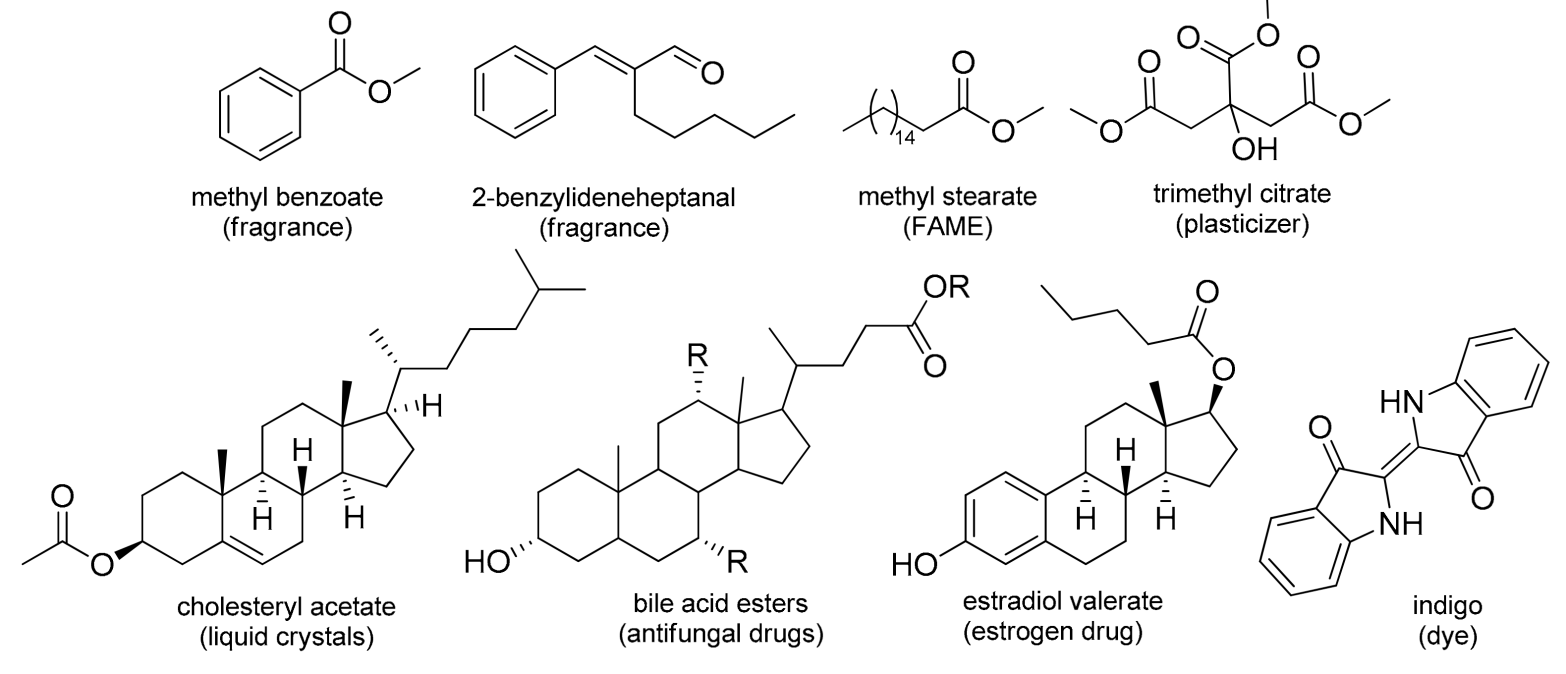
Structures of a selection of significant ester and aldol products.

(Trans)esterification is one of the most prominent transformations of carbonyl functionality in academia and industry due to the undeniable significance of the ester functional group in biological systems [1] and in the fabrication of a variety of products, such as cosmetics, plasticizers, pharmaceuticals, flavor chemicals, fine chemicals, adhesives, lubricants, electronic materials, etc. [2]. It is a vital reaction in the preparation of fatty acid methyl esters (FAME), which play a crucial role in producing detergents [3] and biodiesel fuel [4,5] and in microbial source tracking (MST) [6,7]. In developing active pharmaceutical ingredients (APIs), esterification is a valuable tool for modifying the physicochemical properties of drugs [8]. Esters with low molecular mass are widely used as fragrances and are components of essential oils [9] and pheromones [10], while certain steroidal esters are known as antifungal agents [11], as biocompatible and biodegradable polyesters for biomedical applications [12], or show antiproliferative and pro-apoptotic effects in cancer treatment [13]. Moreover, cholesterol esters and triglycerides belong to the main classes of lipids, which form the bulk of animal fats and vegetable oils. A particularly interesting cholesterol ester is cholesteryl acetate, since aside from being a component of gallstones, it has been readily exploited in constructing electronic displays [14]. On the other hand, bile acids play an important role in the digestion of lipids and in lipophilic vitamin reabsorbtion [15], and act as effective excipients (intestinal absorption enhancers, promoters, etc.) [16] (Figure 1).

The most extensively studied and commonly utilized esterification reaction is direct condensation between carboxylic acid and alcohol (Fischer esterification) [17]. Conventional approaches involve excessive reagents/dehydrative agents or use activated carboxylic acid derivatives in the presence of a stoichiometric base, leading to considerable amounts of byproducts and waste at the end of the process, and culminating in resource-consuming purification [18,19]. Consequently, catalytic direct esterification [20], lacking these disadvantages, has become the preferred method among researchers, employing a vast number of catalysts: Brønsted acids/bases [21,22], Lewis acids [23], metal catalysts [24], solid-supported catalysts [25,26], solid acids [27,28], ionic liquids [29,30], PPh_3_-based catalysts [31], enzymes [32], zeolites [33], etc.

However, in the context of sustainable industrial processes, there is an omnipresent struggle between the economical and ecological aspects of production. Therefore, an ideal method for a condensation reaction should include the use of an easily manipulable, low-cost, non-metal, water- and air-tolerant catalyst under mild, solvent-free reaction conditions without the need for simultaneous water removal, stoichiometric amounts of activators or large excesses of reagents. Furthermore, the methodology should be applicable to a broad substrate scope with high selectivity, providing high product yields, allowing scaling-up, and involving a simple purification procedure.

1,3-Dibromo-5,5-dimethylhydantoin (DBDMH) belongs to the group of cost-effective *N*-halamine disinfectants, which are becoming increasingly popular due to their long-term stability in dry storage or in a wide pH range of aqueous solutions, their safety for humans and the environment, and their ability to rapidly kill most microorganisms [34]. Furthermore, relative to inorganic halogens, they are less corrosive, more stable, and relatively cheap [35]. In addition, DBDMH is among the more established, commercially available *N*-halo reagents that are gradually attracting attention for being safe, stable, easily-handled solids that can be utilized under mild conditions for highly selective organic transformations [36,37,38,39]. Their distinctive chemical properties, owing to the presence of an N-X bond, instil them with broad, synthetic usability, especially as a source of halenium ions (X^+^) or halogen radicals (X^•^) [40]. Although DBDMH-mediated acetyl ester preparation has previously been reported, this method is limited only to acylation and includes the use of an activated carboxylic acid derivative (acetic anhydride) in the presence of a halogenated solvent (CH_2_Cl_2_) [41]. 

Our previous work described a convenient esterification method mediated by *N*-bromosuccinimide in substoichiometric amounts for direct esterification [42]. With this in mind, and in the context of our continued interest in improving and developing environmentally acceptable synthetic protocols [43,44,45,46,47,48], we now report the precatalytic activity of easily manipulable, metal-free DBDMH in the direct esterification of carboxylic acids and alcohols and in the aldol condensation of aldehydes.

## 2. Results and Discussion

### 2.1. Esterification

**Figure 2:**
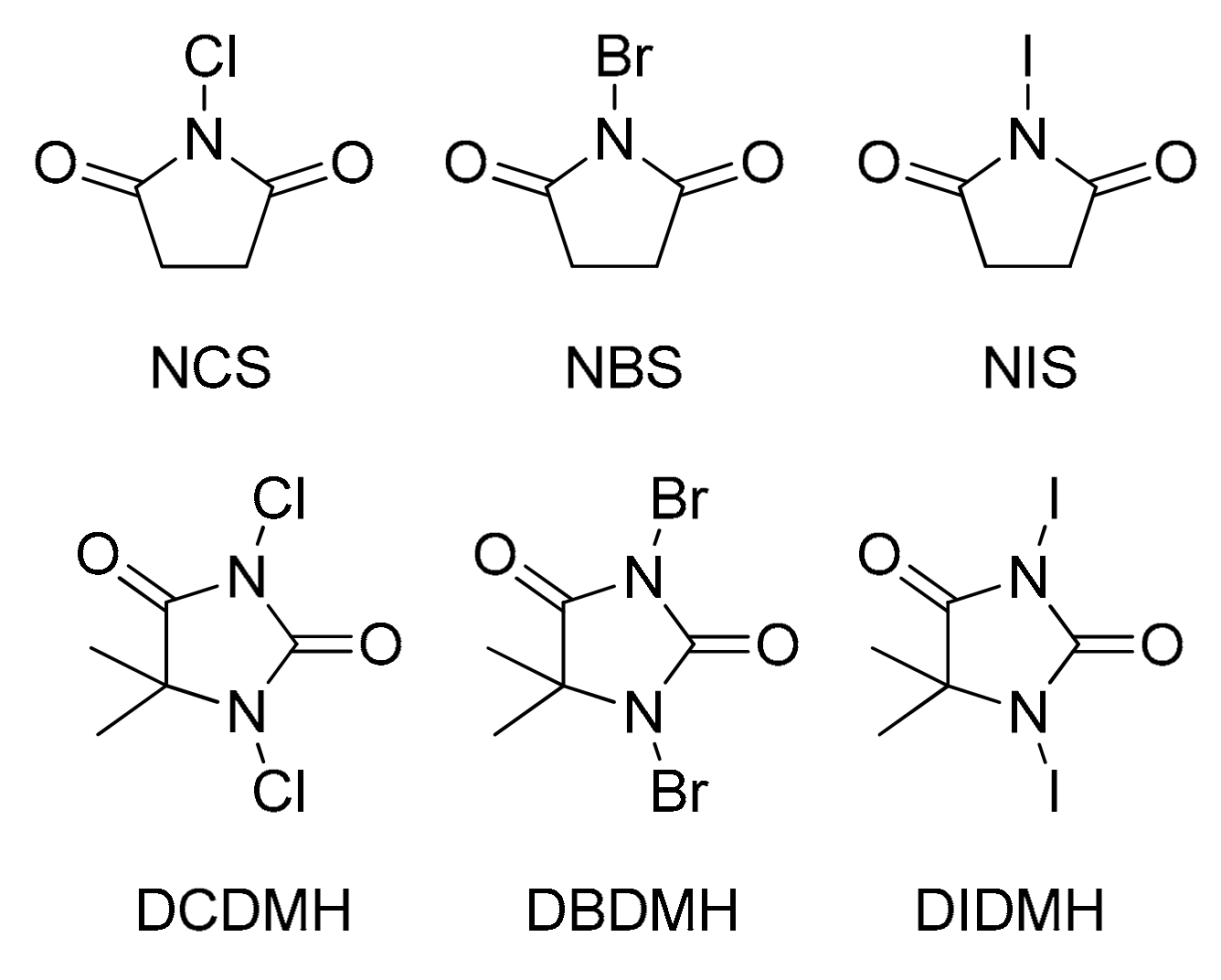
Structures of *N*-halosuccinimides and 1,3-dihalo-5,5-dimethylhydantoins (NCS: *N*-chlorosuccinimide, NBS: *N*-bromosuccinimide, NIS: *N*-iodosuccinimide, DCDMH: 1,3-dicholoro-5,5-dimethylhydantoin, DBDMH: 1,3-dibromo-5,5-dimethylhydantoin, DIDMH: 1,3-diiodo-5,5-dimethylhydantoin).

Initially, benzoic acid (**1**) was chosen as a model substrate to optimize the esterification reaction protocol. In the typical experimental procedure, the mixture of **1** and methanol (MeOH) was heated at 70 °C in the presence of different catalysts (Figure 2).

**Scheme 1:**
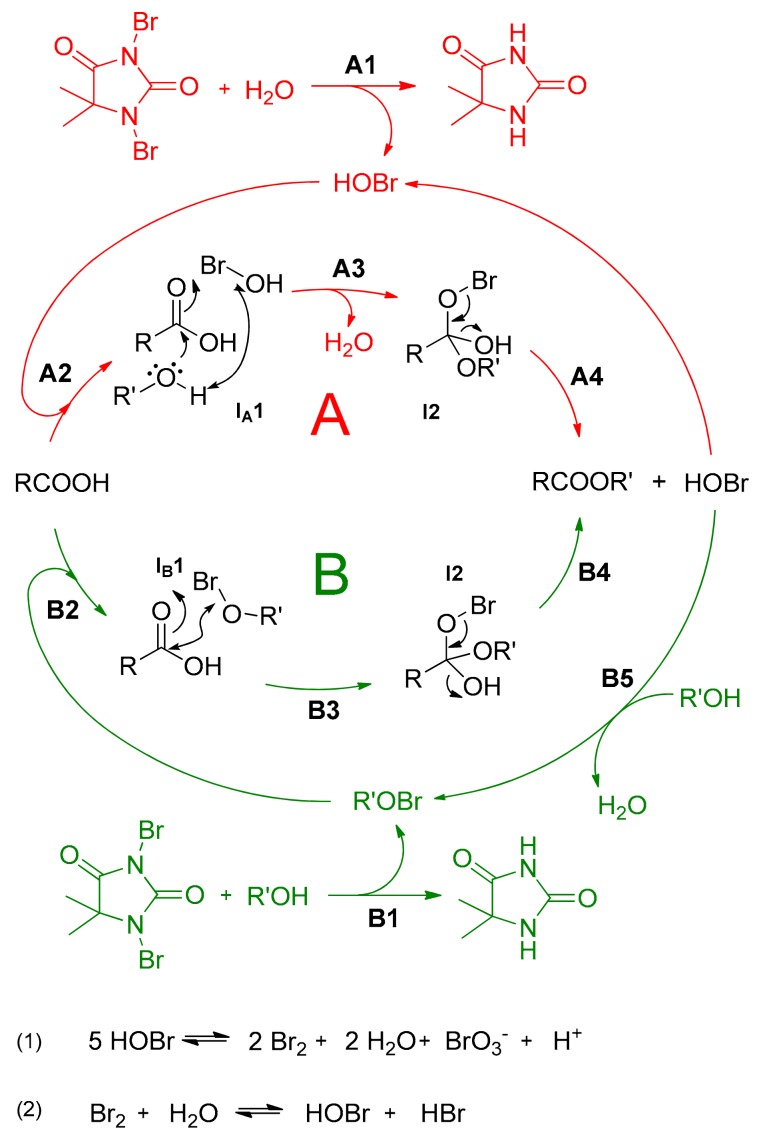
Plausible reaction pathway.

The results of this optimization are presented in Table 1. Among the studied catalysts (*N*-halosuccinimides, 1,3-dihalo-5,5-dimethylhydantoins, halogen mineral acids, molecular bromine and iodine), DBDMH exhibited the highest efficiency (Entry 3). In contrast, no conversion was noticed in the absence of catalyst (Entry 1). Furthermore, the effect of DBDMH loading was examined by changing its amount from a 3.5 molar percentage (mol%) to 7 mol% (Table 1, Entries 3, 7, and 8). No competing reaction was noticed, and although an excellent conversion was obtained even at 3.5 mol% (92%, Entry 8), a quantitative transformation to methyl benzoate was observed in the presence of 7 mol% of DBDMH (Entry 3). Therefore, further optimization was performed with 7 mol% of DBDMH. Furthermore, varying the temperature from 30 °C to 70 °C (Entries 3, 10, and 11) showed a considerable impact on esterification efficiency, with the optimal temperature being 70 °C (Entry 3). Under dry reaction conditions, the efficiency of esterification dropped for a substantial 20% (Entry 9), which suggests a water-assisted mechanism, as discussed below (Scheme 1).

**Figure 3:**
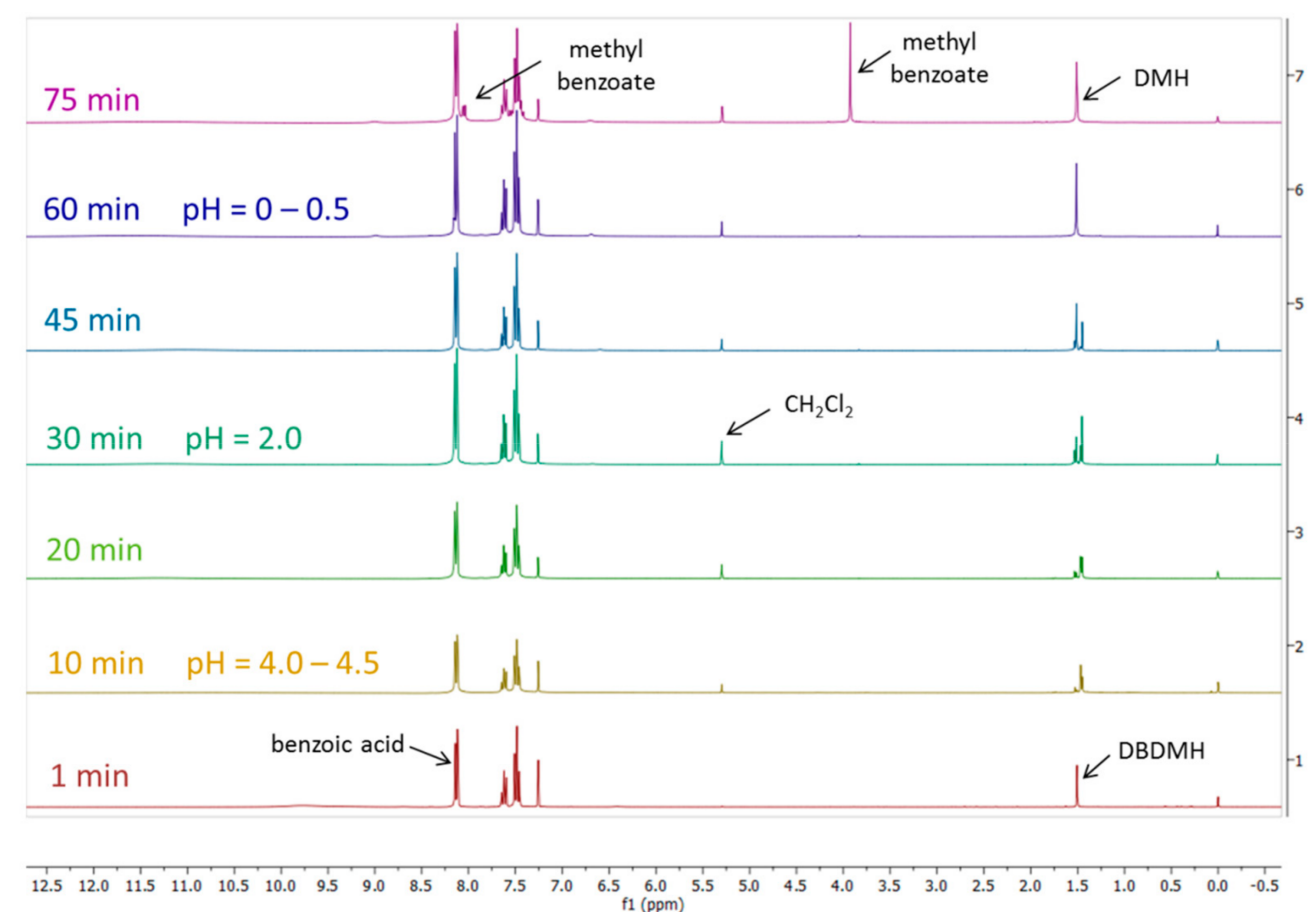
Timeline of the ^1^H-NMR spectra and pH measurements for esterification of benzoic acid with methanol.

To gain a clearer insight into the course of the reaction pathway, esterification of benzoic acid with methanol in the presence of 7 mol% of DBDMH under optimal reaction conditions was performed, with the reaction mixture being sampled every 10–15 min for the first 75 min of the reaction time and the spectra of each sample subsequently measured by ^1^H-NMR (Figure 3). Moreover, the pH of the reaction mixture was also measured after 10, 30, and 60 min. The results presented in Figure 3 show the decomposition of DBDMH to 5,5-dimethylhydantoin (DMH) over the first 60 min. Interestingly, esterification started after the complete conversion of DBDMH to DMH, after 75 min. Furthermore, no conversion to the ester product was observed when a control experiment was performed by heating a mixture of benzoic acid and methanol under optimal reaction conditions at 70 °C in the presence of 7 mol% DMH, suggesting that the formation of bromine specie(s) during DBDMH decomposition is crucial for the esterification reaction and that the role of DBDMH is precatalytic rather than catalytic (Table 2, Entry 3). Figure 3 shows a considerable drop in pH value from 4.0–4.5 to 0–0.5 over 60 min, which stayed unchanged throughout the remainder of the reaction (24 h).

These promising results encouraged us to investigate the efficiency of DBDMH in mediating the direct esterification of carboxylic acids with alcohols (Table 3, Table 4, Table 5, Table 6 and Table 7). In all cases, octanoic acid (**2**) showed significantly higher activity towards esterification than benzoic acid (**1**), yielding higher conversions (Table 3). Since, unlike in the case of octanoic acid, the carbonyl C-atom in the electron accepting carboxyl group of benzoic acid can receive electron density from resonance stabilization of the phenyl ring, benzoic acid is less reactive toward the nucleophile attack than octanoic acid. As reaction-limiting factors were found to include the nucleophilic character and steric properties of the substrate, different alcohols were tested (**a**–**j**, Table 3). The elongation of the alcohol alkyl chain (**a**, **d**, and **f**) had a stronger impact on the esterification efficiency of benzoic acid than on that of octanoic acid. In general, DBDMH mediation exhibited significantly higher conversions relative to *N*-bromosuccinimide (NBS), especially in the case of the secondary alcohols isopropanol (**c**) and cyclopentanol (**g**), as NBS mediation yielded only trace products or no conversion at all [42]. The reaction limitation was observed for the bulky tertiary alcohols *t*-BuOH (**e**) and adamantanol (**i**), as well as for phenol (**h**). However, if the sterically-demanding adamantyl group was separated from the alcohol group with a CH_2_ unit, the conversion increased to 79% for benzoic acid and 99% for alkyl acid (Table 3, **1j**, **2j**). The unreactivity in the case of the phenol molecule can be explained by the aromatic phenol structure, which possesses lower nucleophilicity due to the resonance delocalization of the oxygen electron pair in the OH group.

Moreover, a modest limitation of the substrate scope was observed for monosubstituted benzoic acids, as the reaction only failed in the case of 2-methoxybenzoic acid (Table 4) and hydroxy derivatives. The lower reactivity in case of ortho-substituted benzoic acids may be attributed to the steric hindrance of the ortho-positioned groups in the phenyl ring. The more the ortho-positioned group is sterically abundant, lower is the conversion. On the other hand, unreactivity of hydroxy-substituted derivatives is caused by the electron-donating properties of the OH group, which gives rise to low electron deficiency on the carbonyl C-atom, and consequently, lower susceptibility of carbonyl group to nucleophile attack. Bader charges for the carbon and oxygen atoms of the carbonyl group were calculated and the analysis confirmed that the carbonyl C-atom in the case of *p*-OH-benzoic acid was not as positively charged, as in the case of benzoic acid (Figure S1 and Table S1). When benzoic acid derivatives bore multiple electron-rich moieties on the aromatic ring, esterification was more limited, as only 3,4-dimethoxybenzoic acid produced an acceptable yield (Table 4, Compound **18a**). In the case of isonicotinic acid (Product **19a**), the unreactivity probably originated from the higher basicity and higher nucleophilicity of the nitrogen atom in the pyridine ring relative to the basicity (nucleophilicity) of the carbonyl oxygen atom in the carboxyl group. The nitrogen atom in the pyridine ring could therefore take a role of a Lewis base in a strong halogen bond interaction. [49] In net effect, it could therefore act as a Br^+^ scavenger, preventing the activation of carboxylic acid toward the nucleophile (alcohol) attack in the esterification reaction.

The commercial importance of certain methyl esters in biodiesel production (FAME) and perfumery (methyl benzoate) encouraged us to test the esterification of different types of alkyl carboxylic acids with MeOH under optimal reaction conditions in the presence of DBDMH (Table 5). No limitations were observed, and excellent yields were achieved, even in the case of polycarboxy derivatives.

In addition, the esterification of a selection of relevant bile acids was conducted to verify the synthetic applicability of this method on larger and more complex structures (Table 6). Esterification with methanol (**a**) and *n*-butanol (**d**) predominantly resulted in excellent yields, while the use of the secondary alcohol isopropanol (**c**) resulted in fair conversion.

Moreover, the results regarding the acetylation of cholesterol and epiandrosterone are presented in Table 7. Due to the high lipophilicity, and consequently, low solubility of these steroid alcohols in acetic acid, no esterification took place, and ethyl acetate was used as a reaction medium to obtain ester derivatives **34k** and **34l** in high yields. Using this approach, we also managed to demonstrate the potential of DBDMH as mediator in transesterification reactions. Additionally, to confirm the synthetic value of the presented methodology, scaled-up syntheses of methyl benzoate (**1a**), methyl stearate (**21a**), methyl citrate (**29a**), and cholic acid methyl ester (**30a**) up to 30 mmol were performed with excellent yields (93–100%).

The disinfection agency of DBDMH is known to originate from its ability to act as a source of Br^+^ in the presence of water through the release of hypobromous acid (HOBr), which further reacts as an oxidant in the process of disinfection [34]. However, as the substoichiometric amounts of DBDMH (7 mol%) used in the esterification method discussed herein were sufficient for high-yield, direct esterification between carboxylic acid and alcohol, Br^+^ clearly acted as a catalytic species rather than an oxidant.

Based on the results of the control experiments presented in Table 2 and Figure 3, the proposed reaction pathway is presented in Scheme 1. In general, DBDMH in the presence of traces of water decomposes to form HOBr (**A1**), which catalyses the esterification reaction and regenerates *via* path **A** (steps **A2**–**A4**, depicted in red colour). However, as the reaction yields 80% conversion even under dry starting reaction conditions (Table 2, Entry 7), the initial DBDMH decomposition most probably originates from its reaction with alcohol to form alkyl hypobromite [50], which further promotes the esterification reaction via path **B** (steps **B2**–**B4**, depicted in green colour). As a consequence of water formation during the esterification reaction (step **A3** or/and **B5**), the reaction can further proceed both ways simultaneously, where the formation of HOBr acts as the ultimate driving force of the reaction.

As already mentioned, a considerable drop in pH from 4.0–4.5 to 0–0.5 was measured during the reaction, which could not be attributed to the acidity of HOBr because of its relatively high pK_a_ value (pK_a_ = 8.65). At the same time, the formation of a distinctive orange color and bromine odor was observed during the reaction, which readily disappeared upon introduction of a sodium thiosulphate solution, NaS_2_O_3(aq)_, to the reaction mixture. All of this could be explained by the instability of HOBr and its decomposition at pH below 4 to form bromine (Br_2_) and bromic acid (HOBr_3_) with a considerably lower pK_a_ of −2 (Scheme 1, Equation 1) [51]. Bromine is well-known to form HOBr and HBr in reactions with water through an equilibrium reaction (Scheme 1, Equation 2), thereby allowing the regeneration of HOBr for subsequent esterification catalysis. Therefore, the water formed through the esterification reaction is more than only an inevitable side product, it is an assisting component in catalysis, due to this equilibrium reaction according to Le Chatelier’s principle. The hypothesis of water-assistance in the proposed mechanism is supported by the fact that only an 80% conversion to the ester product was observed when the reaction was performed in the presence of a drying agent (Na_2_SO_4_), which is in contrast to the general requirement for water removal for efficient transformation in an acid-catalyzed Fischer esterification methodology (Table 2, Entry 7). However, if the reaction was performed in water as a solvent, the hydrolysis of the ester product due to the carboxylic acid/ester equilibrium dominated the beneficial increased concentration of HOBr and the reaction resulted in no conversion. Furthermore, to eliminate the possibility of classic Brønsted acid catalysis acting as the major driving force of the quantitative esterification reaction (by the *in*-*situ* formed HBrO_3_ and HBr), the reaction was conducted in the presence of 7 mol% of hydrobromic acid (pK_a_ = −9), and only 84 percent conversion to methyl benzoate was noticed (Table 1, Entry 9).

### 2.2. Aldol Condensation

**Scheme 2:**
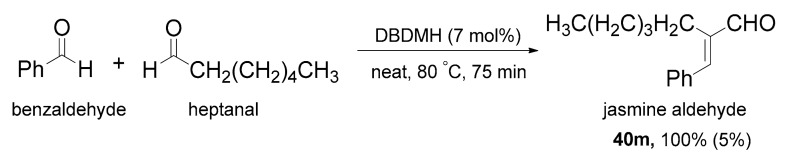
Synthesis of (*E*)-2-benzylideneheptanal (jasmine aldehyde).

To furtherly investigate possible DBDMH mediation in the activation of the carbonyl moiety, aldol condensation as an additional reaction model was chosen. Aldol condensation remains one of the most versatile, effective, and cheap methods for C–C bond formation in organic synthesis, widely employed in industry for the preparation of indigo dye as well as in preclinical and clinical drug-discovery research [52,53]. It also serves as a powerful tool in the fragrance industry for synthesising aromatic compounds, such as 2-benzylideneheptanal (jasmine odor), 2-methyl-3-(4-isopropylphenyl)propanal (cyclamen odor), and 2-methyl-4-(2,2,3-trimethyl-3-cyclopenten-1-yl)butanol (sandalwood odor) [54]. To optimize the reaction conditions, hexanal was used as a model substrate for self-aldol condensation of aldehydes, and the results of the optimization are presented in Table 8. The reaction proceeded under neat reaction conditions and was found to be time sensitive, as prolonging the reaction time resulted in the formation of polymeric material (Table 8, Entries 5–7). Furthermore, the optimal reaction conditions were then applied in studies to examine the scope of the methodology (Table 9). Excellent conversions and regioselectivity were obtained in the aldol condensation of alkyl aldehydes, and it was observed that chain elongation did not exhibit a significant influence on reaction conversion. Finally, the synthesis of the commercially-important product (*E*)-2-benzylideneheptanal (jasmine aldehyde, Scheme 2) as an example of cross-aldol condensation was performed, with quantitative conversion, yet with low yields, as a consequence of difficult separation resulting from the presence of excessive amounts of benzaldehyde reagent (3:1).

## 3. Materials and Methods

### 3.1. General Information

All reactions were performed in a Mettler-Toledo Easymax 102 Advanced Synthesis Workstation, using closed 25 mL vials. NMR spectra were recorded on a Varian Inova 300 spectrometer (300 MHz ^1^H, 75 MHz ^13^C, 285 MHz ^19^F) at 25 °C. ^1^H-NMR spectra were obtained as solutions in CDCl_3_ with TMS as an internal standard. ^19^F-NMR spectra were obtained as solutions in CDCl_3_ with CFCl_3_ as an internal standard. pH was measured with Whatman Panpeha pH indicator strips (pH range 0–14). All chemicals used for synthetic procedures were obtained from commercial sources and were of reagent grade purity or better (Merck, Darmstadt, Germany; Sigma Aldrich, St. Louis, MO, USA; Carlo Erba, Milano, Italy; Fluka, Seelze, Germany, Fisher Scientific, Waltham, MA, USA; Apollo Scientific, Bredbury, United Kingdom; etc.). Reactions were monitored by thin layer chromatography (TLC) with silica gel coated plates (Silica gel/TLC cards; DC-Alufolien-Kieselgel; with 60 Å medium pore diameter; Sigma Aldrich), and detection was conducted by UV absorption (254 nm, Camag, Muttenz, Switzerland). Purification of certain products was conducted on preparative silica gel glass plates (PLC Kieselgel 60 F254 with 2 mm layer thickness). Density functional theory (DFT) calculations were performed with the PWscf (Plain-Wave Self-Consistent Field) code from the Quantum ESPRESSO distribution [55,56], using the generalized gradient approximation (GGA) of Perdew–Burke–Ernzerhof (PBE) [57]. Bader charge analysis was performed by generating charge densities with single point self-consistent-field calculations of Ultra-Soft Pseudopotential (US-PP) optimized [58] structures using the PAW (projector-augmented-wave) potentials [59] and 1000 Ry kinetic energy cutoff for charge density, and then computing the Bader charges using the Bader program [60,61].

### 3.2. Experimental Procedures

#### 3.2.1. General Procedure for the Esterification between Carboxylic Acids and Alcohols

The mixture of carboxylic acid, alcohol, and 1,3-dibromo-5,5-dimethylhydantoin was stirred in a 25 mL reactor tube at 70 °C for 2–40 h. After reaction completion, the mixture was cooled to room temperature and the alcohol was evaporated under reduced pressure. The isolation procedure was as follows, except where noted differently in the Supporting Information. The residue was dissolved in 10 mL ethyl acetate and washed with a mixture of 1 mL saturated NaHCO_3(aq)_, 1 mL saturated Na_2_S_2_O_3(aq)_, and 10 mL distilled water, and the water phase was extracted with ethyl acetate (2 × 10 mL). The organic layers were combined, dried over Na_2_SO_4_, and the solvent was evaporated under reduced pressure.

#### 3.2.2. General Procedure for the Self-aldol Condensation of Aldehydes

The mixture of aldehyde (2.0 mmol) and 1,3-dibromo-5,5-dimethylhydantoin (0.14 mmol, 40 mg) was stirred in a 25 mL reactor tube at 80 °C for 45–90 min. After reaction completion, the mixture was cooled to room temperature and dissolved in 10 mL EtOAc. The solution was washed with the mixture of 1 mL saturated Na_2_S_2_O_3(aq)_, 1 mL saturated NaHCO_3(aq)_, and 10 mL distilled water. The water phase was extracted with ethyl acetate (3 × 10 mL). The organic layers were combined, dried with Na_2_SO_4_, and the solvent was evaporated under reduced pressure.

## 4. Conclusions

In summary, the precatalytic potential of cost-effective, accessible, and easily manipulable 1,3-dibromo-5,5-dimethylhydantoin as an air-tolerant mediator for the activation of carbonyl moieties has been demonstrated by developing a metal-free methodology for direct dehydrative esterification of carboxylic acids with alcohols, as well as for aldol condensation of aldehydes (self- and cross-condensation). This approach allows neat reaction conditions and does not need simultaneous water removal or stoichiometric amounts of activators. The esterification method using 1,3-dibromo-5,5-dimethylhydantoin provides good yields for a wide range of substrates. It was also successfully applied to certain significant steroidal backbones, such as cholesterol, bile acids, and epiandrosterone. Scaled-up synthesis of some commercially-interesting esters was successfully accomplished with high-to-excellent yields, and an esterification mechanism has been proposed. The effects of chain extension on self-aldol condensation products have been presented.

## Figures and Tables

**Table 1:** Optimization of reaction conditions ^1^.

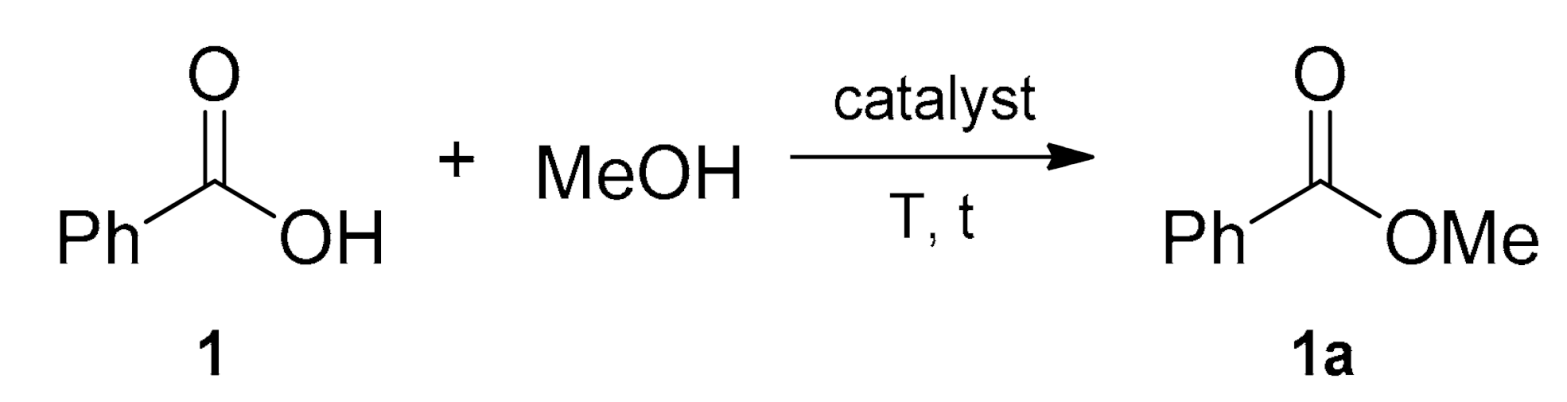					
Entry	Catalyst	Loading [mol%]	Temp. [°C]	Time [h]	Conv. [%] ^2^ (Yield [%])
1	/	/	70	20	0
2	DCDMH	7	70	20	88 (85)
3	DBDMH	7	70	20	100 (95)
4	DIDMH	7	70	20	30 (26)
5	HBr	7	70	20	84 (80) ^3^
6	I_2_	7	70	20	6 (3)
7	DBDMH	5	70	20	95 (90)
8	DBDMH	3.5	70	20	92 (88)
9	DBDMH	7	70	20	80 (77) ^4^
10	DBDMH	7	50	20	75 (72)
11	DBDMH	7	30	20	0
12	DBDMH	7	70	14	90 (85)
13	DBDMH	7	70	8	86 (82)
14	DBDMH	7	70	6	79 (75)
15	DBDMH	7	70	4	70 (66)
16	DBDMH	7	70	2	15 (10)

^1^ Reaction conditions: **1** (1.0 mmol), MeOH (0.5 mL), NXS, DXDMH, HCl (37%), HBr (48%), HI (57%), Br_2_, I_2_, T, t. ^2^ Conversions were determined by ^1^H-NMR analysis of the crude reaction mixtures. ^3^ Data from reference [42]. ^4^ Freshly dried MeOH was used, and the reaction performed over anhydrous Na_2_SO_4_ and under nitrogen atmosphere.

**Table 2:** pH measurements and conversion percentages of control esterification reactions ^1,2^.

Entry	Components of the Mixture	pH ^3^	Conv. [%]
1	MeOH	5.5	0
2	MeOH + DMH	5.5	0
3	MeOH + benzoic acid + DMH	3.5–4.0	0
4	MeOH + DBDMH	0.0–0.5	0
5	MeOH + benzoic acid	3.5–4.0	0
6	MeOH + benzoic acid + DBDMH	0.0–0.5	100 (95)
7	MeOH + benzoic acid + DBDMH + anh. Na_2_SO_4_^4^	0.0–0.5	80 (77)
8	MeOH + DCDMH	3.0–3.5	0
9	MeOH + DIDMH	4.0–4.5	0
10	*n*-octanol	5.0	0
11	*n*-octanol + DBDMH ^5^	0–0.5	<1% (octyl octanoate)
12	*n*-octanol + benzoic acid	5.0	0
13	*n*-octanol + benzoic acid/DBDMH	0–0.5	70 (65)

^1^ Reaction conditions: carboxylic acid (1 mmol), MeOH (0.5 mL), precatalyst (0.07 mmol), 70 °C, 20 h; ^2^ Conversions were determined after 20 h by ^1^H-NMR spectroscopy. ^3^ pH was measured after a reaction time of 1 h; ^4^ MeOH was dried with molecular sieves (4Å) and the reaction was performed under nitrogen atmosphere in the presence of Na_2_SO_4_; ^5^ Trace amounts of octyl octanoate were detected after 20 h.

**Table 3:** The effect of alcohol structure on the esterification of benzoic acid (**1**) and octanoic acid (**2**) ^1,2,3^.

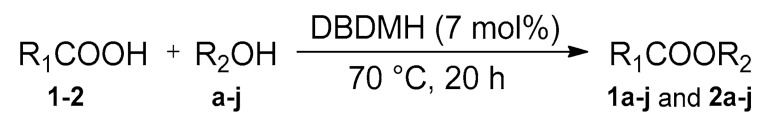		
**R_2_OH**	**R_1_ = Ph**	**R_1_ = Heptyl**
MeOH (**a**)	**1a**, 100% (95%)	**2a**, 100% (97%) ^5^
FCH_2_CH_2_OH (**b**)	**1b**, 100% (96%) 4	**2b**, 98% (92%) ^4^
*i*-PrOH (**c**)	**1c**, 29% (23%)	**2c**, 90% (86%)
*n*-BuOH (**d**)	**1d**, 82% (78%)	**2d**, 100% (95%) ^6^
*t*-BuOH (**e**)	**1e**, 0%	**2e**, 0%
*n*-Octanol (**f**)	**1f**, 70% (65%)	**2f**, 91% (88%)
Cyclopentanol (**g**)	**1g**, 23% (21)	**2g**, 100% (94%) ^6^
Phenol (**h**)	**1h**, 0%	**2h**, 0%
1-Adamantanol (**i**)	**1i**, 0%	**2i**, 0%
1-Adamantanemethanol (**j**)	**1j**, 79% (75%)	**2j**, 99% (94%)

^1^ Reaction conditions: carboxylic acid (1 mmol), alcohol (1 mmol, 2 mmol, or 0.5 mL), DBDMH (0.07 mmol), 70 °C, 20 h. ^2^ Conversions were determined by ^1^H-NMR spectroscopy. ^3^ The values in brackets indicate yields of isolated products. ^4^ Conversion (yield of isolated product) after 40 h. ^5^ Conversion (yield of isolated product) after 2 h. ^6^ Conversion (yield of isolated product) after 15 h.

**Table 4:** Scope width of aromatic carboxylic acids tested under optimized esterification conditions ^1,2,3^.

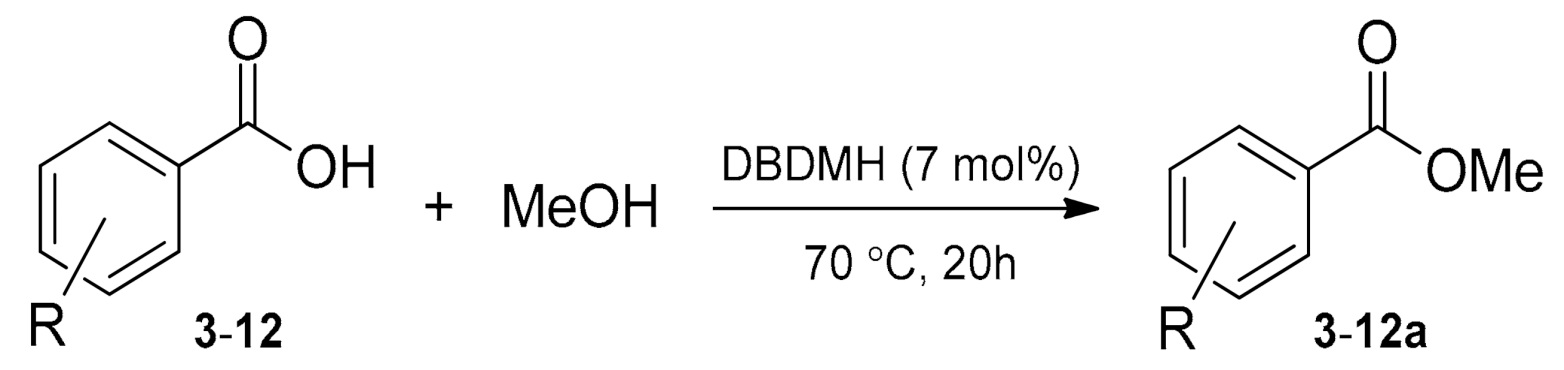			
**Methyl Benzoates**			
Entry	**R**	Compound	Conversion (Yield) [%]
1	4-NO_2_	**3a**	100% (97%) ^5^
2	4-F	**4a**	89% (85%) ^6^
3	4-Me	**5a**	94% (90%) ^6^
4	4-OMe	**6a**	74% (70%) ^6^
5	3-NO_2_	**7a**	100% (94%) ^6^
6	3-F	**8a**	91% (87%)
7	3-Me	**9a**	100% (98%) ^6^
8	3-OMe	**10a**	83% (78%) ^6^
9	2-NO_2_	**11a**	30% (25%)
10	2-F	**12a**	91% (85%)
11	2-Me	**13a**	82% (78%)
12	2-OMe	**14a**	0%
13	2-I	**15a**	62% (58%)
14	2,4-MeO	**16a**	0%
15	3,5-MeO	**17a**	0%
16	3,4-MeO	**18a**	70% (64%) ^4,6^
17	methyl isonicotinate	**19a**	0%

^1^ Reaction conditions: carboxylic acid (1 mmol), MeOH (0.5 mL), DBDMH (0.07 mmol), 70 °C, 20 h. ^2^ Conversions were determined by ^1^H-NMR spectroscopy. ^3^ The values in brackets stand for yields of isolated products. ^4^ Conversion (yield of isolated product) after 40 h. ^5^ A MeOH volume of 1.5 mL was used. ^6^ A MeOH volume of 2 mL was used.

**Table 5:** Scope of alkyl acids ^1,2,3^.

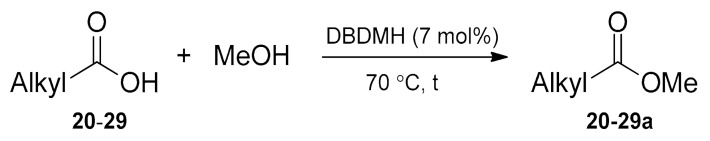
**Methyl alkyl esters**
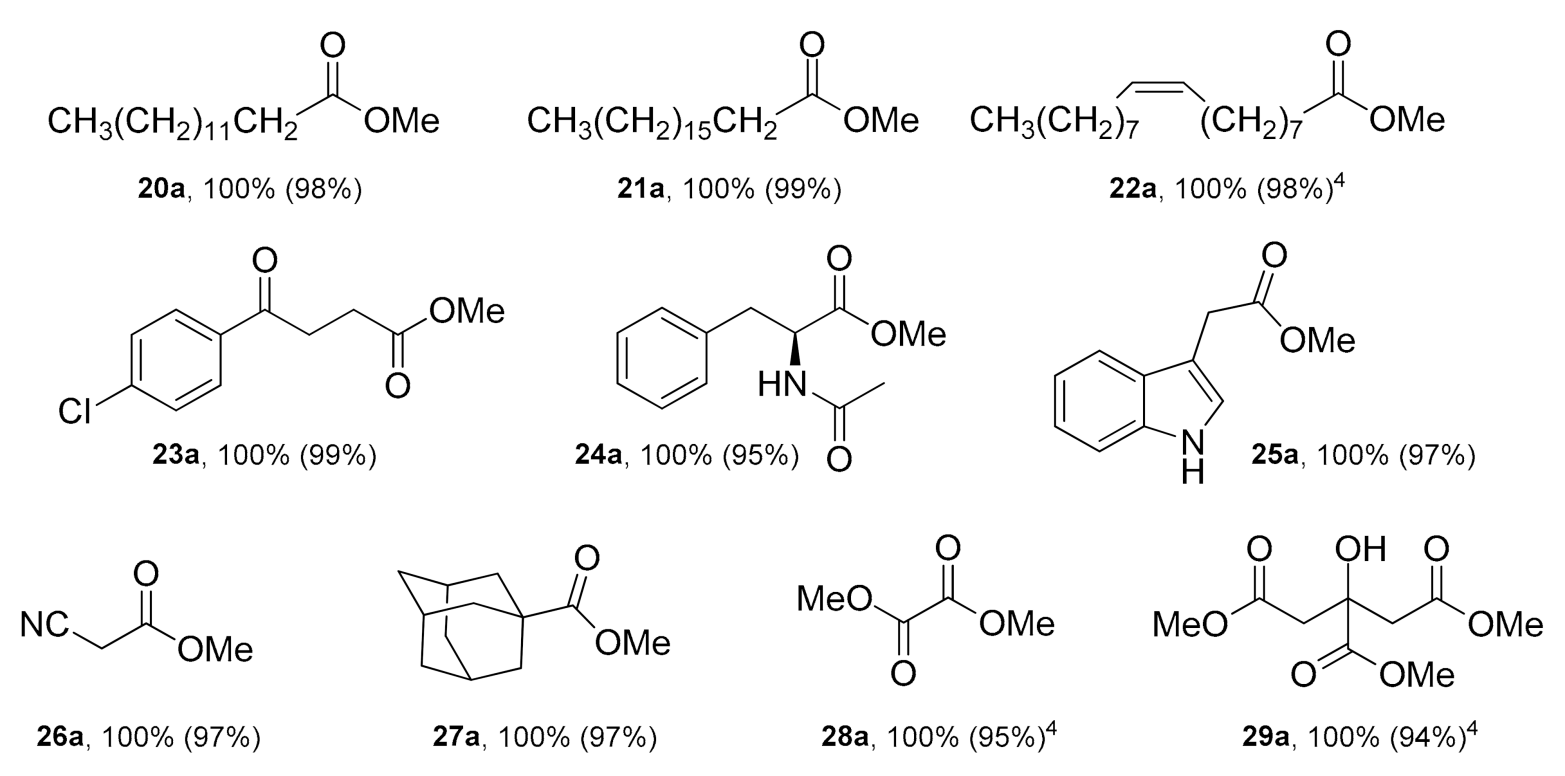

^1^ Reaction conditions: carboxylic acid (1 mmol), MeOH (0.5 mL), DBDMH (0.07 mmol), 70 °C, 2–20 h. ^2^ Conversions were determined by ^1^H-NMR spectroscopy. ^3^ The values in brackets indicate yields of isolated products. ^4^ A MeOH volume of 2 mL was used.

**Table 6:** Esterification of bile acids ^1,2,3^.

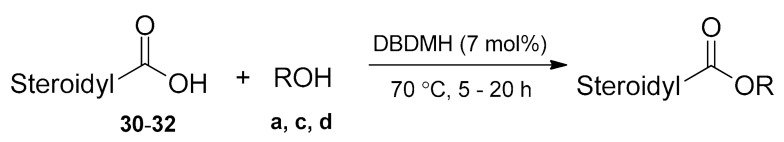
**Methyl Esters of Cholic Acid Derivatives**
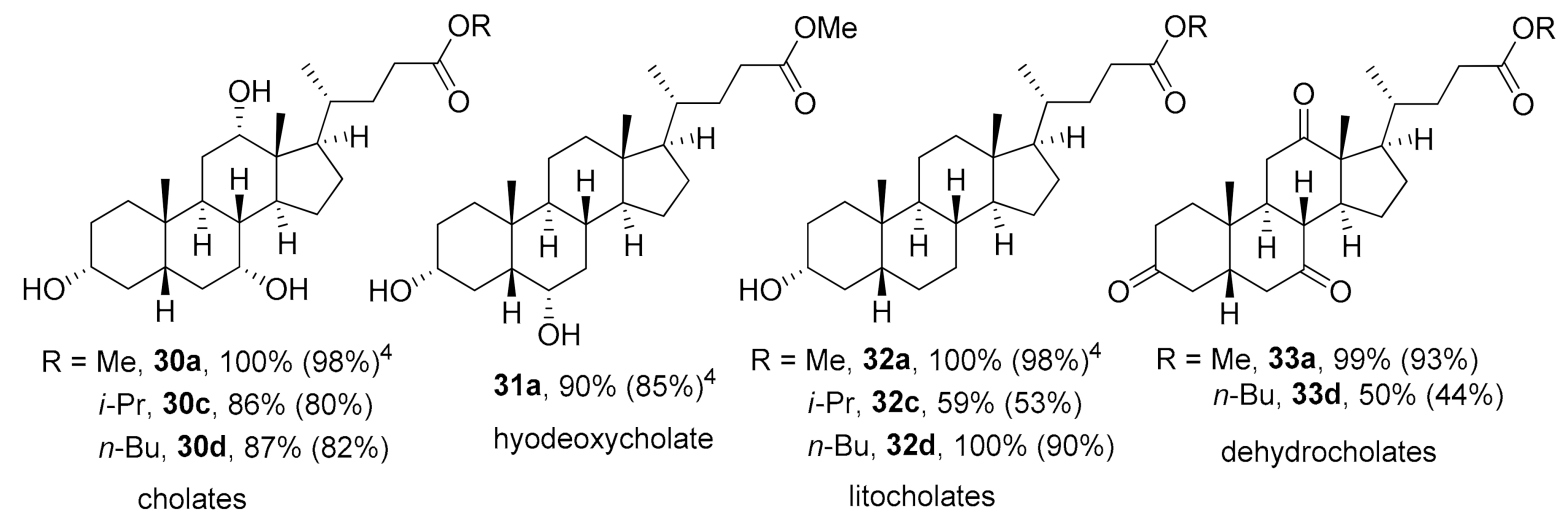

^1^ Reaction conditions: carboxylic acid (0.25 mmol), alcohol (0.5–2 mL), DBDMH (0.018 mmol), 70 °C, 5–20 h. ^2^ Conversions were determined by ^1^H-NMR spectroscopy. ^3^ The values in brackets indicate yields of isolated products. ^4^ A 2 mL volume of MeOH was used.

**Table 7:** Transesterification of cholesterol and epiandrosterone ^1,2,3^.

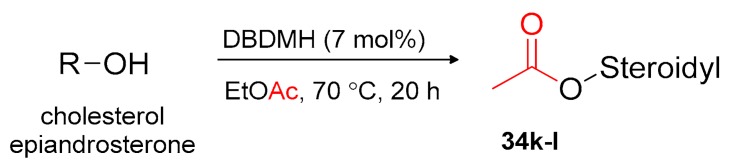
**Cholesteryl Acetate and Epiandrosteryl Acetate**
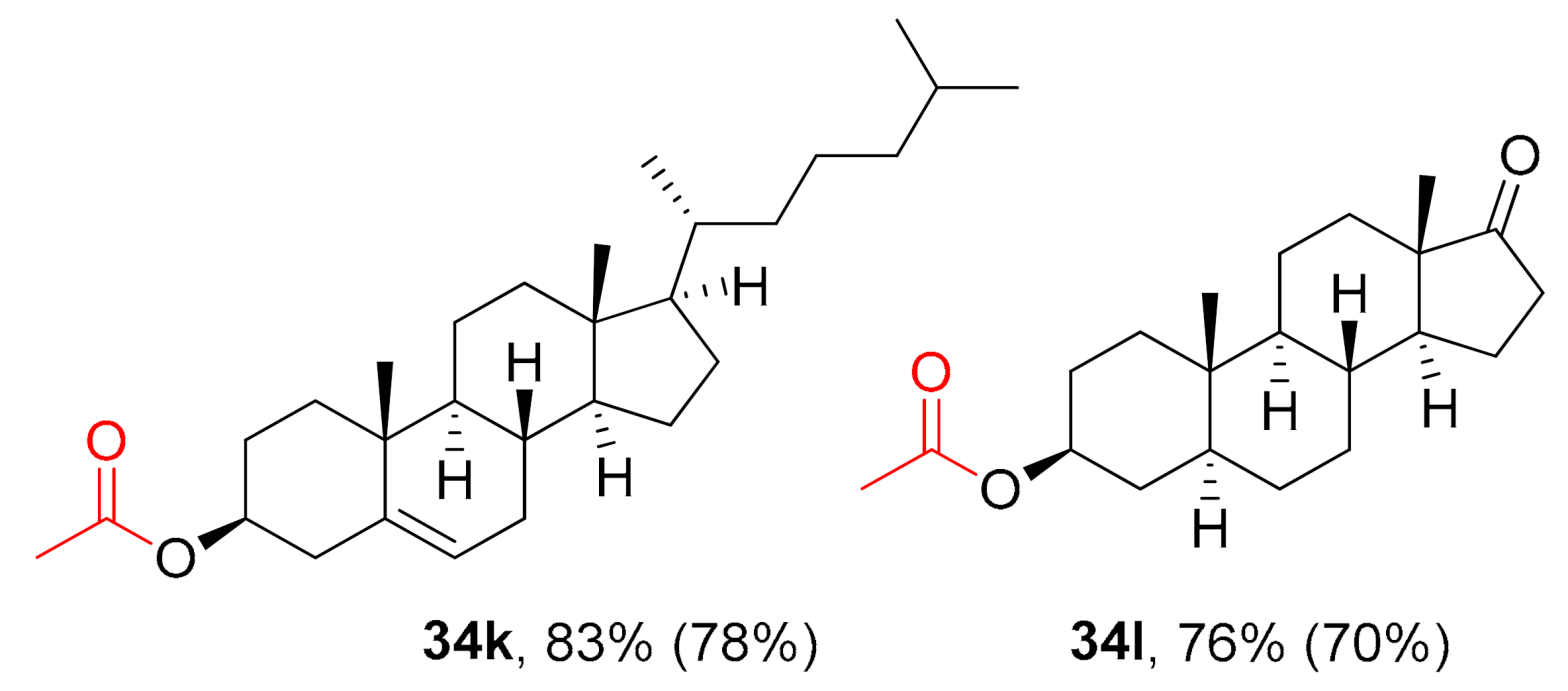

^1^ Reaction conditions: steroidal alcohol (0.25–0.5 mmol), EtOAc (1 mL), DBDMH (0.018–0.035 mmol), 70 °C, 20 h. ^2^ Conversions were determined by ^1^H-NMR spectroscopy. ^3^ The values in brackets stand for yields of isolated products.

**Table 8:** Catalyst, catalyst loading, temperature, and time optimization for aldol condensation of hexanal ^1^.

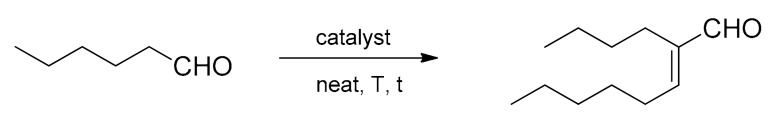					
**Entry**	**Catalyst**	**Loading [mol%]**	**Temperature [°C]**	**Time**	**Conv. [%]** ^2^
1	/	/	70	17 h	0
2	NCS	7	70	17 h	21
3	NBS	7	70	1 h	58
4	NIS	7	70	1 h	58
5	DBDMH	7	70	1 h	90 ^3^
6	DBDMH	7	80	1 h	99 ^3^
7	DBDMH	7	60	1 h	84 ^3^
8	DBDMH	7	80	45 min	100
9	DBDMH	7	80	30 min	97
10	DBDMH	7	80	15 min	91
11	DBDMH	7	80	10 min	76
12	DBDMH	7	80	5 min	62
13	DBDMH	5	80	45 min	94
14	DBDMH	3	80	45 min	90

^1^ Reaction conditions: hexanal (2.0 mmol), DBDMH, T, t. ^2^ Conversions were determined by ^1^H-NMR analysis of the crude reaction mixtures. ^3^ Considerable amounts of polymeric material were detected in the reaction mixture.

**Table 9:** Effect of chain elongation on aldol condensation efficiency ^1,2,3^.

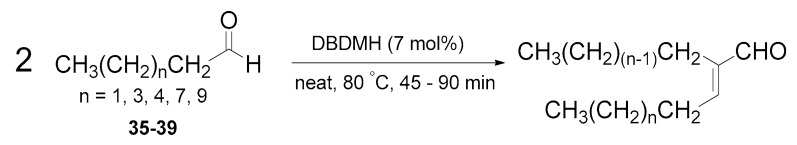				
**Product**	**n**	**Aldehyde**	**Product Structure**	**Conversion [%] (Yield [%])**
**35m**	1	Butanal	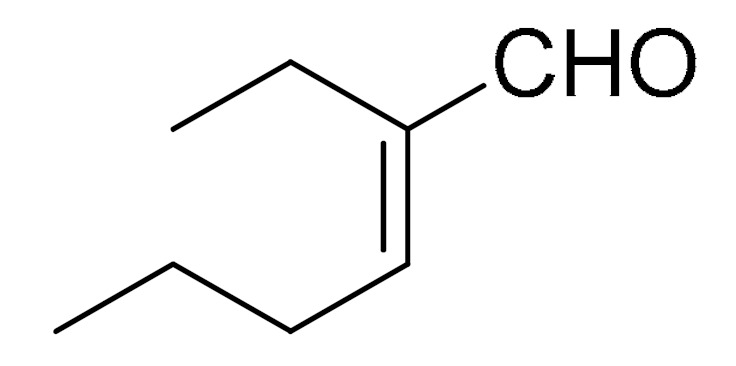	94 (90)
**36m**	3	Hexanal	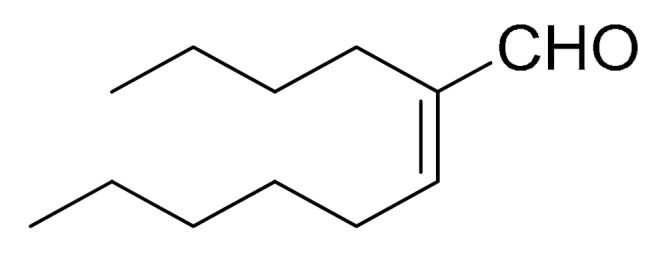	100 (94)
**37m**	4	Heptanal	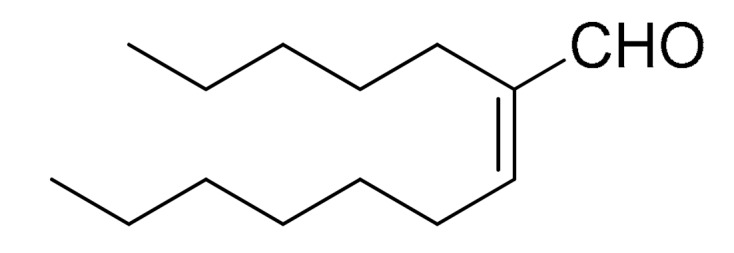	100 (95)
**38m**	7	Decanal	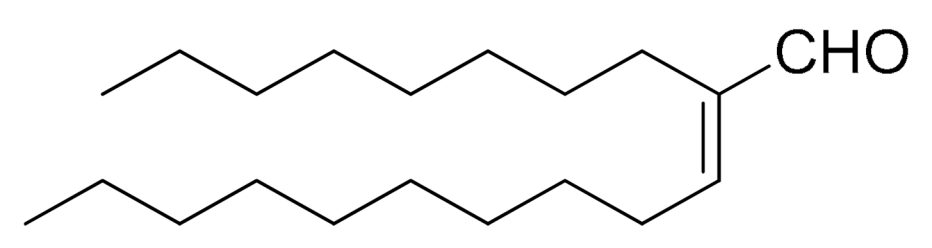	94 (90)
**39m**	9	Dodecanal	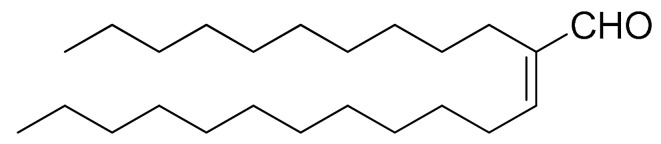	97 (92)

^1^ Reaction conditions: aldehyde (2.0 mmol), DBDMH (7 mol%), neat, 80 °C, 45–90 min. ^2^ Conversions were determined by ^1^H-NMR spectroscopy. ^3^ The values in brackets indicate yields of isolated products.

## Supplementary Materials

The following are available online, detailed experimental data, ^1^H-NMR, ^13^C-NMR, and ^19^F-NMR spectra of isolated final products.
