# Supplementary material for: 1,3-Dibromo-5,5-dimethylhydantoin as a Precatalyst for Activation of Carbonyl Functionality
Source: Molecules. 2019 Jul 17;24(14):2608. doi: 10.3390/molecules24142608 (PMC6681419; doi:10.3390/molecules24142608)
Supplement: Supplementary file 1 [file molecules-24-02608-s001.pdf]

Supporting Information

1,3-Dibromo-5,5-dimethylhydantoin as precatalyst for activation of carbonyl functionality

Klara Čebular[1,2], Bojan Đ. Božić[1,3], Stojan Stavber[1,2*]

[1] *Department of Physical and Organic Chemistry, Jožef Stefan Institute, Jamova 39, 1000 Ljubljana, Slovenia*
[2] *Jožef Stefan International Postgraduate School, Jamova 39, 1000 Ljubljana, Slovenia*
[3] *Institute of Physiology and Biochemistry, Faculty of Biology, University of Belgrade, Studentski trg 16, 11000 Belgrade, Serbia*

[*]*Corresponding author:* e-mail: stojan.stavber@ijs.si; tel.: +38614773660; fax: +38614235400

A. Optimization of reaction conditions for esterification of steroidyl esters 2

B. Experimental data 3

C. Scale-up procedure for preparation of methyl benzoate (**1a**) and isolation of 5,5-dimethylhydantoin 20

D. Scale-up procedure for preparation of methyl citrate (**29a**) 21

E. Scale-up procedure for preparation of methyl stearate (**21a**) 21

F. Scale-up procedure for preparation of cholic acid methyl ester (**30a**) 22

G. Copies of ^{1}H, ^{13}C and ^{19}F NMR spectra 23

H. References 83

A. Bader charge analysis

DFT calculations were performed with the PWscf code from the Quantum ESPRESSO distribution using the generalized gradient approximation (GGA) of Perdew--Burke--Ernzerhof (PBE). Bader charge analysis was performed by generating charge densities with single point self-consistent-field calculations of US-PP optimized structures using the PAW (projector-augmented-wave) potentials and 1000 Ry kinetic energy cutoff for charge density and then computing the Bader charges using the bader program.

The results of the Bader charge analysis are summarized in Table S1. Bader charges for the carbon and oxygen atoms of the carbonyl group were calculated and the analysis confirmed that the positive charge on the carbonyl carbon atom decreases by changing the substituents on the *para*-position of the phenyl ring in the following order: NO_2 > H > OMe > OH. In the case of *p*-OH-benzoic acid the carbon atom is not as positively charged as in the case of benzoic acid. It should however be noted that the results refer to gas-phase calculations and the presence of solvent could influence these results.

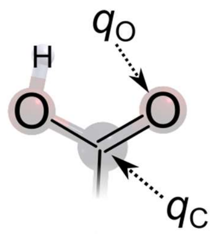

Figure S1. Schematic depiction of the meaning of labels used Table S1. The q_O label designated the charge of oxygen of the carbonyl group, while the q_C label designates the charge of the carbon of the carbonyl group, of the various investigated carboxylic acids.

Table S1. Calculated Bader charges for the carbon (q_C) and oxygen (q_O) atoms of the carbonyl group in units of elementary charge (schematically depicted in Fig. S1), for investigated substituted benzoic acids.

**Species**	**q_C [*e*]**	**q_O [*e*]**
p-NO_2-benzoic acid	2.70	-1.84
benzoic acid	2.66	-1.85
p-OMe-benzoic acid	2.64	-1.87
p-OH-benzoic acid	2.60	-1.85

1.

B. Optimization of reaction conditions for esterification of steroidyl esters

Table S2. Catalyst, catalyst loading, temperature and time optimization for esterification of cholic acid with methanol.[a,b]

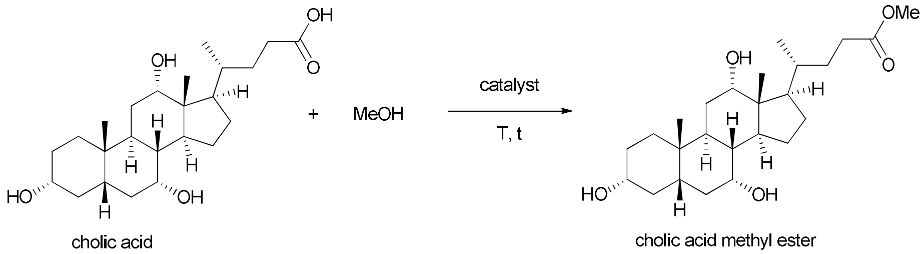

Entry	Catalyst	Loading [mol%]	Temperature [°C]	Time [h]	Conv. [%][b]
1	NCS	7	70	1	17
2	NBS	7	70	1	91
3	NIS	7	70	1	39
4	I_2	7	70	1	71
5	DBDMH	7	70	1	92
6	DBDMH	10	70	1	92
7	DBDMH	5	70	1	85
8	DBDMH	3.5	70	1	82
9	DBDMH	7	80	1	92
10	DBDMH	7	60	1	90
11	DBDMH	7	50	1	82
12	DBDMH	7	70	2	93
13	DBDMH	7	70	3	94
14	DBDMH	7	70	4	94
15	DBDMH	7	70	5	100

[a] Reaction conditions: cholic acid (0.25 mmol), MeOH (0.5 mL), NXS, DBDMH, I_2, T, t.
[b] Conversions were determined by ^{1}H NMR analysis of the crude reaction mixtures.

C. Experimental data

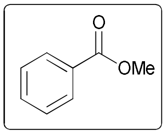

Methyl benzoate (1a)[1]
Reaction conditions: According to the general procedure; benzoic acid (1 mmol, 122.1 mg), MeOH (0.5 mL), DBMDH (0.070 mmol, 20.0 mg), 70 °C, 20 h.
Purification: Not necessary.
Yield: 129 mg, 95%; colourless oil;
^{1}H NMR (300 MHz, $CDCl_3$) δ 8.07 – 8.01 (m, 2H), 7.59 – 7.51 (m, 1H), 7.43 (ddt, J = 8.2, 6.8, 1.1 Hz, 2H), 3.91 (s, 3H); **^{13}C NMR** (76 MHz, CDCl3) δ 167.2, 133.0, 130.3, 129.7, 128.4, 52.2; **HRMS** (ESI) for $C_8H_8O_2$: calculated m/z = 137.0603 (MH^+); found m/z = 137.0606 (MH^+).

Methyl octanoate (2a)[2]

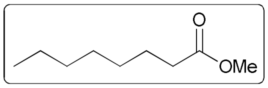

Reaction conditions: According to the general procedure; octanoic acid (1 mmol, 158.5 μL), MeOH (0.5 mL), DBDMH (0.070 mmol, 20.0 mg), 70 °C, 2 h.
Purification: Not necessary.
Yield: 153 mg, 97% yield; colourless oil;
^{1}H NMR (300 MHz, $CDCl_3$) δ 3.67 (s, 3H), 2.30 (t, *J* = 7.5 Hz, 2H), 1.69 – 1.56 (m, 2H), 1.35 – 1.25 (m, 8H), 0.88 (t, *J* = 6.9 Hz, 3H); **^{13}C NMR** (76 MHz, $CDCl_3$) δ 174.5, 51.5, 34.2, 31.8, 29.2, 29.0, 25.1, 22.7, 14.2; **HRMS** (ESI) for $C_9H_{18}O_2$: calculated m/z = 159.1385 (MH^+); found m/z = 159.1389 (MH^+).

Methyl 4-nitrobenzoate (3a)[1]

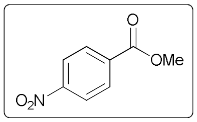

Reaction conditions: According to the general procedure; 4-nitrobenzoic acid (1 mmol, 167.1 mg), MeOH (1.5 mL), DBDMH (0.070 mmol, 20.0 mg), 70 °C, 20 h.
Purification: Not necessary.
Yield: 176 mg, 97% yield; white solid;
^{1}H NMR (300 MHz, $CDCl_3$) δ 8.34 – 8.19 (m, 4H), 3.99 (s, 3H); **^{13}C NMR** (76 MHz, $CDCl_3$) δ 165.2, 150.6, 135.6, 130.8, 123.6, 52.9. **HRMS** (ESI) for $C_8H_7NO_4$: calculated m/z = 182.0453 (MH^+); found m/z = 182.0457 (MH^+).

Methyl 4-fluorobenzoate (4a)[3]

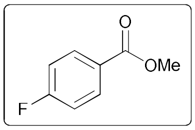

Reaction conditions: According to the general procedure; 4-fluorobenzoic acid (1 mmol, 140.1 mg), MeOH (2 mL), DBDMH (0.070 mmol, 20.0 mg), 70 °C, 20 h.
Purification: Not necessary.
Yield: 131 mg, 85% yield; colourless oil;
^{1}H NMR (300 MHz, $CDCl_3$) δ 8.02 (ddd, *J* = 10.1, 5.2, 2.5 Hz, 2H), 7.13 – 7.03 (m, 2H), 3.89 (s, 3H); **^{13}C NMR** (76 MHz, CDCl3) δ 166.2, 165.8 (d, J = 253.5 Hz) 132.2 (d, J = 9.4 Hz), 126.5 (d, J = 3.0 Hz), 115.6 (d, J = 22.0 Hz), 52.2; **^{19}F NMR** (285 MHz, CDCl3) δ -106.34 (tt, J = 8.5, 5.4 Hz); **HRMS** (ESI) for $C_8H_7FO_2$: calculated m/z = 155.0508 (MH^+); found m/z = 155.0505 (MH^+).

Methyl 4-methylbenzoate (5a)[3]

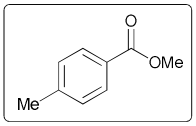

Reaction conditions: According to the general procedure; 4-methylbenzoic acid (1 mmol, 136.1 mg), MeOH (2 mL), DBDMH (0.070 mmol, 20.0 mg), 70 °C, 20 h.
Purification: Not necessary.
Yield: 135 mg, 90% yield; colourless oil;
^{1}H NMR (300 MHz, $CDCl_3$) δ 7.97 – 7.86 (m, 2H), 7.22 (d, *J* = 8.2 Hz, 2H), 3.88 (s, 3H), 2.39 (s, 3H); **^{13}C NMR** (76 MHz, $CDCl_3$) δ 167.2, 143.6, 129.6, 129.1, 127.5, 52.0, 21.7; **HRMS** (ESI) for $C_9H_{10}O_2$: calculated m/z = 151.0759 (MH^+); found m/z = 151.0755 (MH^+).

Methyl 4-methoxybenzoate (6a)[4]

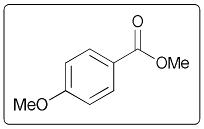

Reaction conditions: According to the general procedure; 4-methoxybenzoic acid (1 mmol, 152.2 mg), MeOH (2 mL), DBDMH (0.070 mmol, 20.0 mg), 70 °C, 20 h.
Purification: Not necessary.
Yield: 116 mg, 70% yield; colourless oil;
^{1}H NMR (300 MHz, $CDCl_3$) δ 8.08 – 7.89 (m, 2H), 7.00 – 6.83 (m, 2H), 3.86 (s, 3H), 3.82 (s, 3H); **^{13}C NMR** (76 MHz, $CDCl_3$) δ 166.8, 163.3, 131.5, 122.6, 113.6, 55.3, 51.8; **HRMS** (ESI) for $C_9H_{10}O_3$: calculated m/z = 167.0708 (MH^+); found m/z = 167.0712 (MH^+).

Methyl 3-nitrobenzoate (7a)[4]

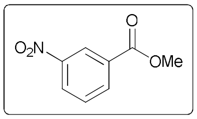

Reaction conditions: According to the general procedure; 3-nitrobenzoic acid (1 mmol, 167.1 mg), MeOH (2 mL), DBDMH (0.070 mmol, 20.0 mg), 70 °C, 20 h.
Purification: Not necessary.
Yield: 141 mg, 78% yield; yellow solid;
^{1}H NMR (300 MHz, $CDCl_3$) δ 8.94 – 8.75 (m, 1H), 8.50 – 8.30 (m, 2H), 7.68 (t, J = 8.0 Hz, 1H), 4.00 (s, 3H); **^{13}C NMR** (76 MHz, $CDCl_3$) δ 165.0, 148.3, 135.3, 131.9, 129.7, 127.4, 124.6, 52.8; **HRMS** (ESI) for $C_8H_7NO_4$: calculated m/z = 182.0453 (MH^+); found m/z = 182.0457 (MH^+).

Methyl 3-fluorobenzoate (8a)[5]

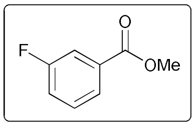

Reaction conditions: According to the general procedure; 3-fluorobenzoic acid (1 mmol, 140.1 mg), MeOH (0.5 mL), DBDMH (0.070 mmol, 20.0 mg), 70 °C, 20 h.
Purification: Not necessary.
Yield: 128 mg, 83% yield; colourless oil;
^{1}H NMR (300 MHz, $CDCl_3$) δ 7.83 (dt, J = 7.7, 1.2 Hz, 1H), 7.71 (ddd, J = 9.4, 2.6, 1.5 Hz, 1H), 7.41 (td, J = 8.0, 5.6 Hz, 1H), 7.25 (tdd, J = 8.3, 2.5, 1.0 Hz, 1H), 3.92 (s, 3H); **^{13}C NMR** (76 MHz, $CDCl_3$) δ 166.0 , 162.6 (d, J = 246.6 Hz), 132.4 (d, J = 7.6 Hz), 130.1 (d, J = 7.6 Hz), 125.4 (d, J = 2.9 Hz), 120.1 (d, J = 21.4 Hz), 116.6 (d, J = 23.0 Hz), 52.4; **^{19}F NMR** (285 MHz, $CDCl_3$) δ -112.92 (ddd, J = 9.4, 8.4, 5.6 Hz); **HRMS** (ESI) for $C_8H_7FO_2$: calculated m/z = 155.0508 (MH^+); found m/z = 155.0511 (MH^+).

Methyl 3-methylbenzoate (9a)[3]

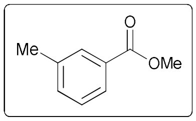

Reaction conditions: According to the general procedure; 3-methylbenzoic acid (1 mmol, 136.1 mg), MeOH (2 mL), DBDMH (0.070 mmol, 20.0 mg), 70 °C, 20 h.
Purification: Not necessary.
Yield: 147 mg, 98% yield; colourless oil;
^{1}H NMR (300 MHz, $CDCl_3$) δ 7.88 – 7.81 (m, 1H), 7.39 – 7.28 (m, 1H), 3.91 (s, 2H), 2.40 (s, 3H). **^{13}C NMR** (76 MHz, $CDCl_3$) δ 167.3, 138.2, 133.7, 130.2, 130.2, 128.3, 126.8, 52.1, 21.3;

HRMS (ESI) for $C_9H_{10}O_2$: calculated m/z = 151.0759 (MH^+); found m/z = 151.0762 (MH^+).

Methyl 3-methoxybenzoate (10a)[6]

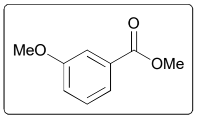

Reaction conditions: According to the general procedure; 3-methoxybenzoic acid (1 mmol, 152.1 mg), MeOH (2 mL), DBDMH (0.070 mmol, 20.0 mg), 70 °C, 20 h.
Purification: Not necessary.
Yield: 130 mg, 78% yield; yellow oil;
^{1}H NMR (300 MHz, $CDCl_3$) δ 7.66 – 7.53 (m, 2H), 7.33 (t, *J* = 7.9 Hz, 1H), 7.09 (ddd, *J* = 8.2, 2.6, 0.9 Hz, 1H), 3.91 (s, 3H), 3.84 (s, 3H); **^{13}C NMR** (76 MHz, $CDCl_3$) δ 167.0, 159.6, 131.5, 129.4, 122.0, 119.5, 114.0, 55.4, 52.2; **HRMS** (ESI) for $C_9H_{10}O_3$: calculated m/z = 167.0708 (MH^+); found m/z = 167.0717 (MH^+).

Methyl 2-nitrobenzoate (11a)[7]

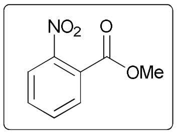

Reaction conditions: According to the general procedure; 2-nitrobenzoic acid (1 mmol, 167.1 mg), MeOH (0.5 mL), DBDMH (0.070 mmol, 20.0 mg), 70 °C, 20 h.
Purification: Not necessary.
Yield: 45 mg, 25% yield; white solid;
^{1}H NMR (300 MHz, $CDCl_3$) δ 7.91 (dd, *J* = 7.4, 1.9 Hz, 1H), 7.78 – 7.59 (m, 3H), 3.93 (s, 3H); **^{13}C NMR** (76 MHz, $CDCl_3$) δ δ 166.0, 148.3, 133.0, 131.9, 129.9, 127.6, 124.0, 53.3; **HRMS** (ESI) for $C_8H_7NO_4$: calculated m/z = 182.0453 (MH^+); found m/z = 182.0456 (MH^+).

Methyl 2-fluorobenzoate (12a)[7]

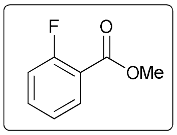

Reaction conditions: According to the general procedure; 2-fluorobenzoic acid (1 mmol, 140.1 mg), MeOH (0.5 mL), DBDMH (0.070 mmol, 20.0 mg), 70 °C, 20 h.
Purification: Not necessary.
Yield: 131 mg, 85% yield; colourless oil;
^{1}H NMR (300 MHz, $CDCl_3$) δ 7.93 (td, *J* = 7.6, 1.9 Hz, 1H), 7.57 – 7.45 (m, 1H), 7.20 (td, *J* = 7.6, 1.1 Hz, 1H), 7.13 (ddd, *J* = 10.9, 8.3, 1.1 Hz, 1H), 3.93 (s, 3H); **^{13}C NMR** (76 MHz, $CDCl_3$) δ 164.96 (d, *J* = 2.9 Hz), 162.00 (d, *J* = 259.7 Hz), 134.55 (d, *J* = 9.0 Hz), 132.20, 124.02 (d, *J* = 3.5 Hz), 118.72 (d, *J* = 10.1 Hz), 117.03 (d, *J* = 22.5 Hz), 52.36; **^{19}F NMR** (285 MHz, $CDCl_3$) δ -109.58 (ddd, *J* = 10.9, 7.3, 4.9 Hz); **HRMS** (ESI) for $C_8H_7FO_2$: calculated m/z = 155.0508 (MH^+); found m/z = 155.0505 (MH^+).

Methyl 2-methylbenzoate (13a)[7]

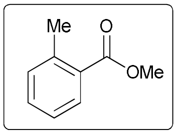

Reaction conditions: According to the general procedure; 2-methylbenzoic acid (1 mmol, 136.1 mg), MeOH (0.5 mL), DBDMH (0.070 mmol, 20.0 mg), 70 °C, 20 h.
Purification: Not necessary.
Yield: 117 mg, 78% yield; colourless oil;

^{1}H NMR (300 MHz, $CDCl_3$) δ 7.90 (dd, *J* = 8.1, 1.5 Hz, 1H), 7.37 (td, *J* = 7.5, 1.5 Hz, 1H), 7.28 – 7.14 (m, 2H), 3.87 (s, 3H), 2.59 (s, 3H); **^{13}C NMR** (76 MHz, $CDCl_3$) δ 168.1, 140.2, 132.0, 131.7, 130.6, 129.6, 125.7, 51.8, 21.8; **HRMS** (ESI) for $C_9H_{10}O_2$: calculated m/z = 151.0759 (MH^+); found m/z = 151.0757 (MH^+).

Methyl 2-iodobenzoate (15a)[8]
Reaction conditions: According to the general procedure; 2-iodobenzoic acid (1 mmol, 248.0 mg), MeOH (0.5 mL), DBDMH (0.070 mmol, 20.0 mg), 70 °C, 20 h.

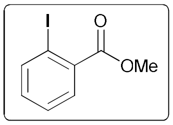

Purification: Not necessary.
Yield: 152 mg, 58% yield; colourless oil;
^{1}H NMR (300 MHz, $CDCl_3$) δ 7.95 (dd, J = 7.9, 1.1 Hz, 1H), 7.76 (dd, J = 7.8, 1.7 Hz, 1H), 7.36 (td, J = 7.6, 1.1 Hz, 1H), 7.11 (td, J = 7.7, 1.7 Hz, 1H), 3.90 (s, 3H); **^{13}C NMR** (76 MHz, $CDCl_3$) δ 166.9, 141.3, 135.1, 132.7, 130.9, 127.9, 94.1, 52.5; **HRMS** (ESI) for $C_8H_7IO_2$: calculated m/z = 262.9569 (MH^+); found m/z = 262.9563 (MH^+).

Methyl 3,4-dimethoxybenzoate (18a)[9]
Reaction conditions: According to the general procedure; 3,4-dimethoxybenzoic acid (1 mmol, 182.2 mg), MeOH (2 mL), DBDMH (0.070 mmol, 20.0 mg), 70 °C, 40 h.

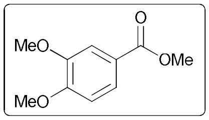

Purification: Not necessary.
Yield: 126 mg, 64% yield; white solid;
^{1}H NMR (300 MHz, $CDCl_3$) δ 7.67 (dd, *J* = 8.4, 2.0 Hz, 1H), 7.54 (d, *J* = 2.0 Hz, 1H), 6.88 (d, *J* = 8.4 Hz, 1H), 3.93 (s, 6H), 3.89 (s, 3H); **^{13}C NMR** (76 MHz, $CDCl_3$) δ 166.8, 152.9, 148.6, 123.5, 122.6, 111.9, 110.2, 55.9, 55.9, 51.9; **HRMS** (ESI) for $C_{10}H_{12}O_4$: calculated m/z = 197.0814 (MH^+); found m/z = 197.0809 (MH^+).

Methyl myristate (20a)[2]
Reaction conditions: According to the general procedure; myristic acid (1 mmol, 228.4 mg), MeOH (0.5 mL), DBDMH (0.070 mmol, 20.0 mg), 70 °C, 2 h.

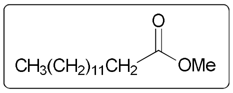

Purification: Not necessary.
Yield: 238 mg, 98% yield; colourless oil;
^{1}H NMR (300 MHz, $CDCl_3$) δ 3.66 (s, 3H), 2.30 (t, *J* = 7.5 Hz, 2H), 1.74 – 1.52 (m, 2H), 1.26 (s, 20H), 0.88 (t, *J* = 6.7 Hz, 3H); **^{13}C NMR** (76 MHz, $CDCl_3$) δ 174.5, 51.5, 34.2, 32.1, 29.8, 29.8, 29.7, 29.6, 29.5, 29.4, 29.3, 25.1, 22.8, 14.2, 11.2; **HRMS** (ESI) for $C_{15}H_{30}O_2$: calculated m/z = 243.2324 (MH^+); found m/z = 243.2318 (MH^+).

Methyl stearate (21a)[10]

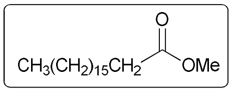

Reaction conditions: According to the general procedure; stearic acid (1 mmol, 284.5 mg), MeOH (0.5 mL), DBDMH (0.070 mmol, 20.0 mg), 70 °C, 2 h.

Purification: Not necessary.
Yield: 296 mg, 99% yield; white solid;
^{1}H NMR (300 MHz, $CDCl_3$) δ 3.67 (s, 3H), 2.30 (t, J = 7.5 Hz, 2H), 1.69 – 1.55 (m, 2H), 1.37 – 1.20 (m, 28H), 0.88 (t, J = 7.0 Hz, 3H); **^{13}C NMR** (76 MHz, $CDCl_3$) δ 174.5, 51.6, 34.3, 32.1, 29.8, 29.8, 29.8, 29.8, 29.8, 29.8, 29.8, 29.7, 29.6, 29.5, 29.4, 29.3, 25.1, 22.8, 14.3; **HRMS** (ESI) for $C_{19}H_{38}O_2$: calculated m/z = 299.2950 (MH^+); found m/z = 299.2957 (MH^+).

Methyl oleate (22a)[10]:
Reaction conditions: According to the general procedure; oleic acid (1 mmol, 315.6 µL), MeOH (2 mL), DBDMH (0.070 mmol, 20.0 mg), 70 °C, 5 h.
Purification: Not necessary.

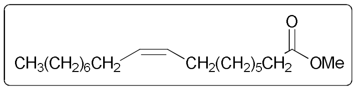

Yield: 291 mg, 98% yield; colourless oil;
^{1}H NMR (300 MHz, $CDCl_3$) δ 5.34 (ddd, J = 5.6, 3.5, 2.1 Hz, 2H), 3.66 (s, 3H), 2.30 (t, J = 7.5 Hz, 2H), 2.10 – 1.91 (m, 4H), 1.71 – 1.54 (m, 2H), 1.42 – 1.17 (m, 18H), 0.88 (t, J = 6.7 Hz, 3H); **^{13}C NMR** (76 MHz, $CDCl_3$) δ 174.4, 130.1, 129.9, 51.5, 34.2, 32.0, 29.9, 29.8, 29.7, 29.5, 29.4, 29.3, 29.3, 29.2, 27.3, 27.3, 25.1, 22.8, 14.2; **HRMS** (ESI) for $C_{19}H_{36}O_2$: calculated m/z = 297.2794 (MH^+); found m/z = 297.2798 (MH^+).

Methyl 4-(4-chlorophenyl)-4-oxobutanoate (23a)[11]
Reaction conditions: According to the general procedure; 3-(4-chlorobenzoyl)propionic acid (1 mmol, 212.6 mg), MeOH (0.5 mL), DBDMH (0.070 mmol, 20.0 mg), 70 °C, 20 h.
Purification: Not necessary.

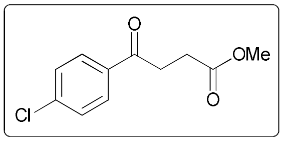

Yield: 224 mg of white solid (99%);
^{1}H NMR (300 MHz, $CDCl_3$) δ 7.90 – 7.81 (m, 2H), 7.41 – 7.33 (m, 2H), 3.64 (s, 3H), 3.22 (t, *J* = 6.6 Hz, 2H), 2.70 (t, *J* = 6.6 Hz, 2H); **^{13}C NMR** (76 MHz, $CDCl_3$) δ 196.8, 173.2, 139.6, 134.8, 129.4, 128.9, 51.8, 33.3, 27.9; **HRMS** (ESI) for $C_{12}H_{18}O_2$: calculated m/z = 249.0294 (MNa^+); found m/z = 249.0297 (MNa^+).

(*S*)-Methyl 2-acetamido-3-phenylpropanoate (24a)[12]
Reaction conditions: According to the general procedure; *N*-acetyl-*L*-phenylalanine (1 mmol, 207.2 mg), MeOH (0.5 mL), DBDMH (0.070 mmol, 20.0 mg), 70 °C, 20 h.
Purification: Not necessary.

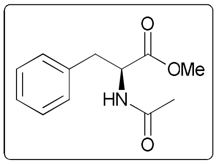

Yield: 210 mg of white solid (95%);
^{1}H NMR (300 MHz, $CDCl_3$) δ 7.33 – 7.18 (m, 3H), 7.18 – 7.05 (m, 2H), 6.38 (d, *J* = 7.5 Hz, 1H), 4.95 – 4.77 (m, 1H), 3.69 (s, 3H), 3.21 – 2.93 (m, 2H), 1.95 (s, 3H); **^{13}C NMR** (76 MHz, $CDCl_3$) δ 172.2, 169.8, 136.0, 129.2, 128.5, 127.0, 53.2, 52.2, 37.8, 22.9; **HRMS** (ESI) for $C_{12}H_{15}NO_3$: calculated m/z = 222.1130 (MH^+); found m/z = 222.1135 (MH^+).

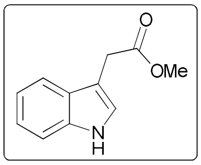

Methyl 2-(1H-indol-3-yl)acetate (25a)[13]
Reaction conditions: According to the general procedure; Heteroauxin (1 mmol, 175.2 mg), MeOH (0.5 mL), DBDMH (0.070 mmol, 20.0 mg), 70 °C, 20 h.
Purification: Not necessary.
Yield: Yield: 183 mg of brown oil (97%);
^{1}H NMR (300 MHz, $CDCl_3$) δ 8.16 (s, 1H), 7.56 (dd, *J* = 6.7, 1.6 Hz, 2H), 7.24 – 6.98 (m, 3H), 6.84 (d, *J* = 2.4 Hz, 1H), 3.72 (s, 2H), 3.63 (s, 3H); **^{13}C NMR** (76 MHz, $CDCl_3$) δ 173.0, 136.1, 127.1, 123.4, 122.0, 119.5, 118.6, 111.4, 107.8, 52.0, 31.1; **HRMS** (ESI) for $C_{11}H_{11}NO_2$: calculated m/z = 190.0868 (MH^+); found m/z = 190.0865 (MH^+).

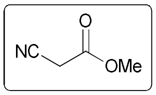

Methyl 2-cyanoacetate (26a)[14]
Reaction conditions: According to the general procedure; Cyanoacetic acid (1 mmol, 85.1 mg), MeOH (0.5 mL), DBDMH (0.070 mmol, 20.0 mg), 70 °C, 20 h.
Purification: Not necessary.
Yield: Yield: 96 mg of colourless oil (97%);
^{1}H NMR (300 MHz, $CDCl_3$) δ 3.76 (s, 3H), 3.46 (s, 2H); **^{13}C NMR** (76 MHz, $CDCl_3$) δ 163.6, 113.2, 53.5, 24.4; **HRMS** (ESI) for $C_4H_5NO_2$: calculated m/z = 100.0399 (MH^+); found m/z = 100.0398 (MH^+).

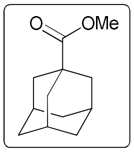

Adamantane-1-carboxylic acid methyl ester (27a)[5]
Reaction conditions: According to the general procedure; 1-adamantanecarboxylic acid (1 mmol, 180.2 mg), MeOH (0.5 mL), DBDMH (0.070 mmol, 20.0 mg), 70 °C, 20 h.
Purification: Not necessary.
Yield: 188 mg, 97% yield; white solid;
^{1}H NMR (300 MHz, $CDCl_3$) δ 3.60 (s, 3H), 2.01 – 1.92 (m, 3H), 1.88 – 1.80 (m, 6H), 1.74 – 1.59 (m, 3H); **^{13}C NMR** (76 MHz, $CDCl_3$) δ 178.08, 51.44, 40.64, 38.81, 36.46, 27.91; **HRMS** (ESI) for $C_{12}H_{18}O_2$: calculated m/z = 195.1385 (MH^+); found m/z = 195.1382 (MH^+).

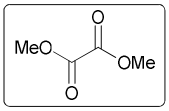

Dimethyl oxalate (28a)[15]
Reaction conditions: According to the general procedure; oxalic acid (1 mmol, 90.0 mg), MeOH (2 mL), DBDMH (0.070 mmol, 20.0 mg), 70 °C, 15 h.
Purification: Not necessary.
Yield: 112 mg, 95% yield; white solid;
^{1}H NMR (300 MHz, $CDCl_3$) δ 3.92 (s, 6H); **^{13}C NMR** (76 MHz, $CDCl_3$) δ 157.8, 53.5; **HRMS** (ESI) for $C_4H_6O_4$: calculated m/z = 119.0344 (MH^+); found m/z = 119.0342 (MH^+).

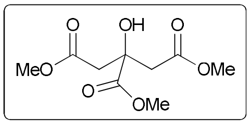

Trimethyl citrate (29a)[16]

Reaction conditions: According to the general procedure; malonic acid (1 mmol, 210.1 mg), MeOH (2 mL), DBDMH (0.070 mmol, 20.0 mg), 70 °C, 20 h.
Purification: Not necessary.
Yield: 220 mg, 94% yield; white solid;
^{1}H NMR (300 MHz, $CDCl_3$) δ 4.10 (s, 1H), 3.81 (s, 3H), 3.67 (s, 6H), 2.89 (d, J = 15.6 Hz, 2H), 2.79 (d, J = 15.6 Hz, 2H); **^{13}C NMR** (76 MHz, $CDCl_3$) δ 173.8, 170.2, 73.3, 53.2, 52.0, 43.1; **HRMS** (ESI) for $C_9H_{14}O_7$: calculated m/z = 235.0818 (MH^+); found m/z = 235.0815 (MH^+).

Cholic acid methyl ester (30a)[17]

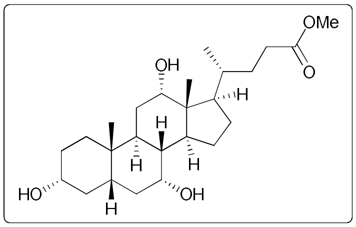

Molecular Weight: 422,60

The mixture of cholic acid (0.25 mmol, 102.1 mg), MeOH (2 mL) and DBDMH (0.018 mmol, 5.1 mg) was stirred in a 25 mL reactor tube at 70 °C for 5 h. After the completion of the reaction, the mixture was cooled to room temperature and MeOH was evaporated under the reduced pressure. The residue was dissolved in ethyl acetate and washed with the mixture of 1 mL of saturated $Na_2S_2O_{3(aq)}$, 1 mL of saturated $NaHCO_{3(aq)}$ and 10 mL of distilled water. The water phase was extracted with ethyl acetate (3 x 10 mL). The organic layers were combined, dried with Na_2SO_4 and the solvent was evaporated under the reduced pressure.
Purification: Not necessary.
Yield: 104 mg, 98% yield; white solid;
^{1}H NMR (300 MHz, $CDCl_3$) δ 3.95 (s, 1H), 3.83 (s, 1H), 3.66 (s, 3H), 3.55 (s, 3H), 3.49 – 3.32 (m, 1H), 2.48 – 1.07 (m, 24H), 0.98 (d, J = 5.8 Hz, 3H), 0.88 (s, 3H), 0.67 (s, 3H). **^{13}C NMR** (76 MHz, $CDCl_3$) δ 174.7, 72.9, 71.7, 68.3, 51.3, 46.8, 46.2, 41.4, 41.4, 39.3, 39.3, 35.2, 35.2 34.6, 34.6, 31.0, 30.8, 30.2, 28.0, 27.4, 26.1, 23.1, 22.3, 17.2, 12.3; **HRMS** (ESI) for $C_{25}H_{42}O_5$: calculated m/z = 423.3110 (MH^+); found m/z = 423.3128 (MH^+).

Hyodeoxycholic acid methyl ester (31a)[18]

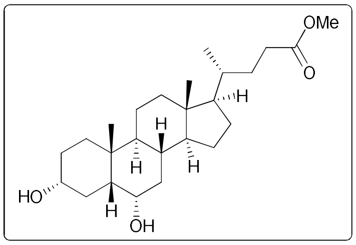

The mixture of hyodeoxycholic acid (0.25 mmol, 98.1 mg), MeOH (2 mL) and DBDMH (0.018 mmol, 5.1 mg) was stirred in a 25 mL reactor tube at 70 °C for 5 h. After the completion of the reaction, the mixture was cooled to room temperature and MeOH was evaporated under the reduced pressure. The residue was dissolved in ethyl acetate and washed with the mixture of 1 mL of saturated $Na_2S_2O_{3(aq)}$, 1 mL of saturated $NaHCO_{3(aq)}$ and 10 mL of distilled water. The water phase was extracted with ethyl acetate (3 x 10 mL). The organic layers were combined, dried with Na_2SO_4 and the solvent was evaporated under the reduced pressure.
Purification: Not necessary.
Yield: 86 mg, 85% yield; white solid;
^{1}H NMR (300 MHz, $CDCl_3$) δ 4.13 – 3.96 (m, 1H), 3.66 (s, 3H), 3.64 – 3.52 (m, 1H), 2.82 (s, 2H), 2.45 – 2.29 (m, 1H),

2.29 – 2.13 (m, 1H), 2.03 – 1.54 (m, 10H), 1.50 – 1.03 (m, 14H), 0.93 (s, 3H), 0.90 (s, 3H), 0.64 (s, 3H); **^{13}C NMR** (76 MHz, $CDCl_3$) δ 174.8, 71.5, 68.0, 56.3, 56.1, 51.6, 48.6, 42.9, 40.1, 40.0, 36.0, 35.7, 35.5, 34.9, 34.9, 31.2, 31.0, 30.2, 29.4, 28.2, 24.3, 23.6, 20.9, 18.3, 12.1; **HRMS** (ESI) for $C_{25}H_{42}O_4$: calculated m/z = 371.2950 ($M-H_2O+H^+$); found m/z = 371.2951 ($M-H_2O+H^+$).

Litocholic acid methyl ester (32a)[19]

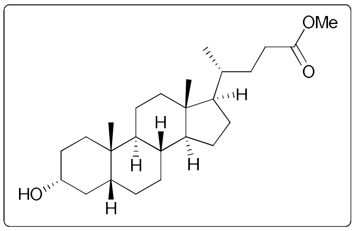

The mixture of litocholic acid (0.25 mmol, 94.1 mg), MeOH (2 mL) and DBDMH (0.018 mmol, 5.1 mg) was stirred in a 25 mL reactor tube at 70 °C for 5 h. After the completion of the reaction, the mixture was cooled to room temperature and MeOH was evaporated under the reduced pressure. The residue was dissolved in ethyl acetate and washed with the mixture of 1 mL of saturated $Na_2S_2O_{3(aq)}$, 1 mL of saturated $NaHCO_{3(aq)}$ and 10 mL of distilled water. The water phase was extracted with ethyl acetate (3 x 10 mL). The organic layers were combined, dried with Na_2SO_4 and the solvent was evaporated under the reduced pressure.

Purification: Not necessary.

Yield: 96 mg, 98% yield; white solid;

^{1}H NMR (300 MHz, $CDCl_3$) δ 3.66 (s, 3H), 3.60 (dt, *J* = 10.8, 4.7 Hz, 1H), 2.35 (ddd, *J* = 15.2, 10.1, 5.1 Hz, 1H), 2.29 – 2.15 (m, 1H), 2.06 (s, 1H), 2.00 – 1.00 (m, 26H), 0.92 (s, 3H), 0.90 (s, 3H), 0.64 (s, 3H). **^{13}C NMR** (76 MHz, $CDCl_3$) δ 174.8, 71.8, 56.5, 56.0, 51.5, 42.8, 42.2, 40.5, 40.2, 36.5, 35.9, 35.4, 35.4, 34.6, 31.1, 31.0, 30.5, 28.2, 27.3, 26.5, 24.3, 23.4, 20.9, 18.3, 12.1; **HRMS** (ESI) for $C_{25}H_{42}O_3$: calculated m/z = 371.3107 ($M-H_2O+H^+$); found m/z = 373.3102 ($M-H_2O+H^+$).

Dehydrocholic acid methyl ester (33a)[20]

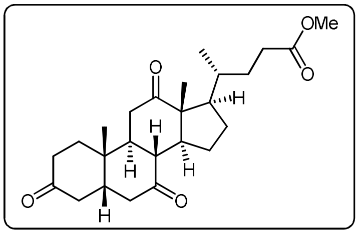

The mixture of dehydrocholic acid (0.25 mmol, 101.6 mg), MeOH (0.5 mL) and DBDMH (0.018 mmol, 5.1 mg) was stirred in a 25 mL reactor tube at 70 °C for 5 h. After the completion of the reaction, the mixture was cooled to room temperature and MeOH was evaporated under the reduced pressure. The residue was dissolved in ethyl acetate and first washed with the mixture of 1 mL of saturated $Na_2S_2O_{3(aq)}$, 1 mL of saturated $NaHCO_{3(aq)}$ and 10 mL of distilled water and then with 10 mL of 10% $HCl_{(aq)}$. The water phase was extracted with ethyl acetate (3 x 10 mL). The organic layers were combined, dried with Na_2SO_4 and the solvent was evaporated under the reduced pressure.

Purification: Not necessary.

Yield: 97 mg, 93% yield; white solid;

^{1}H NMR (300 MHz, $CDCl_3$) δ 3.66 (s, 3H), 3.01 – 2.75 (m, 3H), 2.51 – 1.16 (m, 21H), 1.41 (s, 3H), 1.08 (s, 3H), 0.85 (d, J = 6.5 Hz, 3H). **^{13}C NMR** (76 MHz, $CDCl_3$) δ 212.0, 209.1, 208.8, 174.5, 56.9, 51.8, 51.5, 49.0, 46.9, 45.7, 45.6, 45.0, 42.8,

38.7, 36.5, 36.1, 35.6, 35.3, 31.3, 30.5, 27.7, 25.2, 22.0, 18.7, 11.9; **HRMS** (ESI) for $C_{25}H_{36}O_5$: calculated m/z = 417.2641 (MH^+); requires m/z = 417.2644 (MH^+).

2-Fluoroethyl benzoate (1b)[21]

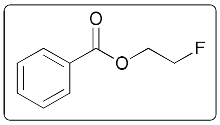

Reaction conditions: According to the general procedure; benzoic acid (1 mmol, 122.1 mg), 2-fluoroethanol (0.5 mL), DBDMH (0.07 mmol, 20.0 mg), 70 °C, 40 h.
Purification: Preparative TLC (CH_2Cl_2/MeOH = 200 : 1)
Yield: 161 mg, 96% yield; colourless oil;
^{1}H NMR (300 MHz, $CDCl_3$) δ 8.20 – 7.86 (m, 2H), 7.60 – 7.52 (m, 1H), 7.49 – 7.37 (m, 2H), 4.85 – 4.44 (m, 4H); **^{13}C NMR** (76 MHz, $CDCl_3$) δ 166.4, 133.3, 129.8, 128.5, 81.5 (d, J = 170.6 Hz), 63.9 (d, J = 20.2 Hz); **^{19}F NMR** (285 MHz, CDCl3) δ 5.03 (tt, J = 47.4, 28.6 Hz); **HRMS** (ESI) for $C_9H_9FO_2$: calculated m/z = 169.0665 (MH^+); found m/z = 169.0668 (MH^+).

2-Fluoroethyl octanoate (2b)[22]

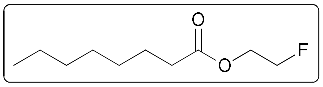

Reaction conditions: According to the general procedure; octanoic acid (1 mmol, 158.5 μL), 2-fluoroethanol (0.5 mL), DBDMH (0.07 mmol, 20.0 mg), 70 °C, 20 h.
Purification: Not necessary.
Yield: 175 mg, 92% yield; yellow oil;
^{1}H NMR (300 MHz, $CDCl_3$) δ 4.73 – 4.20 (m, 4H), 2.36 (t, J = 7.5 Hz, 2H), 1.71 – 1.57 (m, 2H), 1.42 – 1.19 (m, 8H), 0.88 (t, J = 7.1 Hz, 3H); **^{13}C NMR** (76 MHz, $CDCl_3$) δ 173.7, 81.5 (d, J = 170.3 Hz), 63.2 (d, J = 20.1 Hz), 34.2, 31.7, 29.1, 29.0, 25.0, 22.7, 14.1. **^{19}F NMR** (285 MHz, CDCl3) δ 4.86 (tt, J = 47.4, 28.7 Hz); **HRMS** (ESI) for $C_{10}H_{19}FO_2$: calculated m/z = 191.1447 (MH^+); found m/z = 191.1446 (MH^+).

Isopropyl benzoate (1c)[23]

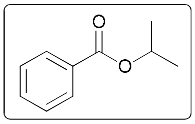

Reaction conditions: According to the general procedure; benzoic acid (1 mmol, 122.1 mg), isopropanol (0.5 mL), DBDMH (0.07 mmol, 20.0 mg), 70 °C, 20 h.
Purification: Not necessary.
Yield: 38 mg, 23% yield; colourless oil;
^{1}H NMR (300 MHz, $CDCl_3$) δ 8.09 – 7.99 (m, 2H), 7.59 – 7.50 (m, 1H), 7.47 – 7.38 (m, 2H), 5.26 (hept, J = 6.3 Hz, 1H), 1.37 (d, J = 6.3 Hz, 6H); **^{13}C NMR** (76 MHz, $CDCl_3$) δ 166.3, 132.8, 131.1, 129.6, 128.4, 68.5, 22.1.

Isopropyl octanoate (2c)[24]

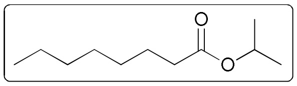

Reaction conditions: According to the general procedure; octanoic acid (1 mmol, 158.5 μL), isopropanol (0.5 mL DBDMH (0.07 mmol, 20.0 mg), 70 °C, 20 h.
Purification: Not necessary.
Yield: 160 mg, 86% yield; colourless oil;

¹H NMR (300 MHz, $CDCl_3$) δ 5.00 (hept, J = 6.3 Hz, 1H), 2.25 (t, J = 7.5 Hz, 2H), 1.67 – 1.55 (m, 2H), 1.35 – 1.25 (m, 8H), 1.23 (d, J = 6.3 Hz, 6H), 0.88 (t, J = 7.0 Hz, 3H); **¹³C NMR** (76 MHz, $CDCl_3$) δ 173.4, 67.3, 34.8, 31.7, 29.2, 29.0, 25.1, 22.7, 21.9, 14.1; **HRMS** (ESI) for $C_{11}H_{22}O_2$: calculated m/z = 187.1698 (MH^+); found m/z = 187.1703 (MH^+).

Cholic acid isopropyl ester (30c)[25]

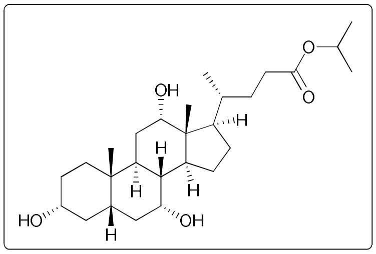

The mixture of cholic acid (0.25 mmol, 102.1 mg), *i*-PrOH (0.5 mL) and DBDMH (0.018 mmol, 5.1 mg) was stirred in a 25 mL reactor tube at 70 °C for 20 h. After the completion of the reaction, the mixture was cooled to room temperature and *i*-PrOH was evaporated under the reduced pressure. The residue was dissolved in ethyl acetate and washed with the mixture of 1 mL of saturated $Na_2S_2O_{3(aq)}$, 1 mL of saturated $NaHCO_{3(aq)}$ and 10 mL of distilled water. The water phase was extracted with ethyl acetate (3 x 10 mL). The organic layers were combined, dried with Na_2SO_4 and the solvent was evaporated under the reduced pressure.

Purification: Not necessary.

Yield: 97 mg, 86% yield; white solid;

¹H NMR (300 MHz, $CDCl_3$) δ 4.99 (hept, *J* = 6.1 Hz, 1H), 3.96 (s, 1H), 3.84 (s, 1H), 3.53 – 3.35 (m, 1H), 3.11 (s, 3H), 2.43 – 1.31 (m, 24H), 1.22 (d, *J* = 6.3 Hz, 6H), 0.98 (d, *J* = 6.0 Hz, 3H), 0.88 (s, 3H), 0.67 (s, 3H); **¹³C NMR** (76 MHz, $CDCl_3$) δ 174.0, 73.2, 72.0, 68.6, 67.5, 47.2, 46.6, 41.7, 41.6, 39.6, 35.4, 35.4, 34.9, 34.8, 31.8, 31.1, 30.5, 28.3, 27.6, 26.4, 23.3, 22.6, 22.0, 17.4, 12.6, 11.1; **HRMS** (ESI) for $C_{27}H_{46}O_5$: calculated m/z = 451.3424 (MH^+); found m/z = 451.3436 (MH^+).

Litocholic acid isopropyl ester (32c)[19]

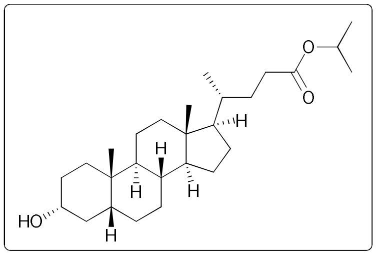

The mixture of litocholic acid (0.25 mmol, 94.1 mg), *i*-PrOH (0.5 mL) and DBDMH (0.018 mmol, 5.1 mg) was stirred in a 25 mL reactor tube at 70 °C for 20 h. After the completion of the reaction, the mixture was cooled to room temperature and *i*-PrOH was evaporated under the reduced pressure. The residue was dissolved in ethyl acetate and washed with the mixture of 1 mL of saturated $Na_2S_2O_{3(aq)}$, 2 mL of saturated $NaHCO_{3(aq)}$ and 10 mL of distilled water. The water phase was extracted with ethyl acetate (3 x 10 mL). The organic layers were combined, dried with Na_2SO_4 and the solvent was evaporated under the reduced pressure.

Purification: Not necessary.

Yield: 55 mg, 53% yield; white solid;

¹H NMR (300 MHz, $CDCl_3$) δ 5.08 – 4.93 (m, 1H), 3.71 – 3.56 (m, 1H), 2.44 – 2.10 (m, 2H), 2.02 – 1.27 (m, 21H), 1.27 – 1.18 (m, 6H), 1.19 – 0.97 (m, 6H), 0.95 – 0.87 (m, 6H), 0.64 (s, 3H); **¹³C NMR** (76 MHz, $CDCl_3$) δ 174.0, 72.0, 67.4, 56.6, 56.1, 42.8, 42.2, 40.5, 40.3, 36.5, 35.9, 35.5, 35.4, 34.7, 31.8, 31.1, 30.6, 28.3, 27.3, 26.5, 24.3, 23.5, 22.0, 20.9, 18.4, 12.1; **HRMS**

(ESI) for $C_{27}H_{46}O_3$: calculated m/z = 419.3498 (MH^+); found m/z = 419.3523 (MH^+).

***n*-Butyl benzoate (1d)[26]**

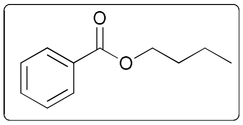

Reaction conditions: According to the general procedure; benzoic acid (1 mmol, 122.1 mg), *n*-butanol (0.5 mL), DBDMH (0.07 mmol, 20.0 mg), 70 °C, 20 h.
Purification: Preparative TLC (CH_2Cl_2/MeOH = 200 : 1)
Yield: 139 mg, 78% yield; colourless oil;
^{1}H NMR (300 MHz, $CDCl_3$) δ 8.10 – 7.99 (m, 2H), 7.58 – 7.49 (m, 1H), 7.42 (t, J = 7.5 Hz, 2H), 4.32 (t, J = 6.6 Hz, 2H), 1.84 – 1.66 (m, 2H), 1.56 – 1.39 (m, 2H), 0.98 (t, J = 7.4 Hz, 3H). **^{13}C NMR** (76 MHz, CDCl3) δ 166.7, 132.8, 130.6, 129.6, 128.4, 64.9, 30.9, 19.4, 13.8; **HRMS** (ESI) for $C_{11}H_{14}O_2$: calculated m/z = 179.1072 (MH^+); found m/z = 179.1070 (MH^+).

***n*-Butyl octanoate (2d)[27]**

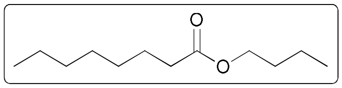

Reaction conditions: According to the general procedure; octanoic acid (1 mmol, 158.5 μL), *n*-butanol (1 mmol, 92 μL), DBDMH (0.07 mmol, 20.0 mg), 70 °C, 15 h.
Purification: Not necessary.
Yield: 190 mg, 95% yield; colourless oil;
^{1}H NMR (300 MHz, $CDCl_3$) δ 4.07 (t, J = 6.6 Hz, 2H), 2.29 (t, J = 7.5 Hz, 2H), 1.70 – 1.52 (m, 4H), 1.47 – 1.21 (m, 10H), 0.94 (t, J = 7.3 Hz, 3H), 0.87 (t, J = 6.9 Hz, 3H); **^{13}C NMR** (76 MHz, $CDCl_3$) δ 174.0, 64.1, 34.4, 31.8, 30.8, 29.2, 29.0, 25.1, 22.7, 19.2, 14.1, 13.7; **HRMS** (ESI) for $C_{12}H_{24}O_2$: calculated m/z = 201.1855 (MH^+); found m/z = 201.1857 (MH^+).

Cholic acid *n*-butyl ester (30d)[28]

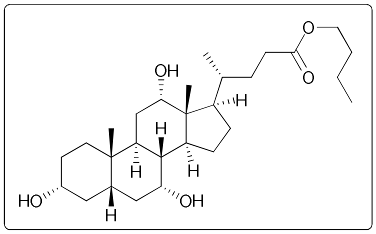

The mixture of cholic acid (0.25 mmol, 102.1 mg), *n*-butanol (0.5 mL) and DBDMH (0.018 mmol, 5.1 mg) was stirred in a 25 mL reactor tube at 70 °C for 20 h. After the completion of the reaction, the mixture was cooled to room temperature and MeOH was evaporated under the reduced pressure. The residue was dissolved in ethyl acetate and washed with the mixture of 1 mL of saturated $Na_2S_2O_{3(aq)}$, 1 mL of saturated $NaHCO_{3(aq)}$ and 10 mL of distilled water. The water phase was extracted with ethyl acetate (3 x 10 mL). The organic layers were combined, dried with Na_2SO_4 and the solvent was evaporated under the reduced pressure.
Purification: Not necessary.
Yield: 95 mg, 82% yield; white solid;
^{1}H NMR (300 MHz, $CDCl_3$) δ 4.06 (t, *J* = 6.6 Hz, 2H), 3.96 (s, 1H), 3.84 (s, 1H), 3.53 – 3.35 (m, 1H), 3.22 (s, 3H), 2.44 – 0.78 (m, 37H), 0.67 (s, 3H); **^{13}C NMR** (76 MHz, $CDCl_3$) δ 174.6, 73.2, 72.0, 68.6, 64.2, 47.1, 46.5, 41.7, 41.6, 39.6, 39.6, 35.4, 35.4, 34.9, 34.8, 31.5, 31.1, 30.8, 30.5, 28.3, 27.6, 26.4, 23.3, 22.6, 19.3, 17.4, 13.8, 12.6. **HRMS** (ESI) for $C_{28}H_{48}O_5$:

calculated m/z = 465.3580 (MH^+); found m/z = 465.3576 (MH^+).

Litocholic acid *n*-butyl ester (32d)[29]

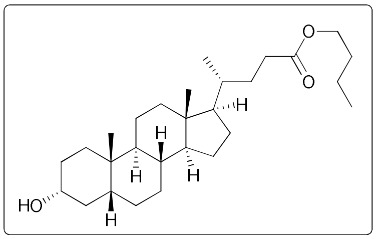

The mixture of litocholic acid (0.25 mmol, 94.1 mg), *n*-BuOH (0.5 mL) and DBDMH (0.018 mmol, 5.1 mg) was stirred in a 25 mL reactor tube at 70 °C for 20 h. After the completion of the reaction, the mixture was cooled to room temperature and *n*-BuOH was evaporated under the reduced pressure. The residue was dissolved in ethyl acetate and washed with the mixture of 1 mL of saturated $Na_2S_2O_{3(aq)}$, 1 mL of saturated $NaHCO_{3(aq)}$ and 10 mL of distilled water. The water phase was extracted with ethyl acetate (3 x 10 mL). The organic layers were combined, dried with Na_2SO_4 and the solvent was evaporated under the reduced pressure.

Purification: Not necessary.

Yield: 97 mg, 90% yield; white solid;

^{1}H NMR (300 MHz, $CDCl_3$) δ 4.06 (t, *J* = 6.7 Hz, 2H), 3.72 – 3.53 (m, 1H), 2.41 – 2.15 (m, 2H), 2.06 (s, 1H), 2.02 – 0.86 (m, 39H), 0.64 (s, 3H); **^{13}C NMR** (76 MHz, $CDCl_3$) δ 174.6, 72.0, 64.2, 56.6, 56.1, 42.8, 42.2, 40.5, 40.3, 36.5, 36.0, 35.5, 35.5, 34.7, 31.4, 31.1, 30.8, 30.6, 28.3, 27.3, 26.5, 24.3, 23.5, 20.9, 19.3, 18.4, 13.8, 12.1; **HRMS** (ESI) for $C_{28}H_{48}O_3$: calculated m/z = 415.3576 ($M-H_2O+H^+$); found m/z = 415.3577 ($M-H_2O+H^+$).

Dehydrocholic acid *n*-butyl ester (33d)[30]

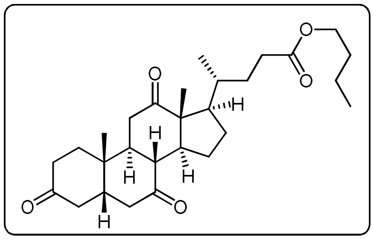

The mixture of dehydrocholic acid (0.25 mmol, 101.6 mg), *n*-BuOH (0.5 mL) and DBDMH (0.018 mmol, 5.1 mg) was stirred in a 25 mL reactor tube at 70 °C for 20 h. After the completion of the reaction, the mixture was cooled to room temperature and *n*-BuOH was evaporated under reduced pressure. The residue was dissolved in ethyl acetate and first washed with the mixture of 1 mL of saturated $Na_2S_2O_{3(aq)}$, 2 mL of saturated $NaHCO_{3(aq)}$ and 10 mL of distilled water and then with 10 mL of 10% $HCl_{(aq)}$. The water phase was extracted with ethyl acetate (3 x 10 mL). The organic layers were combined, dried with Na_2SO_4 and the solvent was evaporated under the reduced pressure.

Purification: Not necessary.

Yield: 50 mg, 44% yield; white solid;

^{1}H NMR (300 MHz, $CDCl_3$) δ 4.07 (t, *J* = 6.6 Hz, 2H), 3.03 – 2.76 (m, 3H), 2.53 – 1.18 (m, 28H), 1.07 (s, 3H), 0.93 (t, *J* = 7.3 Hz, 3H), 0.85 (d, *J* = 6.5 Hz, 3H). **^{13}C NMR** (76 MHz, $CDCl_3$) δ 212.0, 209.1, 208.8, 174.3, 64.2, 57.0, 51.9, 49.1, 46.9, 45.8, 45.6, 45.1, 42.9, 38.7, 36.6, 36.1, 35.6, 35.4, 31.6, 30.8, 30.6, 27.7, 25.2, 22.0, 19.2, 18.7, 13.8, 11.9; **HRMS** (ESI) for $C_{28}H_{42}O_5$: calculated m/z = 459.3110 (MH^+); requires m/z = 459.3109 (MH^+).

***n*-Octyl benzoate (1f)[2]**

Reaction conditions: According to the general procedure; benzoic acid (1 mmol, 122.1 mg), *n*-octanol (1 mmol, 158 µL), DBDMH (0.07 mmol, 20.0 mg), 70 °C, 20 h.

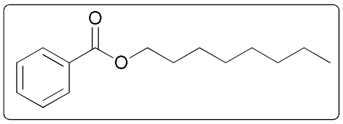

Purification: Preparative TLC (CH_2Cl_2/Hexane = 1 : 1)

Yield: 152 mg, 65% yield; white solid;

^{1}H NMR (300 MHz, CDCl3) δ 8.10 – 8.00 (m, 2H), 7.59 – 7.51 (m, 1H), 7.47 – 7.39 (m, 2H), 4.31 (t, J = 6.7 Hz, 2H), 1.83 – 1.70 (m, 2H), 1.51 – 1.21 (m, 10H), 0.88 (t, *J* = 6.6 Hz, 3H); **^{13}C NMR** (76 MHz, $CDCl_3$) δ 166.8, 132.9, 130.7, 129.7, 128.4, 65.3, 31.9, 29.4, 29.3, 28.9, 26.2, 22.8, 14.2; **HRMS** (ESI) for $C_{15}H_{22}O_2$: calculated m/z = 235.1698 (MH^+); found m/z = 235.1697 (MH^+).

***n*-Octyl octanoate (2f)[31]**

Reaction conditions: According to the general procedure; octanoic acid (1 mmol, 158.5 µL), *n*-octanol (1 mmol, 158 µL), DBDMH (0.07 mmol, 20.0 mg), 70 °C, 20 h.

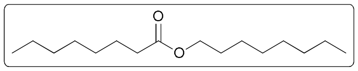

Purification: Preparative TLC (CH_2Cl_2/MeOH = 200 : 1)

Yield: 226 mg, 88% yield; colourless oil;

^{1}H NMR (300 MHz, $CDCl_3$) δ 4.06 (t, J = 6.7 Hz, 2H), 2.28 (t, J = 7.5 Hz, 2H), 1.68 – 1.54 (m, 4H), 1.39 – 1.19 (m, 18H), 0.88 (t, J = 6.3 Hz, 6H); **^{13}C NMR** (76 MHz, $CDCl_3$) δ 173.9, 64.4, 34.4, 31.9, 31.8, 29.3, 29.3, 29.2, 29.0, 28.8, 26.0, 25.1, 22.7, 22.7, 14.1, 14.1; **HRMS** (ESI) for $C_{16}H_{32}O_2$: calculated m/z = 257.2481 (MH^+); found m/z = 257.2486 (MH^+).

Cyclopentyl benzoate (1g)[32]

Reaction conditions: According to the general procedure; benzoic acid (1 mmol, 122.1 mg), cyclopentanol (1.1 mmol, 100 µL), DBDMH (0.07 mmol, 20.0 mg), 70 °C, 20 h.

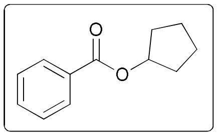

Purification: Column chromatography (EtOAc/Hexane = 1:10)

Yield: 36 mg, 19% yield; colourless oil;

^{1}H NMR (300 MHz, CDCl3) δ 8.02 (dt, *J* = 7.1, 1.4 Hz, 2H), 7.57 – 7.49 (m, 1H), 7.46 – 7.37 (m, 2H), 5.41 (tt, *J* = 6.1, 2.8 Hz, 1H), 2.12 – 1.51 (m, 8H); **^{13}C NMR** (76 MHz, $CDCl_3$) δ 166.4, 132.7, 131.0, 129.6, 128.3, 77.7, 32.9, 23.9; **HRMS** (ESI) for $C_{12}H_{14}O_2$: calculated m/z = 213.0891 (MNa^+); found m/z = 213.0894 (MNa^+).

Cyclopentyl octanoate (2g)[33]

Reaction conditions: According to the general procedure; octanoic acid (1 mmol, 158.5 µL), cyclopentanol (1 mmol, 91 µL), DBDMH (0.07 mmol, 20.0 mg), 70 °C, 15 h.

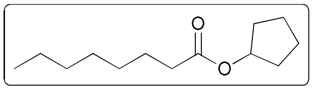

Purification: Not necessary.

Yield: 200 mg, 94% yield; colourless oil;

^{1}H NMR (300 MHz, $CDCl_3$) δ 5.16 (tt, J = 5.9, 2.6 Hz, 1H), 2.25 (t, J = 7.5 Hz, 2H), 1.95 – 1.47 (m, 10H), 1.41 – 1.20 (m, 8H), 0.88 (d, J = 7.0 Hz, 3H); **^{13}C NMR** (76 MHz, $CDCl_3$) δ

173.8, 76.8, 34.8, 32.8, 31.8, 29.2, 29.0, 25.2, 23.8, 22.7, 14.1; **HRMS** (ESI) for $C_{13}H_{24}O_2$: calculated m/z = 213.1855 (MH^+); found m/z = 213.1860 (MH^+).

Adamantan-1-ylmethyl benzoate (1j)[34]

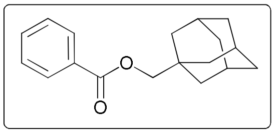

Reaction conditions: According to the general procedure; benzoic acid (1 mmol, 122.1 mg), 1-adamantanemethanol (2.0 mmol, 332.5 mg), DBDMH (0.07 mmol, 20.0 mg), 70 °C, 20 h.
Purification: Column chromatography (EtOAc/Hexane = 1:10)
Yield: 203 mg, 75% yield; white solid;
^{1}H NMR (300 MHz, CDCl3) δ 8.06 (dt, *J* = 7.1, 1.4 Hz, 2H), 7.60 – 7.51 (m, 1H), 7.49 – 7.40 (m, 2H), 3.92 (s, 2H), 2.32 – 1.28 (m, 15H); **^{13}C NMR** (76 MHz, $CDCl_3$) δ 166.8, 132.9, 130.7, 129.6, 128.4, 74.5, 39.5, 37.1, 33.6, 28.2; **HRMS** (ESI) for $C_{18}H_{22}O_2$: calculated m/z = 271.1698 (MH^+); found m/z = 271.1704 (MH^+).

Adamantan-1-ylmethyl octanoate (2j)[35]

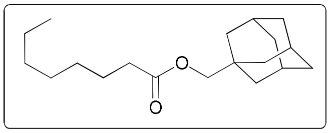

Reaction conditions: According to the general procedure; octanoic acid (1 mmol, 158.5 μL), 1-adamantanemethanol (2.0 mmol, 332.5 mg), DBDMH (0.07 mmol, 20.0 mg), 70 °C, 20 h.
Purification: Not necessary.
Yield: 275 mg, 94% yield; colourless oil;
^{1}H NMR (300 MHz, $CDCl_3$) δ 3.67 (s, 2H), 2.31 (t, *J* = 7.5 Hz, 2H), 2.07 – 1.87 (m, 3H), 1.84 – 1.21 (m, 22H), 0.94 – 0.80 (m, 3H); **^{13}C NMR** (76 MHz, $CDCl_3$) δ 174.2, 73.9, 39.4, 37.1, 34.5, 33.3, 31.8, 29.3, 29.1, 28.2, 25.2, 22.7, 14.2; **HRMS** (ESI) for $C_{19}H_{32}O_2$: calculated m/z = 293.2481 (MH^+); found m/z = 293.2486 (MH^+).

3β-O-Acetyl-cholesterol (34k)[36]

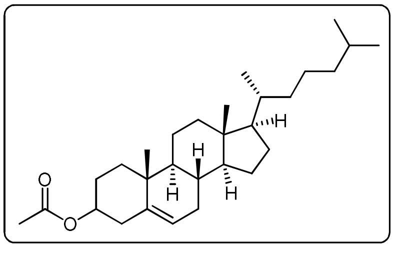

The mixture of cholesterol (0.25 mmol, 96.6 mg), EtOAc (1 mL) and DBDMH (0.018 mmol, 5.1 mg) was stirred in a 25 mL reactor tube at 70 °C for 20 h. After the completion of the reaction, the mixture was cooled to room temperature and additional 10 mL of ethyl acetate was added. The solution was washed with the mixture of 1 mL of saturated $Na_2S_2O_{3(aq)}$, 2 mL of saturated $NaHCO_{3(aq)}$ and 10 mL of distilled water. The water phase was extracted with ethyl acetate (3 x 10 mL). The organic layers were combined, dried with Na_2SO_4 and the solvent was evaporated under the reduced pressure.
Purification: Column chromatography (EtOAc/Hexane = 1:5)
Yield: 84 mg, 78% yield; white solid;
^{1}H NMR (300 MHz, $CDCl_3$) δ 5.37 (d, *J* = 4.6 Hz, 1H), 4.73 – 4.48 (m, 1H), 2.32 (d, *J* = 7.6 Hz, 2H), 2.03 (s, 3H), 2.01 – 0.79 (m, 38H), 0.68 (s, 3H); **^{13}C NMR** (76 MHz, $CDCl_3$) δ 170.6, 139.8, 122.8, 74.1, 56.8, 56.3, 50.2, 42.4, 39.9, 39.7, 38.3, 37.1,

36.7, 36.3, 35.9, 32.0, 32.0, 28.4, 28.1, 27.9, 24.4, 24.0, 23.0, 22.7, 21.6, 21.2, 19.4, 18.9, 12.0.

3β-Acetyloxy-5α-androstan-17-on (34l)[37]

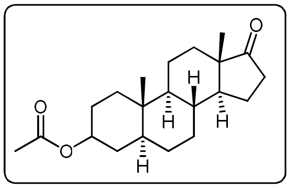

The mixture of epi-androsterone (0.5 mmol, 145.2 mg), EtOAc (1 mL) and DBDMH (0.018 mmol, 5.1 mg) was stirred in a 25 mL reactor tube at 70 °C for 20 h. After the completion of the reaction, the mixture was cooled to room temperature and additional 10 mL of ethyl acetate was added. The solution was washed with the mixture of 1 mL of saturated $Na_2S_2O_{3(aq)}$, 2 mL of saturated $NaHCO_{3(aq)}$ and 10 mL of distilled water. The water phase was extracted with ethyl acetate (3 x 10 mL). The organic layers were combined, dried with Na_2SO_4 and the solvent was evaporated under the reduced pressure.
Purification: Column chromatography (EtOAc/Hexane = 1:3)
Yield: 116 mg, 70% yield; white solid;
^{1}H NMR (300 MHz, $CDCl_3$) δ 4.69 (ddd, *J* = 16.3, 11.2, 4.9 Hz, 1H), 2.43 (dd, *J* = 18.7, 8.7 Hz, 1H), 2.10 (t, *J* = 9.0 Hz, 1H), 2.02 (s, 3H), 1.98 – 0.92 (m, 19H), 0.86 (s, 3H), 0.85 (s, 3H), 0.71 (tt, *J* = 12.1, 6.0 Hz, 1H); **^{13}C NMR** (76 MHz, $CDCl_3$) δ 170.7, 73.6, 54.4, 51.5, 47.9, 44.8, 36.8, 35.9, 35.7, 35.1, 34.0, 31.6, 30.9, 28.4, 27.5, 21.9, 21.5, 20.6, 13.9, 12.3; **HRMS** (ESI) for $C_{21}H_{32}O_3$: calculated m/z = 333.2430 (MH^+); found m/z = 333.2417 (MH^+).

(*E*)-2-ethylhex-2-enal (35m)[38]

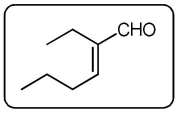

The mixture of butanal (2.0 mmol, 180.2 μL) and DBDMH (0.14 mmol, 40 mg) was stirred in a 25 mL reactor tube at 80 °C for 45 min. After the completion of the reaction, the mixture was cooled to room temperature and dissolved in 10 mL of EtOAc. The solution was washed with the mixture of 1 mL of saturated $Na_2S_2O_{3(aq)}$, 1 mL of saturated $NaHCO_{3(aq)}$ and 10 mL of distilled water. The water phase was extracted with ethyl acetate (3 x 10 mL). The organic layers were combined, dried with Na_2SO_4 and the solvent was evaporated under the reduced pressure.
Purification: Not necessary.
Yield: 114 mg, 90% yield; dark oil;
^{1}H NMR (300 MHz, $CDCl_3$) δ 9.37 (s, 1H), 6.43 (t, *J* = 7.5 Hz, 1H), 2.40 – 2.22 (m, 4H), 1.55 (m, 2H), 0.98 (t, *J* = 7.4 Hz, 3H), 0.97 (t, *J* = 7.6 Hz, 3H); **^{13}C NMR** (76 MHz, CCl_3) δ 195.2, 154.7, 145.5, 30.8, 22.1, 17.4, 14.0, 13.4.

(*E*)-2-butyloct-2-enal (36m)[39]

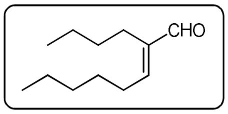

The mixture of hexanal (2.0 mmol, 244.2 μL) and DBDMH (0.14 mmol, 40 mg) was stirred in a 25 mL reactor tube at 80 °C for 45 min. After the completion of the reaction, the mixture was cooled to room temperature and dissolved in 10 mL of EtOAc. The solution was washed with the mixture of 1 mL of saturated $Na_2S_2O_{3(aq)}$, 1 mL of saturated $NaHCO_{3(aq)}$ and 10 mL

of distilled water. The water phase was extracted with ethyl acetate (3 x 10 mL). The organic layers were combined, dried with Na_2SO_4 and the solvent was evaporated under the reduced pressure.
Purification: Not necessary.
Yield: 171 mg, 94% yield; dark oil;
^{1}H NMR (300 MHz, $CDCl_3$) δ 9.36 (s, 1H), 6.45 (t, *J* = 7.4 Hz, 1H), 2.35 (q, *J* = 7.4 Hz, 2H), 2.24 (t, *J* = 7.3 Hz, 2H), 1.60 – 1.15 (m, 10H), 0.91 (td, *J* = 6.9, 4.4 Hz, 6H); **^{13}C NMR** (76 MHz, CCl_3) δ 195.4, 155.4, 143.9, 31.6, 31.0, 28.9, 28.4, 23.8, 22.8, 22.5, 14.0, 13.9.

(*E*)-2-pentylnon-2-enal (37m)[40]

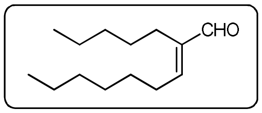

The mixture of heptanal (2.0 mmol, 279.6 μL) and DBDMH (0.14 mmol, 40 mg) was stirred in a 25 mL reactor tube at 80 °C for 1.5 h. After the completion of the reaction, the mixture was cooled to room temperature and dissolved in 10 mL of EtOAc. The solution was washed with the mixture of 1 mL of saturated $Na_2S_2O_{3(aq)}$, 1 mL of saturated $NaHCO_{3(aq)}$ and 10 mL of distilled water. The water phase was extracted with ethyl acetate (3 x 10 mL). The organic layers were combined, dried with Na_2SO_4 and the solvent was evaporated under the reduced pressure.
Purification: Not necessary.
Yield: 200 mg, 95% yield; dark oil;
^{1}H NMR (300 MHz, $CDCl_3$) δ 9.36 (s, 1H), 6.54 – 6.27 (m, 1H), 2.35 (q, *J* = 7.4 Hz, 2H), 2.28 – 2.17 (m, 2H), 1.75 – 1.12 (m, 14H), 0.99 – 0.74 (m, 6H); **^{13}C NMR** (76 MHz, CCl_3) δ 195.3, 155.3, 143.9, 31.9, 31.6, 29.1, 28.9, 28.7, 28.5, 24.0, 22.6, 22.5, 14.0, 13.9.

(*E*)-2-octyldodec-2-enal (38m)[41]

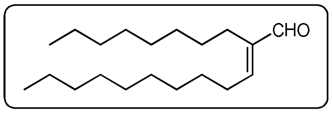

The mixture of decanal (2.0 mmol, 376.6 μL) and DBDMH (0.14 mmol, 40 mg) was stirred in a 25 mL reactor tube at 80 °C for 1.5 h. After the completion of the reaction, the mixture was cooled to room temperature and dissolved in 10 mL of EtOAc. The solution was washed with the mixture of 1 mL of saturated $Na_2S_2O_{3(aq)}$, 1 mL of saturated $NaHCO_{3(aq)}$ and 10 mL of distilled water. The water phase was extracted with ethyl acetate (3 x 10 mL). The organic layers were combined, dried with Na_2SO_4 and the solvent was evaporated under the reduced pressure.
Purification: Not necessary.
Yield: 265 mg, 90% yield; dark oil;
^{1}H NMR (300 MHz, $CDCl_3$) δ 9.36 (s, 1H), 6.50 – 6.37 (m, 1H), 2.35 (q, *J* = 7.4 Hz, 2H), 2.26 – 2.20 (m, 2H), 1.68 – 1.12 (m, 26H), 0.88 (q, *J* = 4.5 Hz, 6H); **^{13}C NMR** (76 MHz, CCl_3) δ 195.3, 155.3, 143.9, 31.9, 30.3, 29.8, 29.6, 29.5, 29.5, 29.5, 29.4, 29.3, 29.2, 29.0, 28.8, 28.8, 24.1, 22.7, 14.1, 14.1.

(*E*)-2-decyltetradec-2-enal (39m)[42]

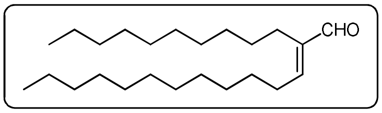

The mixture of dodecanal (2.0 mmol, 443.6 μL) and DBDMH (0.14 mmol, 40 mg) was stirred in a 25 mL reactor tube at 80 °C for 1.5 h. After the completion of the reaction, the mixture was cooled to room temperature and dissolved in 10 mL of EtOAc. The solution was washed with the mixture of 1 mL of saturated $Na_2S_2O_{3(aq)}$, 1 mL of saturated $NaHCO_{3(aq)}$ and 10 mL of distilled water. The water phase was extracted with ethyl acetate (3 x 10 mL). The organic layers were combined, dried with Na_2SO_4 and the solvent was evaporated under the reduced pressure.

Purification: Not necessary.

Yield: 340 mg, 92% yield; dark oil;

^{1}H NMR (300 MHz, $CDCl_3$) δ 9.35 (s, 1H), 6.43 (t, *J* = 7.4 Hz, 1H), 2.42 – 2.27 (m, 2H), 2.27 – 2.15 (m, 2H), 1.70 – 1.01 (m, 34H), 0.88 (t, *J* = 6.4 Hz, 6H); **^{13}C NMR** (76 MHz, CCl_3) δ 195.3, 155.3, 144.0, 32.0, 29.8, 29.7, 29.6, 29.6, 29.6, 29.5, 29.5, 29.5, 29.4, 29.4, 29.2, 29.0, 28.9, 28.9, 28.8, 28.7, 24.1, 22.8, 14.2, 14.2.

(*E*)-2-benzylideneheptanal (40m)[40]

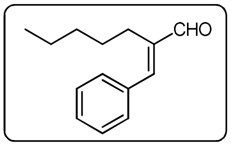

Heptanal (1.0 mmol, 140.0 μL) was added dropwise to the mixture of benzaldehyde (3.0 mmol, 306.1 μL) and DBDMH (0.07 mmol, 20 mg), stirring in a 25 mL reactor tube at 80 °C over a time period of 20 min and the reaction mixture was heated for additional 45 min. After the completion of the reaction, the mixture was cooled to room temperature and dissolved in 10 mL of EtOAc. The solution was washed with the mixture of 1 mL of saturated $Na_2S_2O_{3(aq)}$, 1 mL of saturated $NaHCO_{3(aq)}$ and 10 mL of distilled water. The water phase was extracted with ethyl acetate (3 x 10 mL). The organic layers were combined, dried with Na_2SO_4 and the solvent was evaporated under the reduced pressure.

Purification: Preparative TLC (Hexane/EtOAc = 9 : 1)

Yield: 10 mg, 5% yield; yellow oil;

^{1}H NMR (300 MHz, $CDCl_3$) δ 9.55 (s, 1H), 7.55 – 7.35 (m, 5H), 7.21 (s, 1H), 2.59 – 2.46 (m, 2H), 1.55 – 1.43 (m, 2H), 1.43 – 1.29 (m, 4H), 0.89 (t, *J* = 7.0 Hz, 3H); **^{13}C NMR** (76 MHz, CCl_3) δ 195.9, 149.9, 143.6, 135.2, 129.8, 129.7, 128.9, 32.2, 28.1, 24.9, 22.6, 14.2.

D. Scale-up procedure for preparation of methyl benzoate (1a) and isolation of 5,5-dimethylhydantoin

The mixture of benzoic acid (30 mmol, 3.66 g), MeOH (15 mL) and 1,3-dibromo-5,5-dimethylhydantoin (2.10 mmol, 0.60 g) was stirred in a 25 mL reactor tube at 70 °C for 20 h. After the completion of the reaction, the mixture was cooled to room temperature and alcohol was evaporated under reduced pressure. The residue was dissolved in 100 mL of ethyl acetate

and washed with water (3 x 30 mL). The water layers were combined, concentrated to the volume of 8 mL by rotary evaporation and again extracted with ethyl acetate (3 x 30 mL). The organic layers were combined, dried over Na_2SO_4 and the solvent was evaporated under reduced pressure to yield 5,5-dimethylhydantoin. The organic layer from the first washing of the crude reaction mixture was washed with the mixture of 20 mL of saturated $NaHCO_3$ (aq), 20 mL of 10% $Na_2S_2O_3$ (aq) and 100 mL of distilled water. The water layer was extracted with ethyl acetate (2 x 100 mL). The organic layers were combined, dried over Na_2SO_4 and the solvent was evaporated under reduced pressure to furnish methyl benzoate as colourless oil.

Yield (methyl benzoate): 3.88 g, 95%.

Yield (5,5-dimethylhydantoin)[43]: 227 mg, 85%.

^{1}H NMR (300 MHz, $CDCl_3$) δ 9.77 (s, 1H), 7.07 (s, 1H), 1.42 (s, 6H).

^{13}C NMR (76 MHz, $CDCl_3$) δ 179.0, 157.0, 60.0, 24.8.

HRMS (ESI) for $C_5H_8N_2O_2$: calculated m/z = 129.0664 (MH^+); found m/z = 129.0666 (MH^+).

E. Scale-up procedure for preparation of methyl citrate (29a)

The mixture of citric acid (30 mmol, 5.76 g), MeOH (60 mL) and DBDMH (0.70 mmol, 0.600 g) was stirred in a 25 mL reactor tube at 70 °C for 20 h. After the completion of the reaction, the mixture was cooled to room temperature and alcohol was evaporated under reduced pressure. The residue was dissolved in 100 mL of ethyl acetate, washed with the mixture of 10 mL of saturated $NaHCO_3$ (aq), 10 mL of saturated $Na_2S_2O_3$ (aq) and 50 mL of distilled water and the water phase was extracted with ethyl acetate (2 x 100 mL). The organic layers were combined, dried with Na_2SO_4 and the solvent was evaporated under reduced pressure to furnish methyl citrate as a white solid. Yield: 6.53 g, 93%.

F. Scale-up procedure for preparation of methyl stearate (21a)

The mixture of stearic acid (30 mmol, 8.53 g), MeOH (15 mL) and DBDMH (0.70 mmol, 0.600 g) was stirred in a 25 mL reactor tube at 70 °C for 2 h. After the completion of the reaction, the mixture was cooled to room temperature and alcohol was evaporated under reduced pressure. The residue was dissolved in 100 mL of ethyl acetate, washed with the mixture of 20 mL of saturated $NaHCO_3$ (aq), 20 mL of 10% $Na_2S_2O_3$ (aq) and 100 mL of distilled water and the water phase was extracted with ethyl acetate (2 x 100 mL). The organic layers were combined, washed with distilled water (2 x 20 mL), dried with Na_2SO_4 and the solvent was evaporated under reduced pressure to furnish methyl stearate as a white solid. Yield: 8.96 g, 100%.

G. Scale-up procedure for preparation of cholic acid methyl ester (30a)

The mixture of cholic acid (2.0 mmol, 0.814 g), MeOH (8 mL) and DBDMH (0.14 mmol, 40 mg) was stirred in a 25 mL reactor tube at 70 °C for 5 h. After the completion of the reaction, the mixture was cooled to room temperature and alcohol was evaporated under reduced pressure. The residue was dissolved in 50 mL of ethyl acetate, washed with the mixture of 5 mL of saturated $NaHCO_{3\ (aq)}$, 5 mL of 10% $Na_2S_2O_{3\ (aq)}$ and 20 mL of distilled water and the water phase was extracted with ethyl acetate (2 x 50 mL). The organic layers were combined, washed with distilled water (2 x 20 mL), dried with Na_2SO_4 and the solvent was evaporated under reduced pressure to furnish cholic acid methyl ester as white solid. Yield: 0.845 g, 100%.

H. Copies of ^{1}H, ^{13}C and ^{19}F NMR spectra

Methyl benzoate (1a)

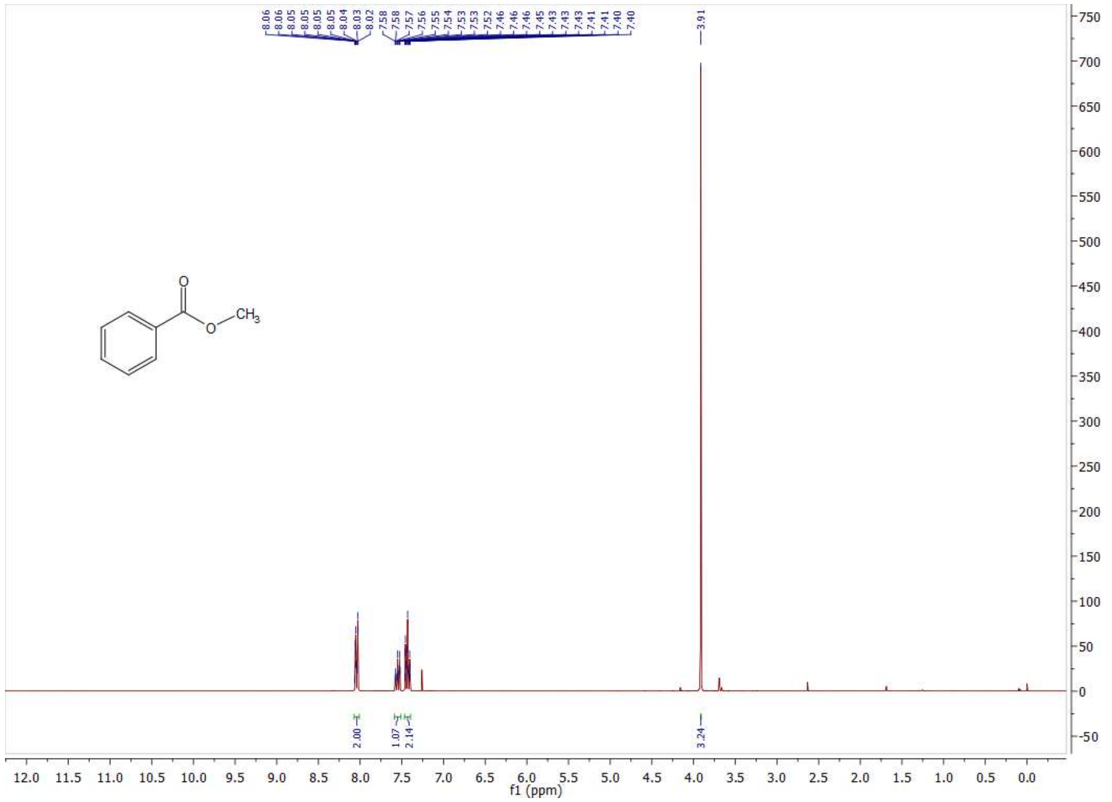

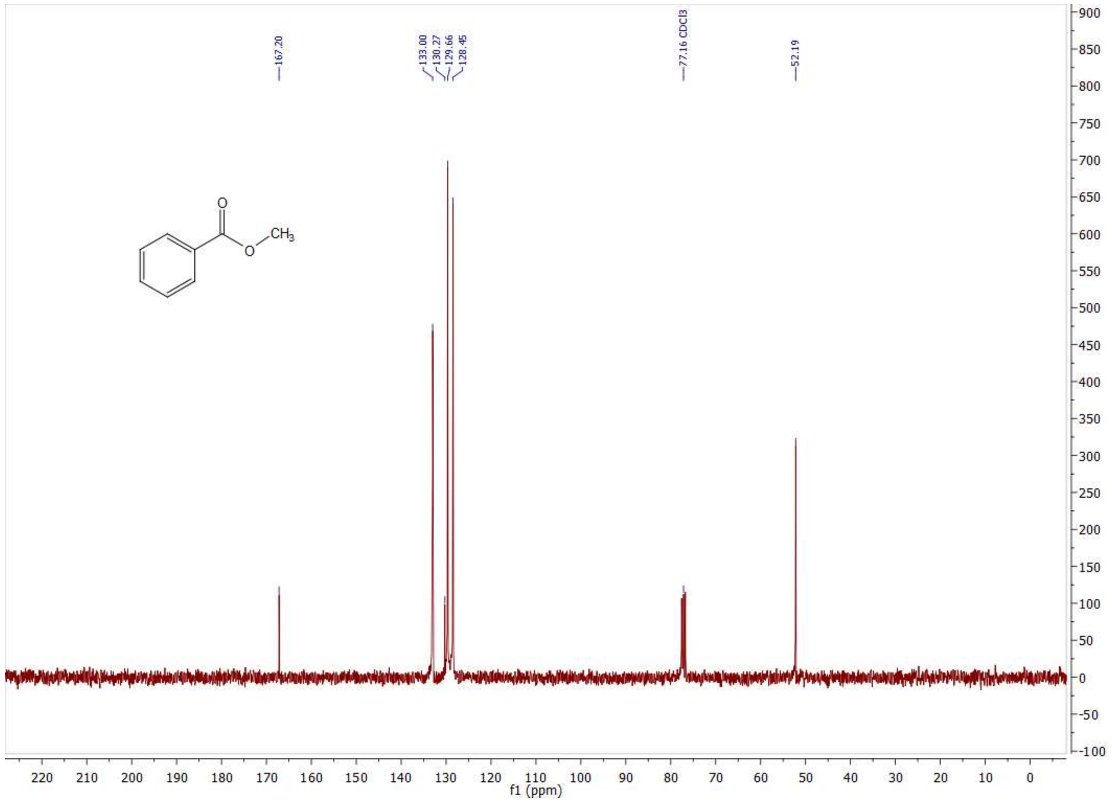

Methyl octanoate (2a)

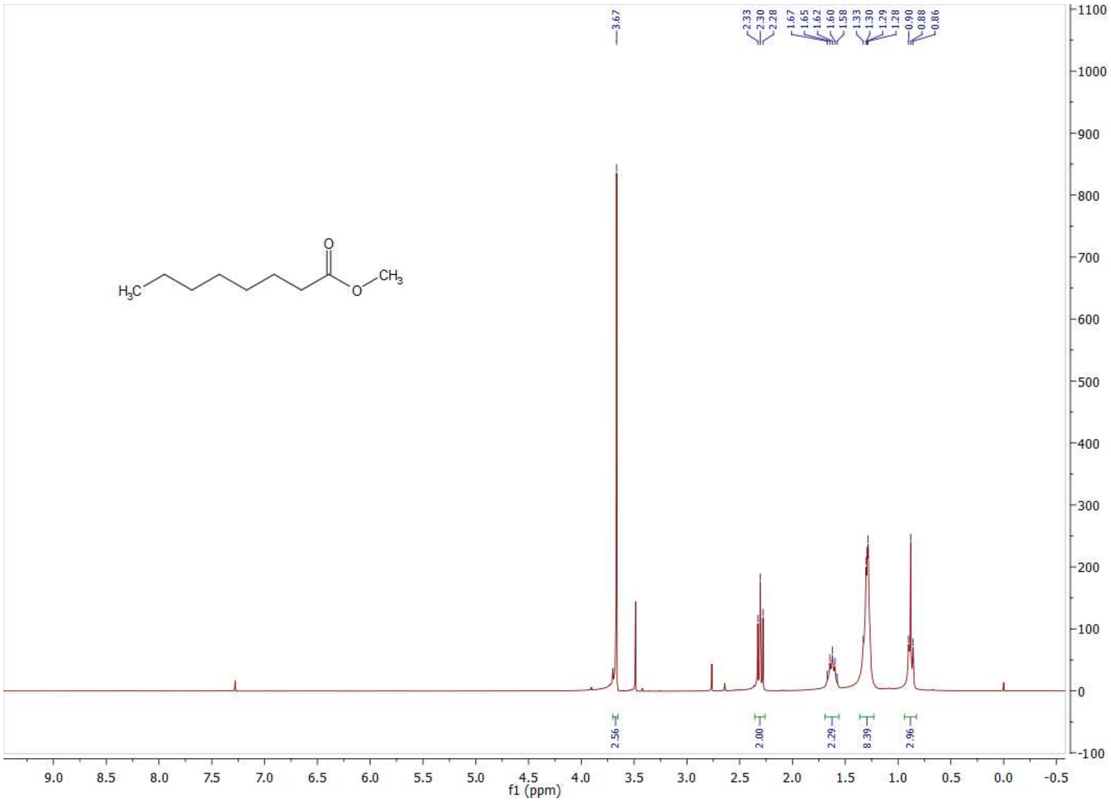

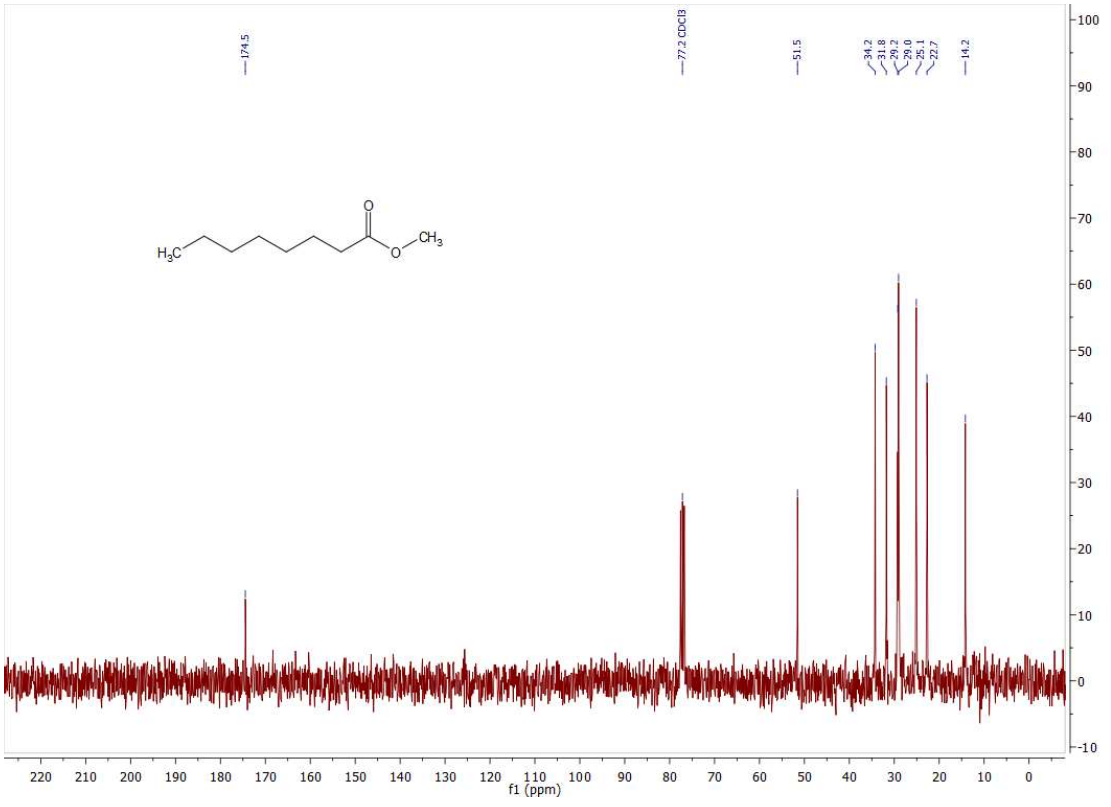

Methyl 4-nitrobenzoate (3a)

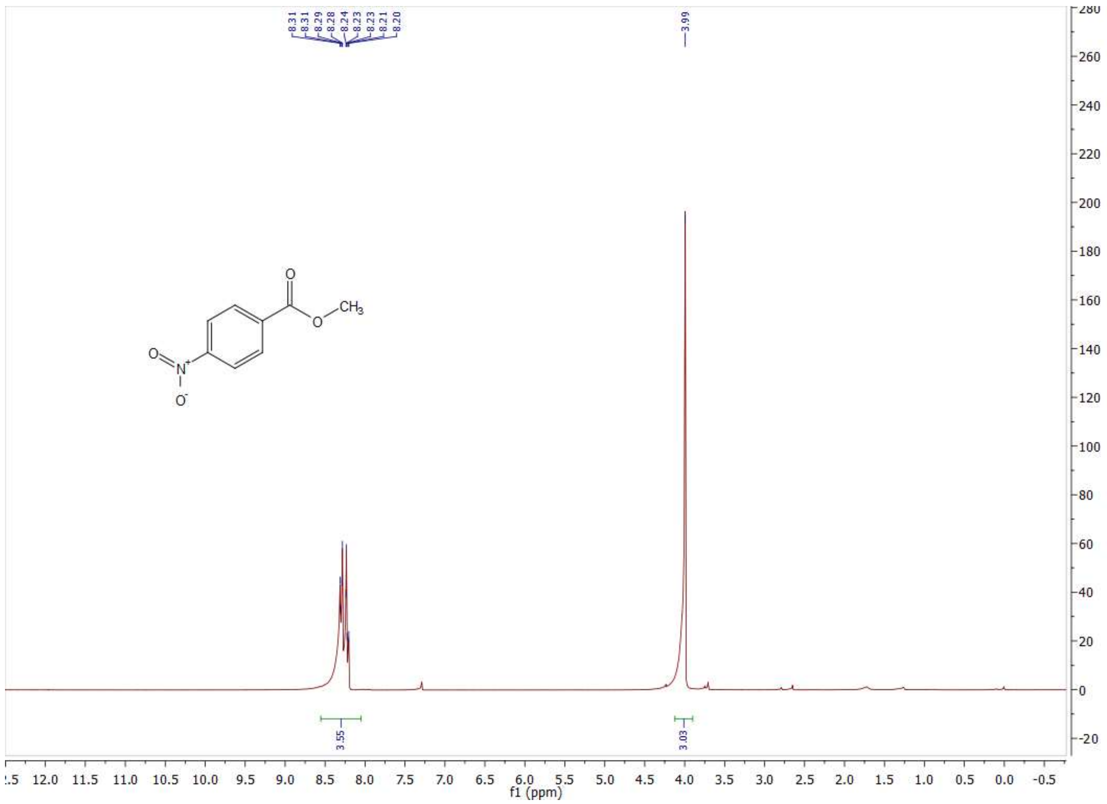

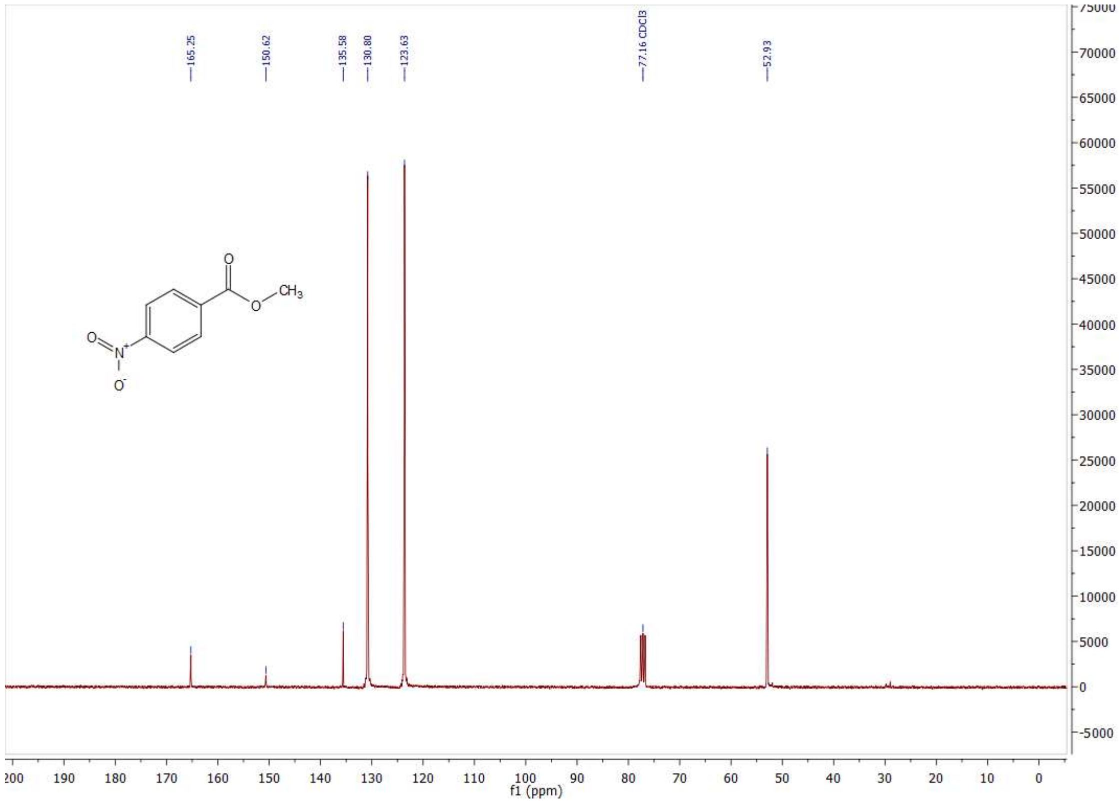

Methyl 4-fluorobenzoate (4a)

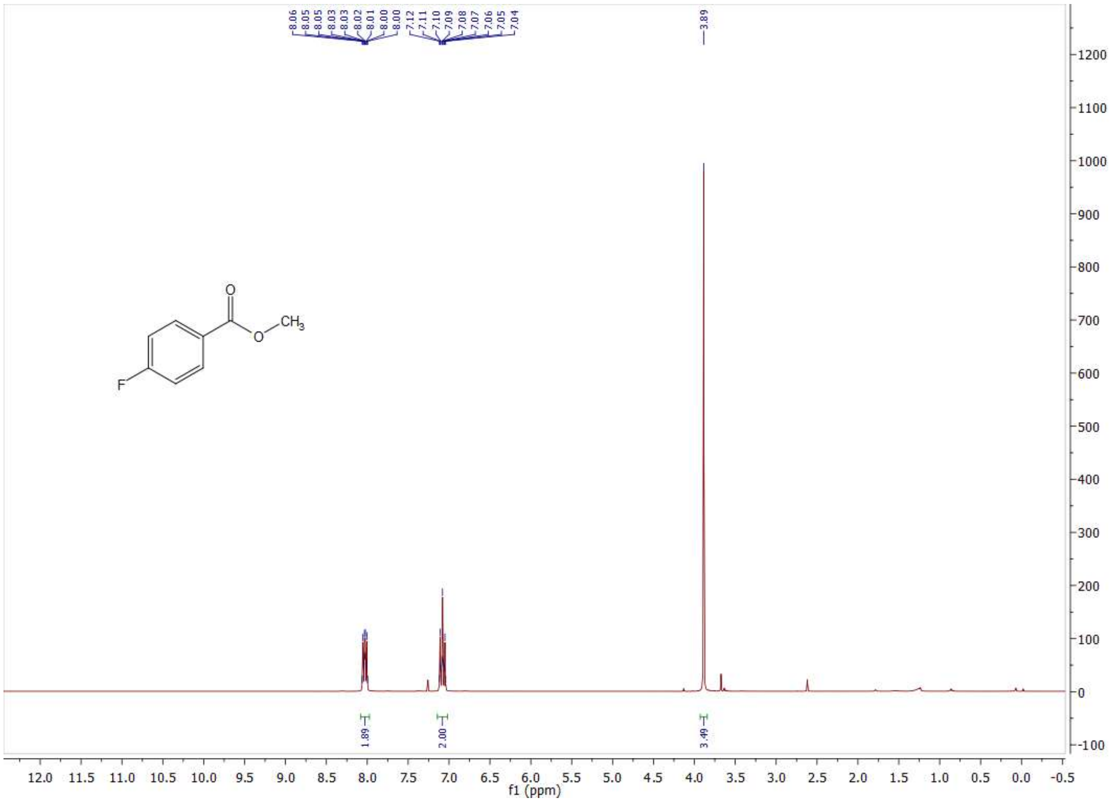

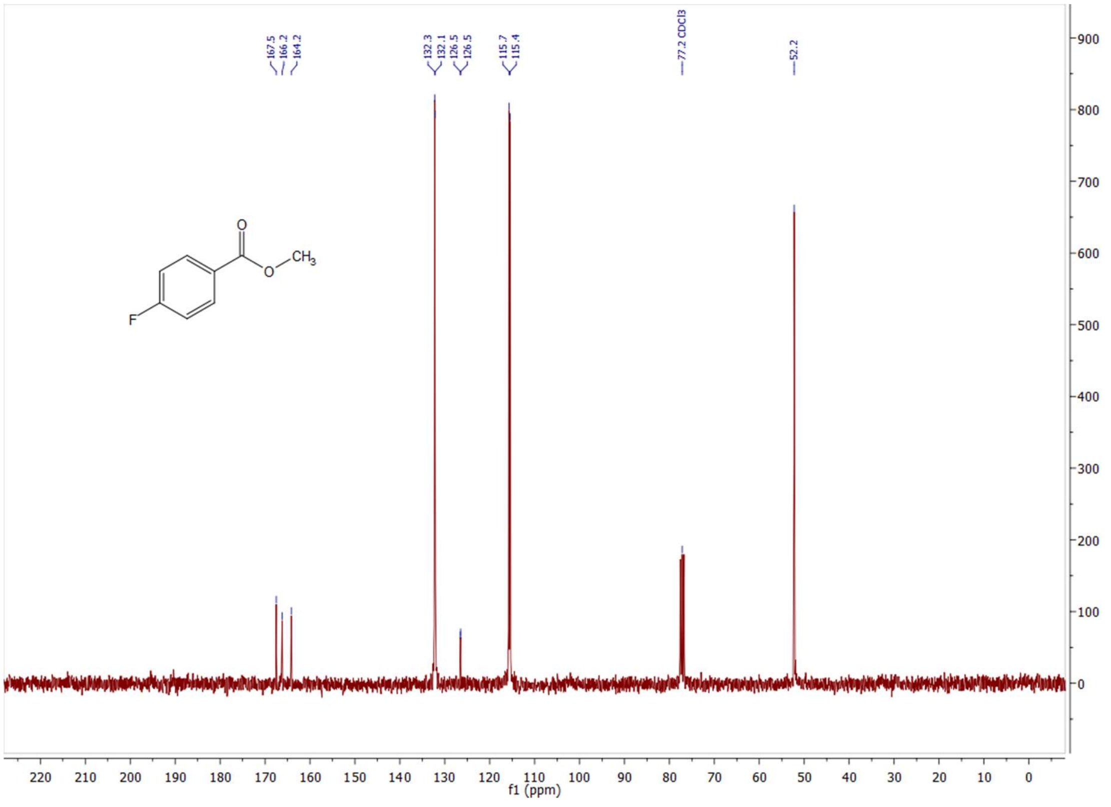

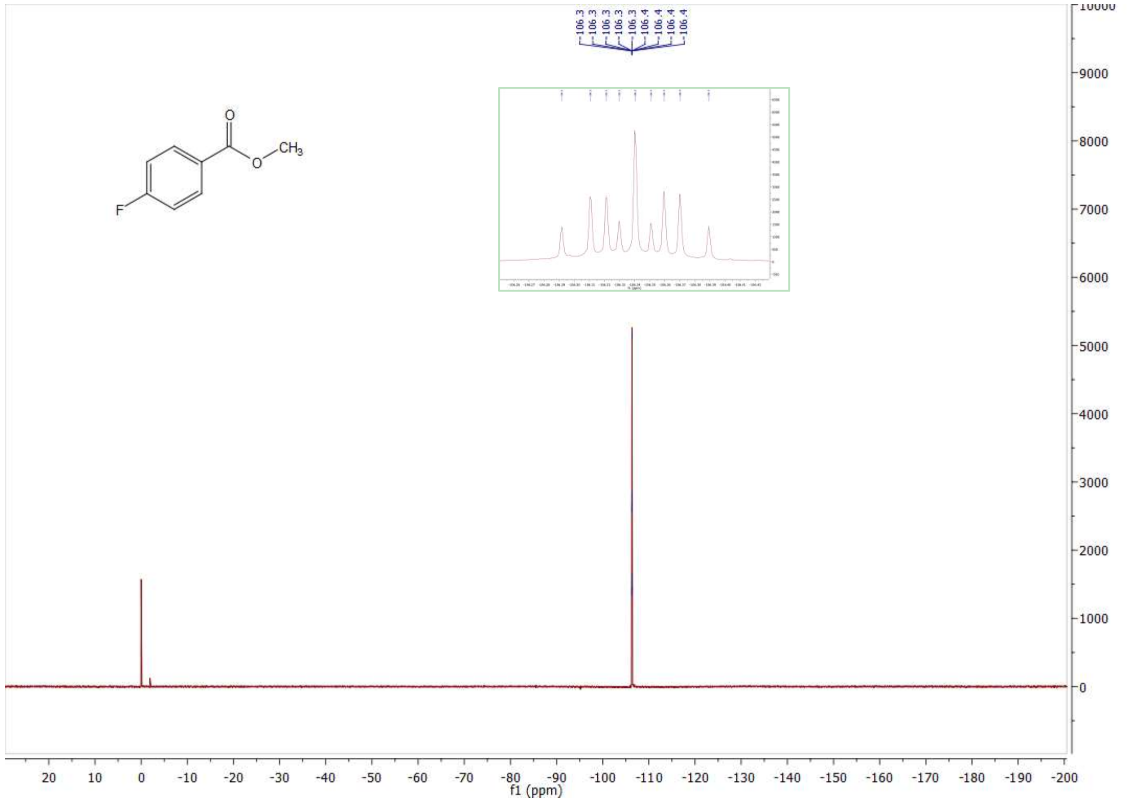

-106.3
-106.3
-106.3
-106.3
-106.3
-106.4
-106.4
-106.4
-106.4
O
O
CH3
F
10000
9000
8000
7000
6000
5000
4000
3000
2000
1000
0
20 10 0 -10 -20 -30 -40 -50 -60 -70 -80 -90 -100 -110 -120 -130 -140 -150 -160 -170 -180 -190 -200
f1 (ppm)

Methyl 4-methylbenzoate (5a)

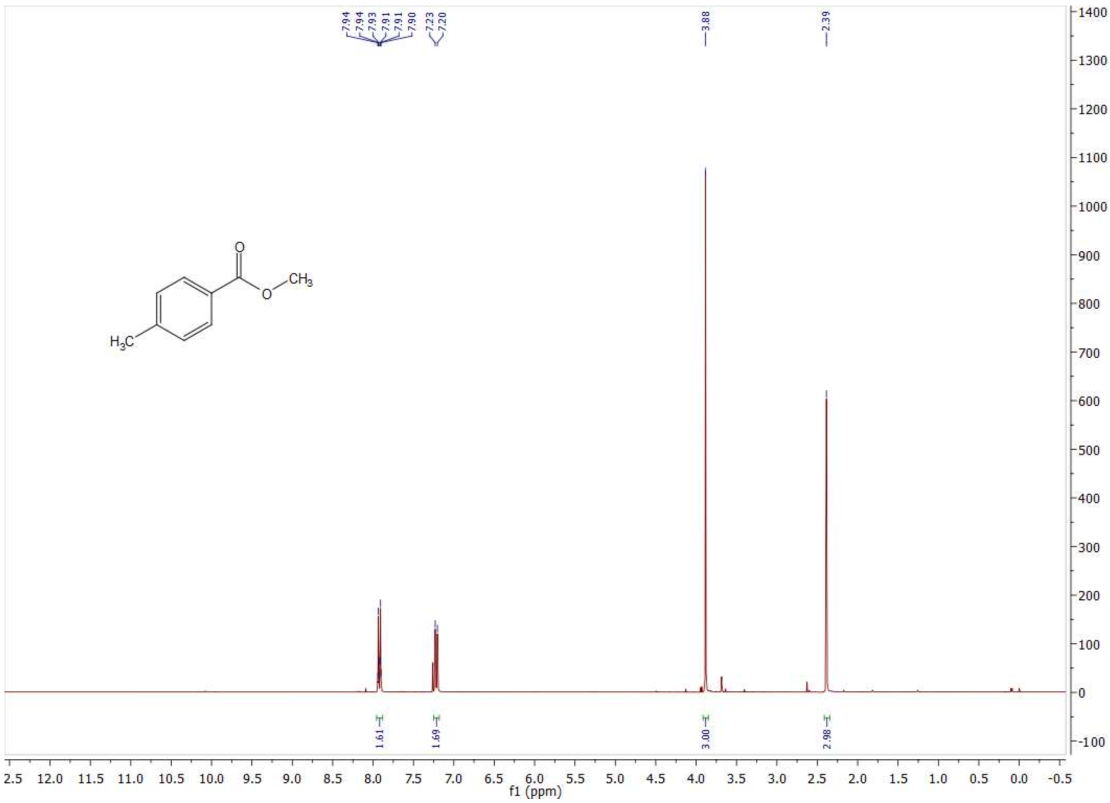

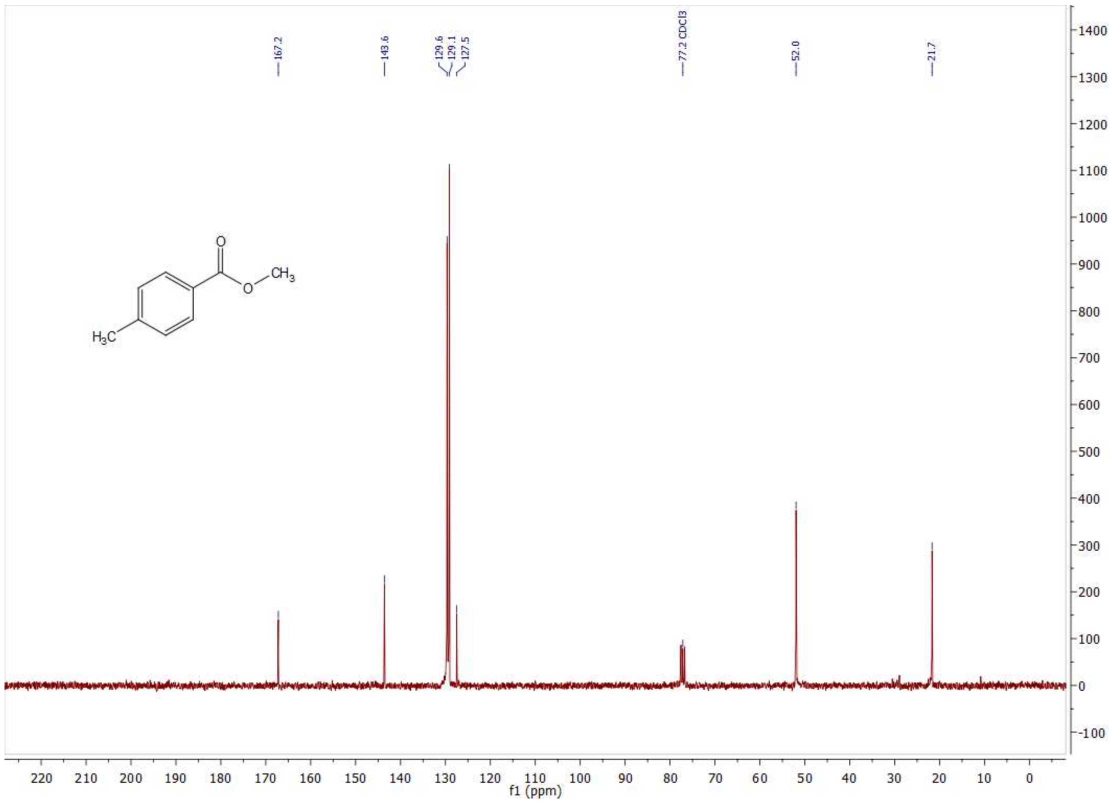

Methyl 4-methoxybenzoate (6a)

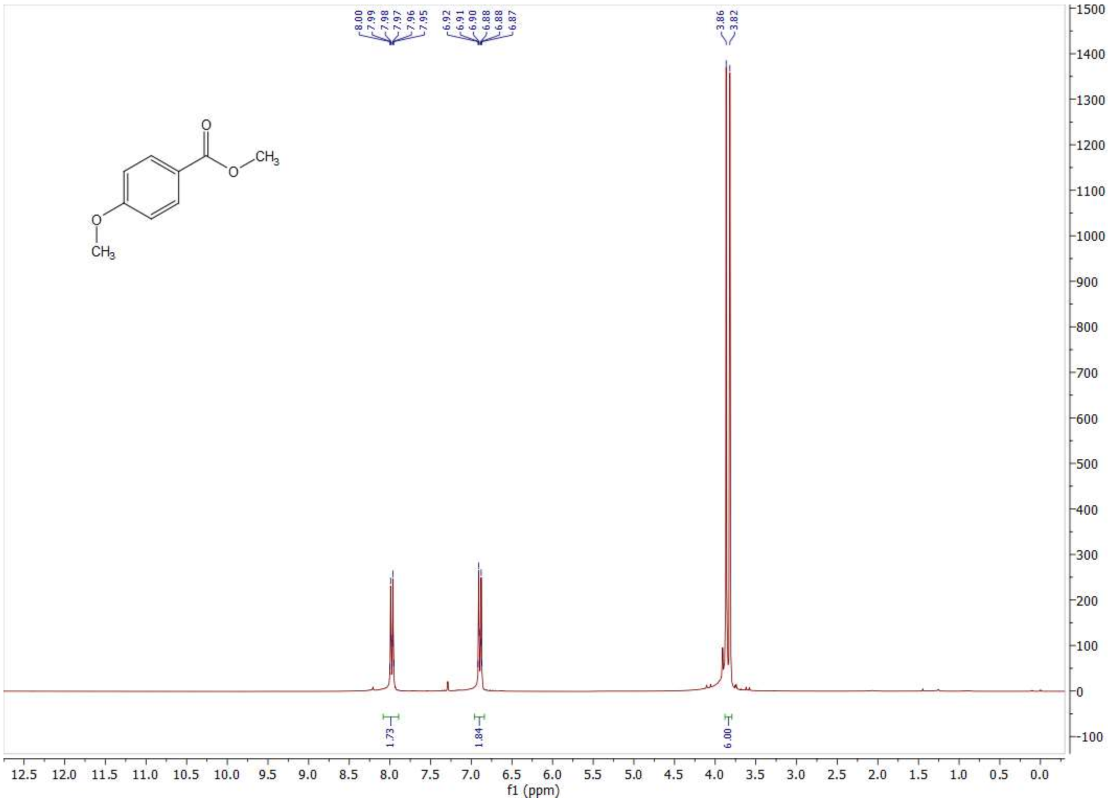

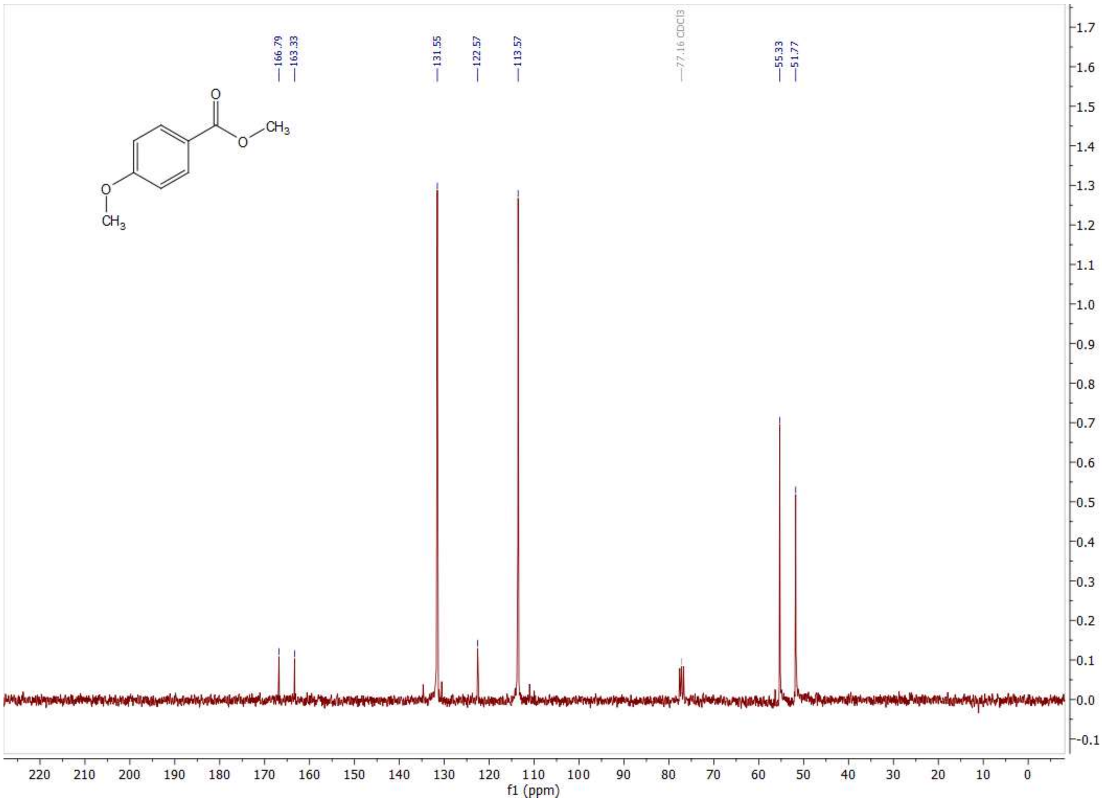

Methyl 3-nitrobenzoate (7a)

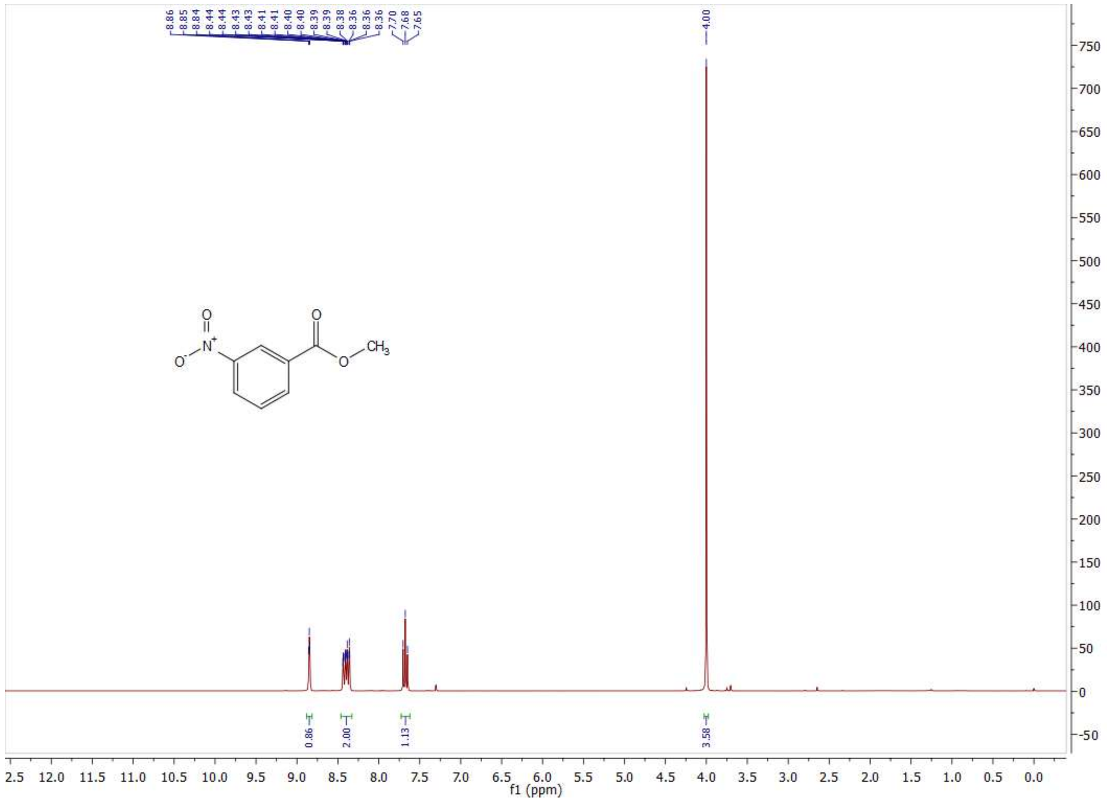

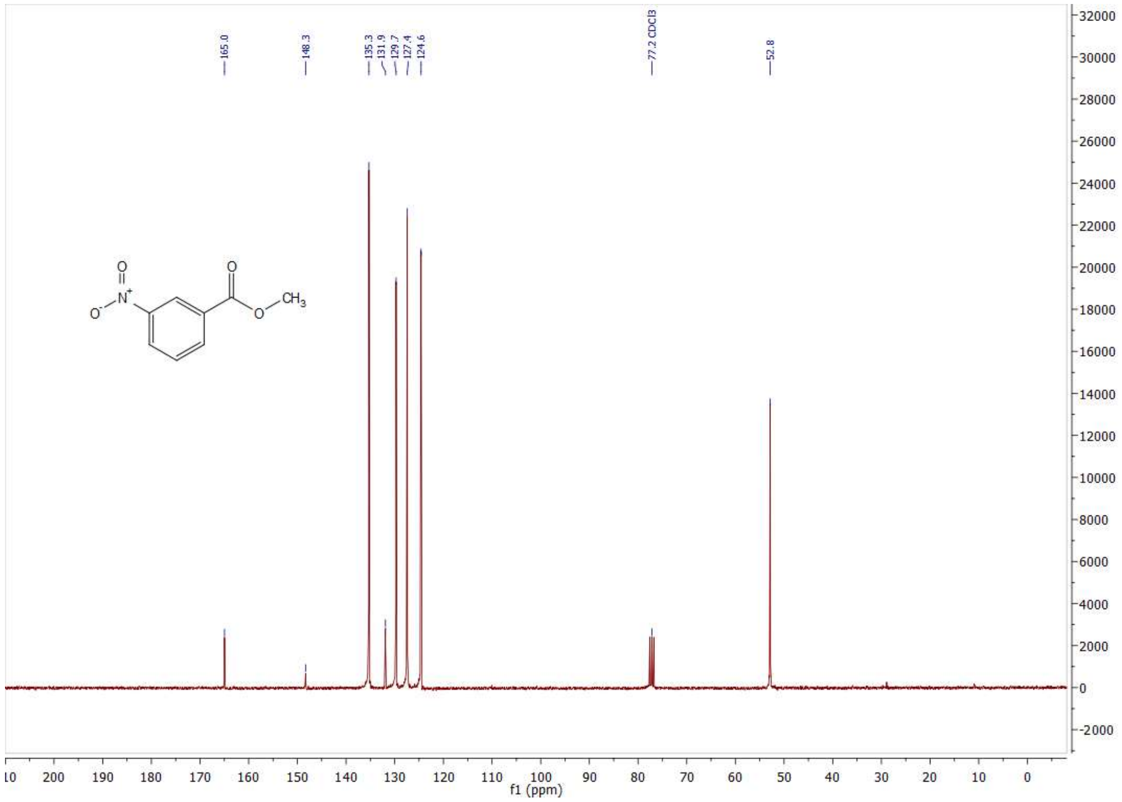

Methyl 3-fluorobenzoate (8a)

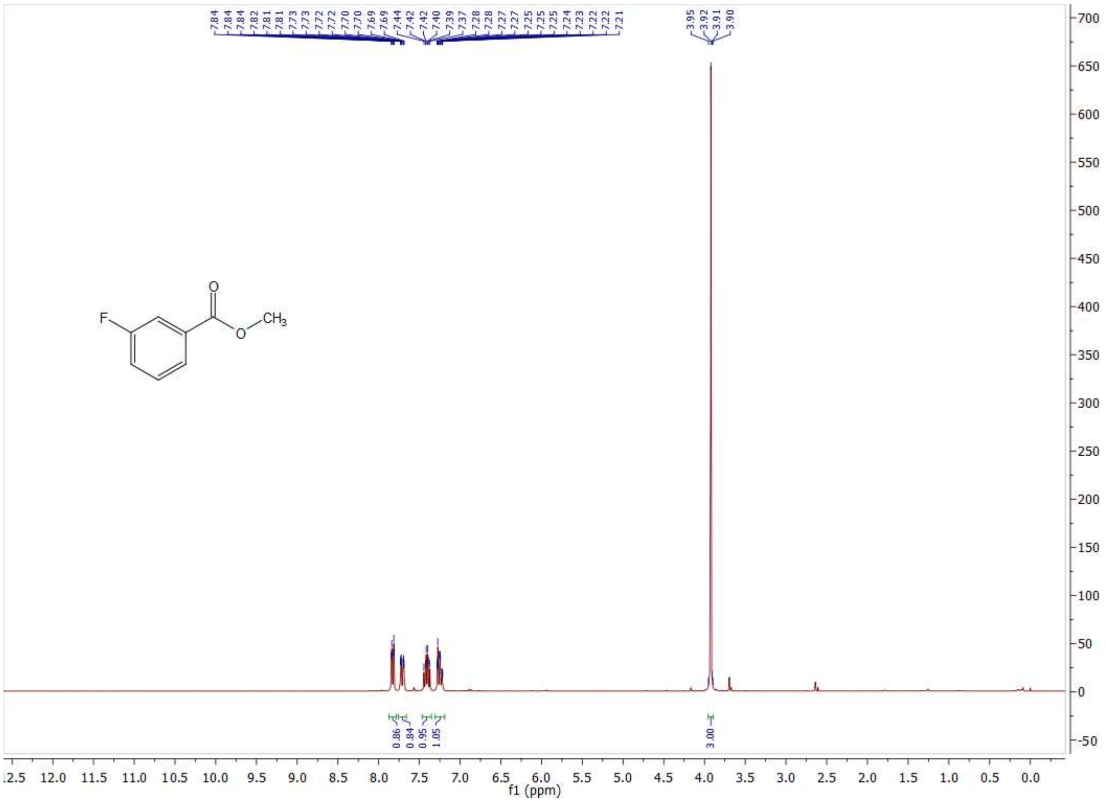

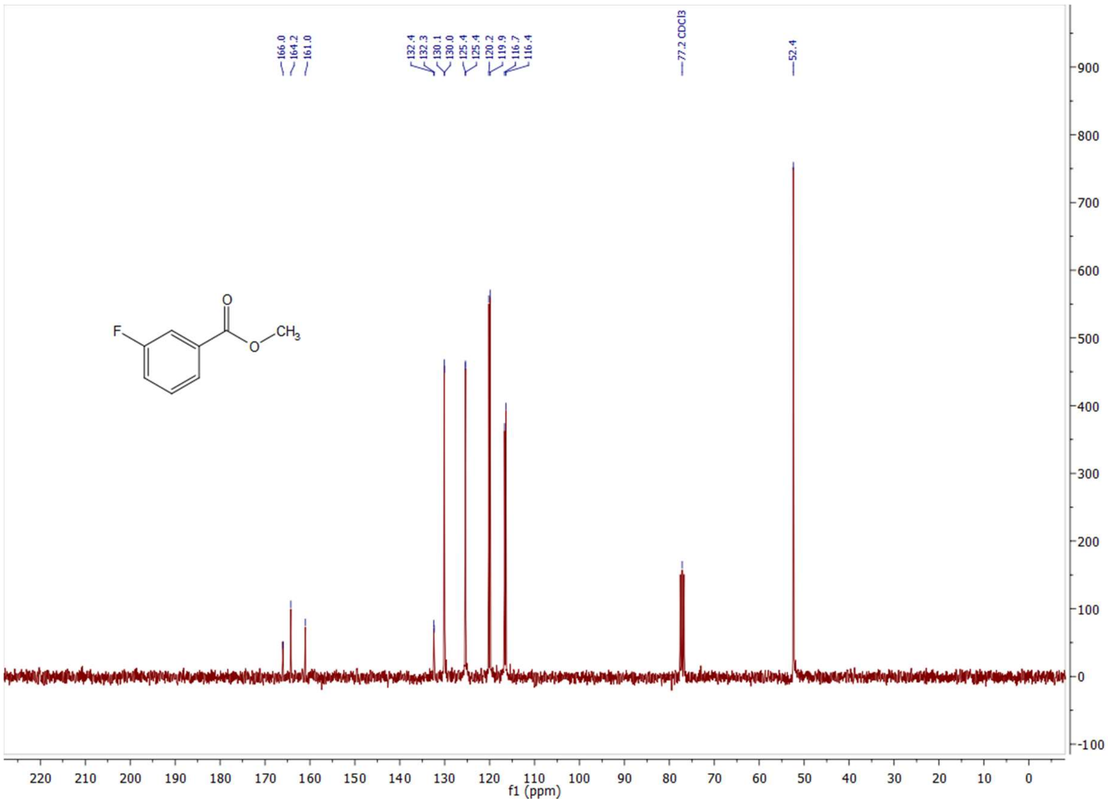

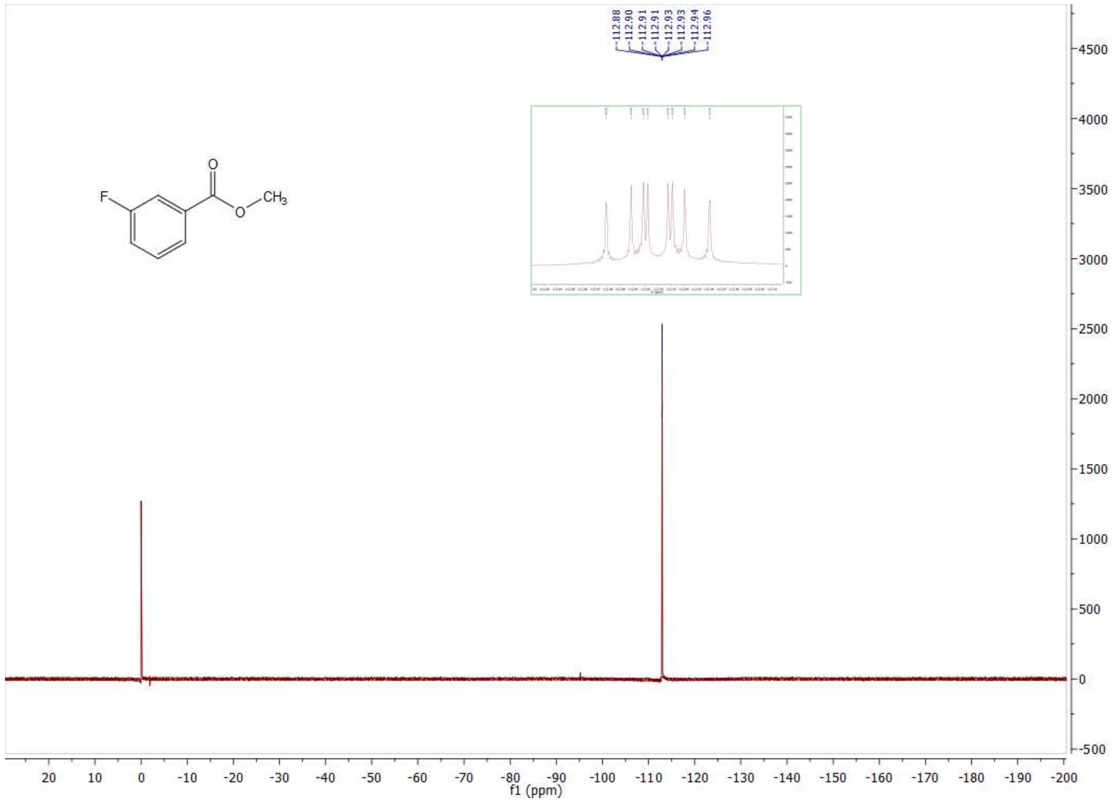
F
O
O
CH3
-112.88
-112.90
-112.91
-112.91
-112.93
-112.93
-112.94
-112.96
f1 (ppm)

Methyl 3-methylbenzoate (9a)

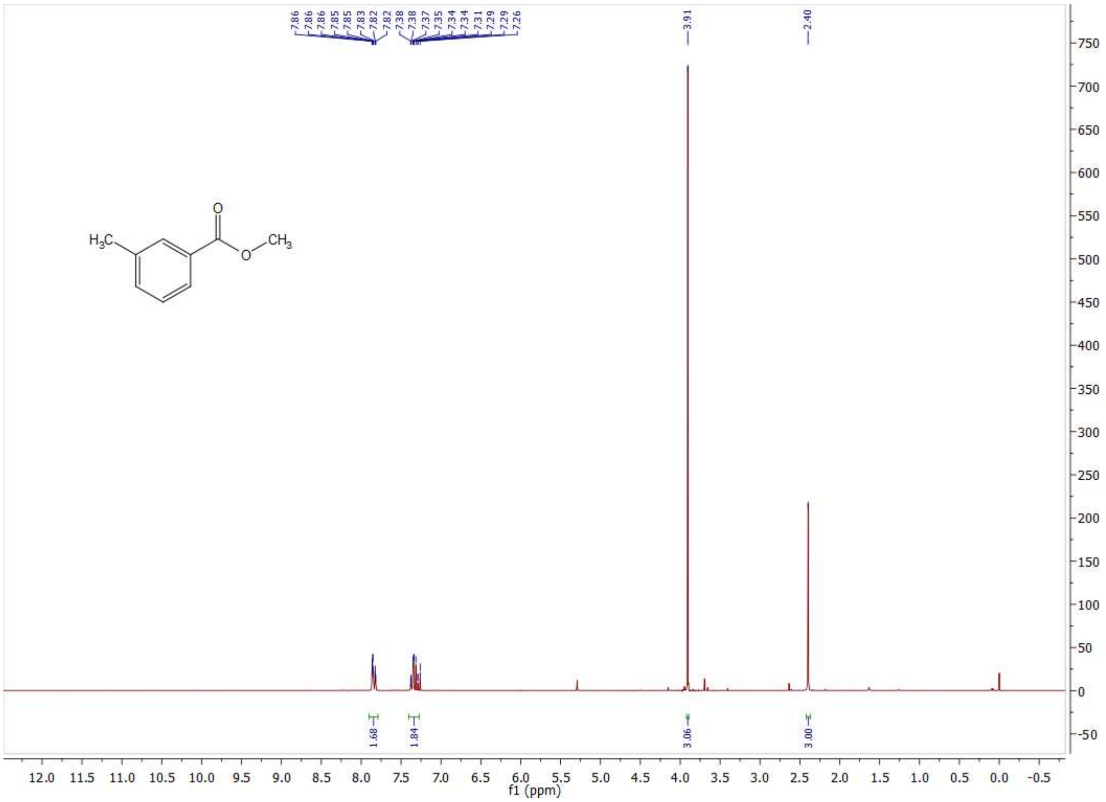

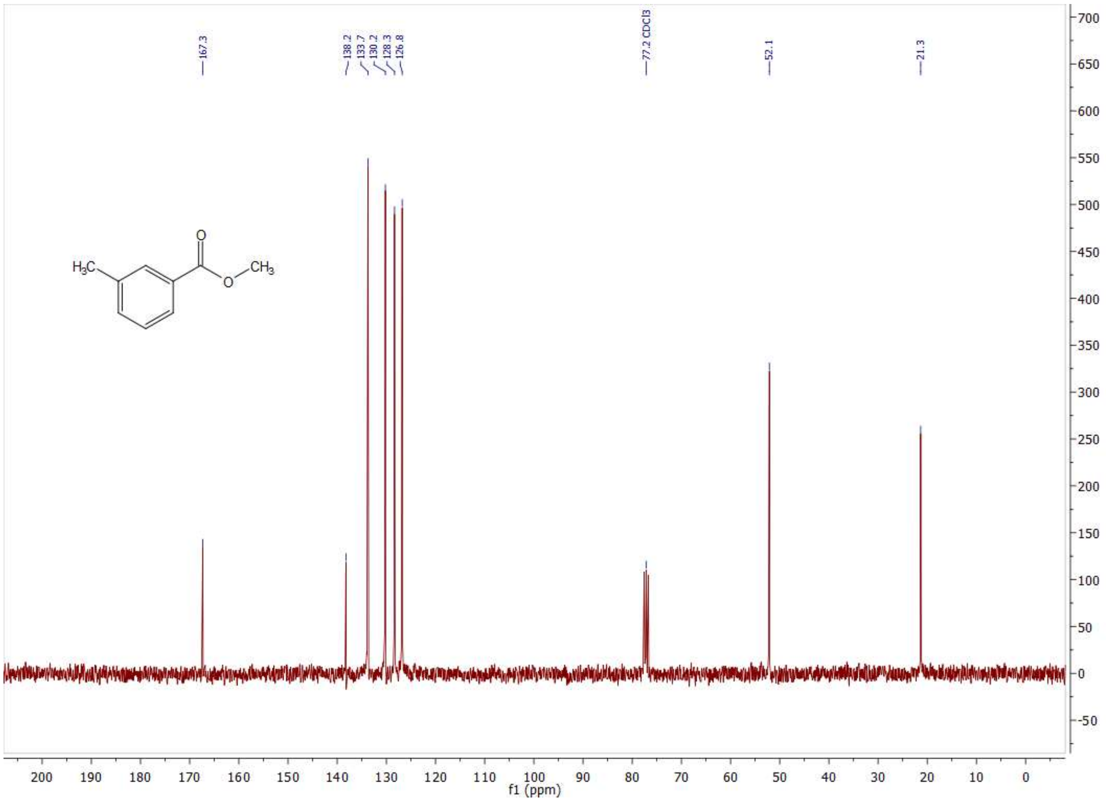

Methyl 3-methoxybenzoate (10a)

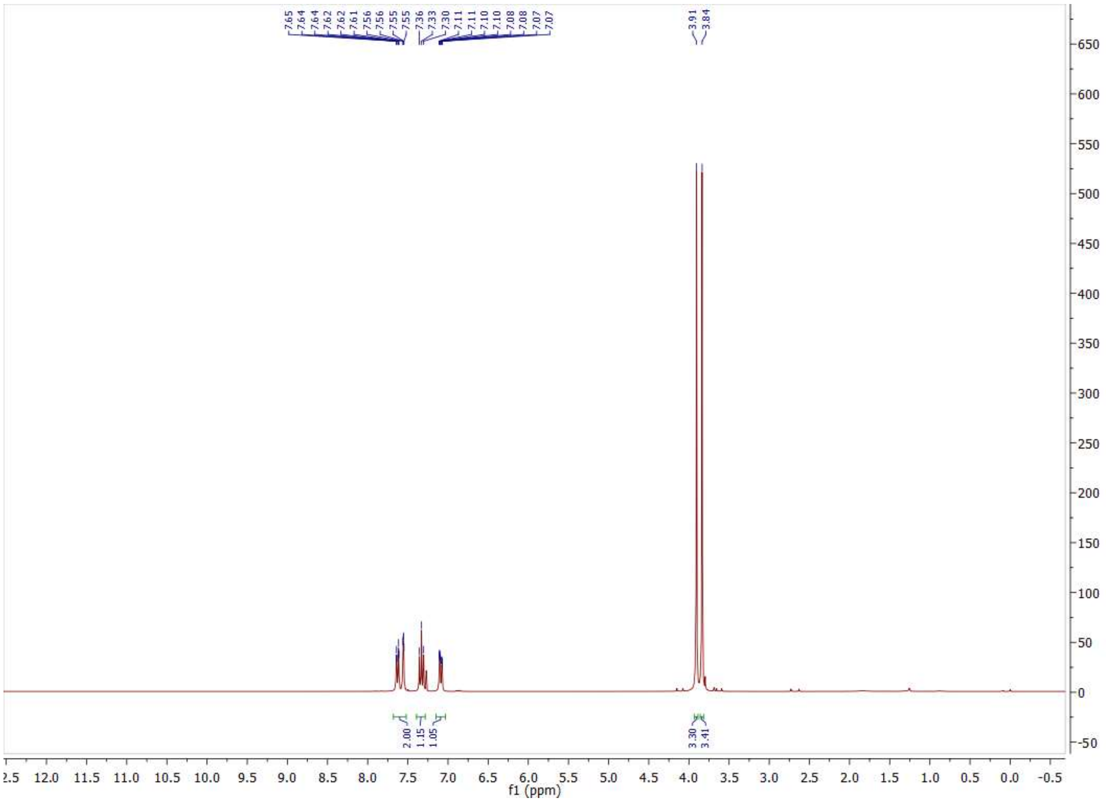

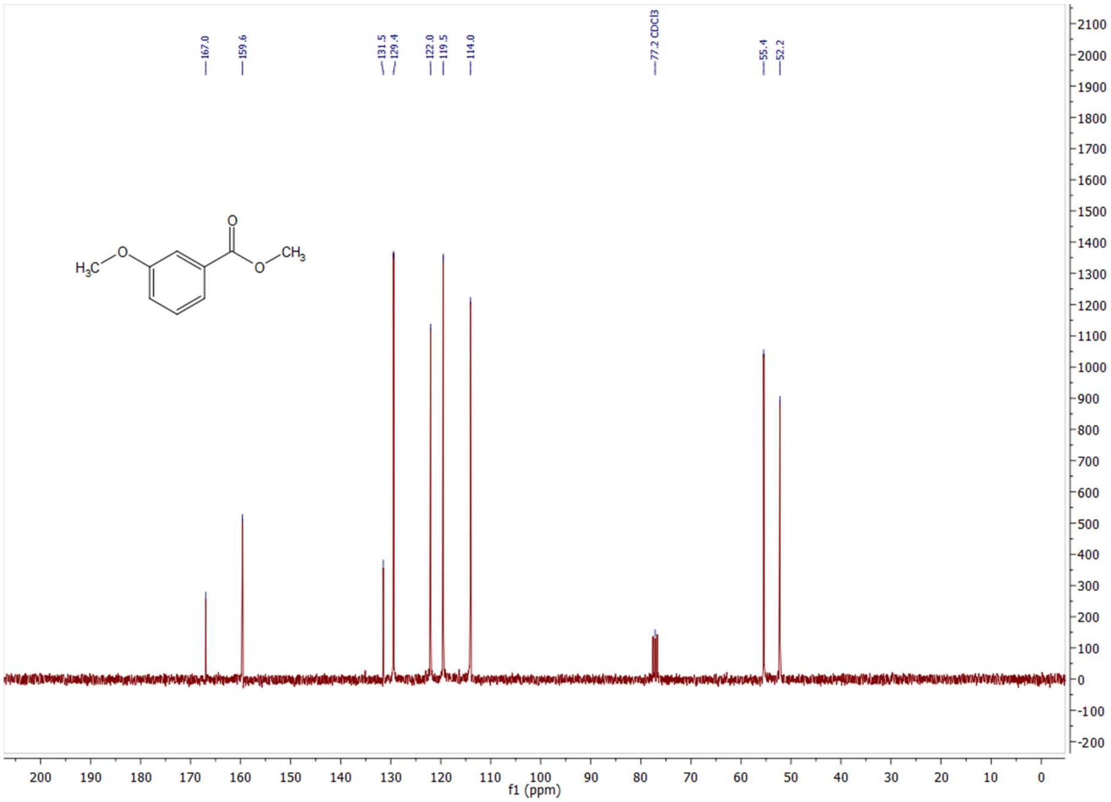

Methyl 2-nitrobenzoate (11a)

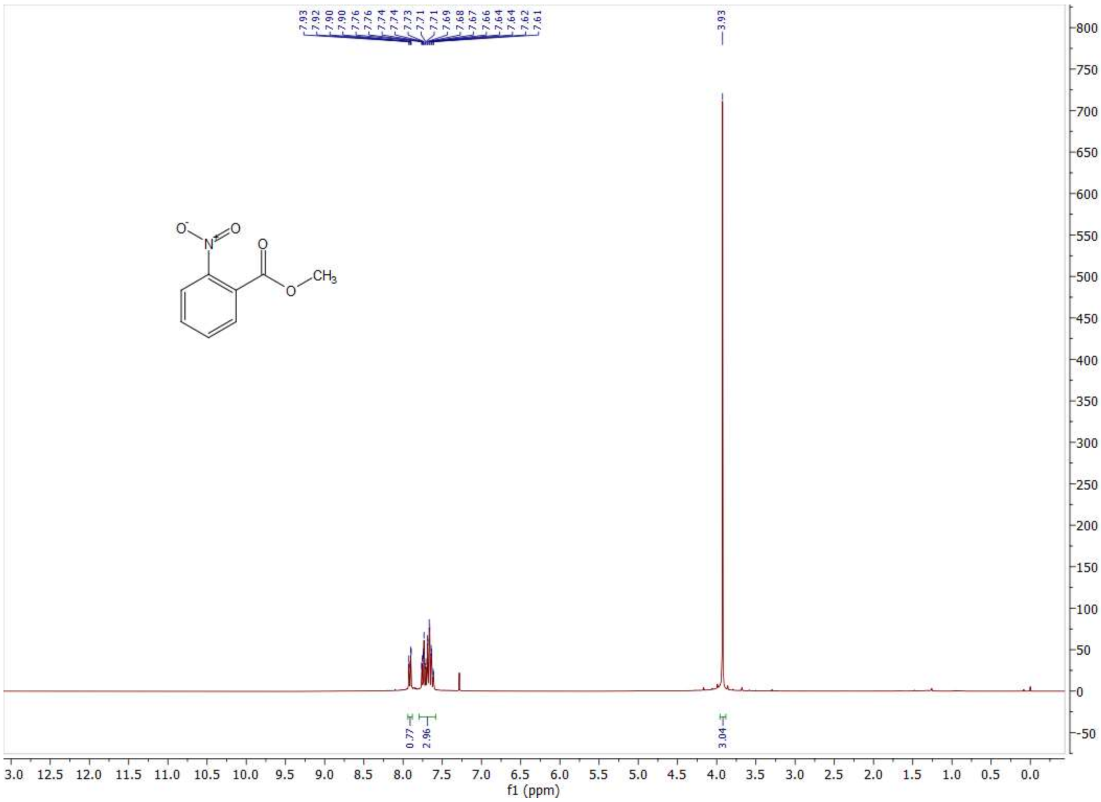

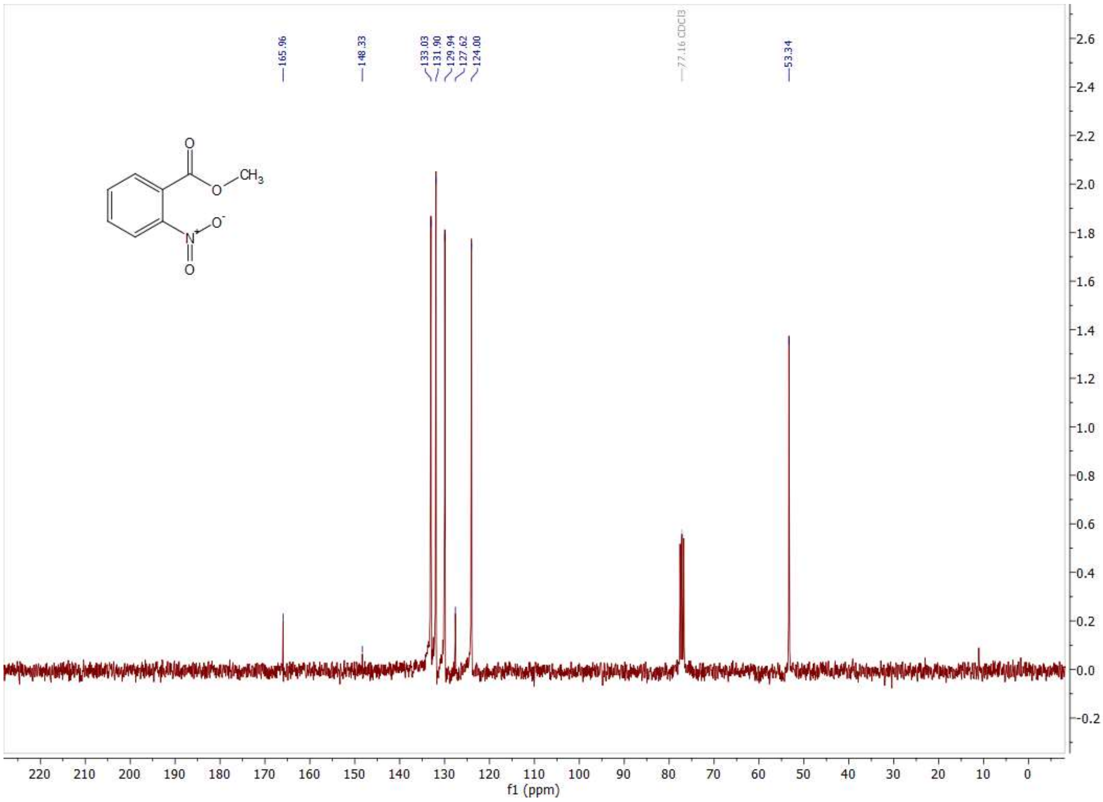

Methyl 2-fluorobenzoate (12a)

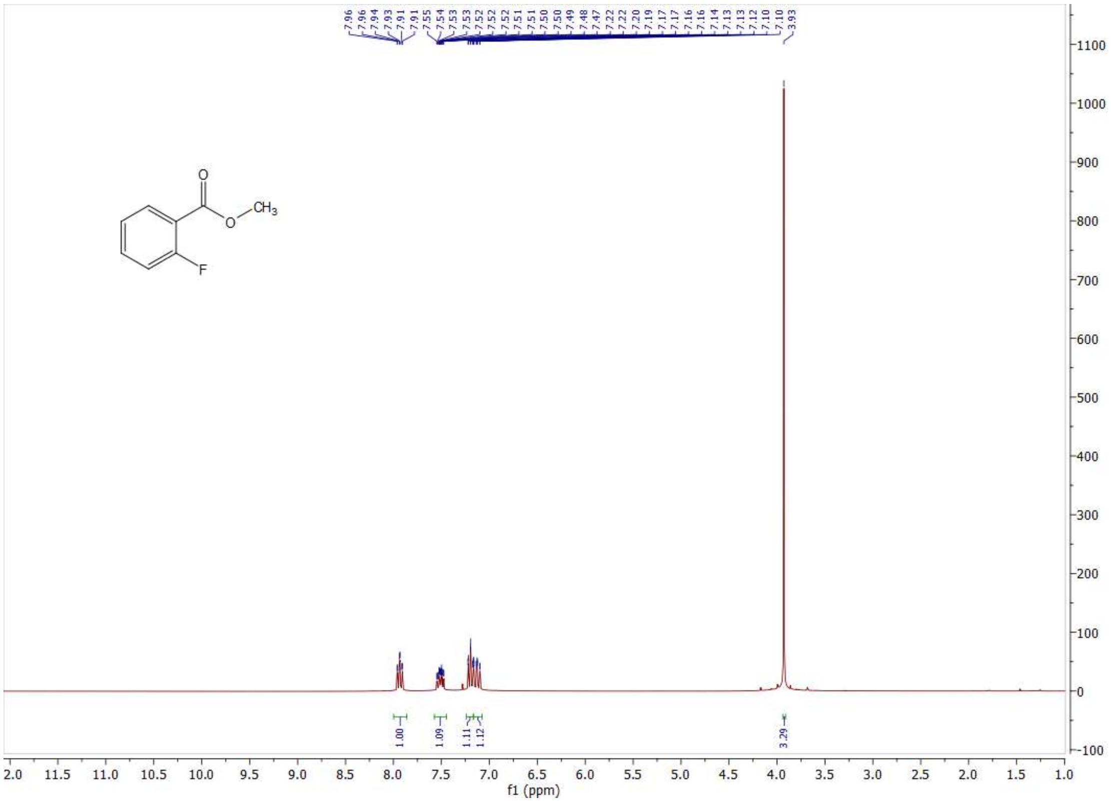

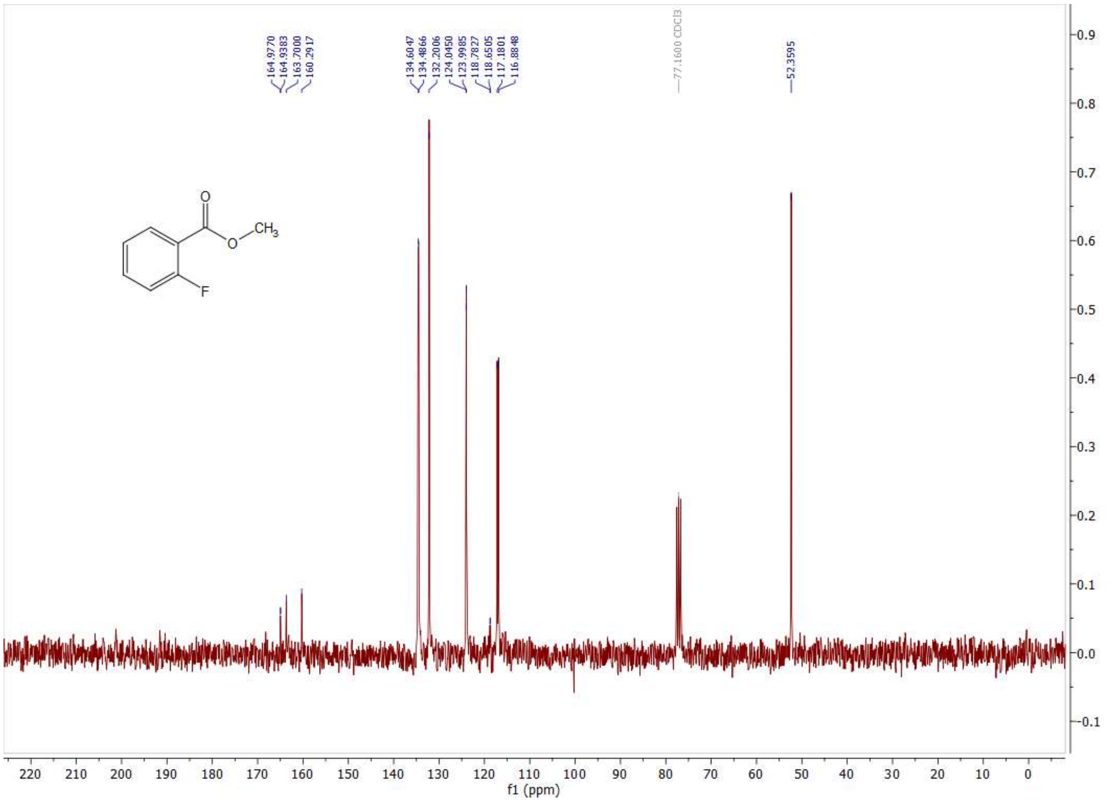

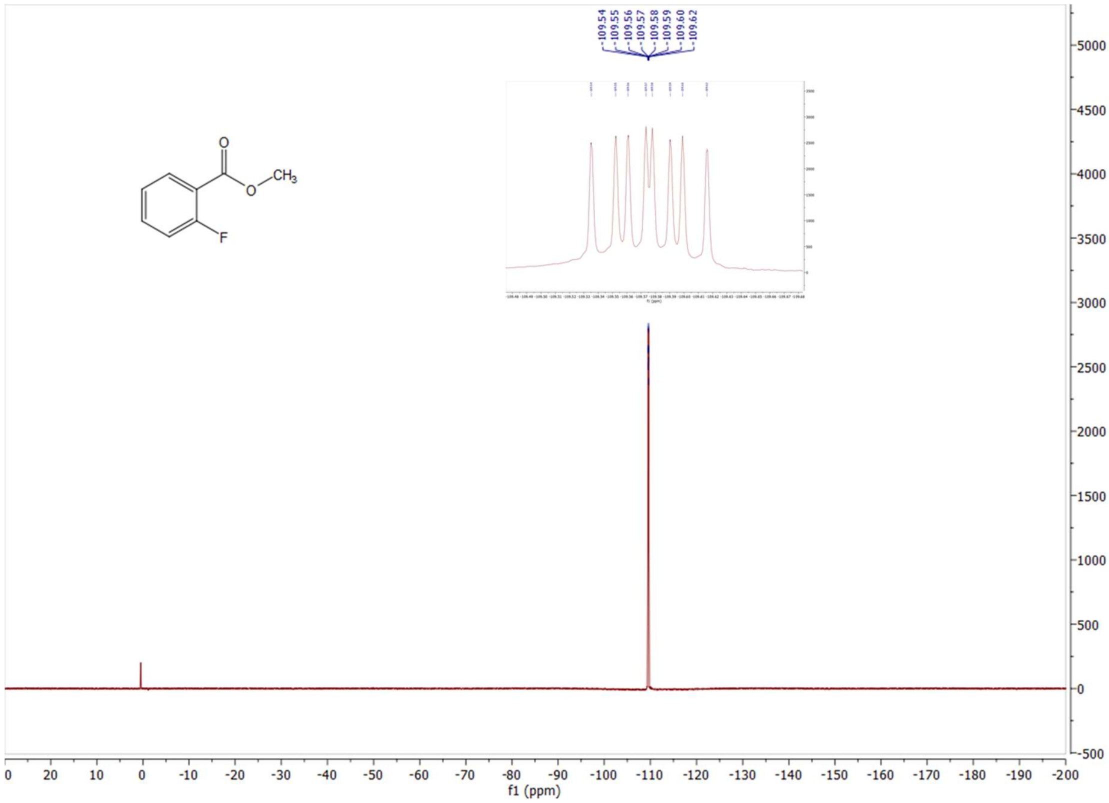

-109.54
-109.55
-109.56
-109.57
-109.58
-109.59
-109.60
-109.62
O
O
CH3
F
f1 (ppm)

Methyl 2-methylbenzoate (13a)

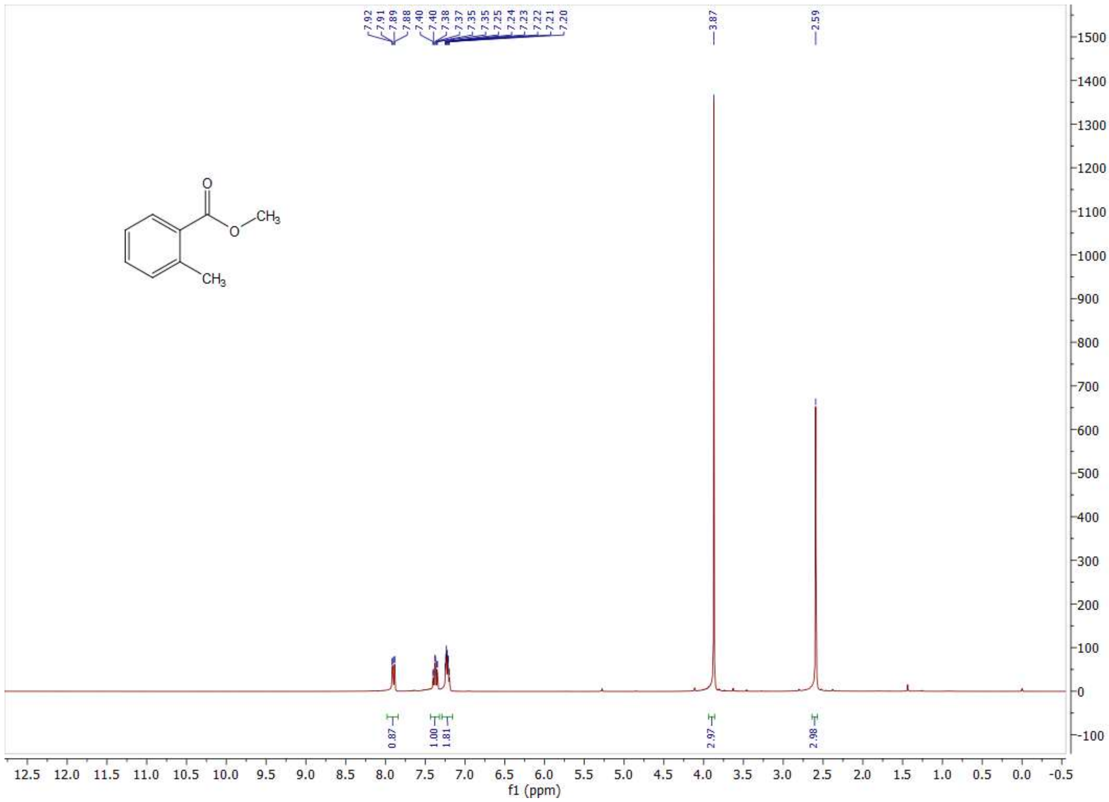

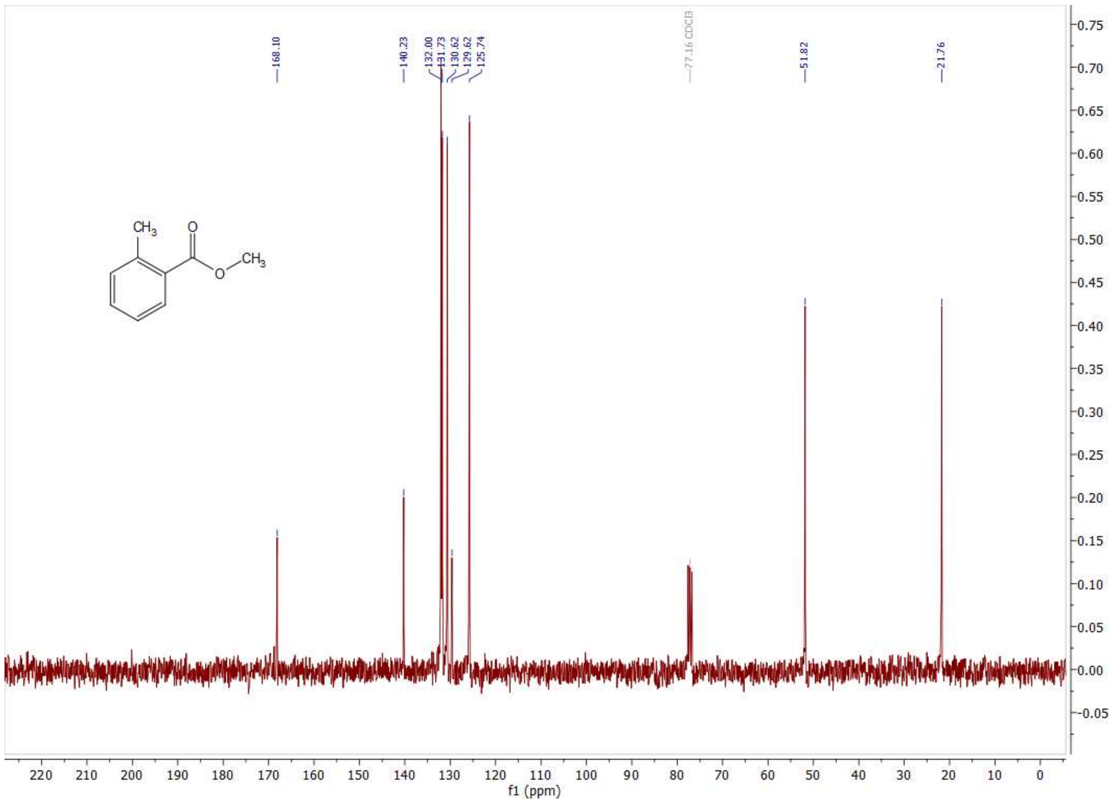

Methyl 2-iodobenzoate (15a)

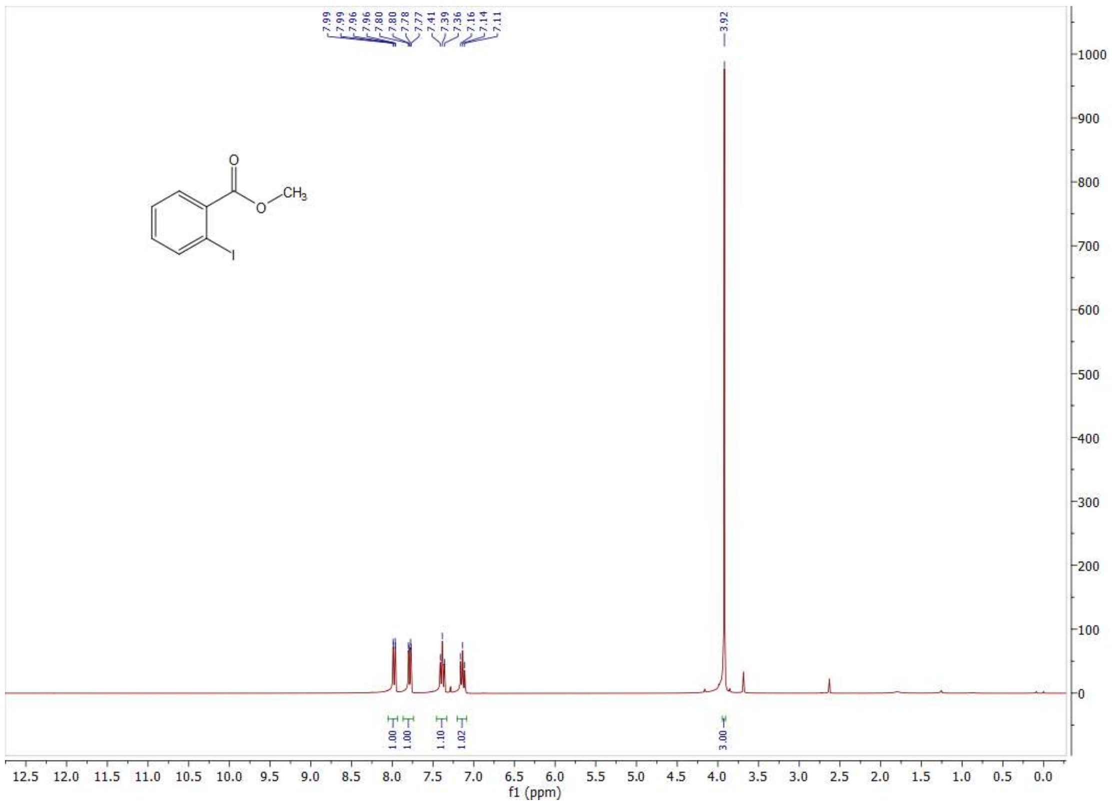

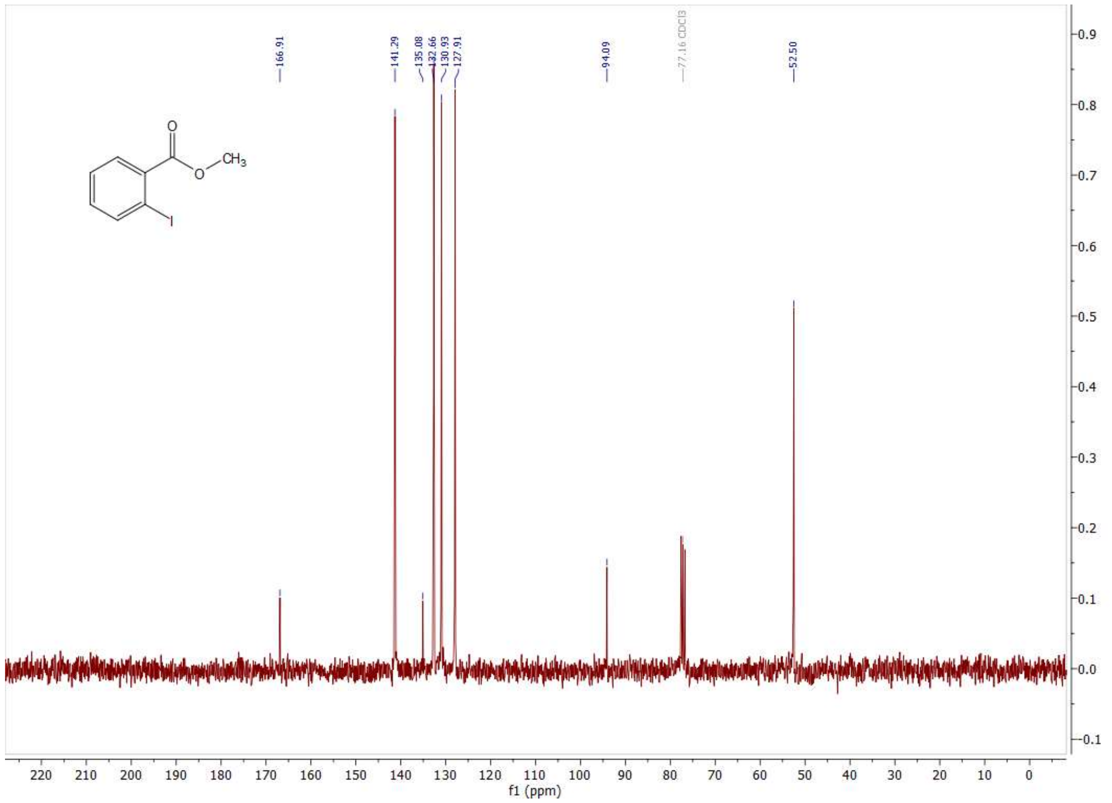

Methyl 3,4-dimethoxybenzoate (18a)

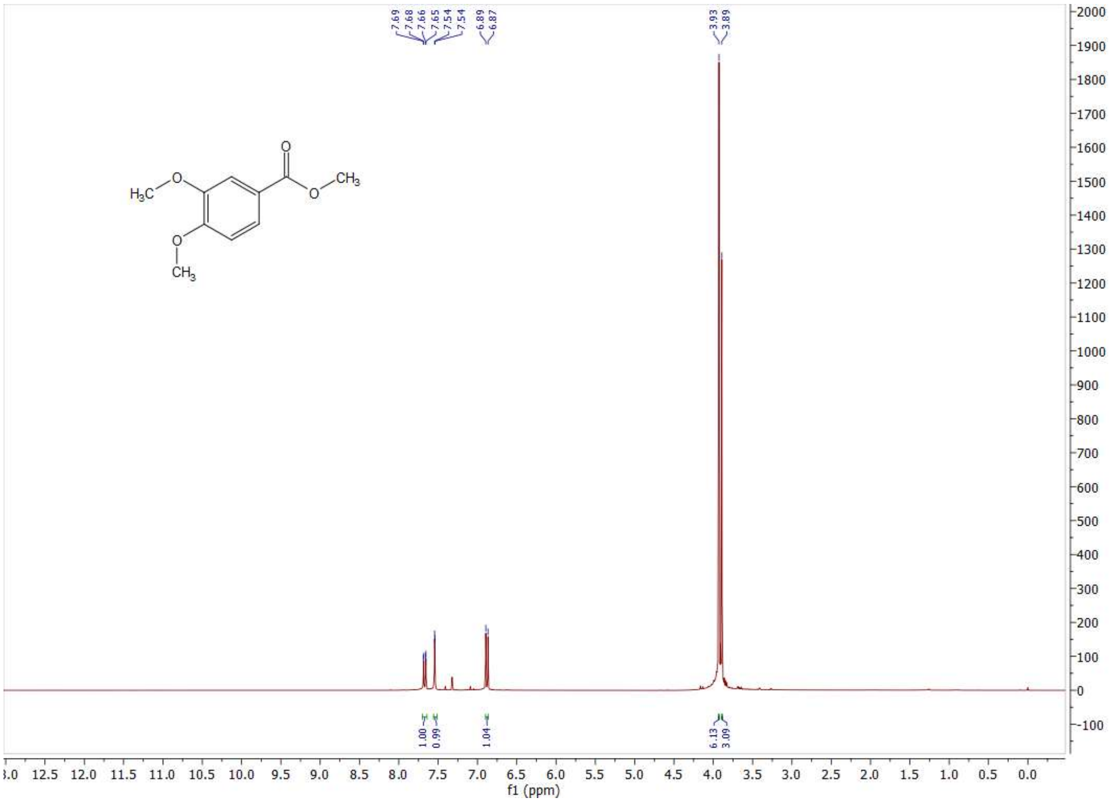

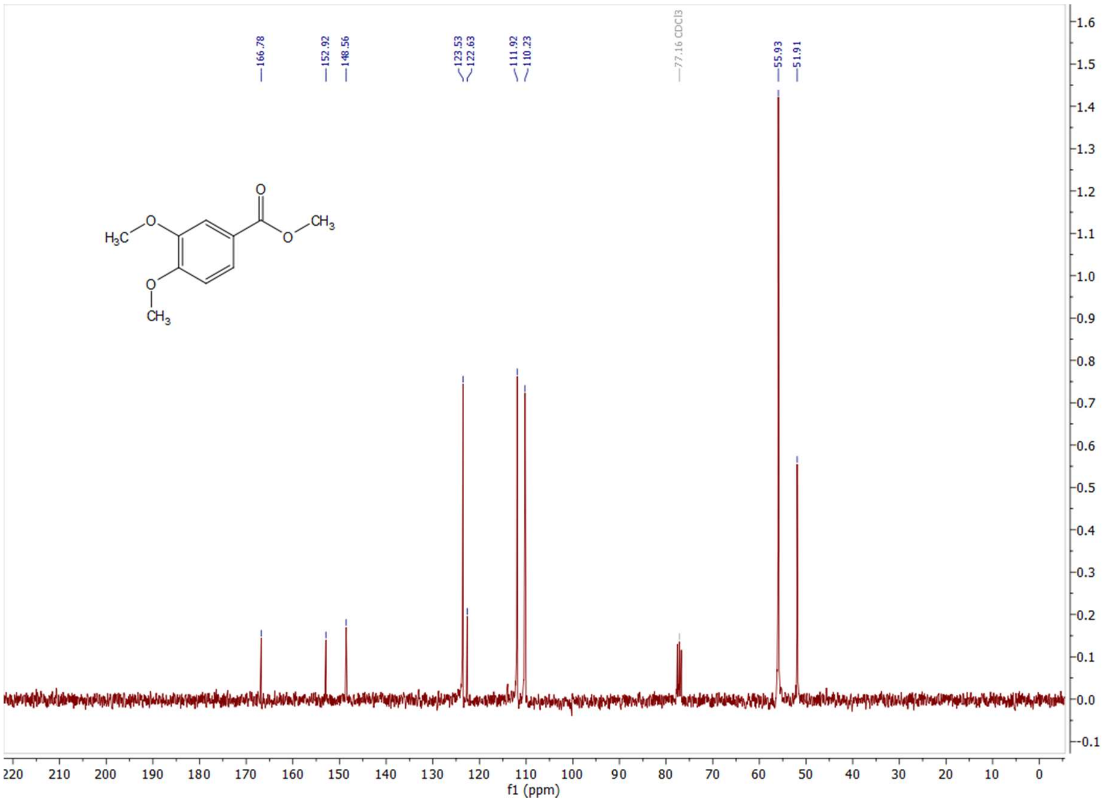

Methyl myristate (20a)

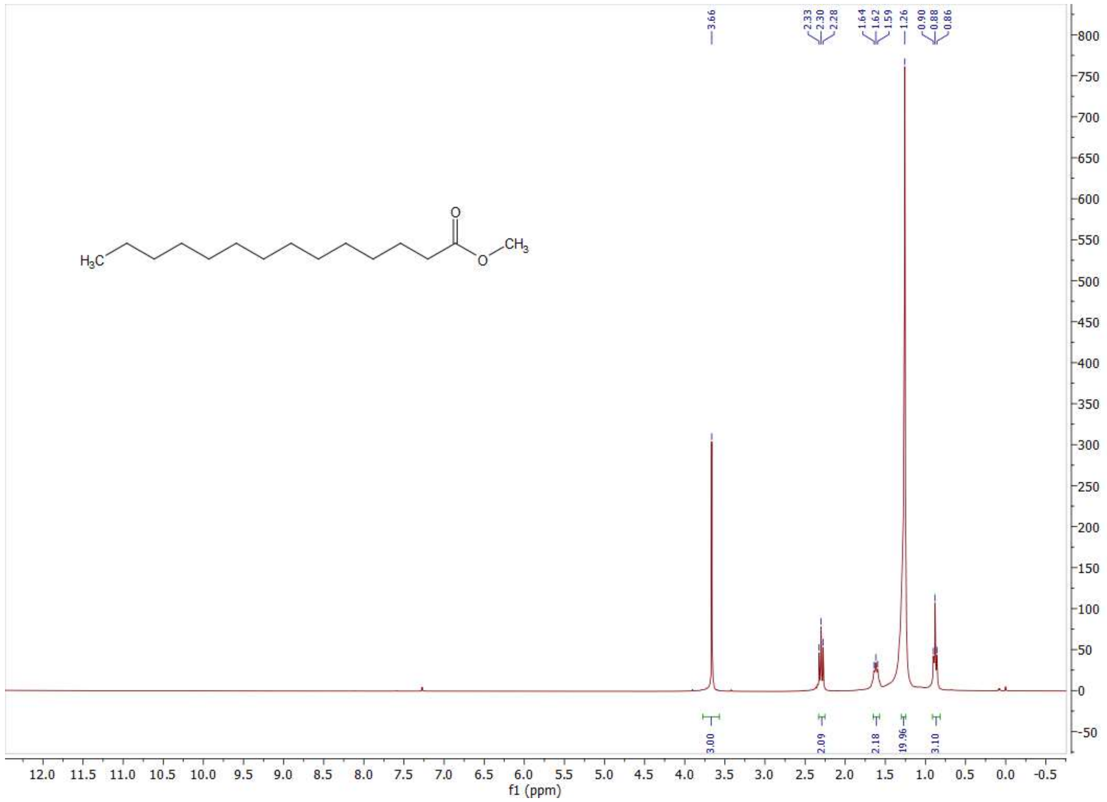

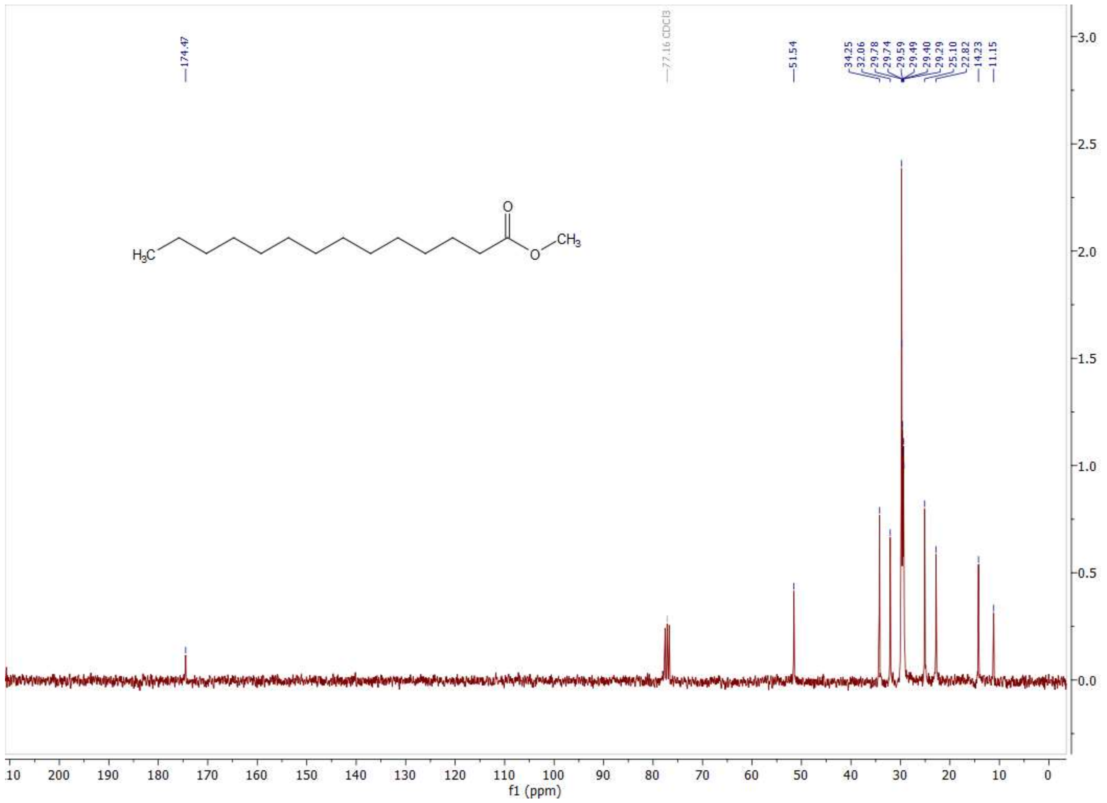

Methyl stearate (21a)

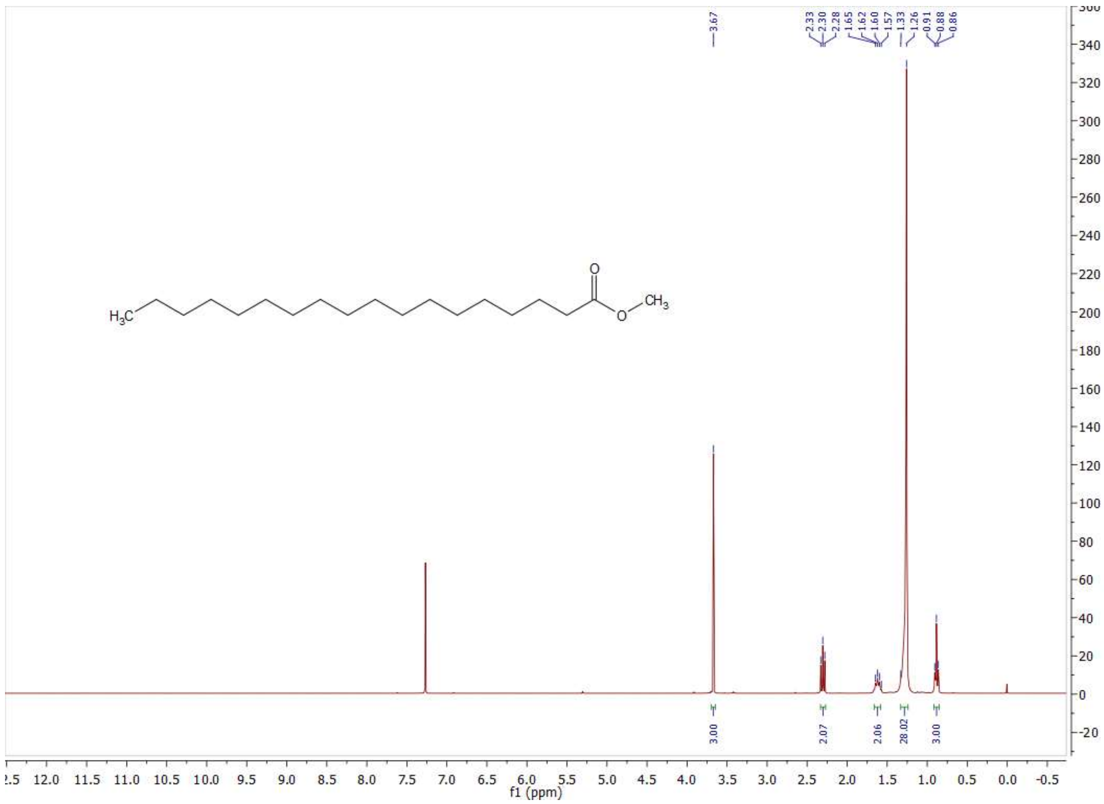

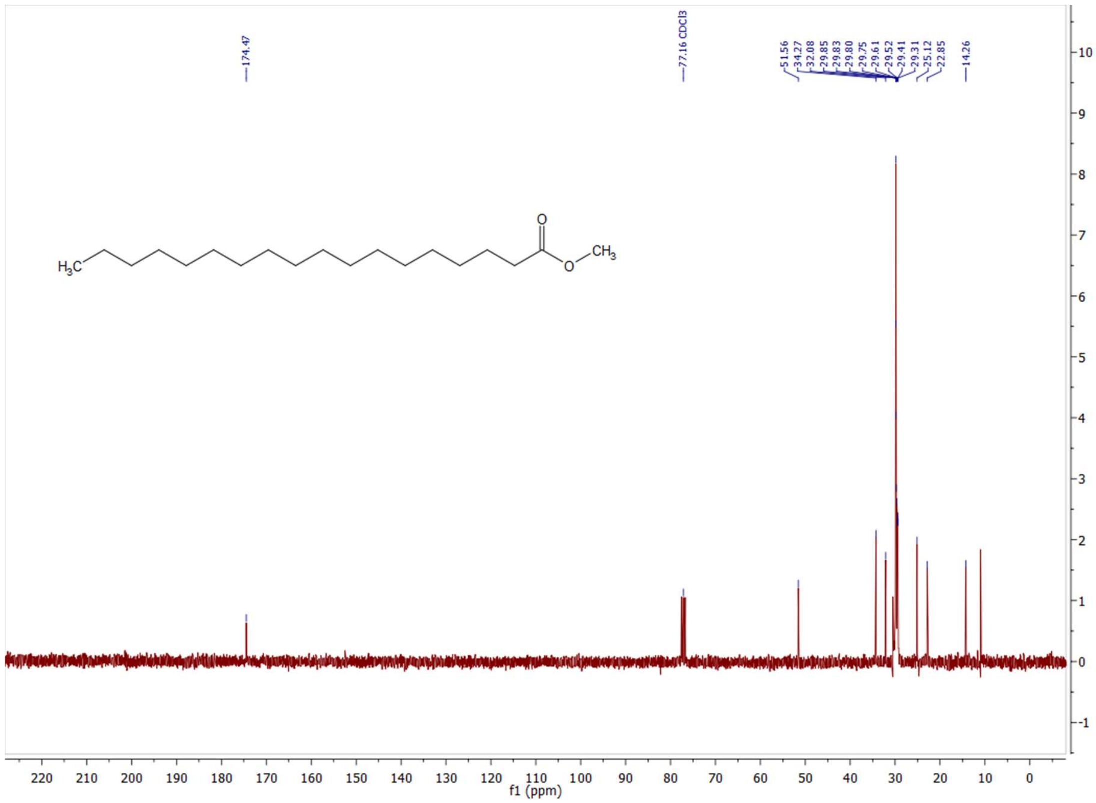

Methyl oleate (22a)

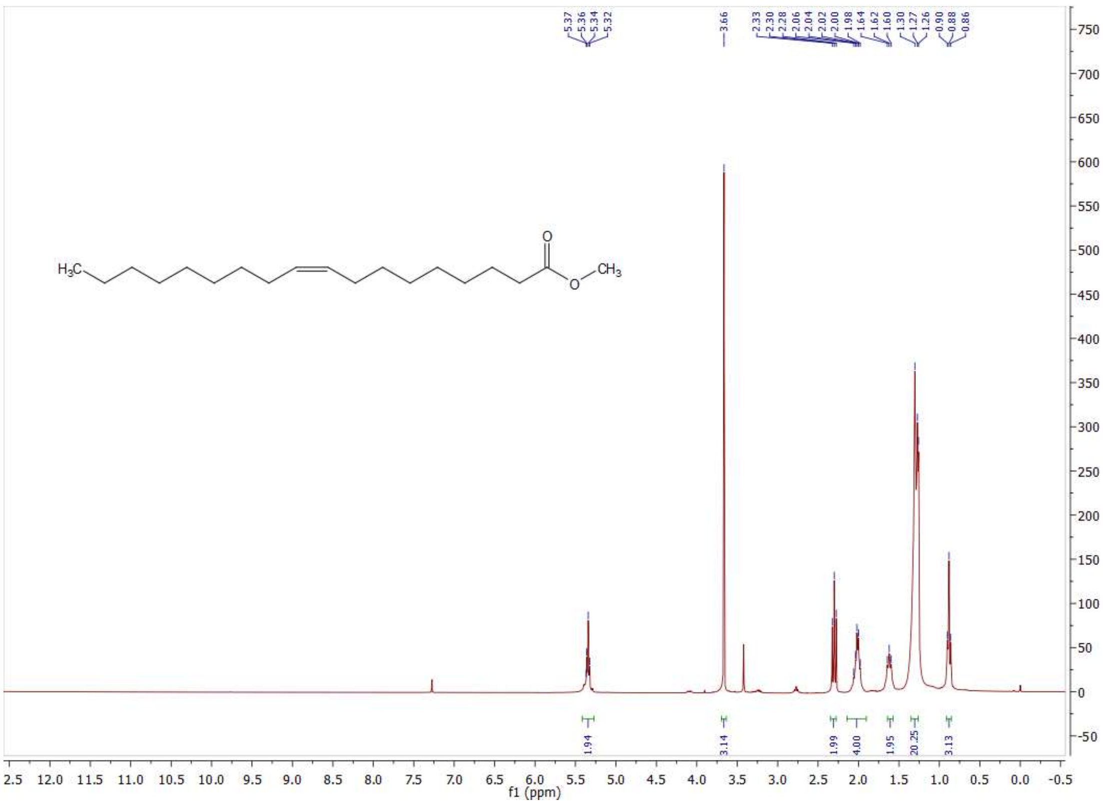

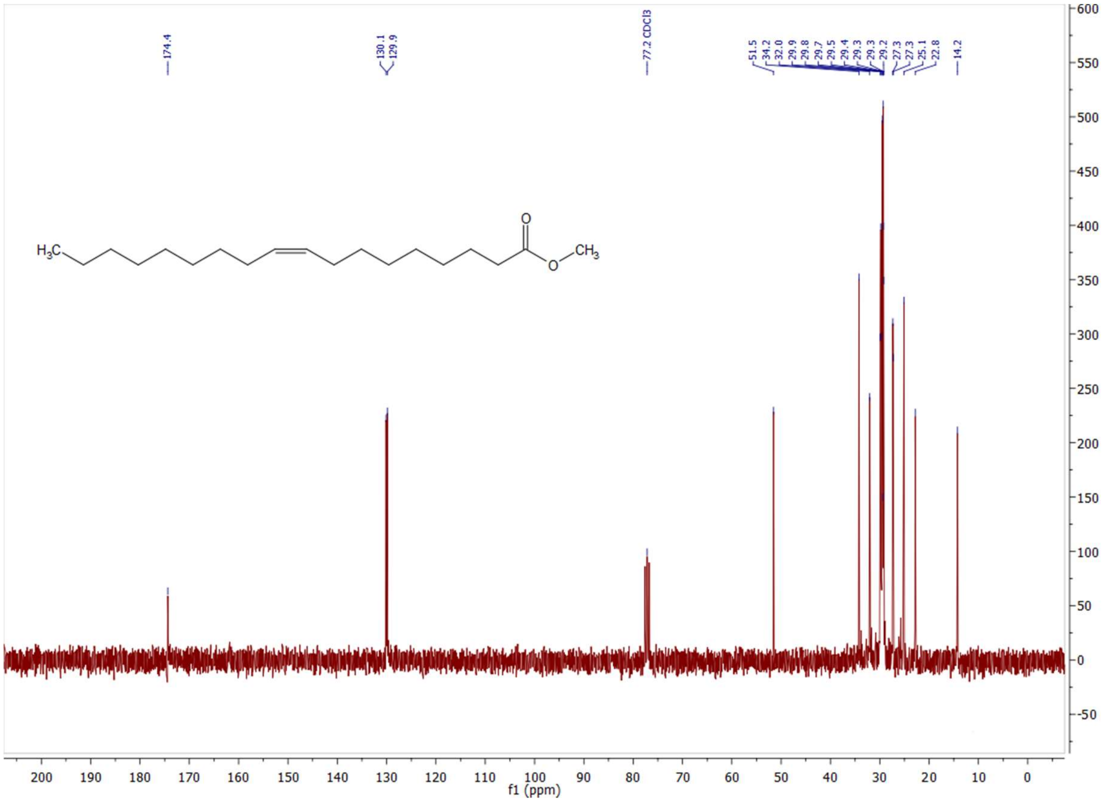

Methyl 4-(4-chlorophenyl)-4-oxobutanoate (23a)

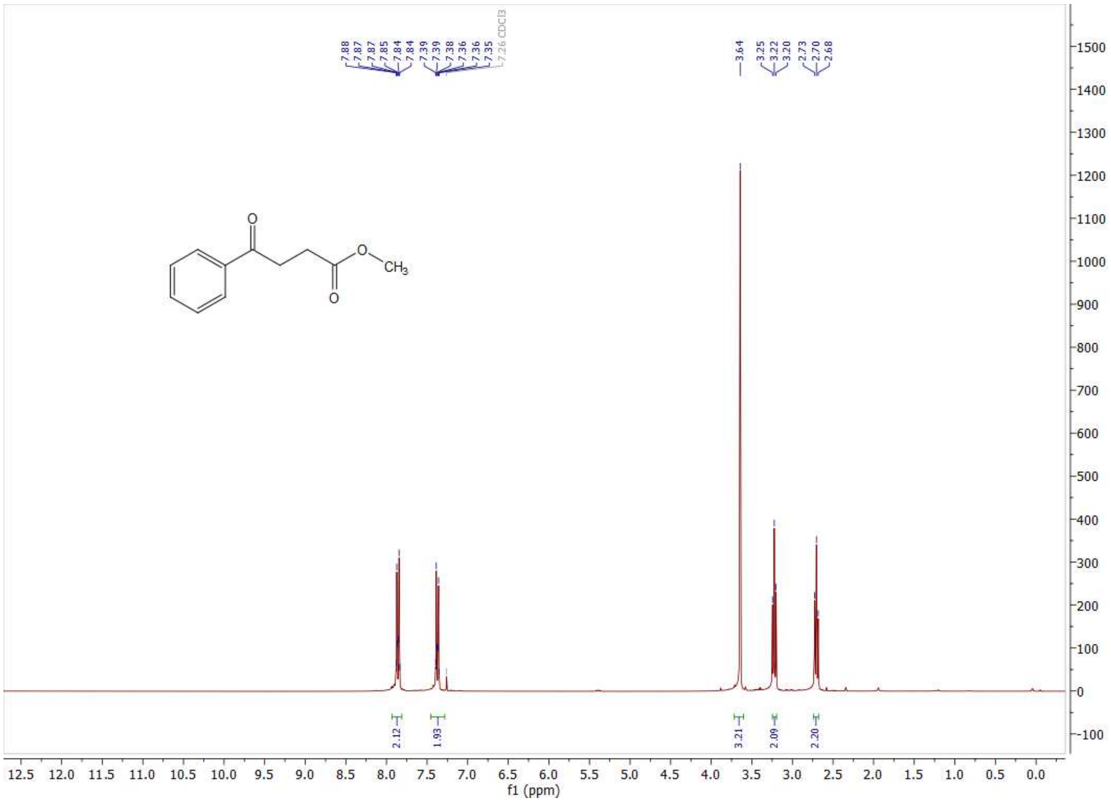

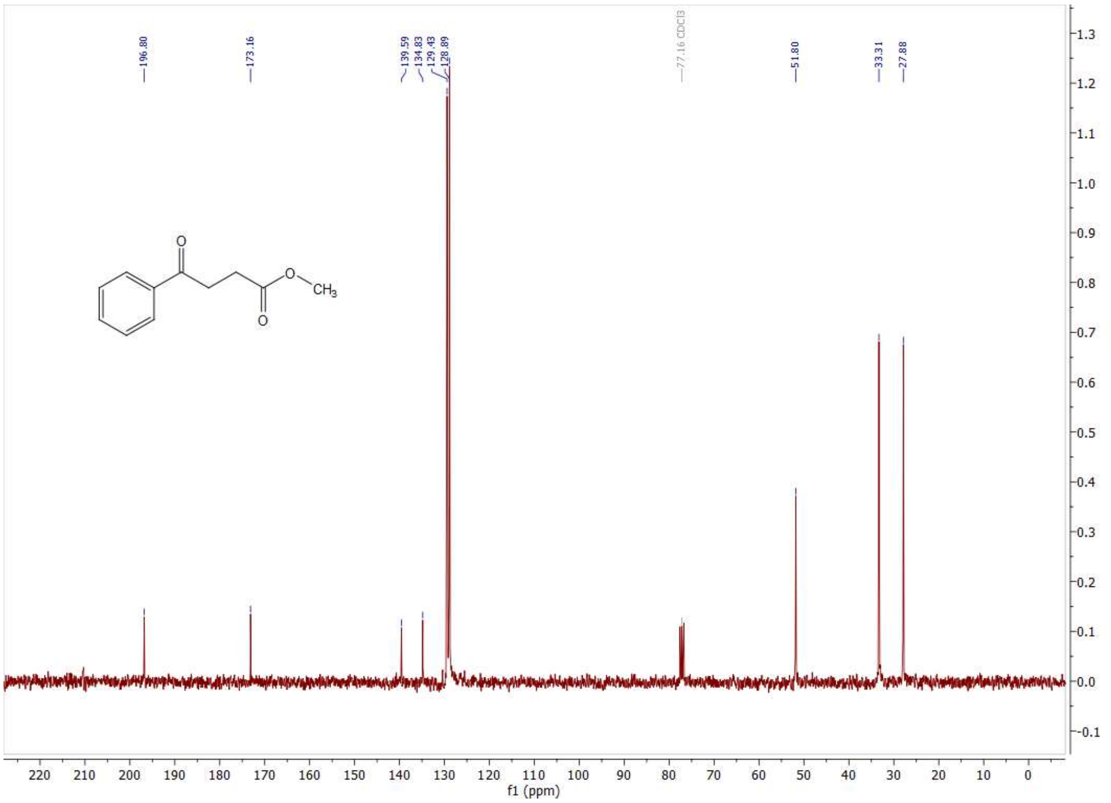

(*S*)-Methyl 2-acetamido-3-phenylpropanoate (24a)

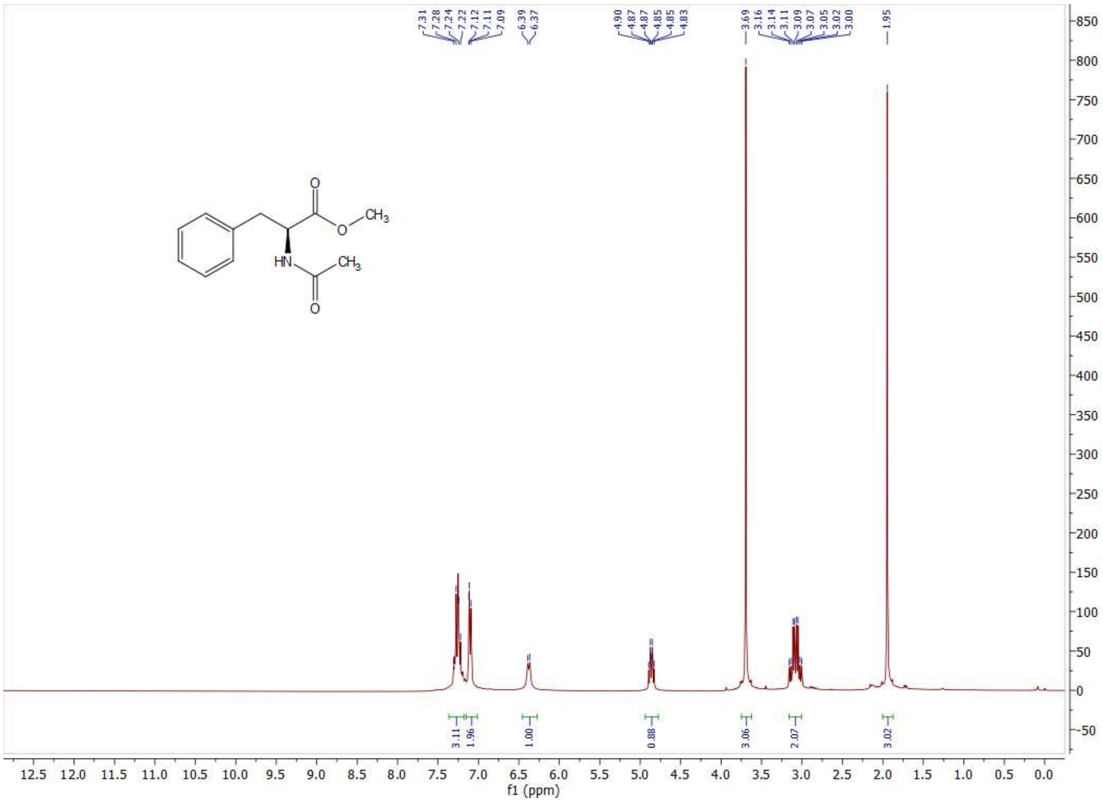

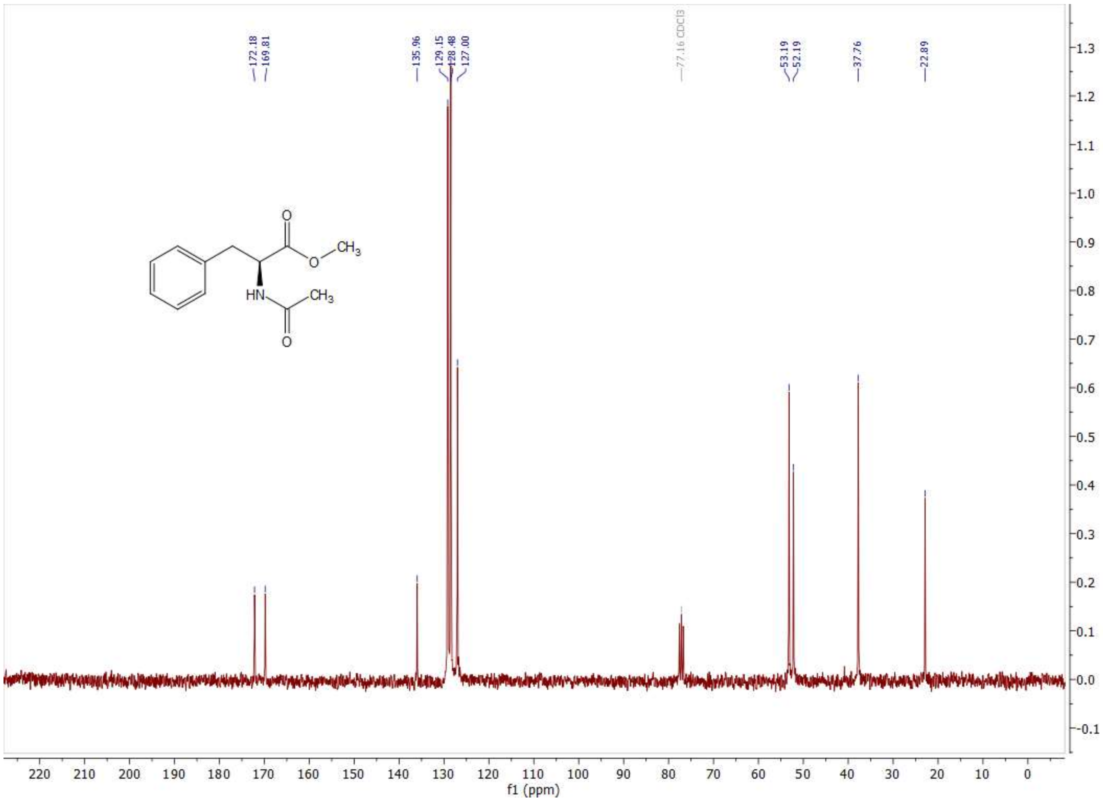

Methyl 2-(1H-indol-3-yl)acetate (25a)

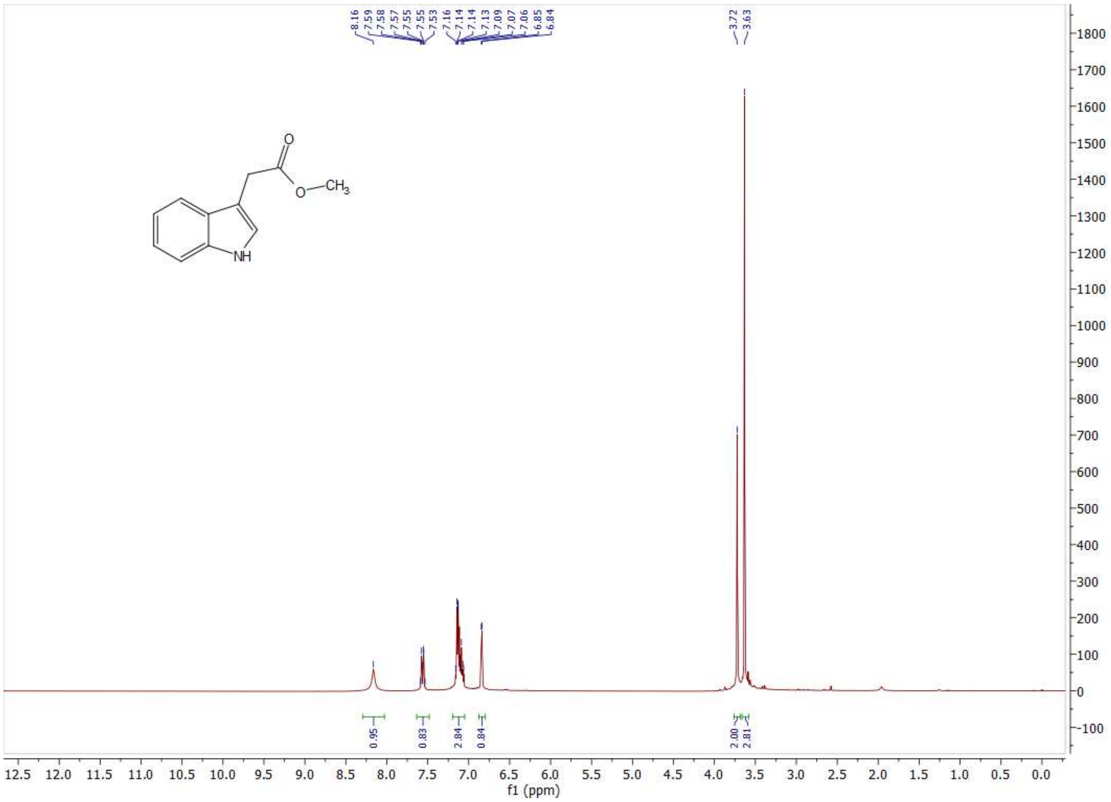

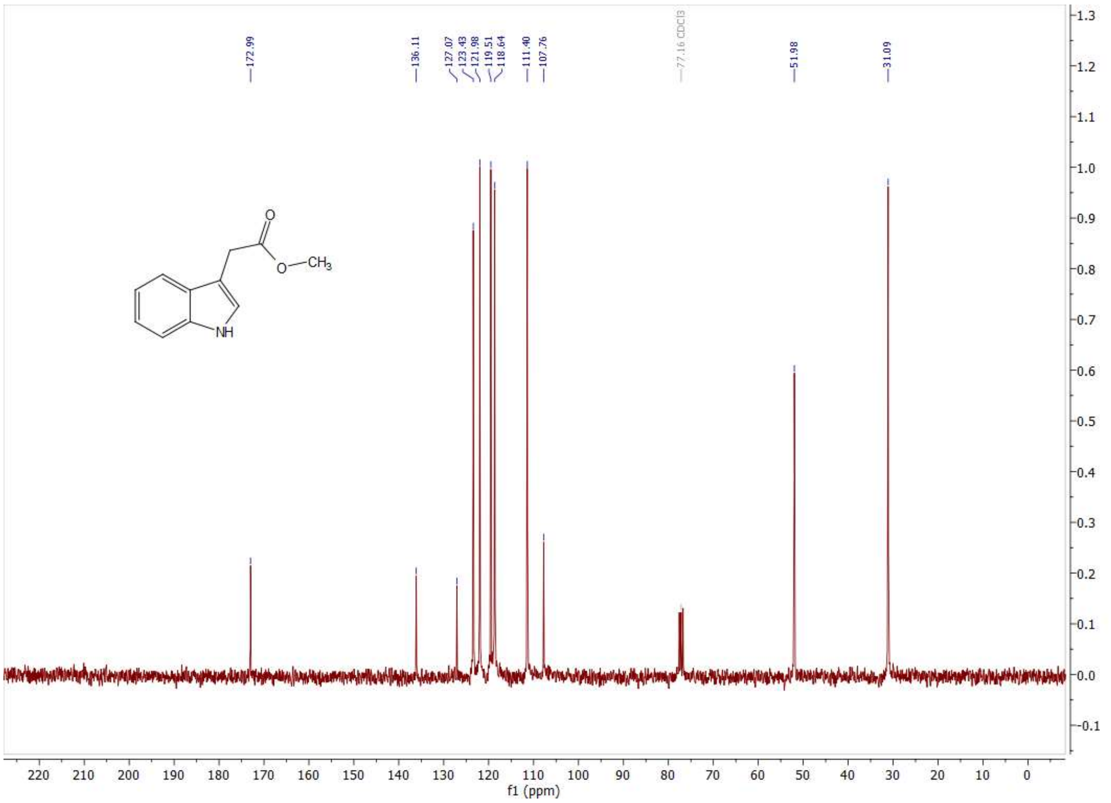

Methyl 2-cyanoacetate (26a)

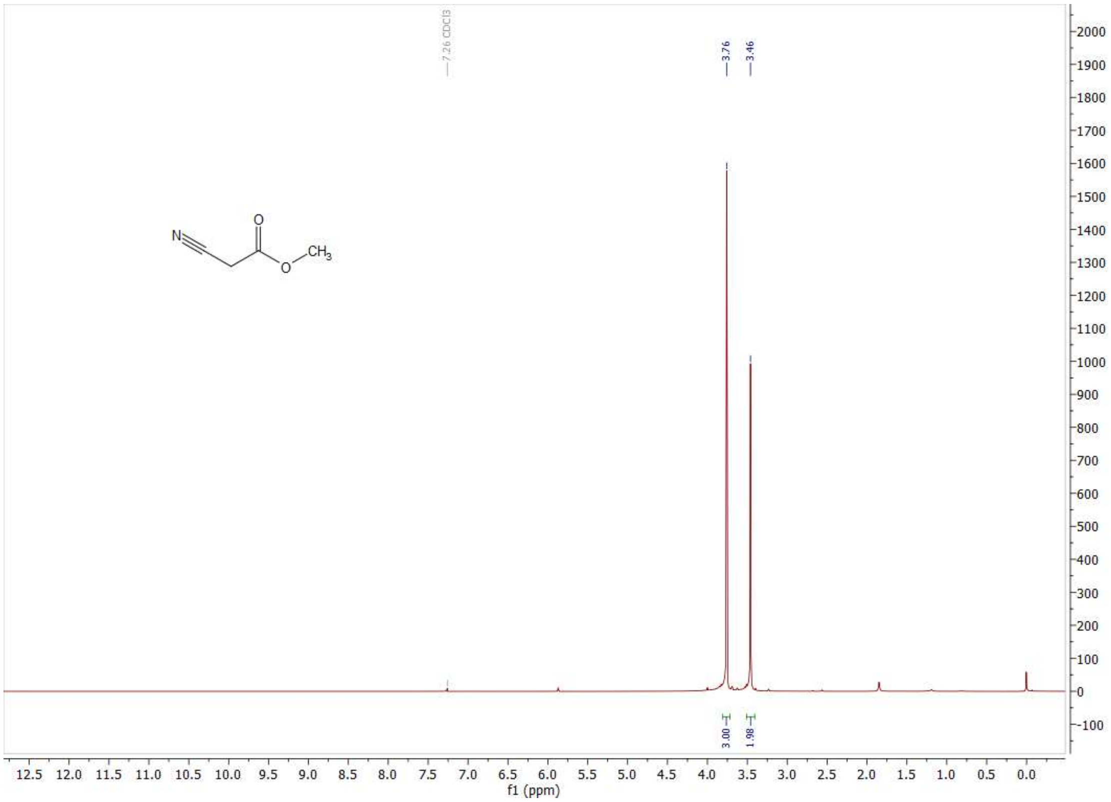

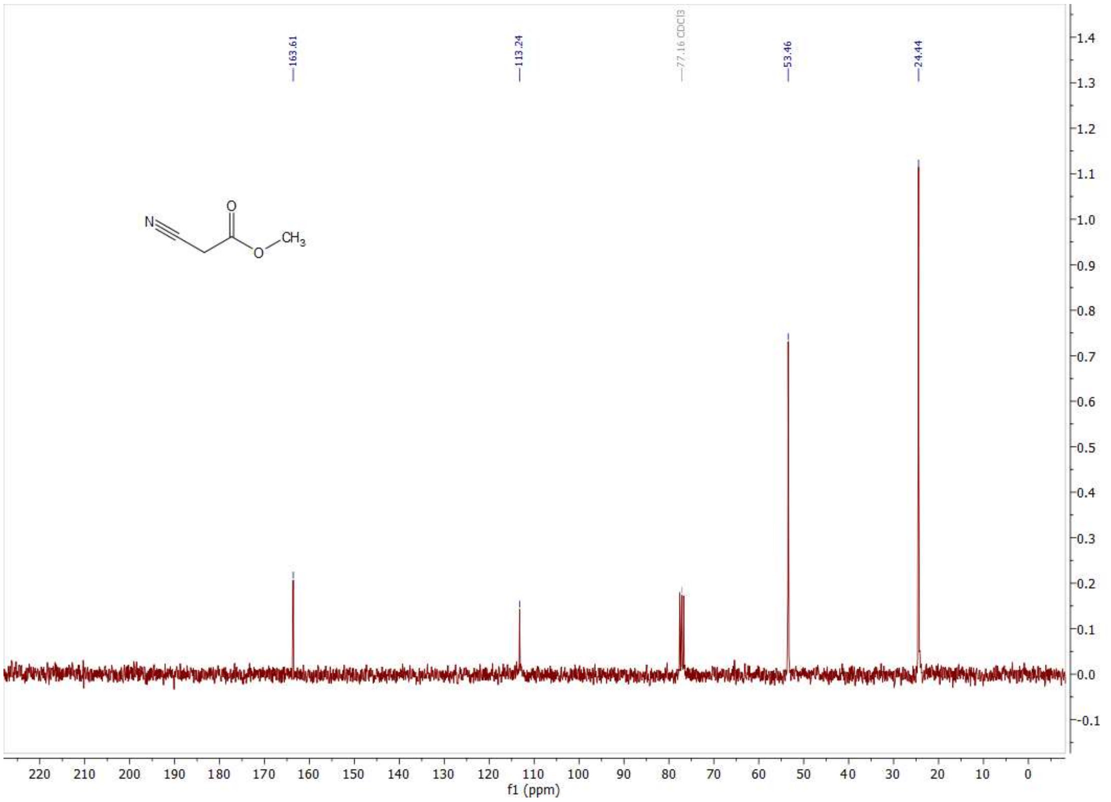

Adamantane-1-carboxylic acid methyl ester (27a)

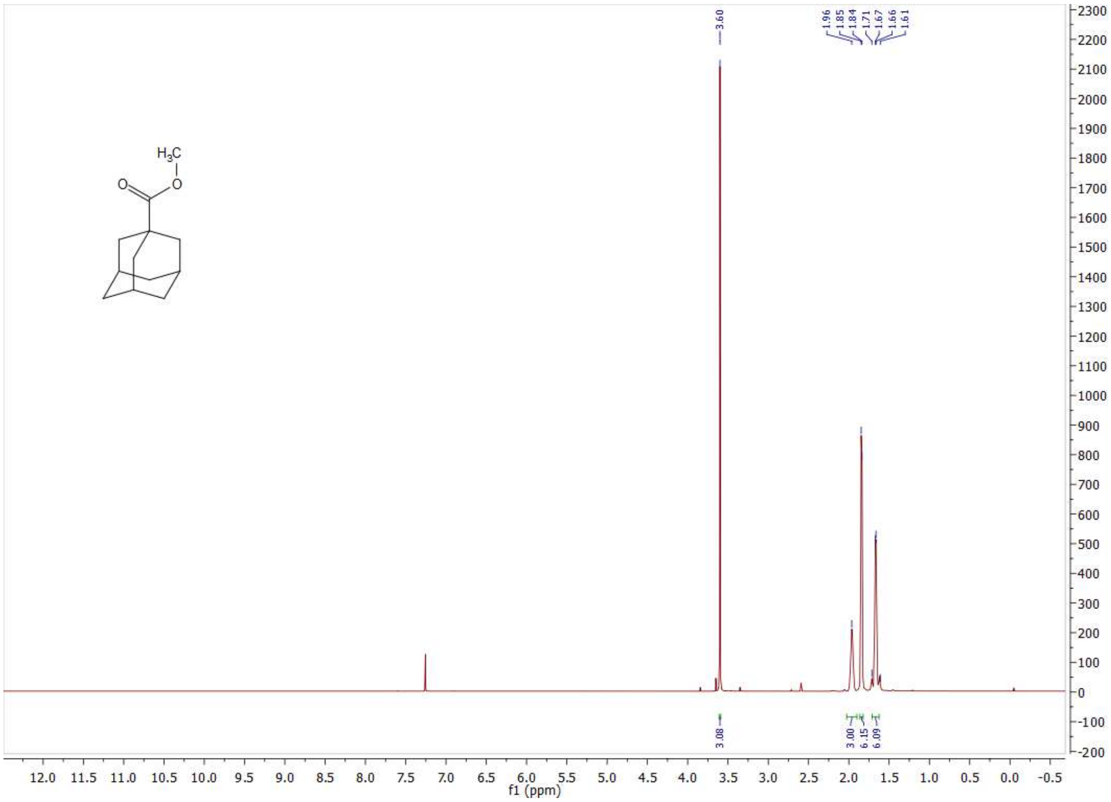

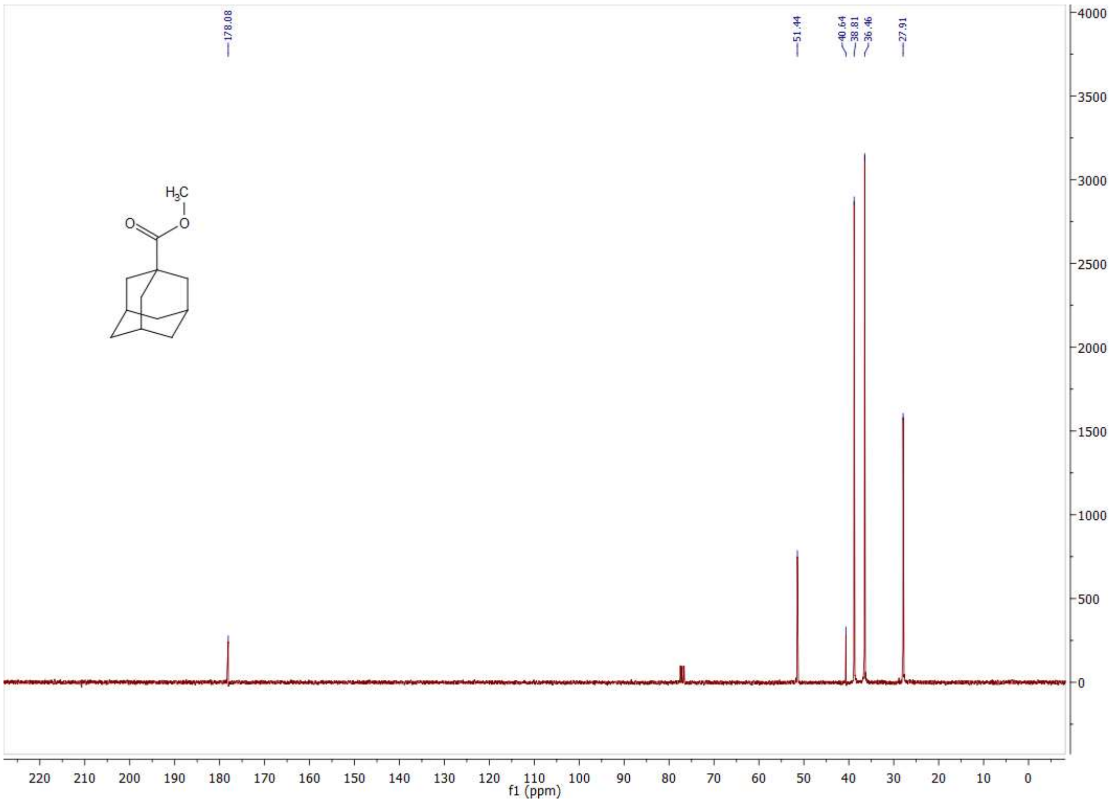

Dimethyl oxalate (28a)

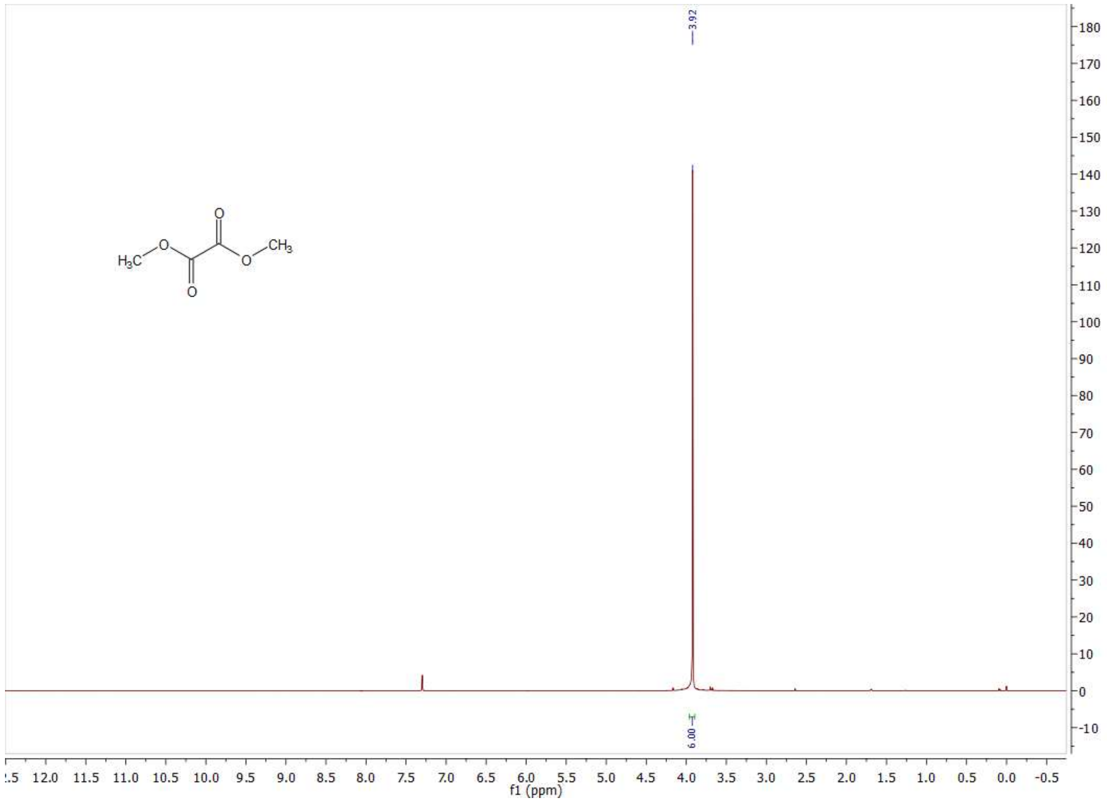

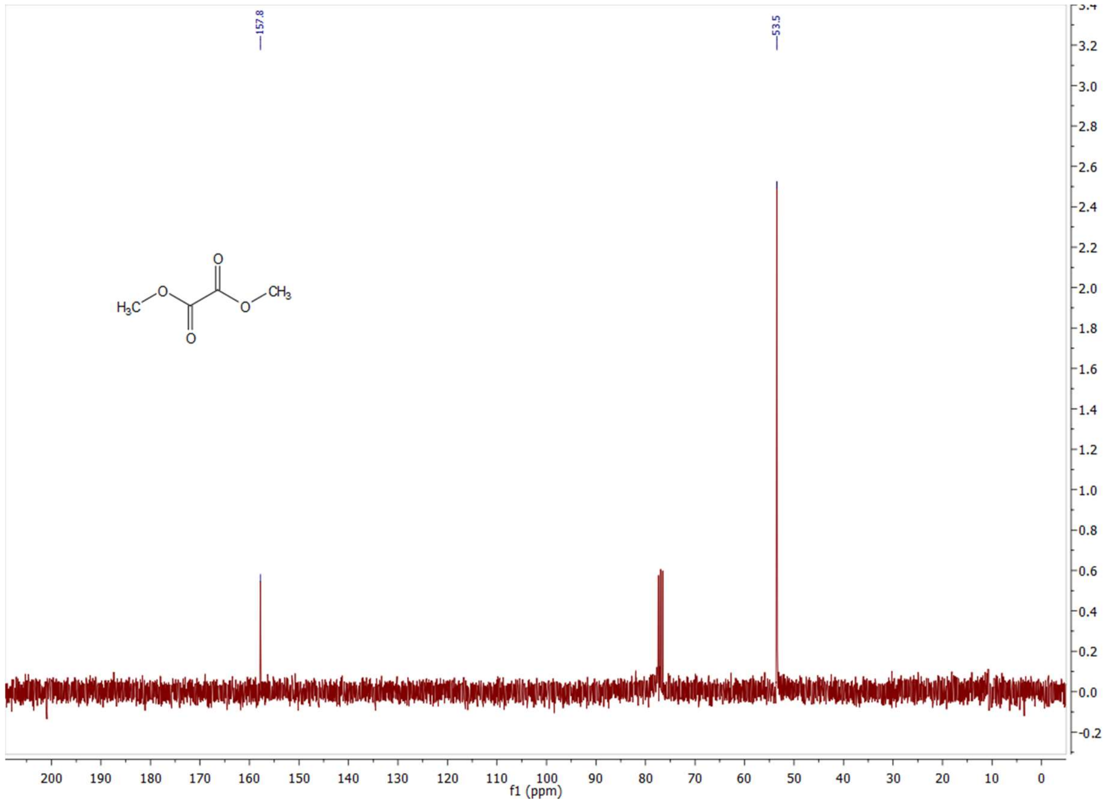

Trimethyl citrate (29a)

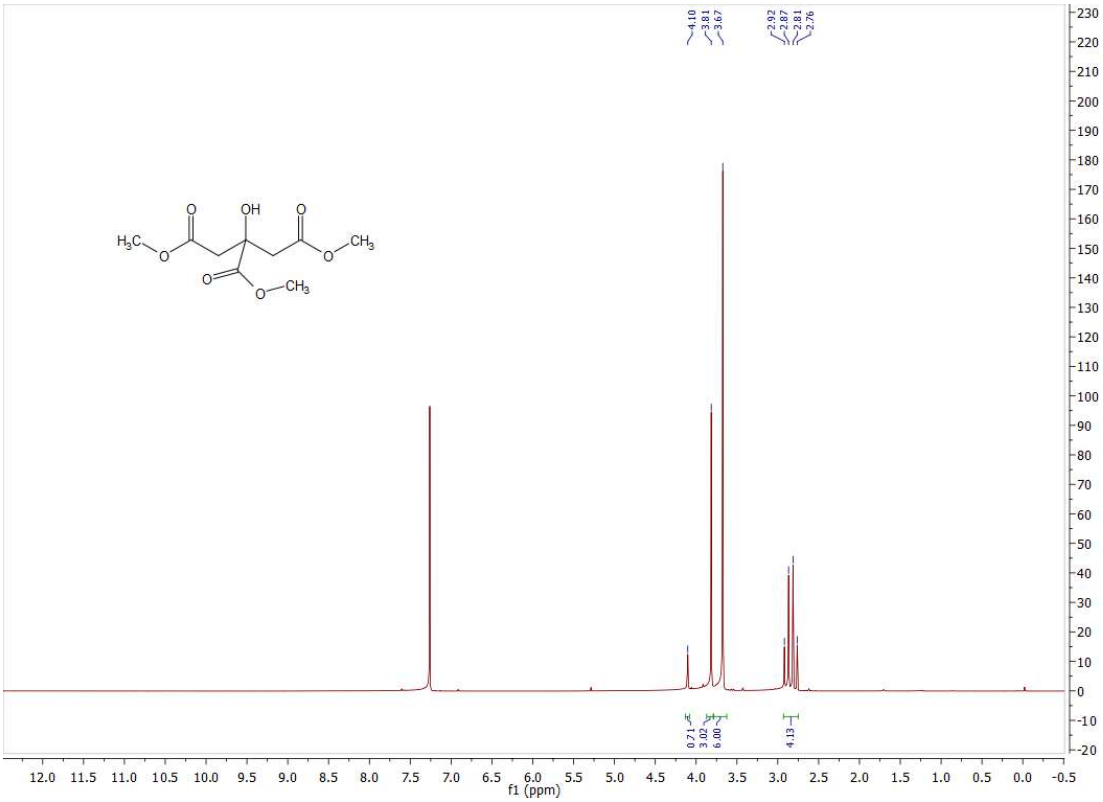

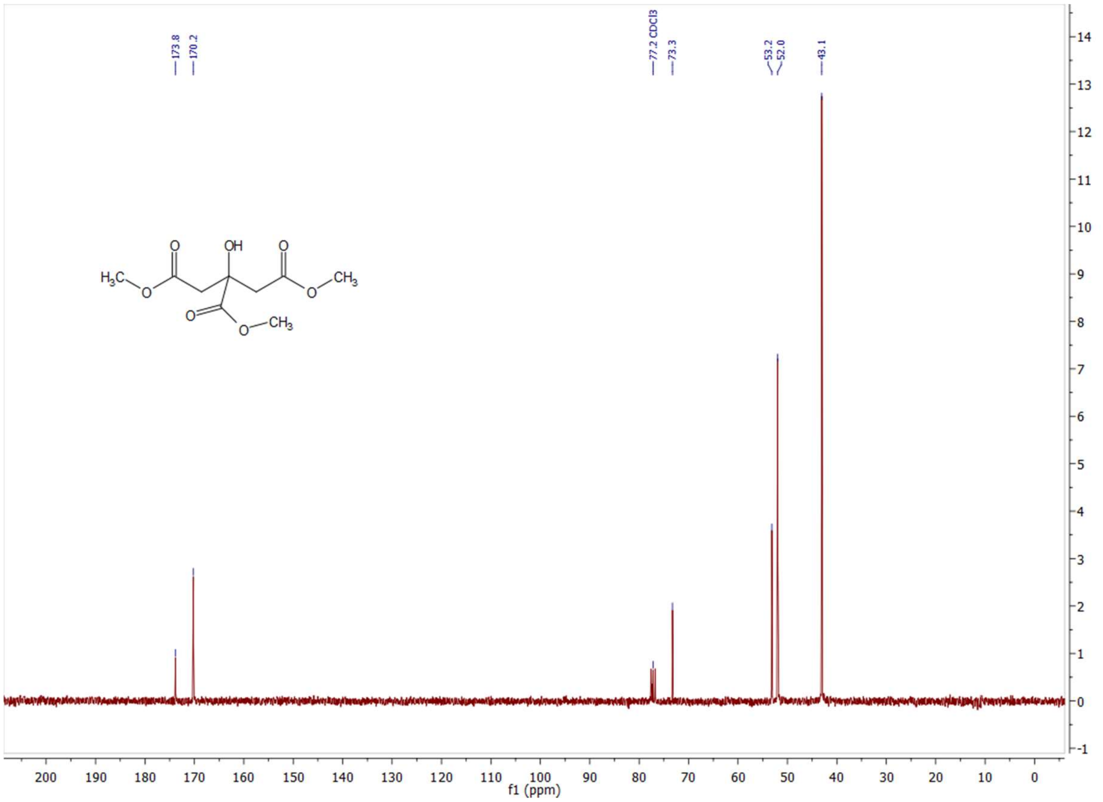

Cholic acid methyl ester (30a)

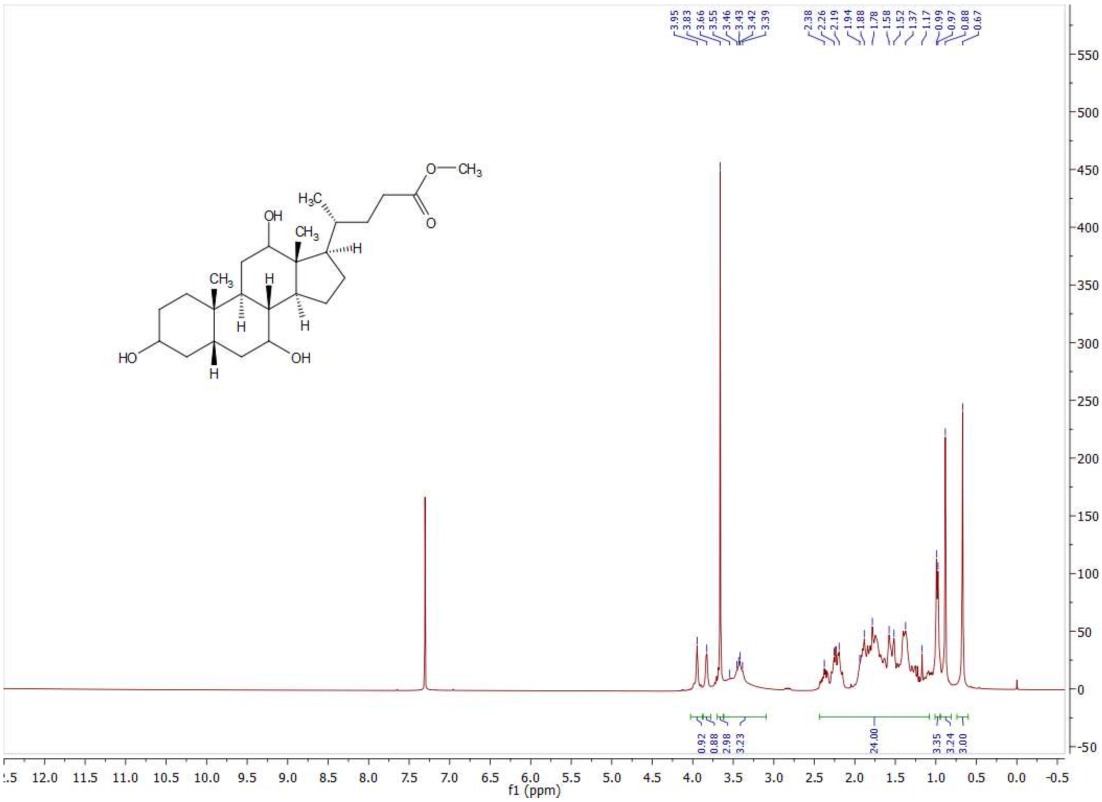

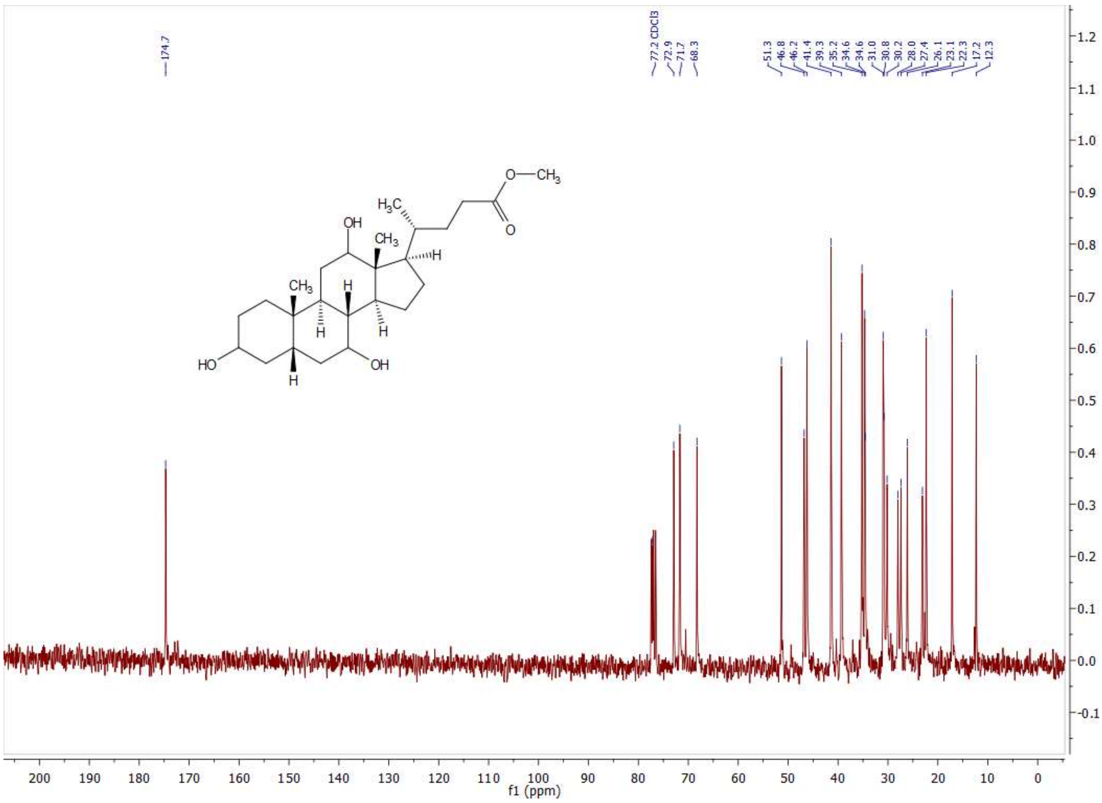

Hyodeoxycholic acid methyl ester (31a)

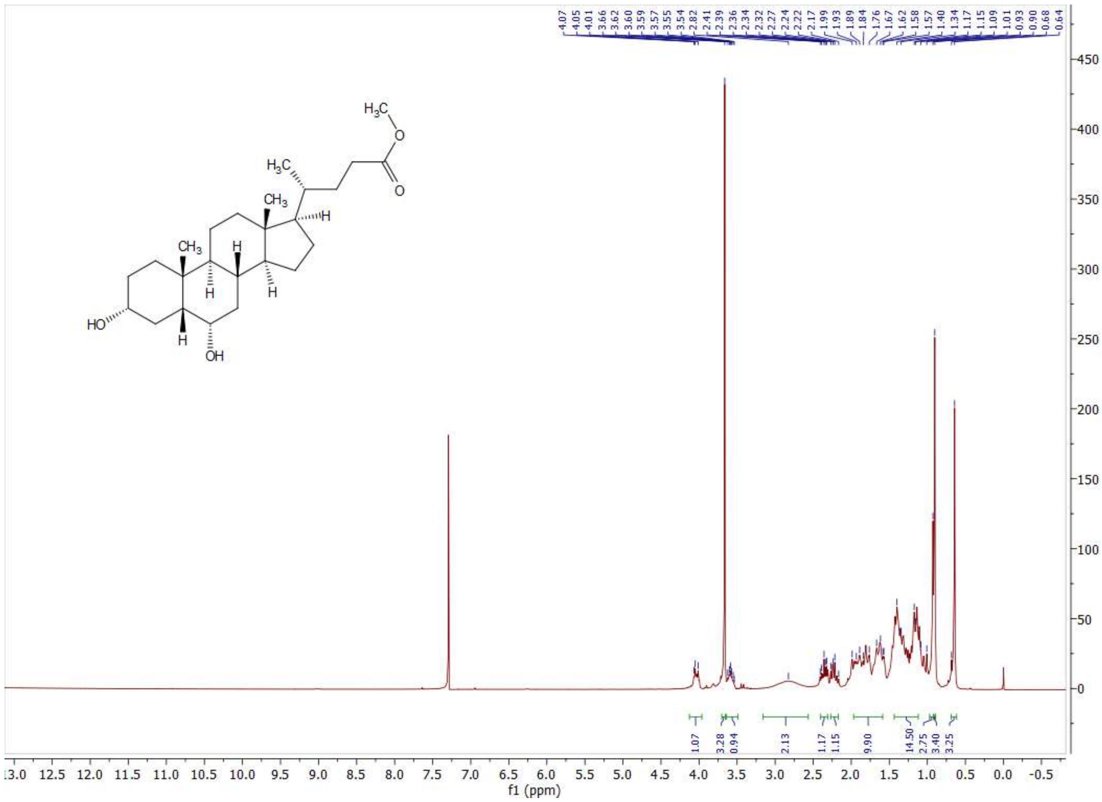

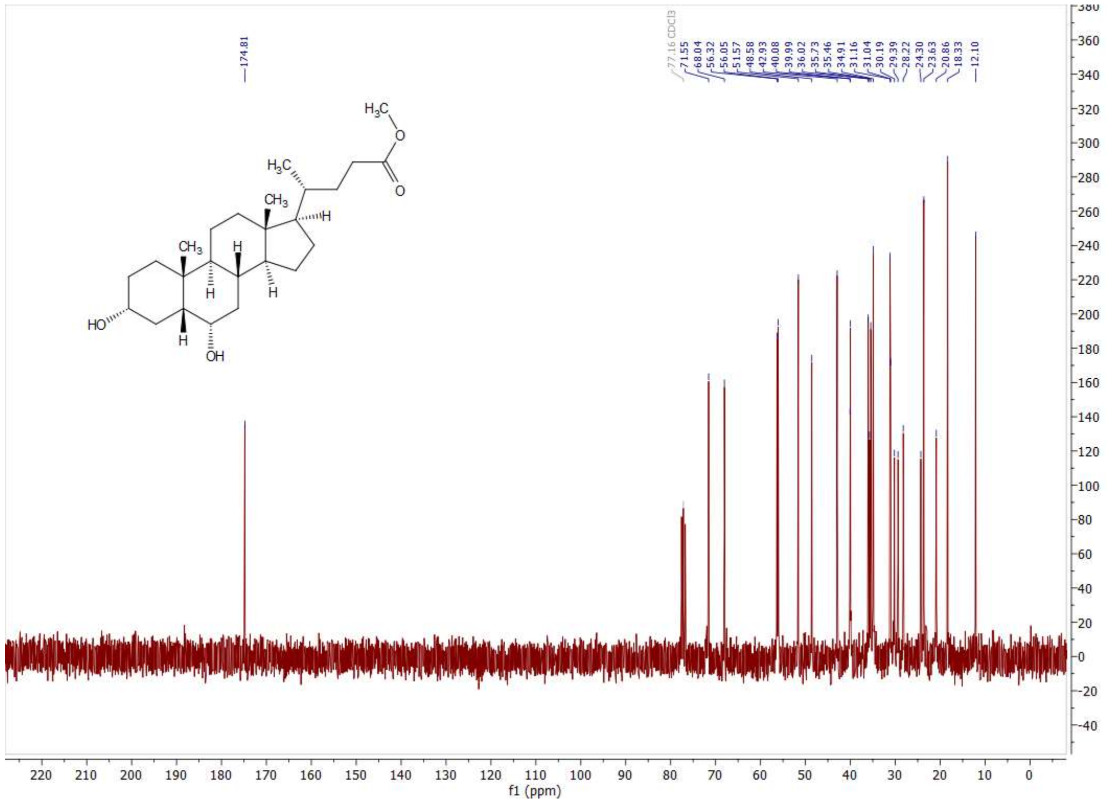

Litocholic acid methyl ester (32a)

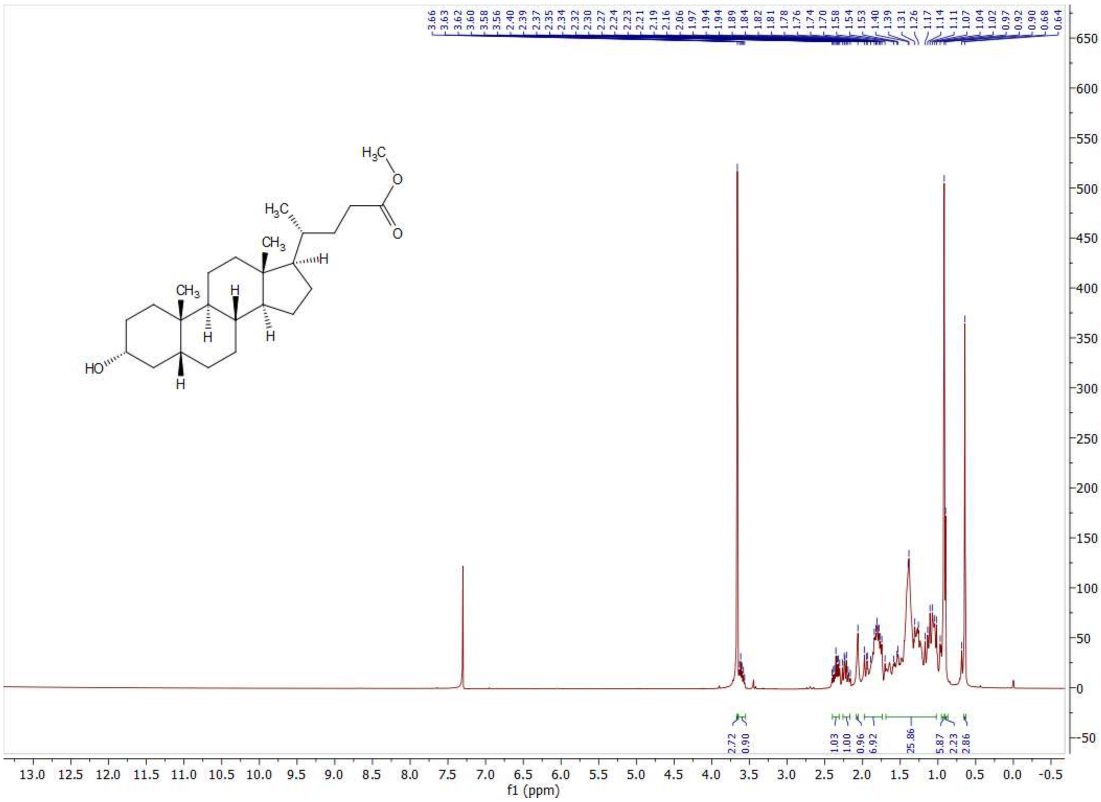

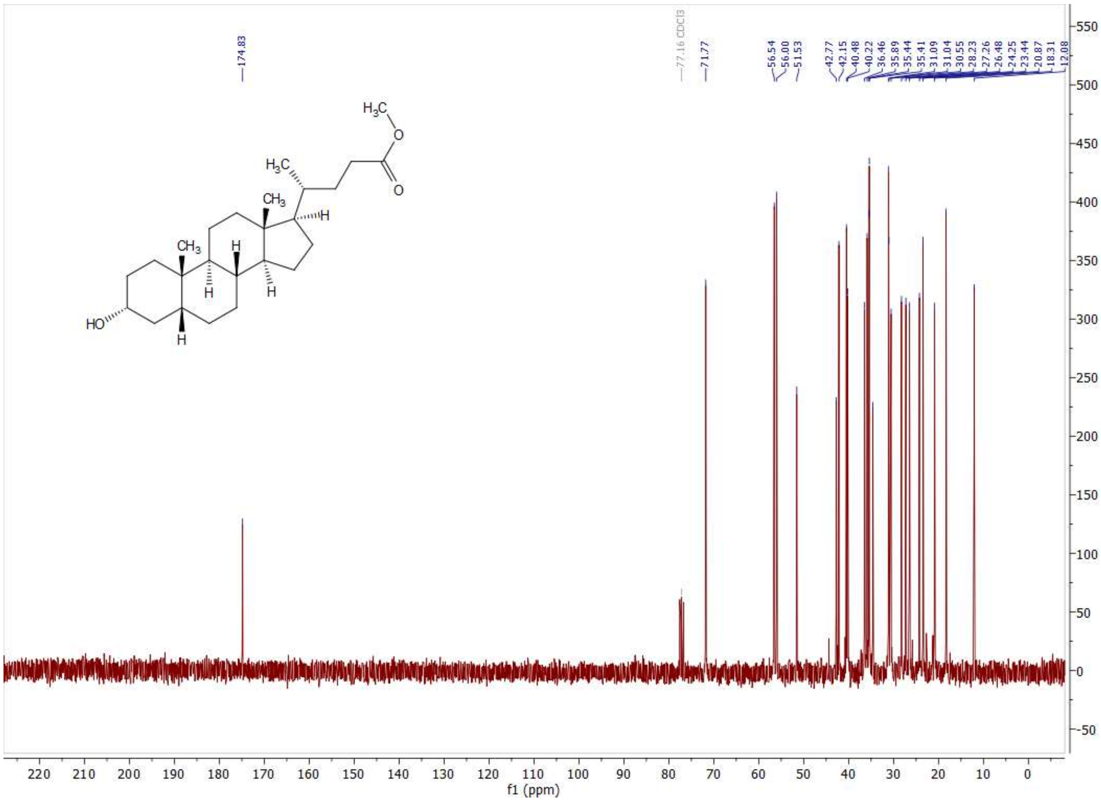

Dehydrocholic acid methyl ester (33a)

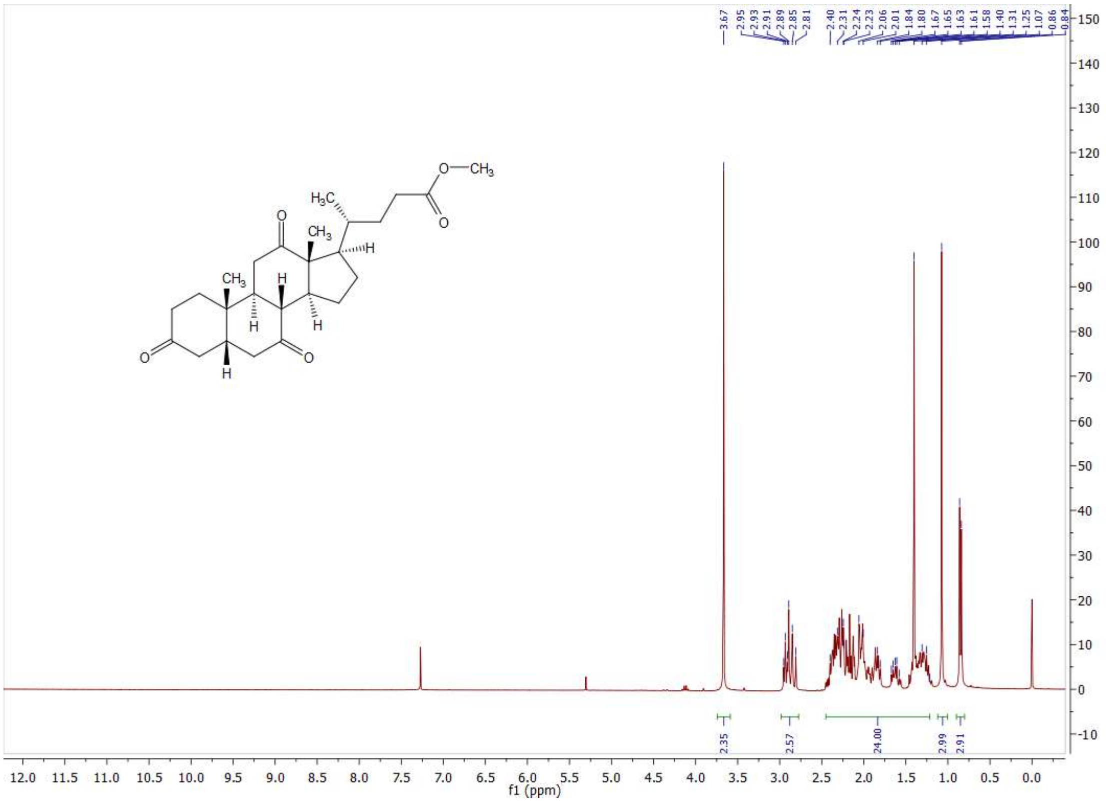

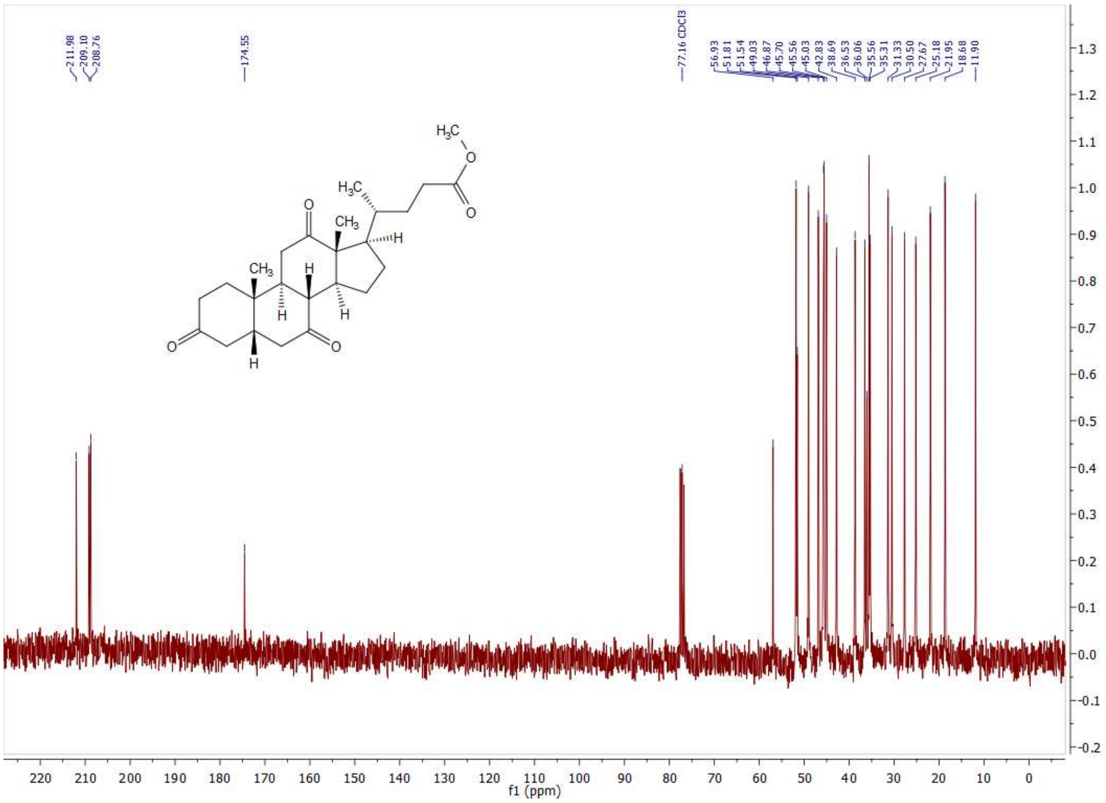

2-Fluoroethyl benzoate (1b)

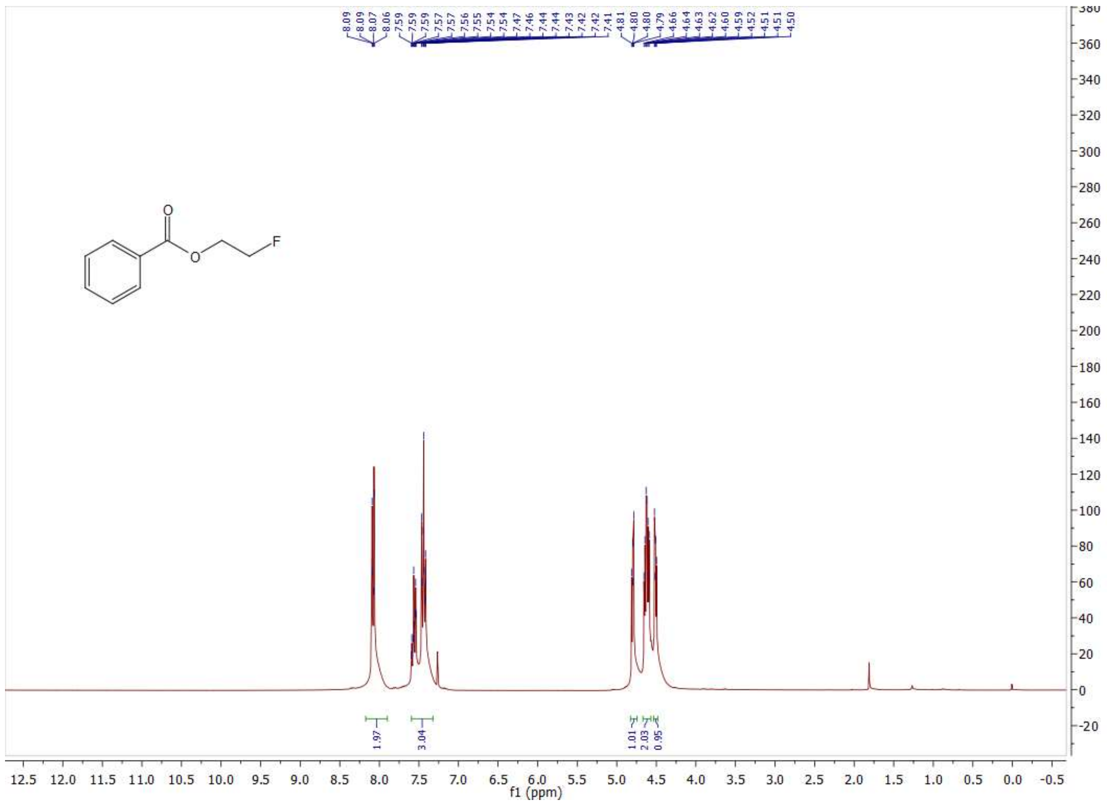

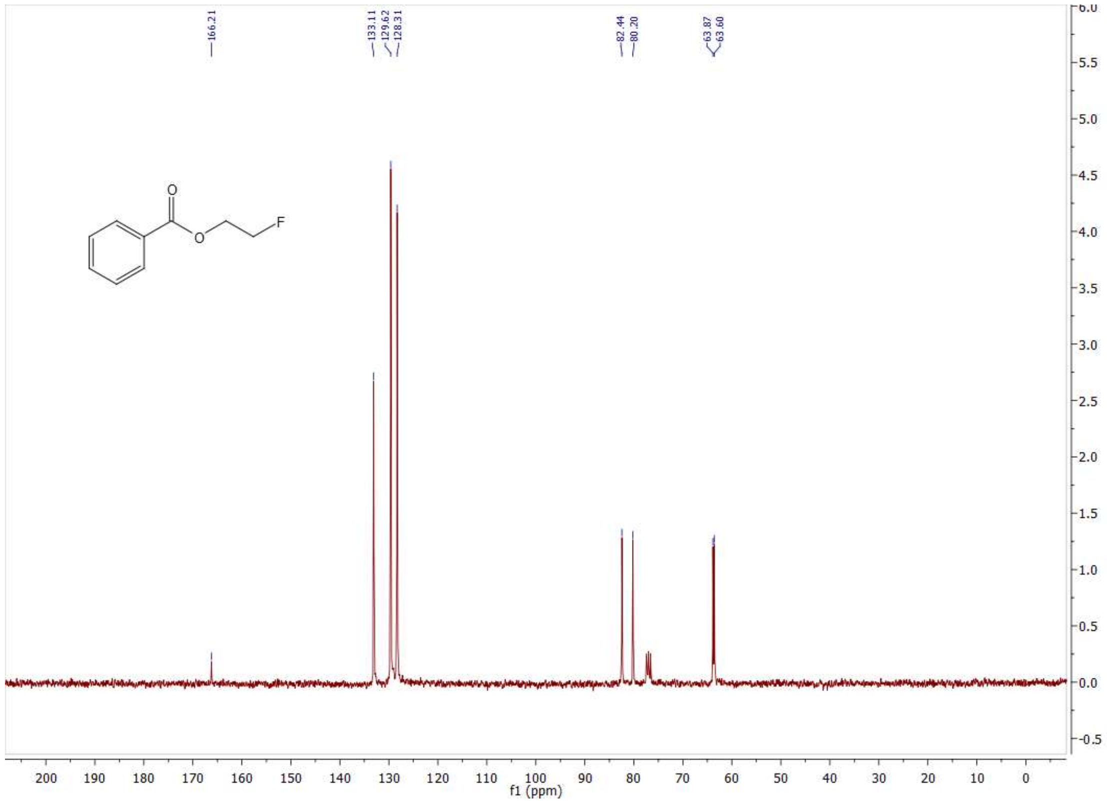

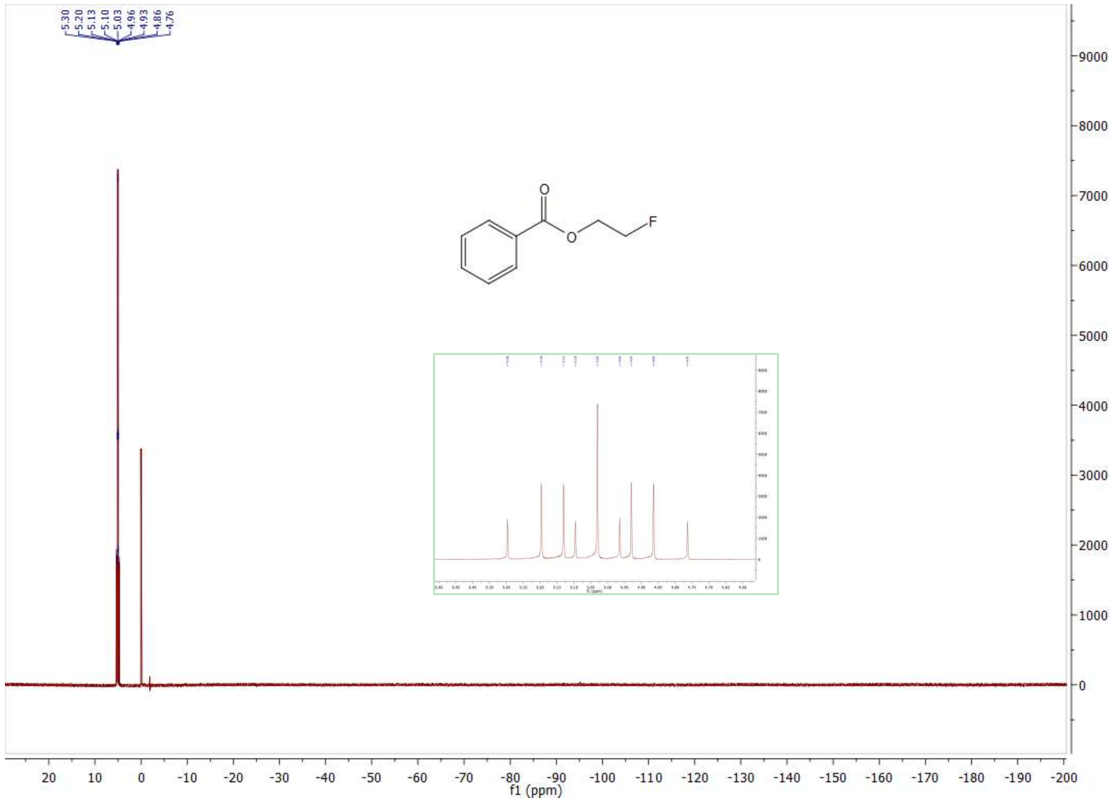
5.30
5.20
5.13
5.10
5.03
4.96
4.93
4.86
4.76
O
O
F
9000
8000
7000
6000
5000
4000
3000
2000
1000
0
20
10
0
-10
-20
-30
-40
-50
-60
-70
-80
-90
-100
-110
-120
-130
-140
-150
-160
-170
-180
-190
-200
f1 (ppm)

2-Fluoroethyl octanoate (2b)

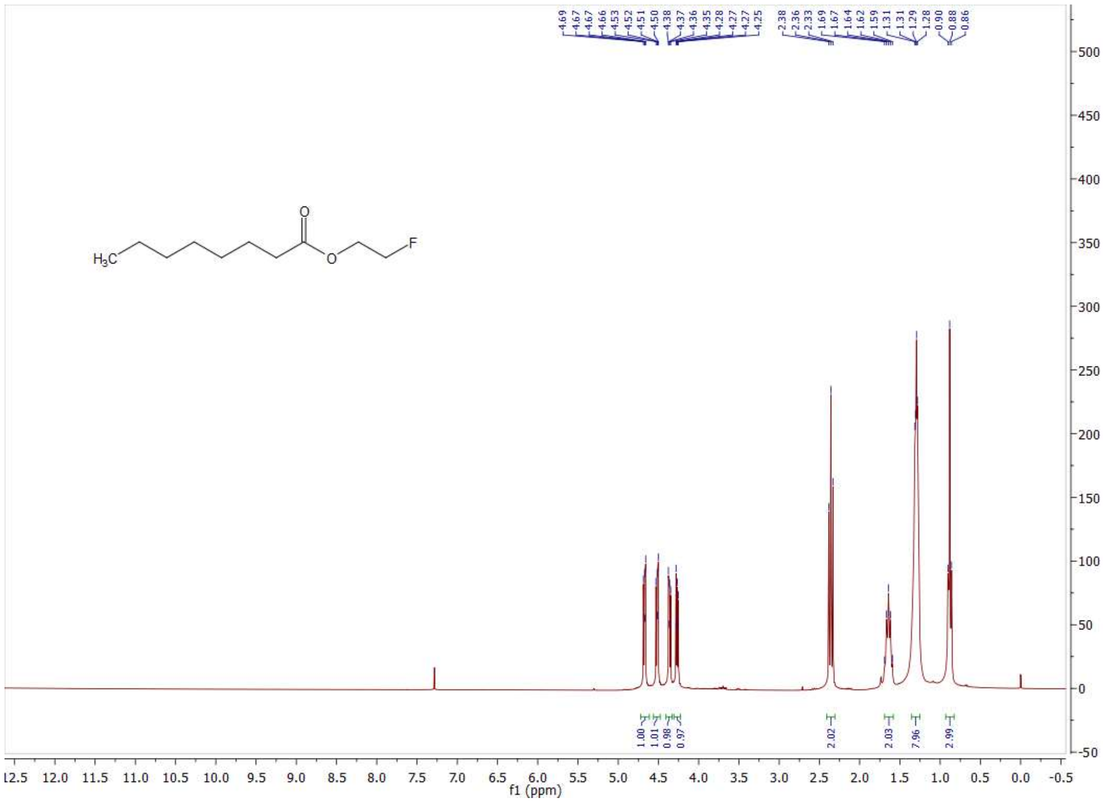

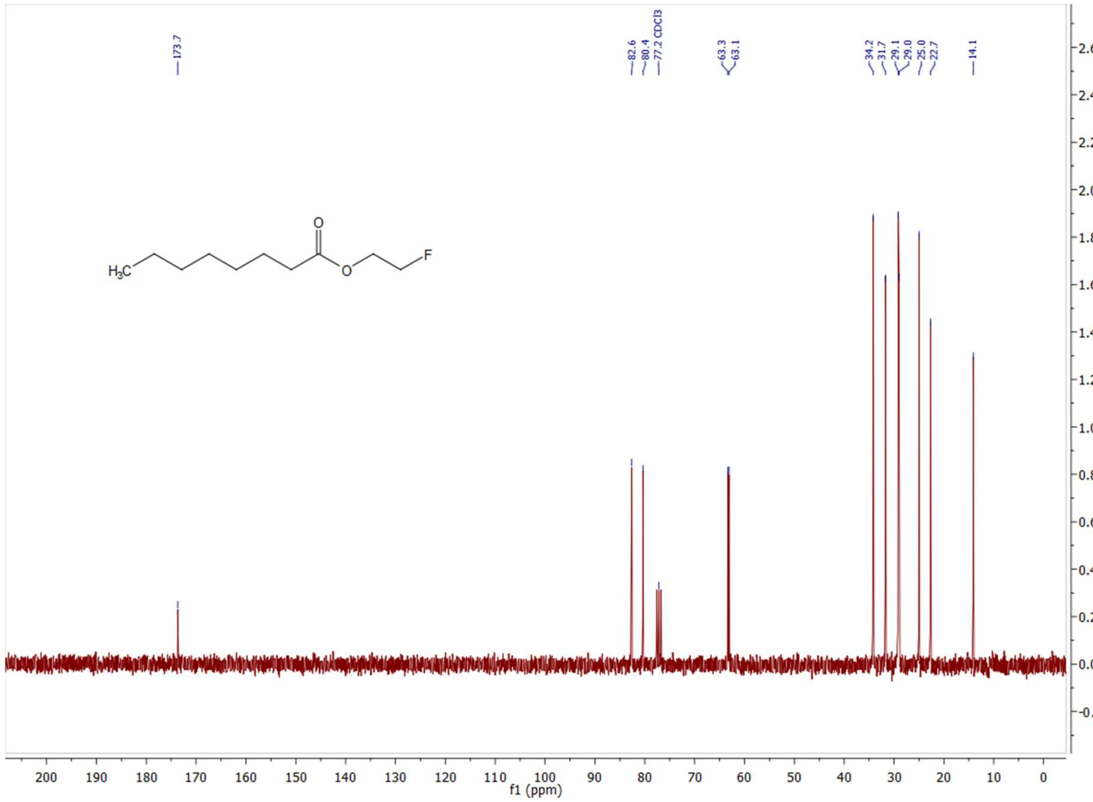

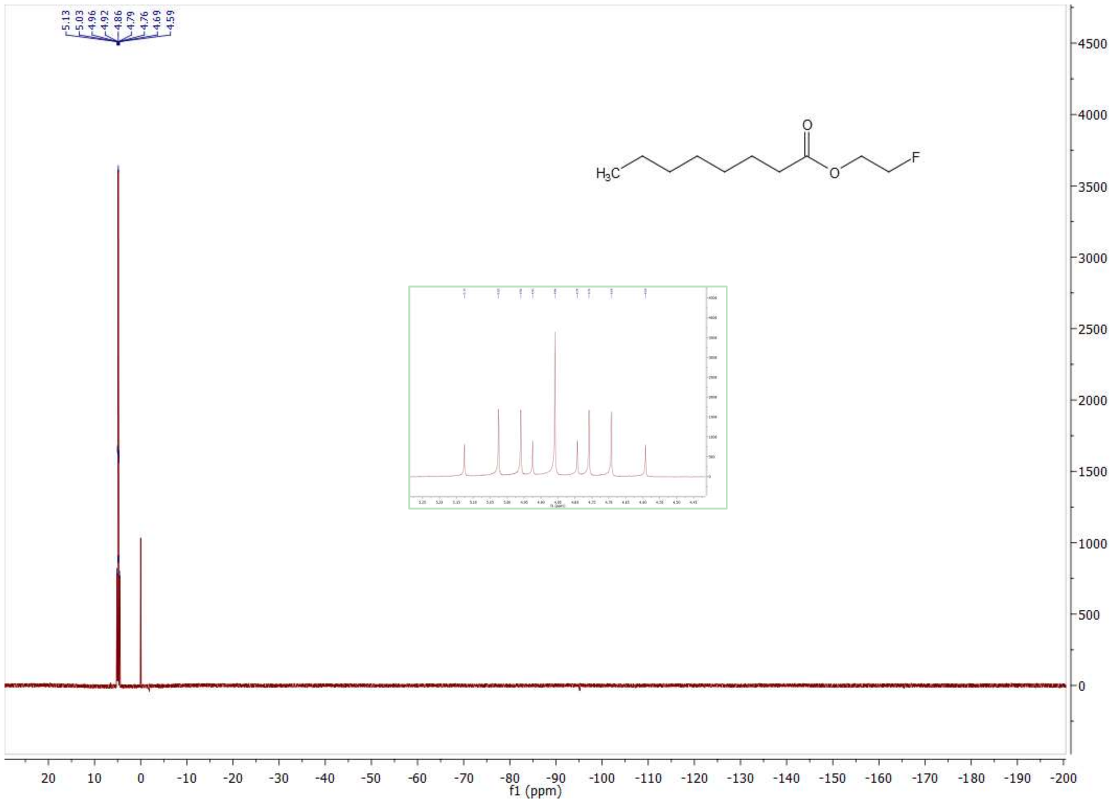

5.13
5.03
4.96
4.92
4.86
4.79
4.76
4.69
4.59
H3C
O
O
F
f1 (ppm)

Isopropyl benzoate (1c)

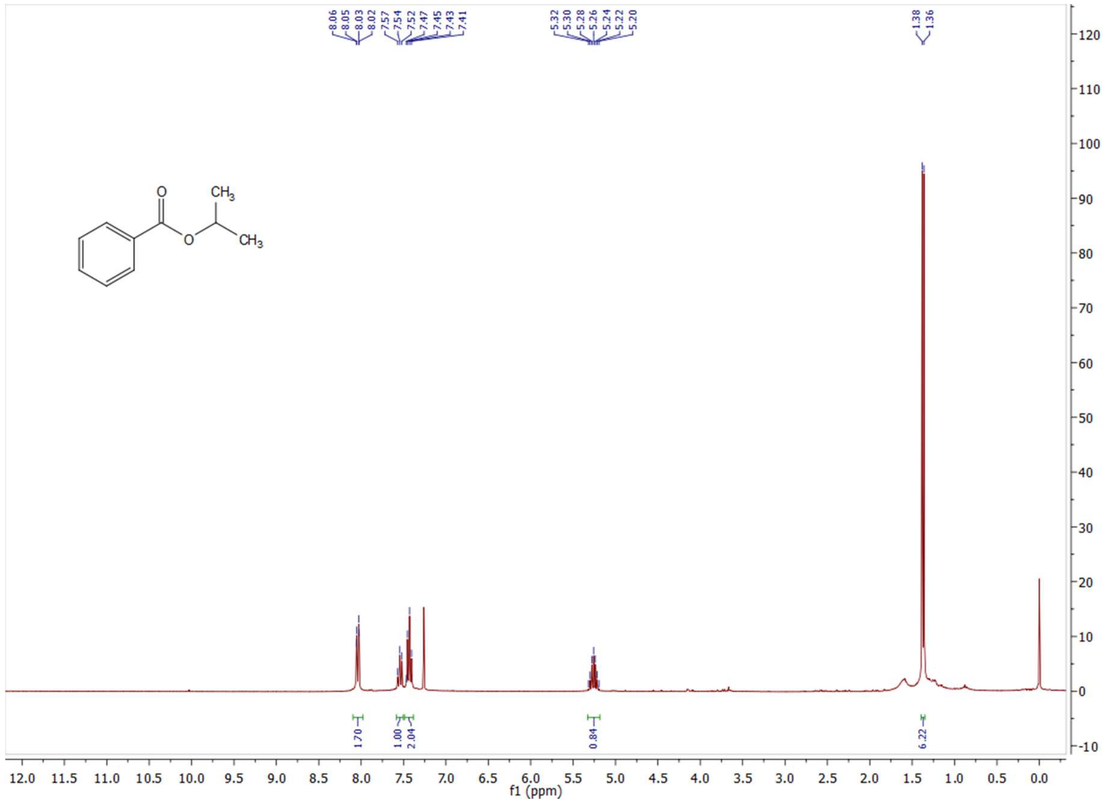

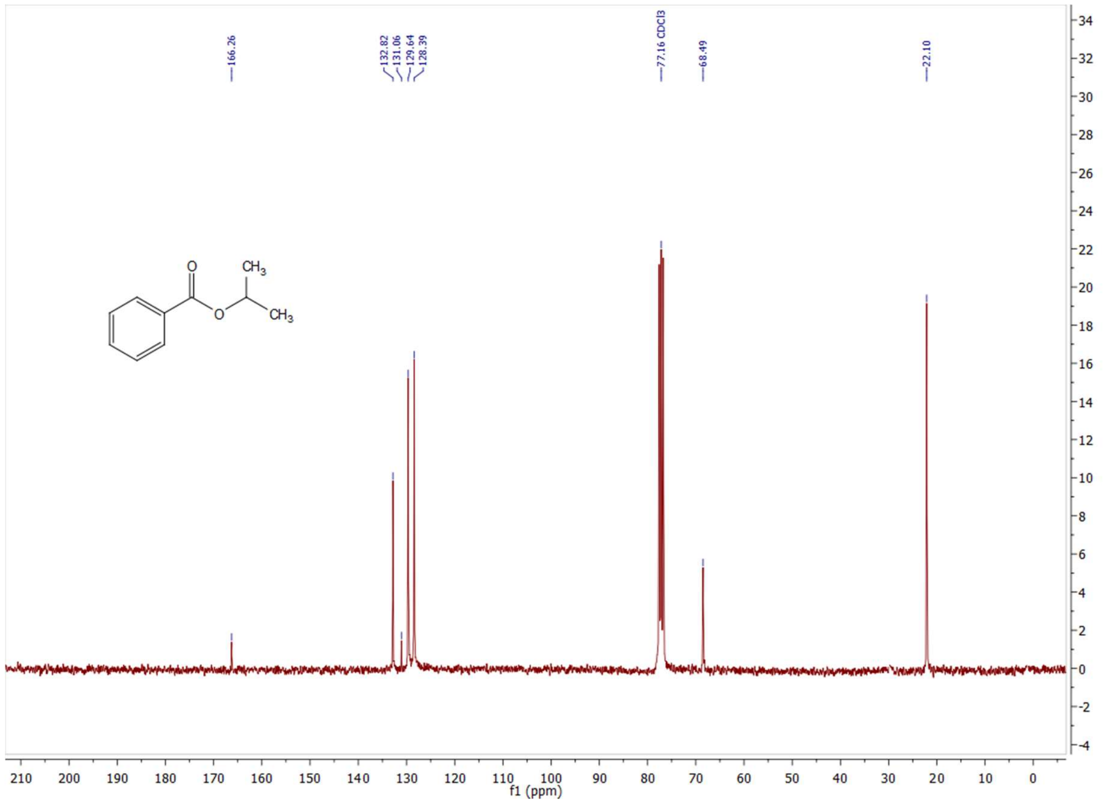

Isopropyl octanoate (2c)

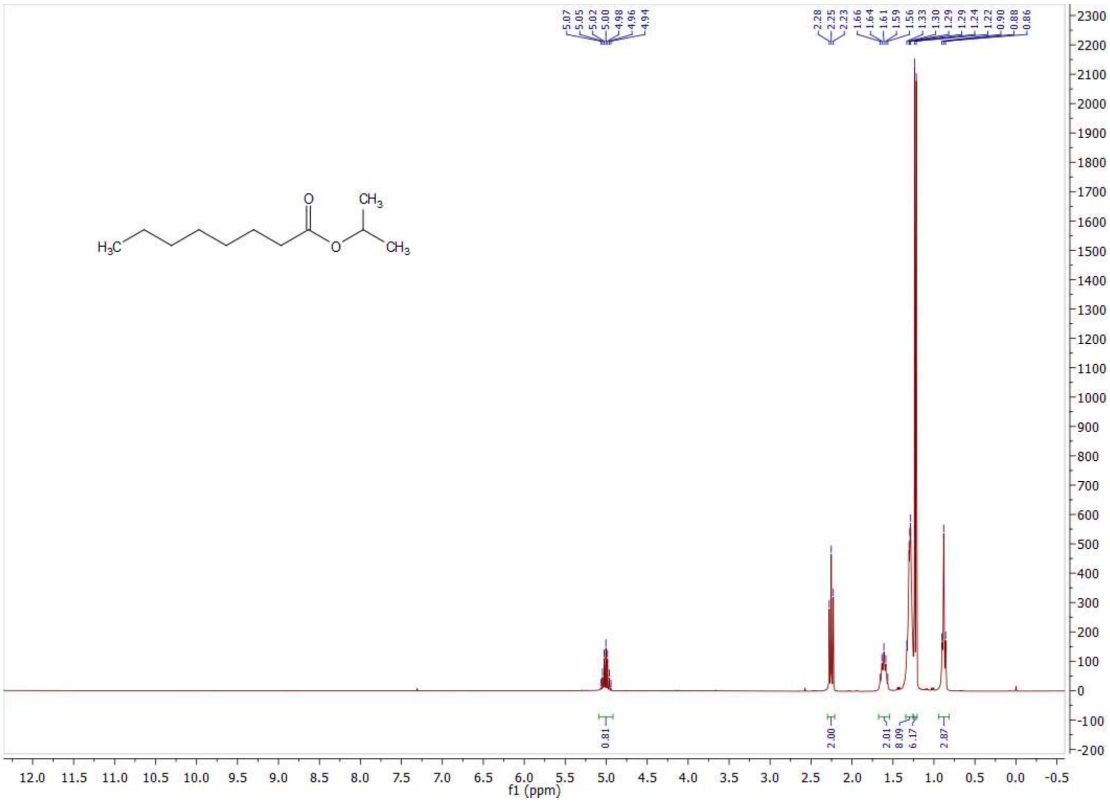

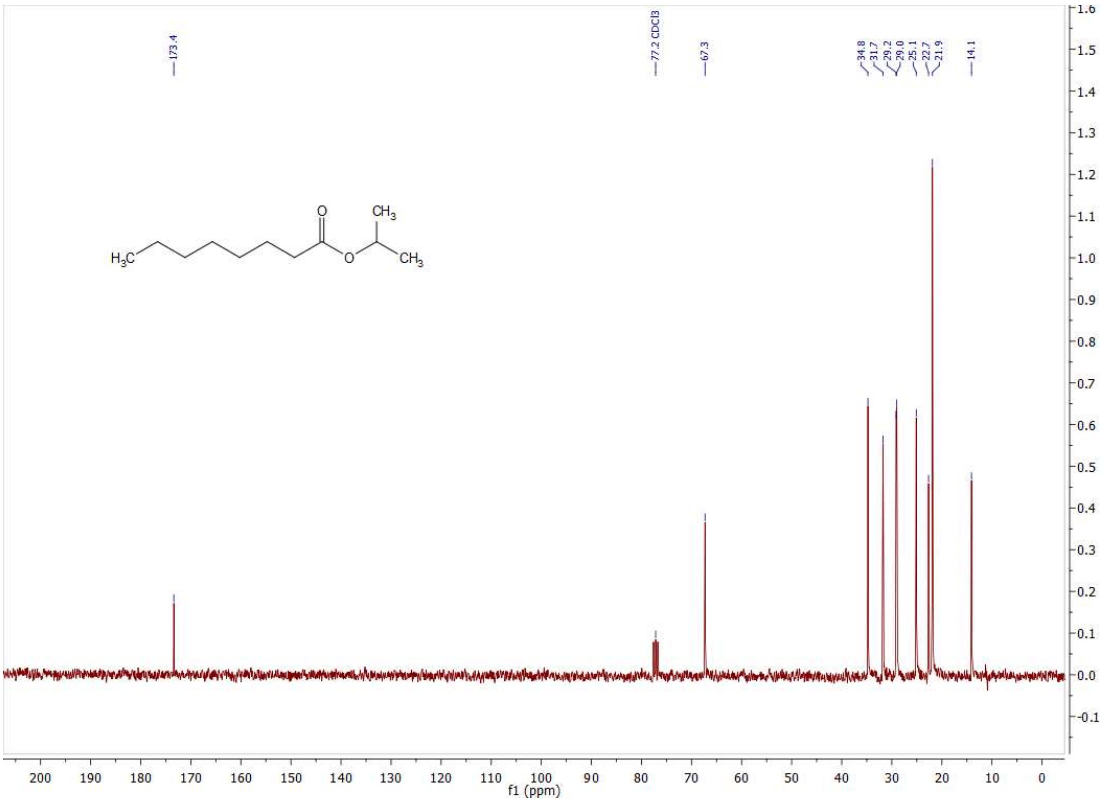

Cholic acid isopropyl ester (30c)

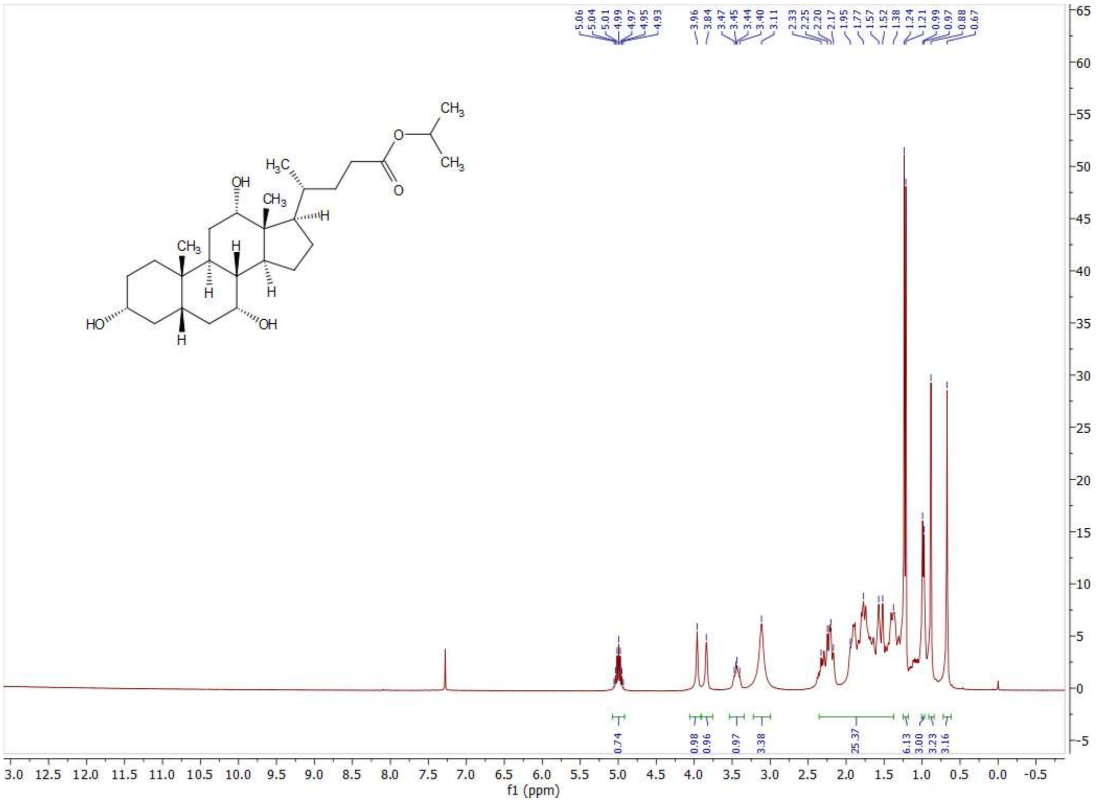

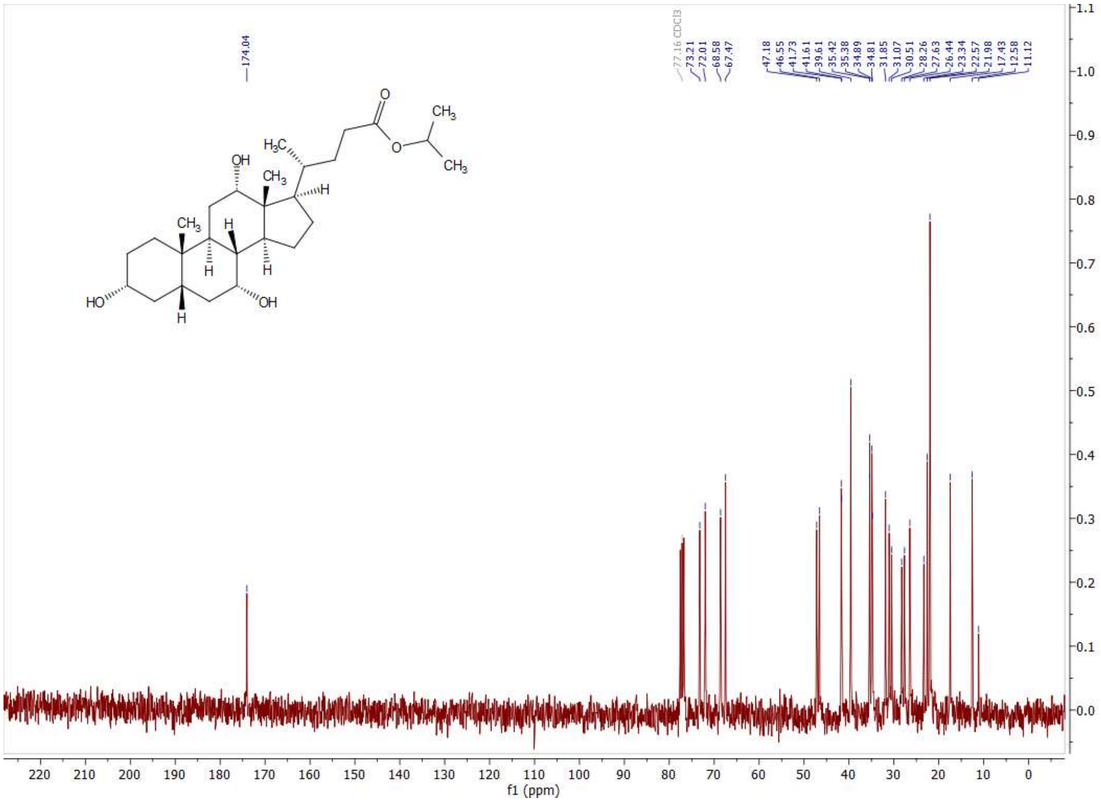

Litocholic acid isopropyl ester (32c)

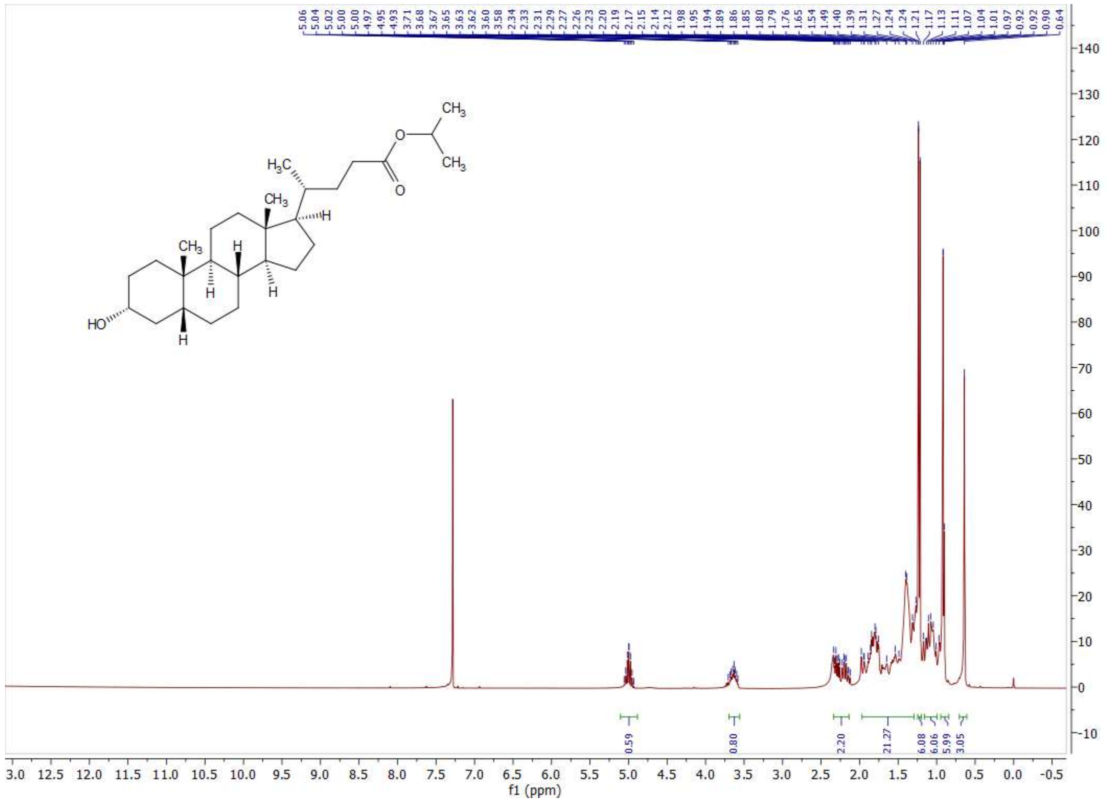

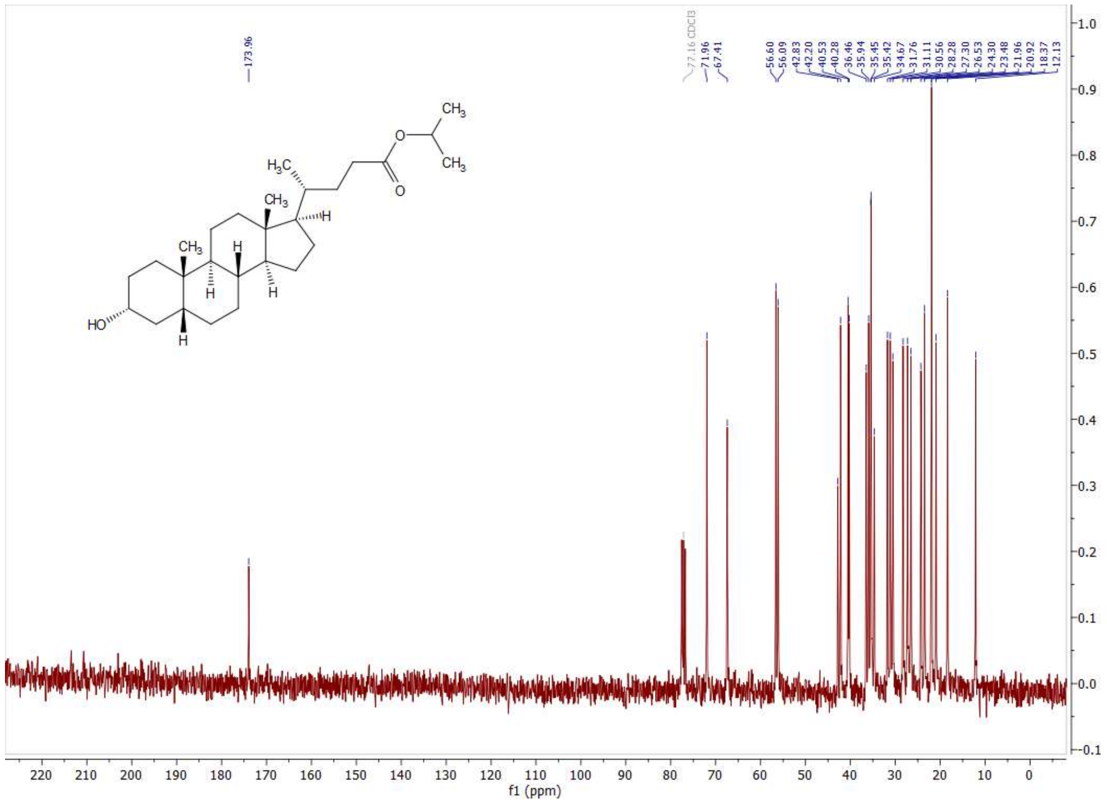

n-Butyl benzoate (1d)

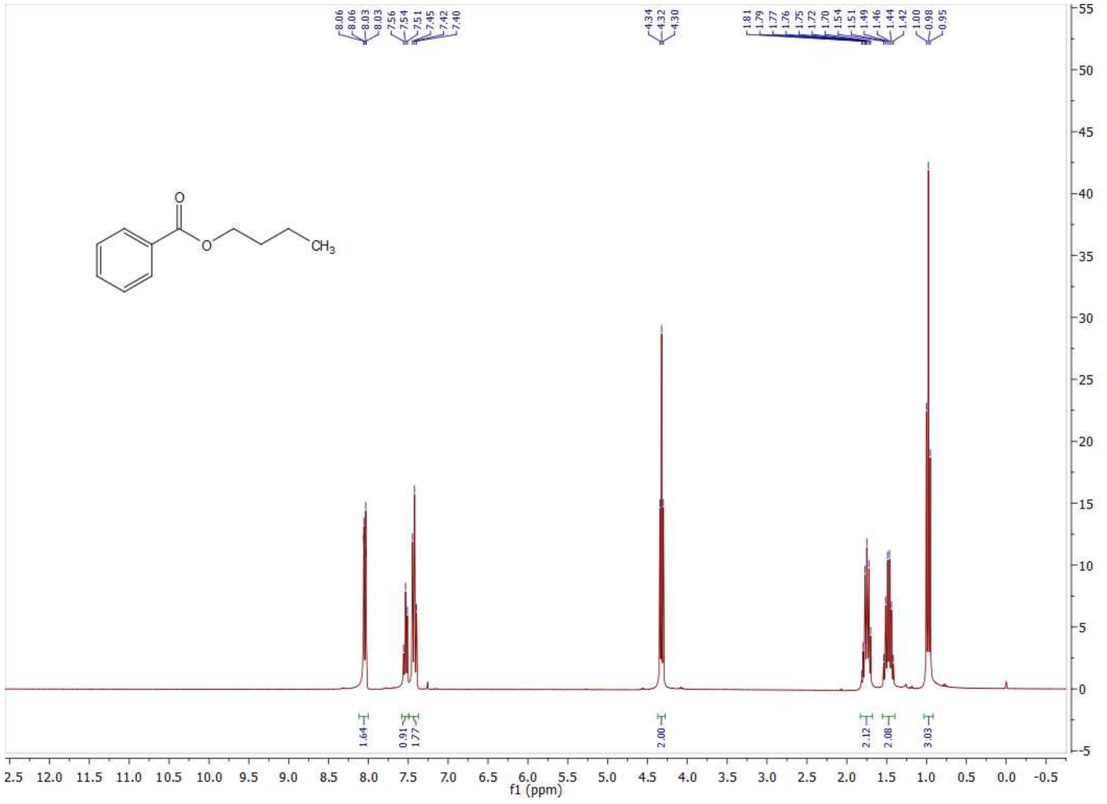

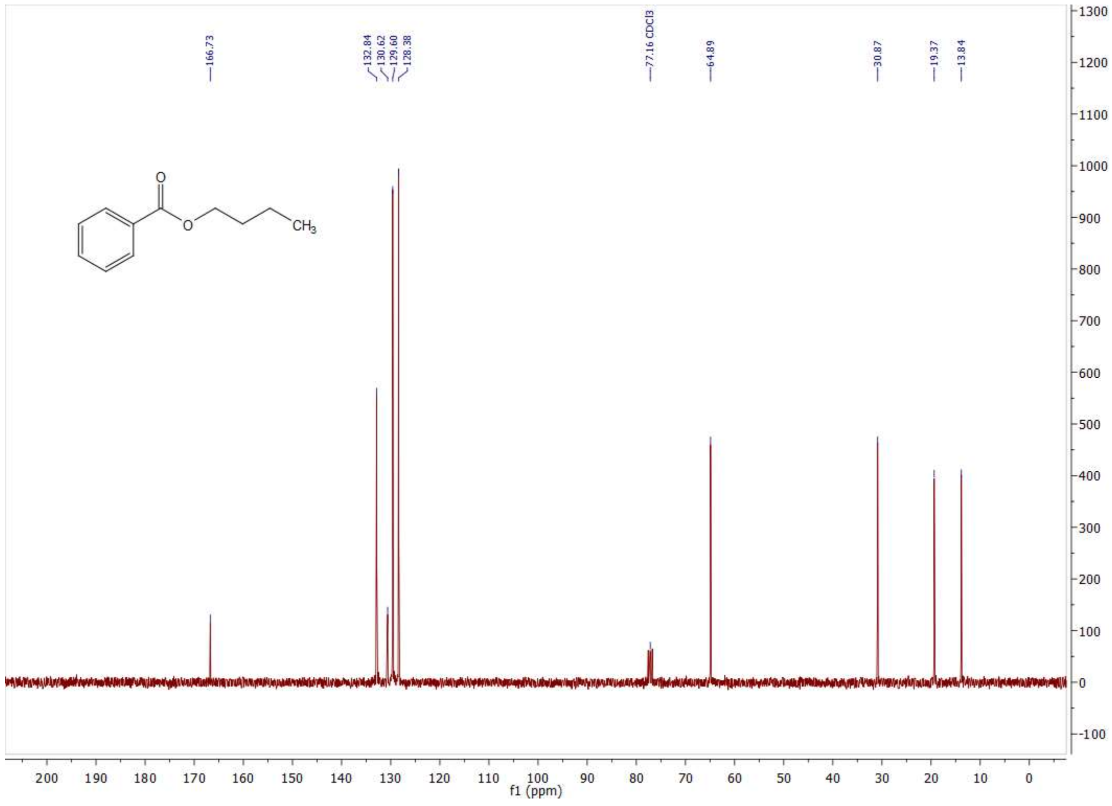

n-Butyl octanoate (2d)

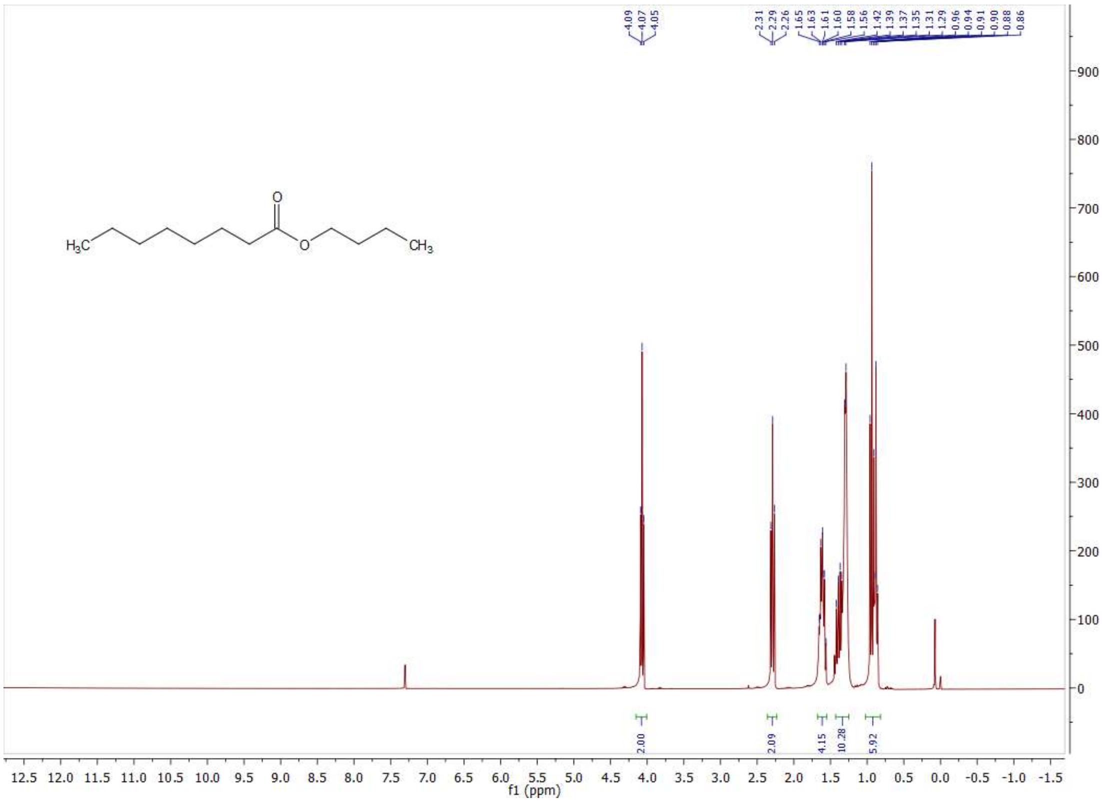

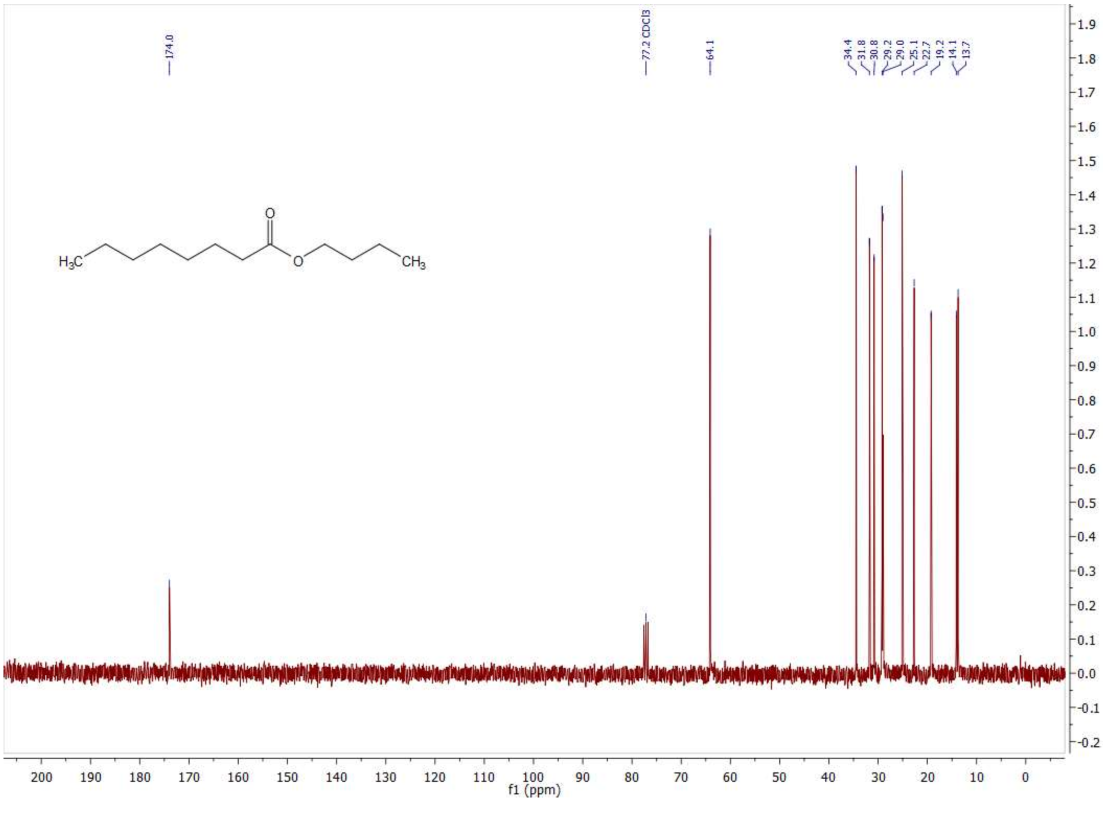

Cholic acid *n*-butyl ester (30d)

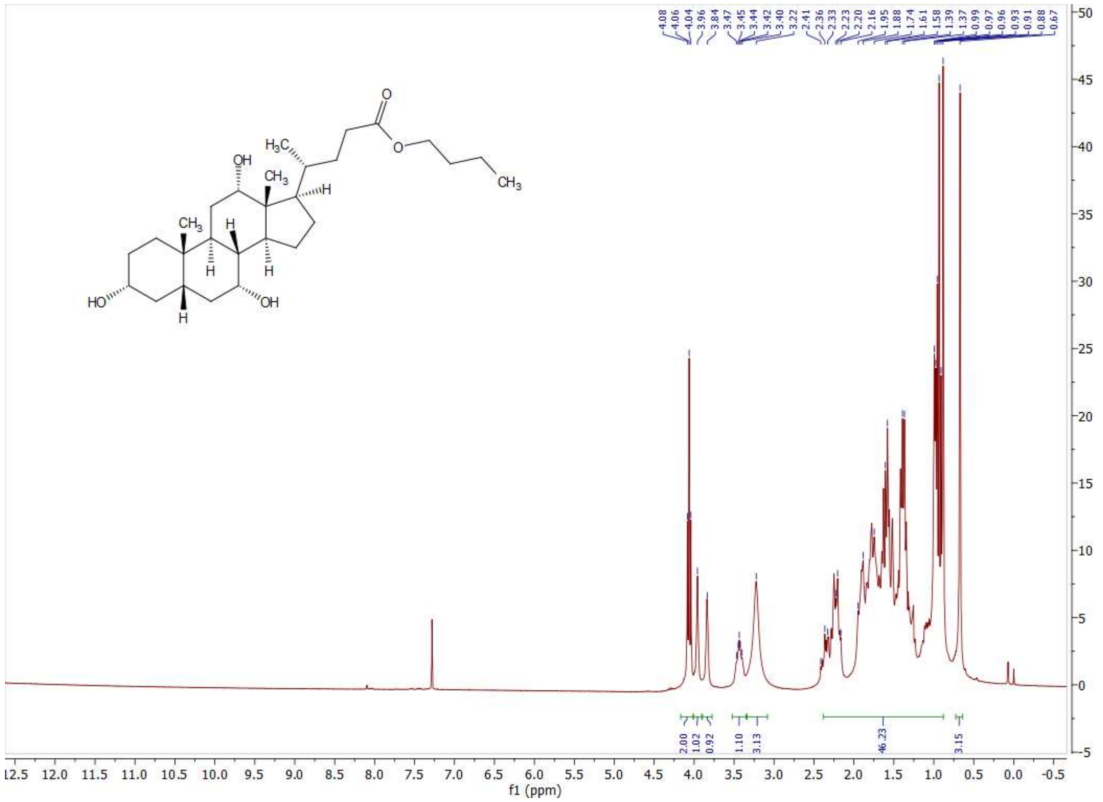

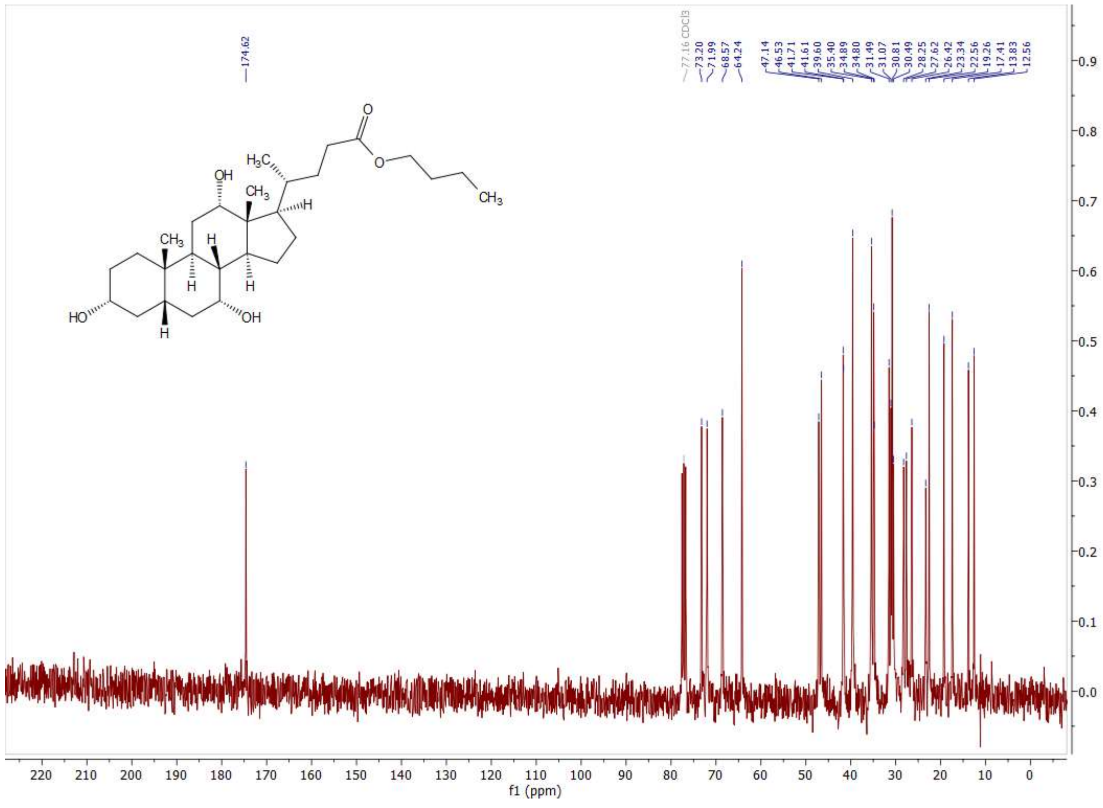

Litocholic acid *n*-butyl ester (32d)

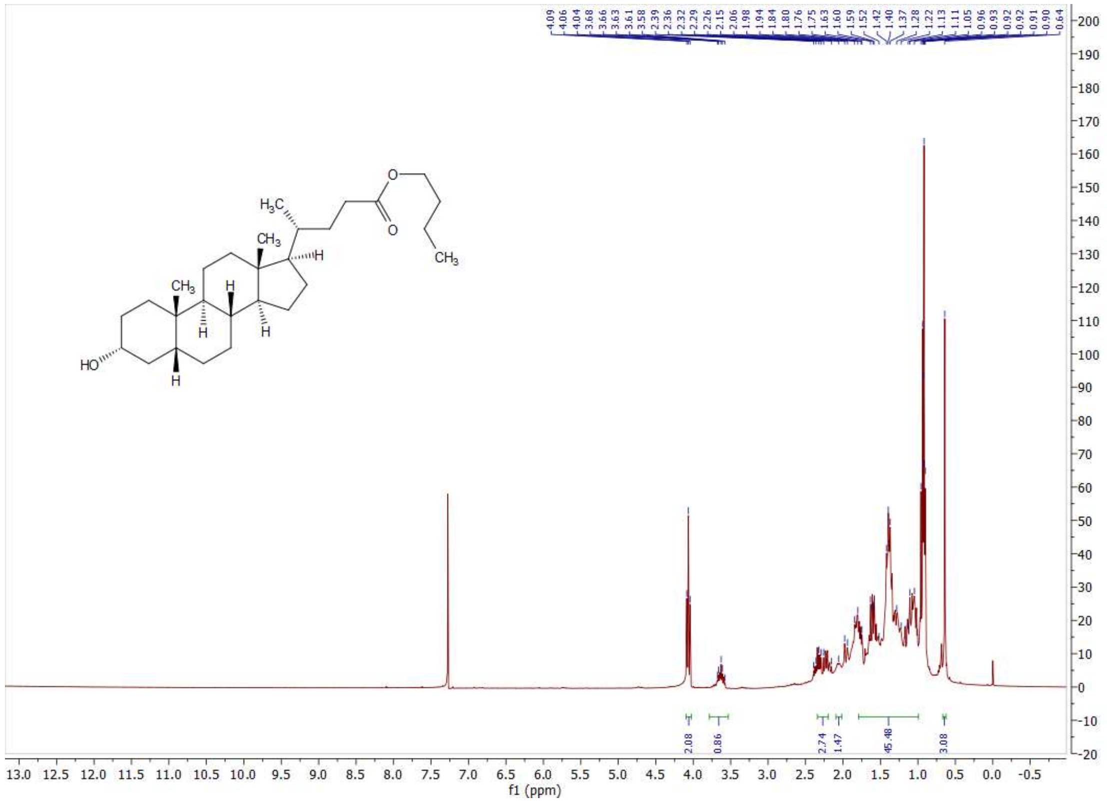

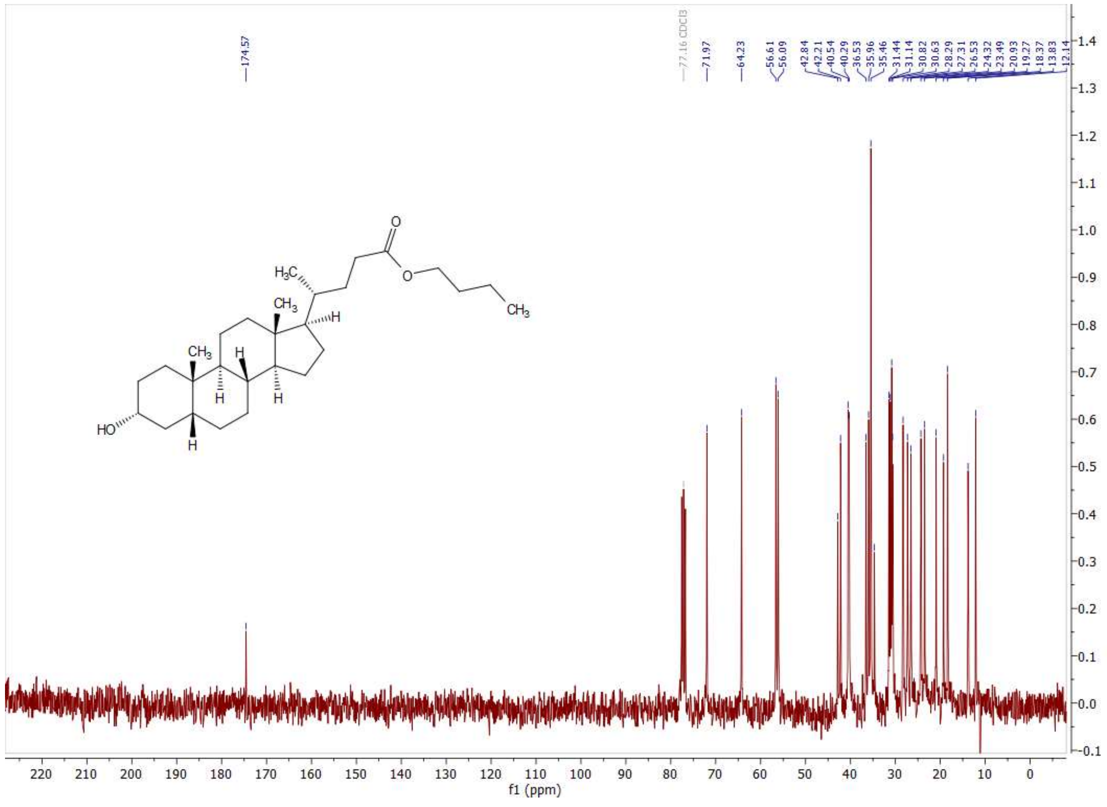

Dehydrocholic acid *n*-butyl ester (33d)

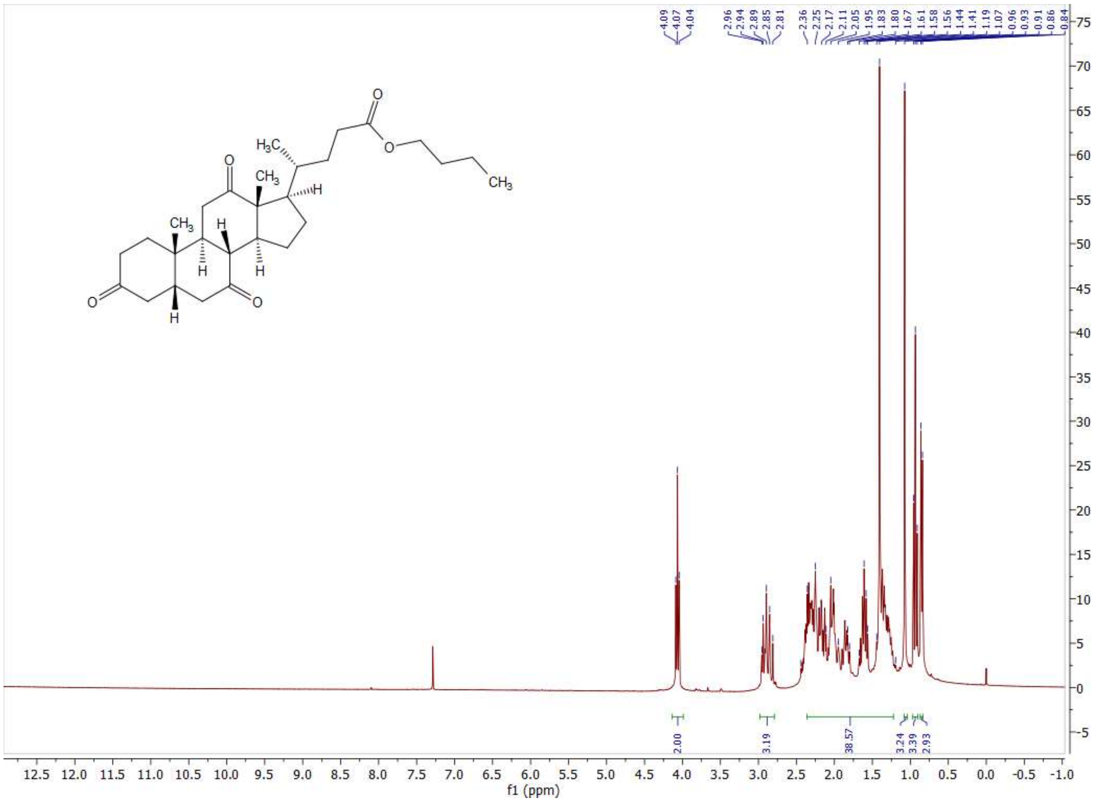

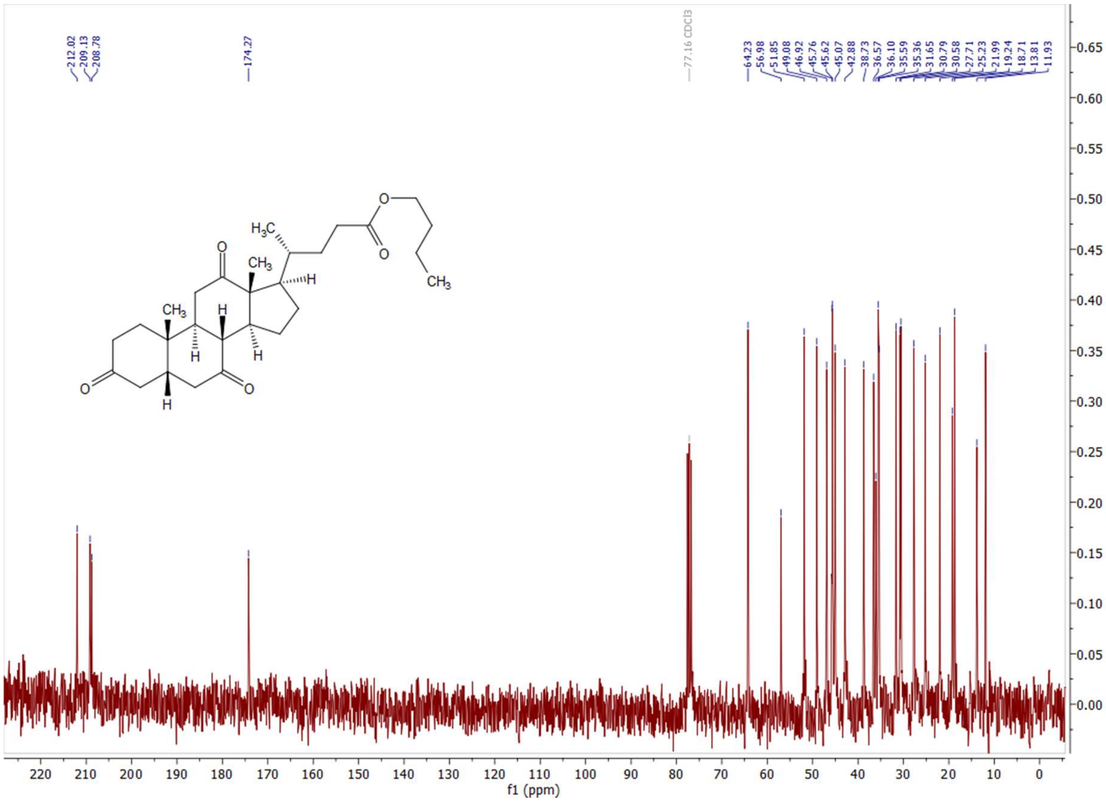

_n_-Octyl benzoate (1f)

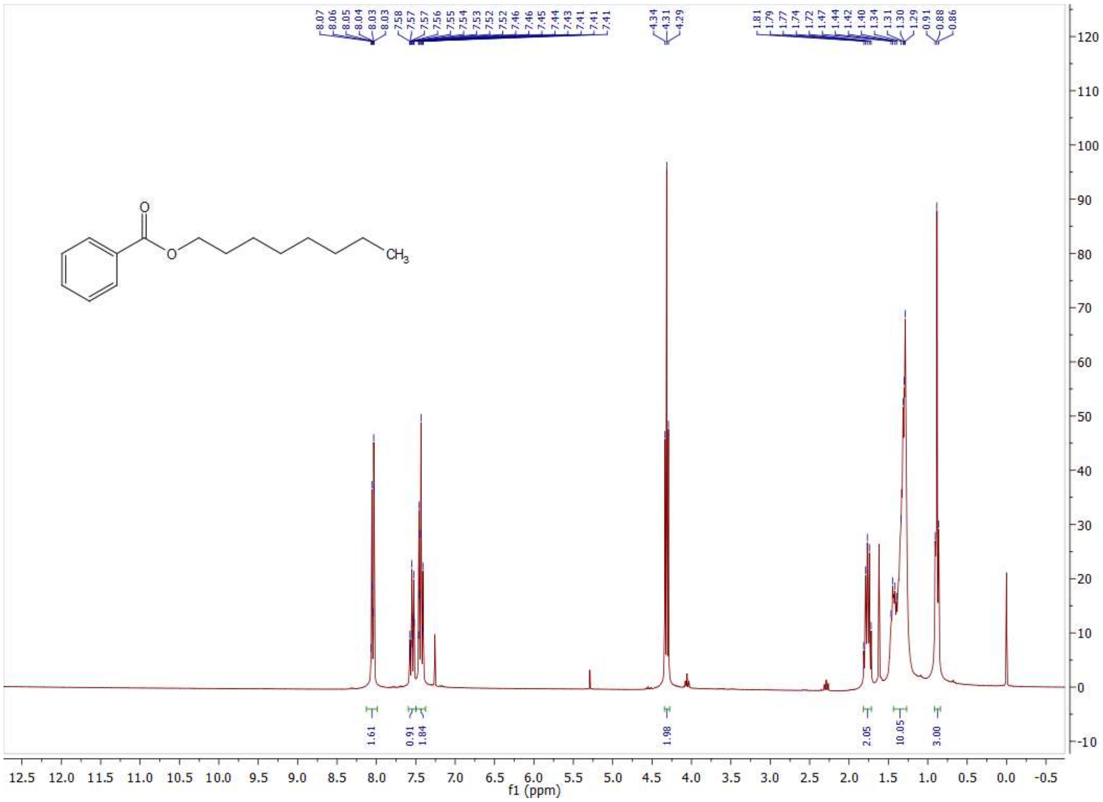

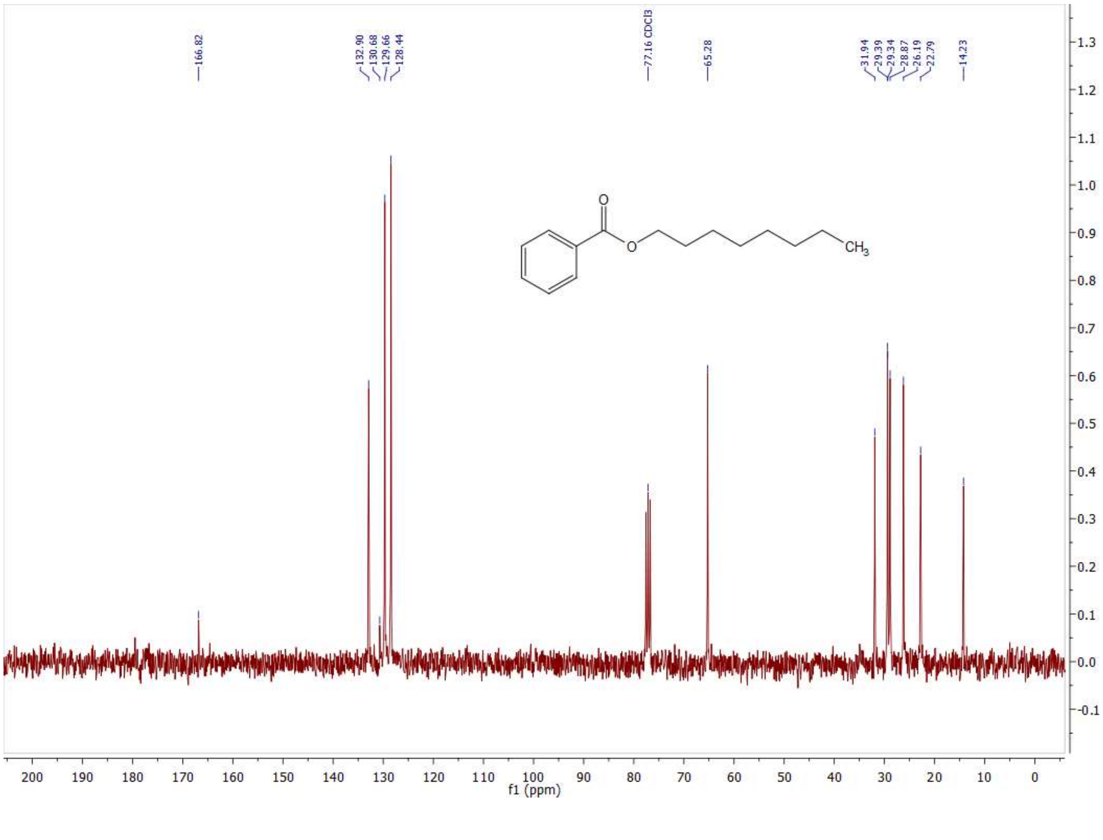

***n*-Octyl octanoate (2f)**

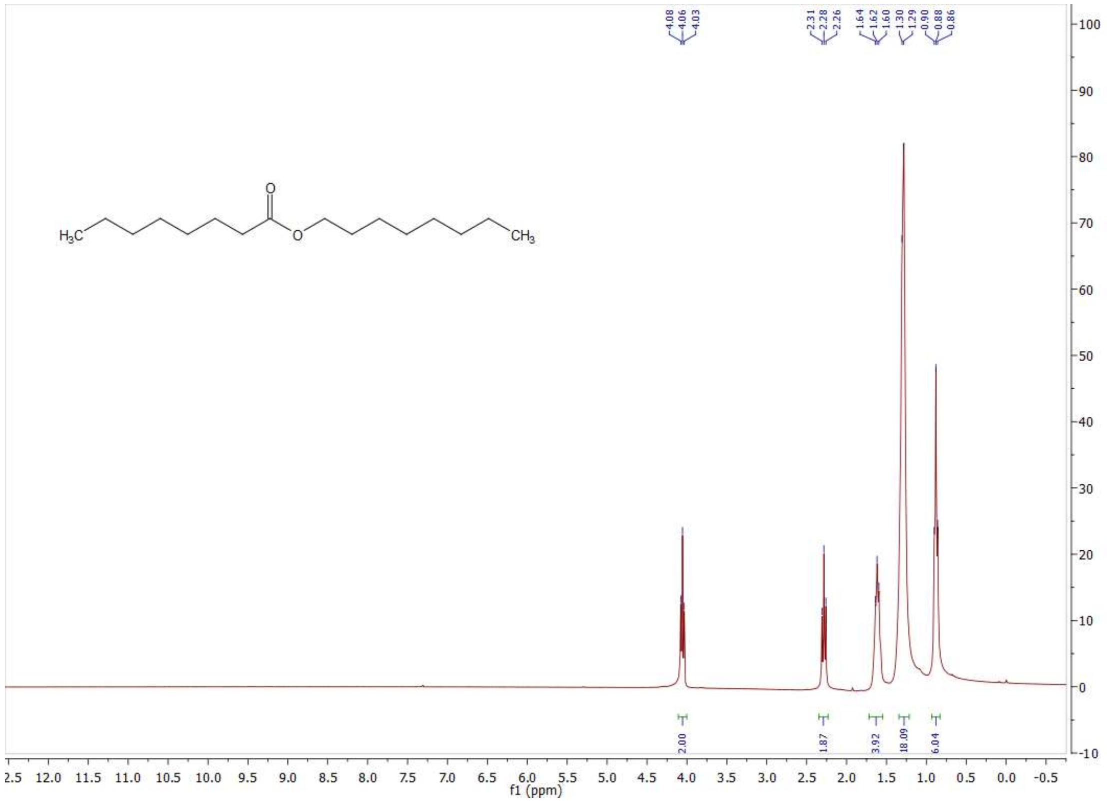

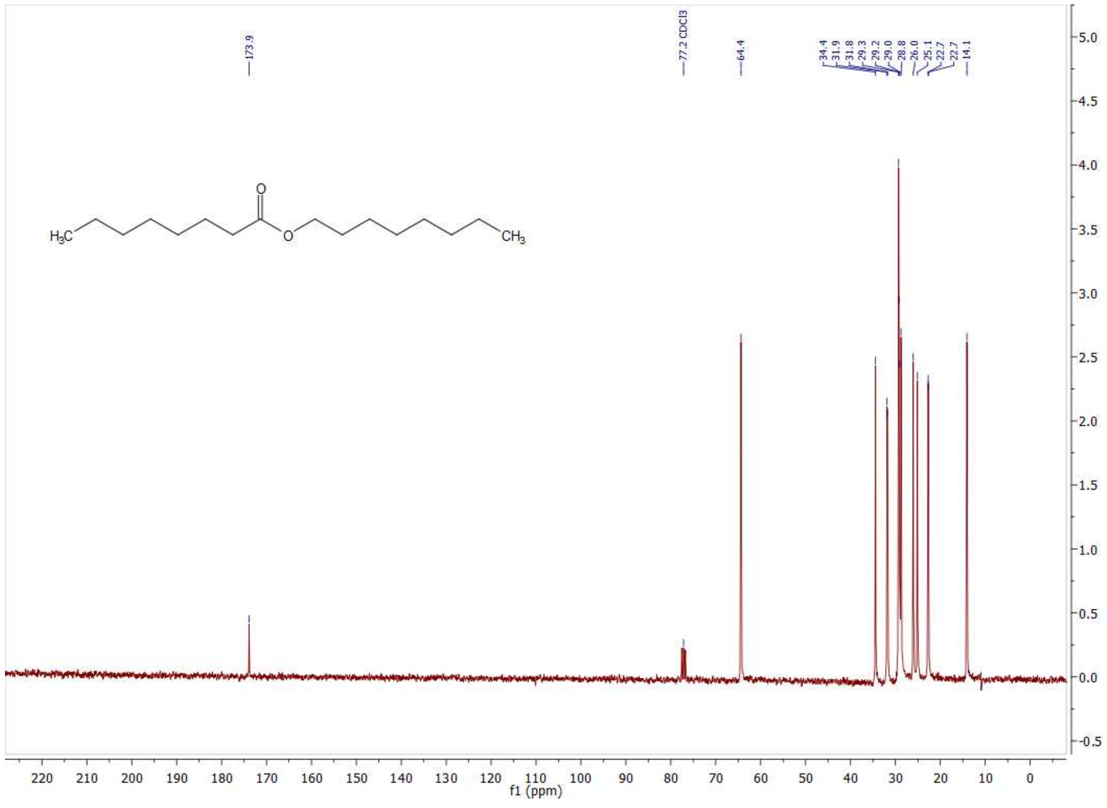

Cyclopentyl benzoate (1g)

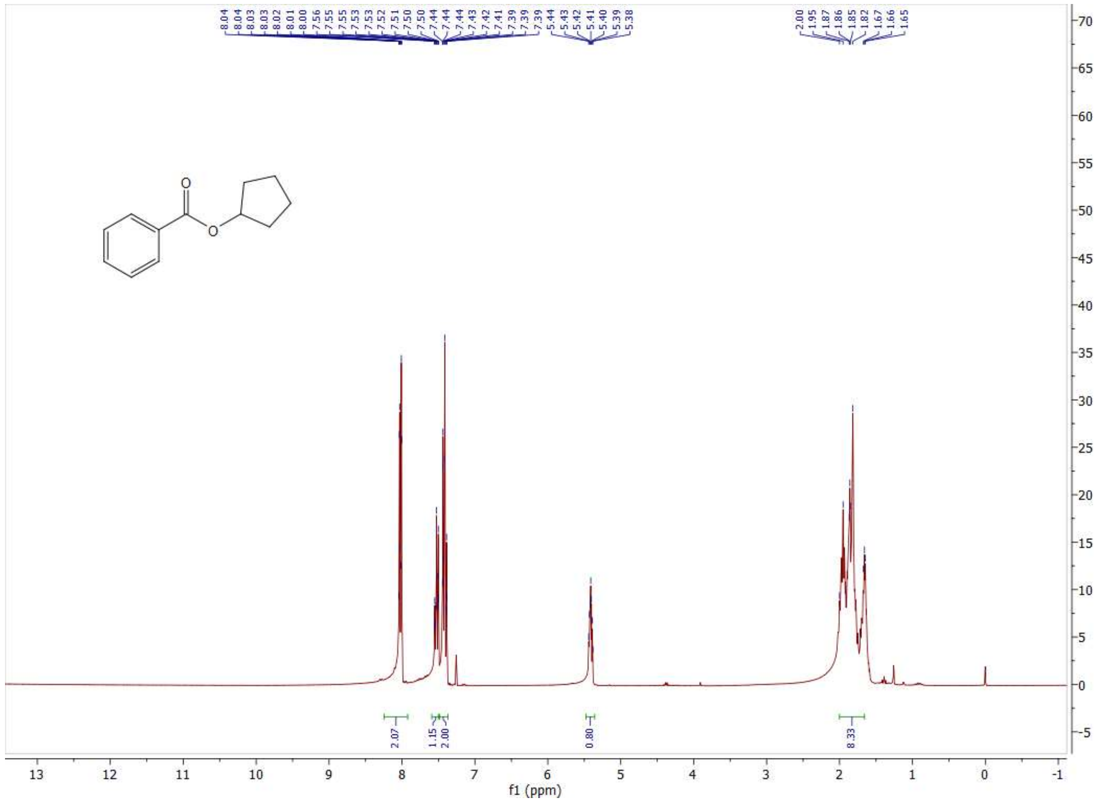

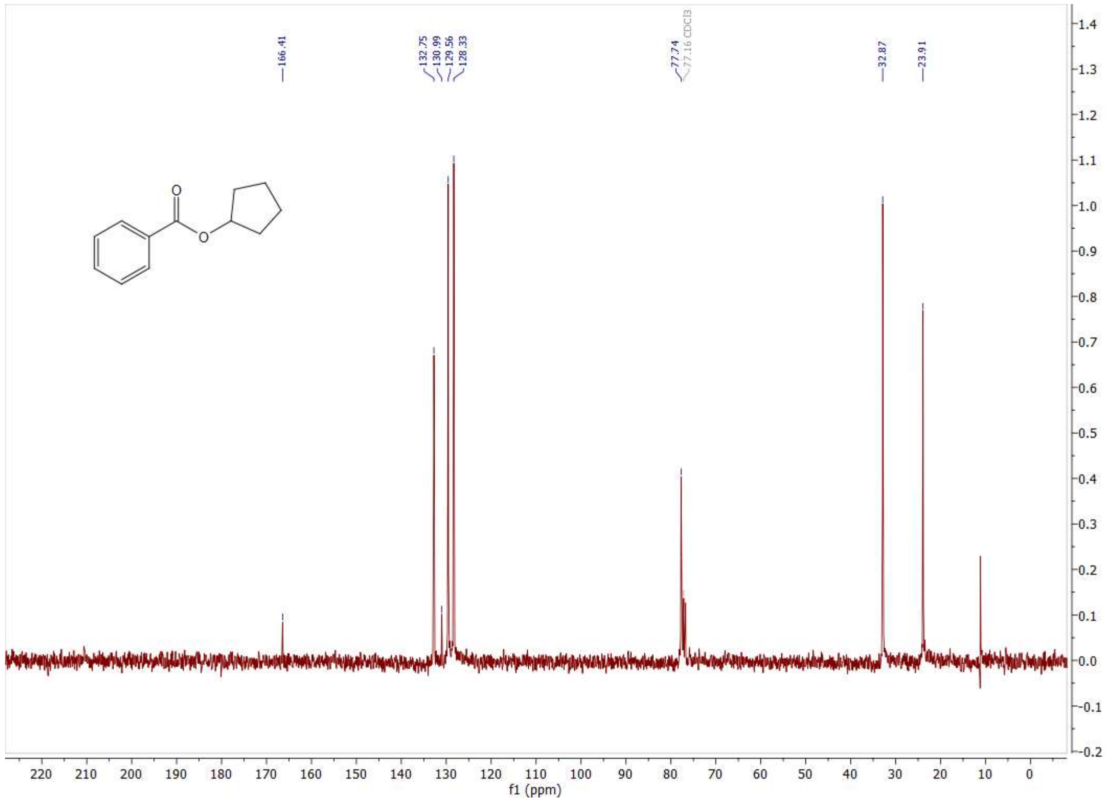

Cyclopentyl octanoate (2g)

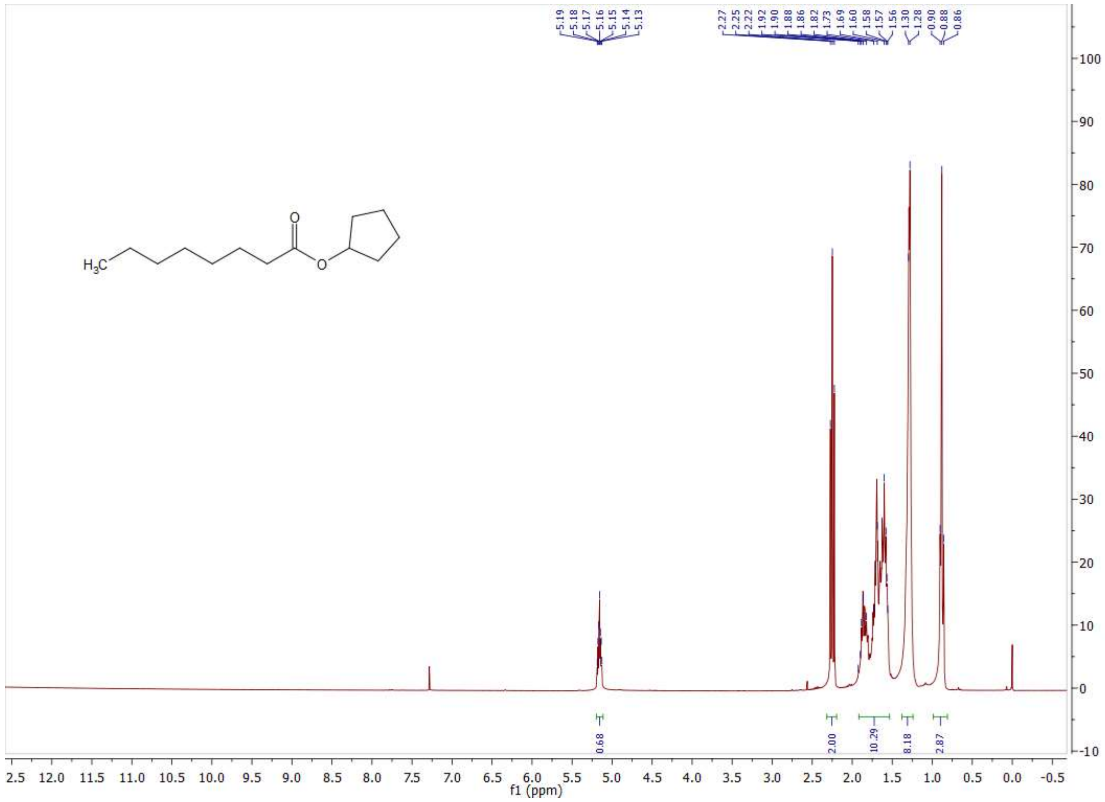

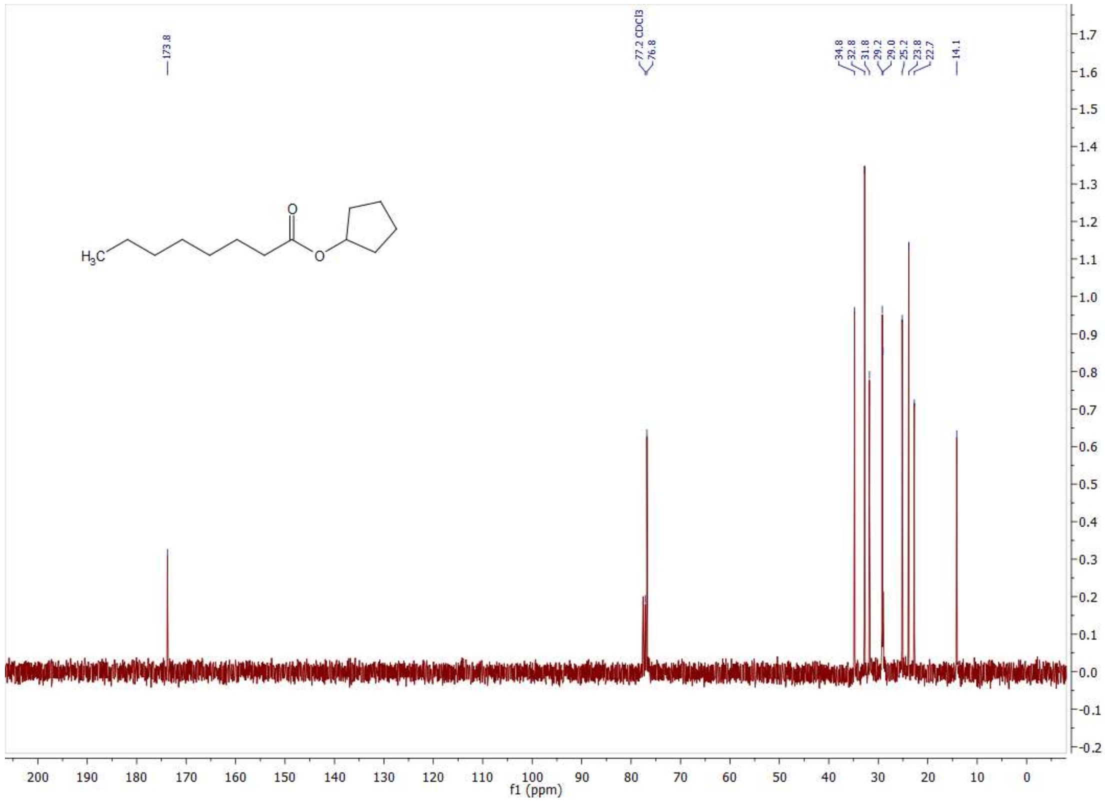

Adamantan-1-ylmethyl benzoate (1j)

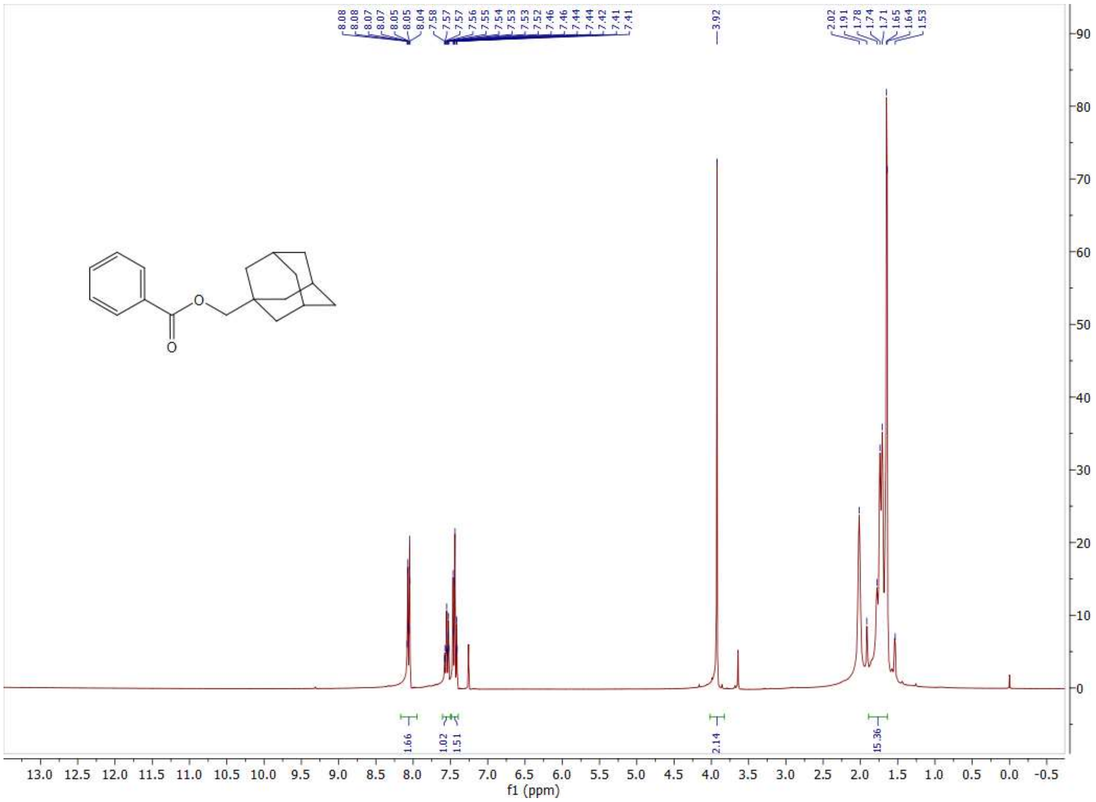

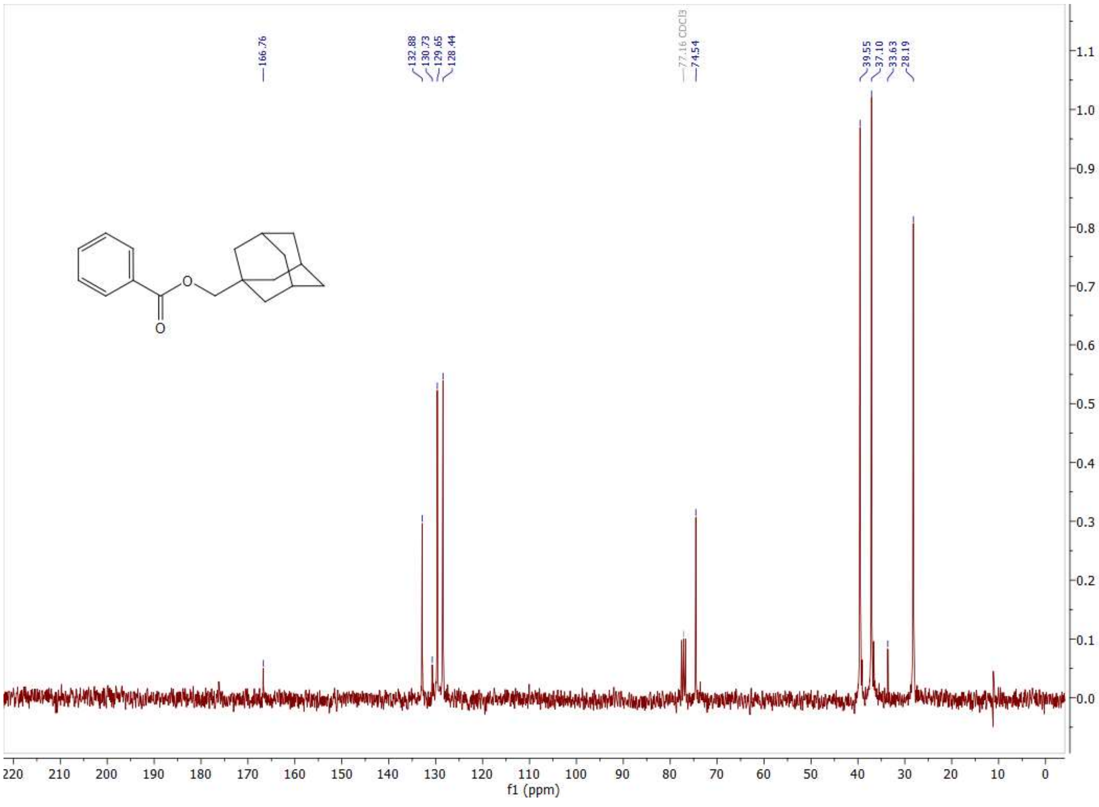

Adamantan-1-ylmethyl octanoate (2j)

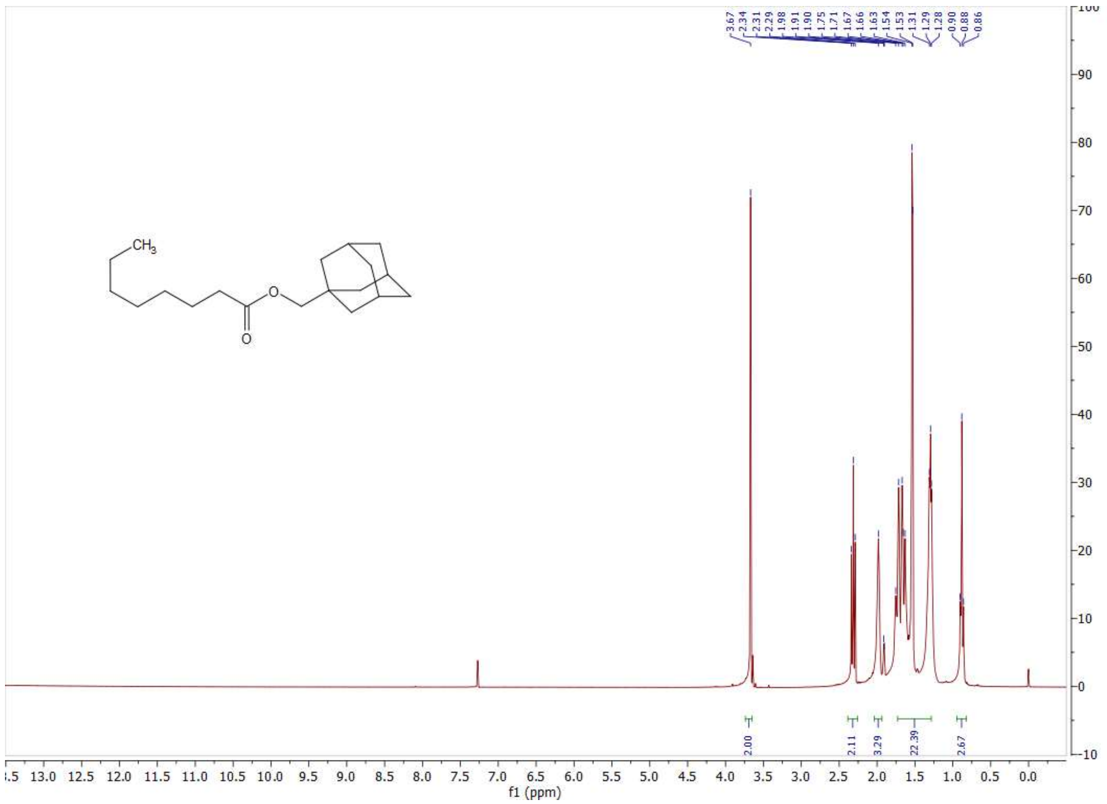

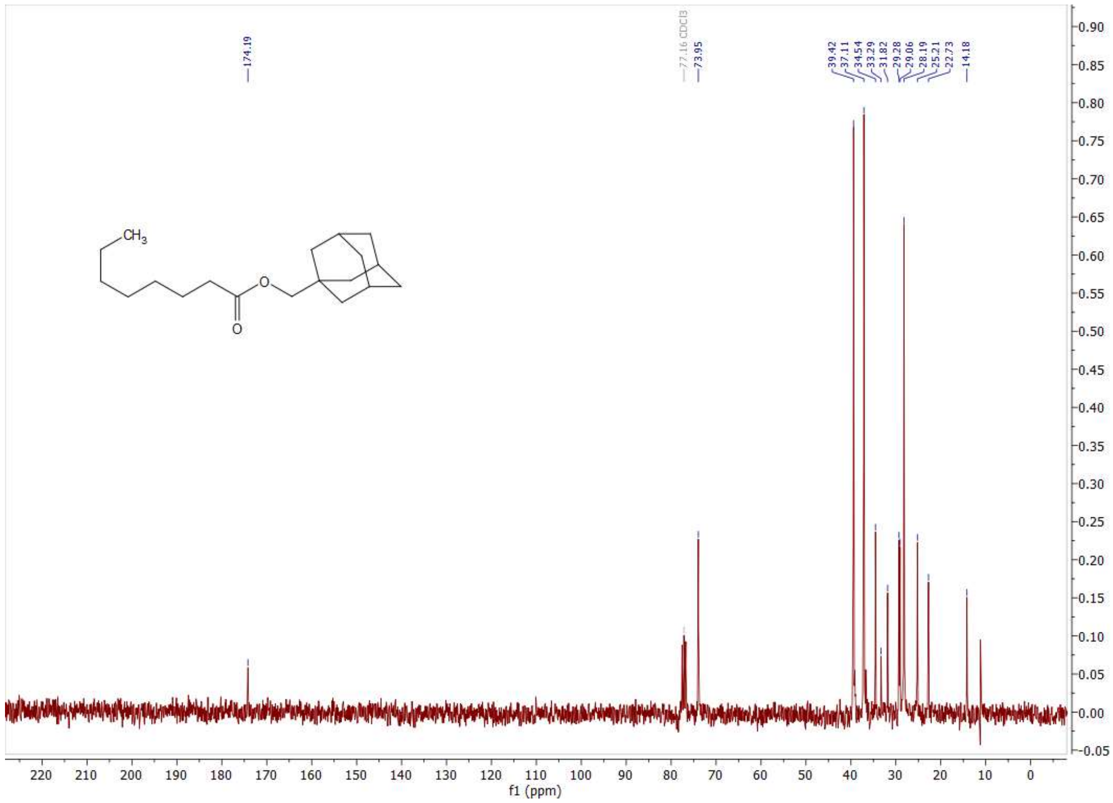

3β-O-Acetyl-cholesterol (34k)

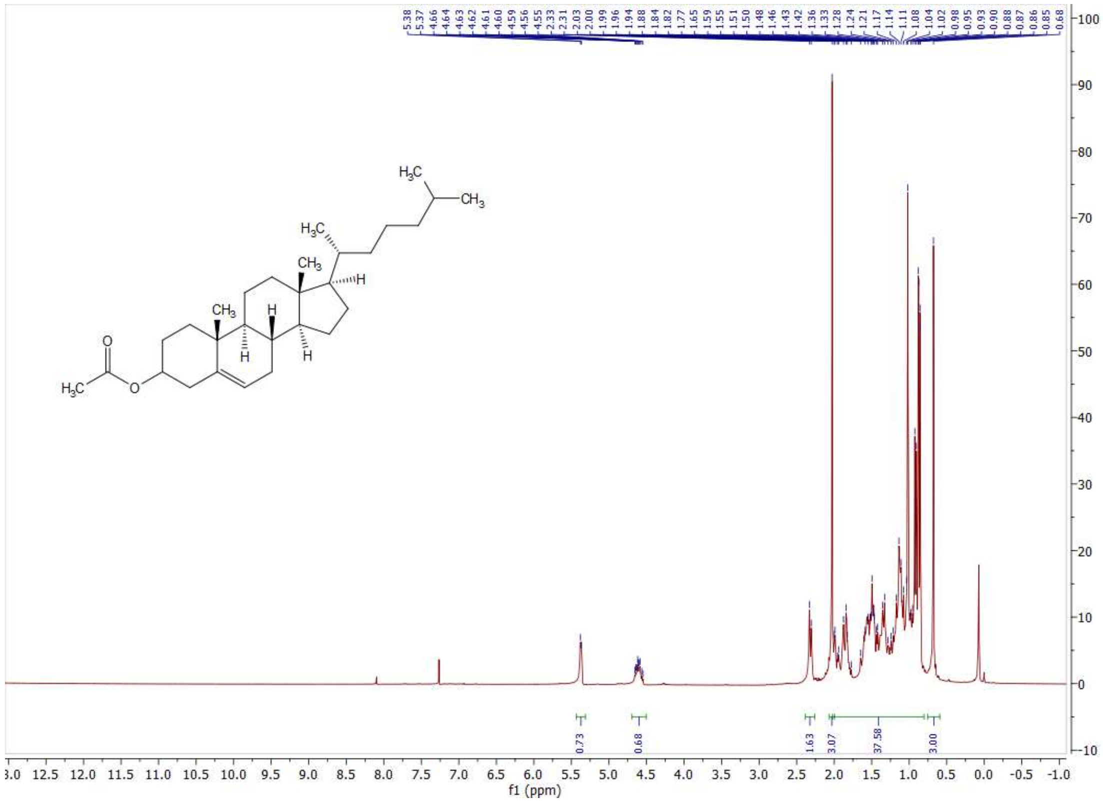

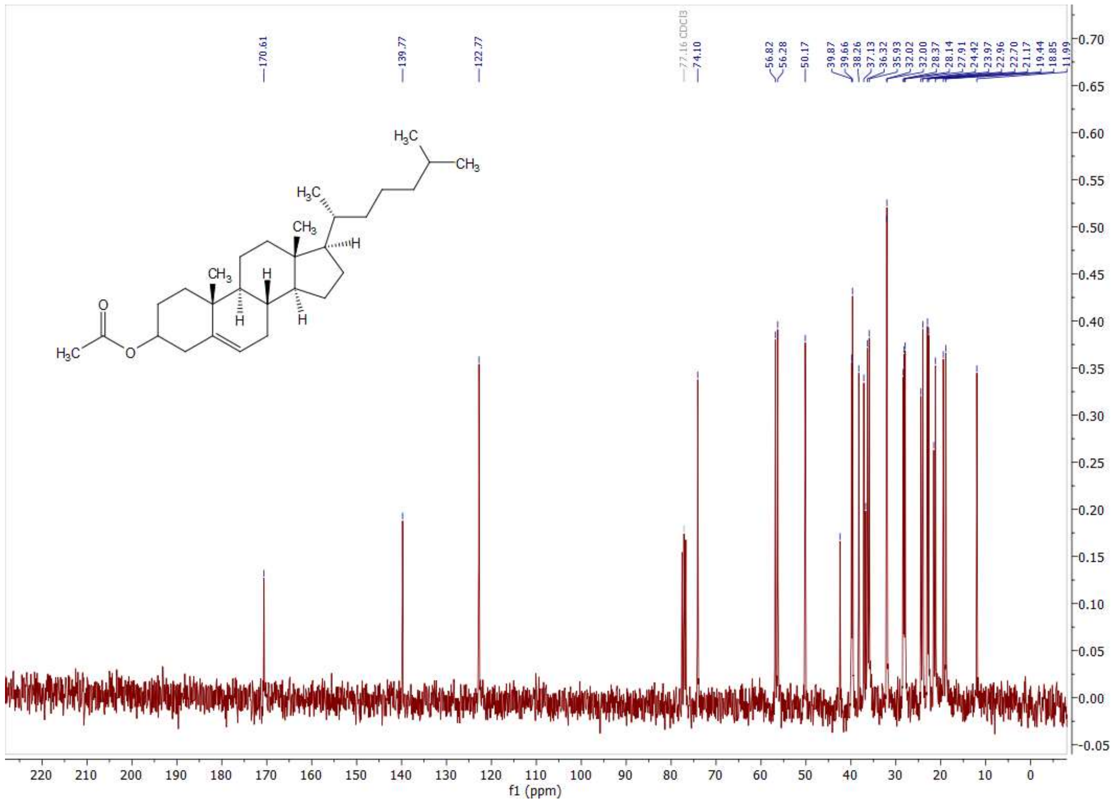

3β-Acetyloxy-5α-androstan-17-on (34l)

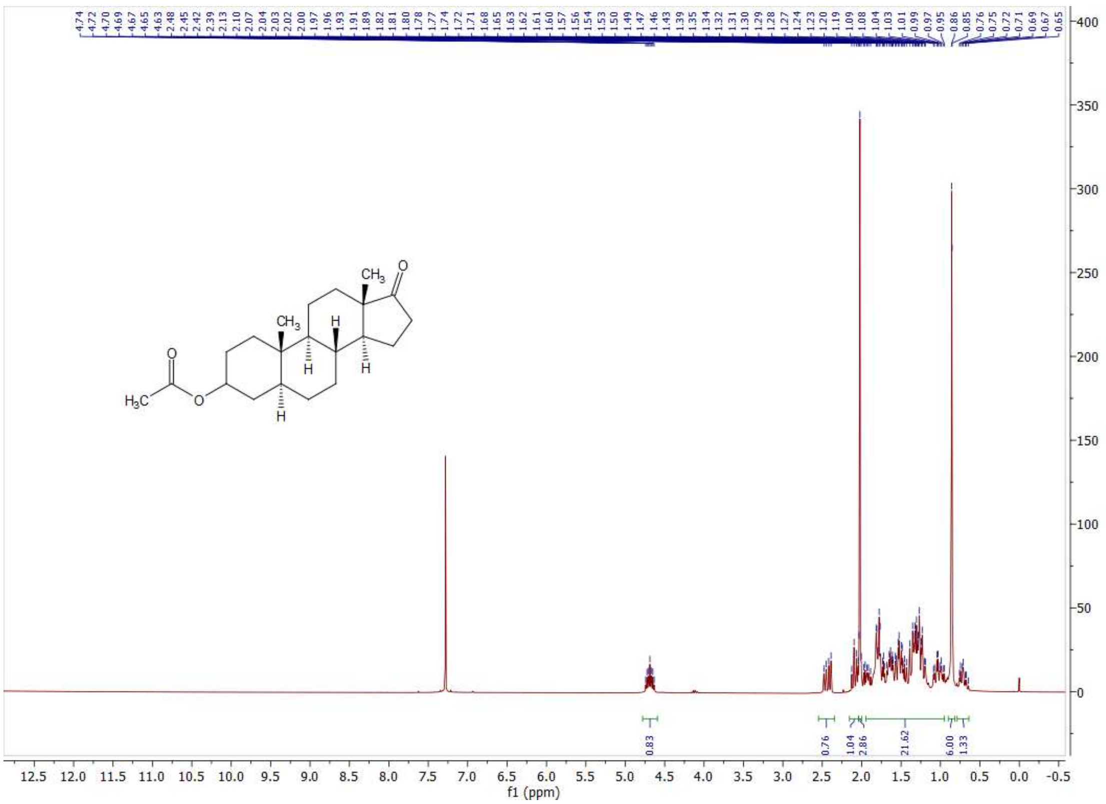

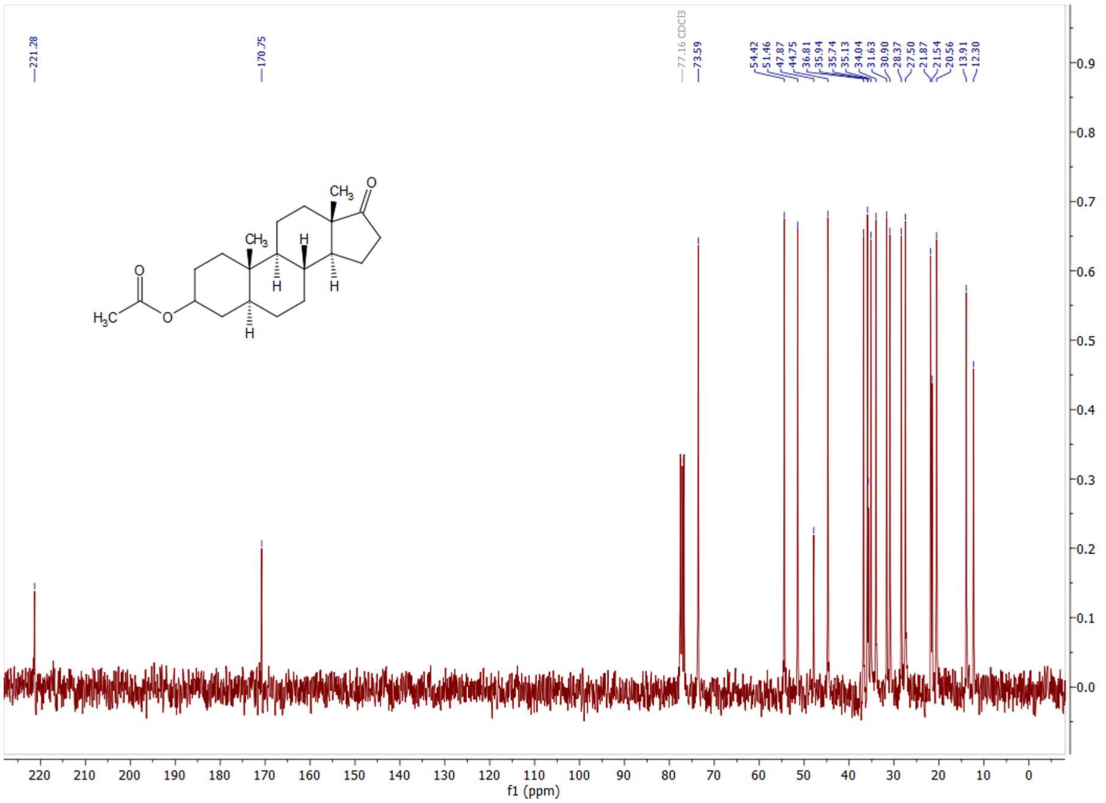

(*E*)-2-ethylhex-2-enal (35m)

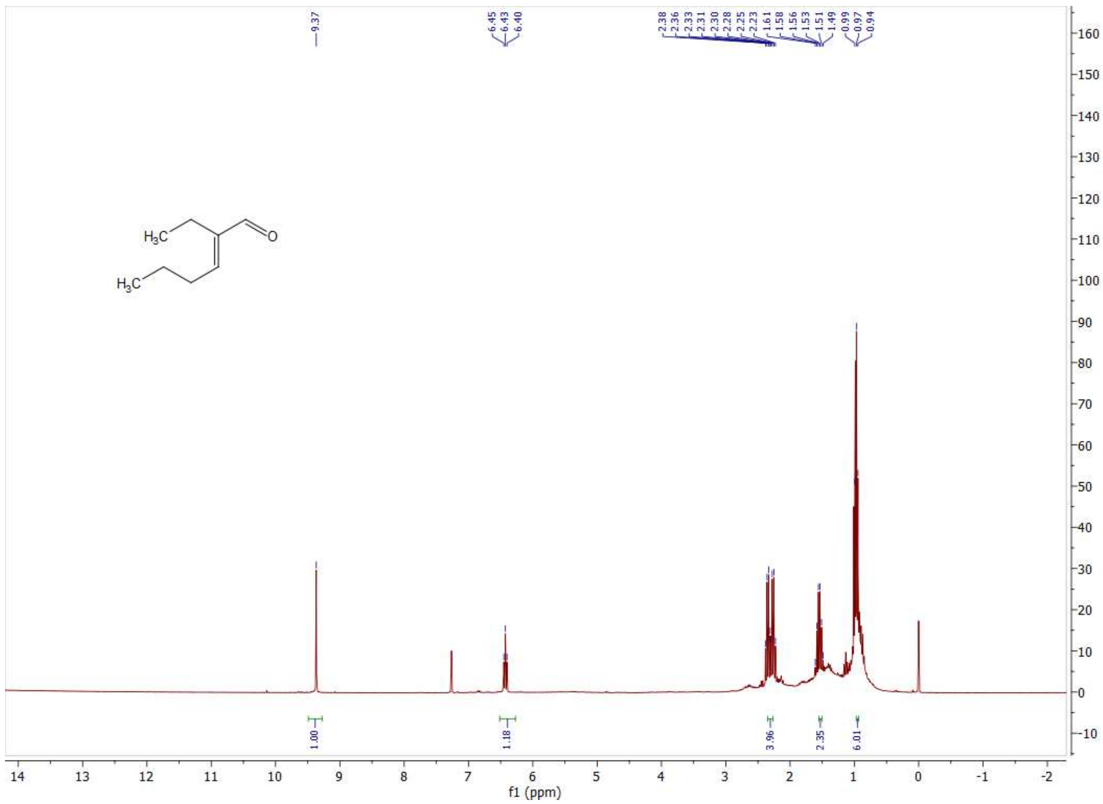

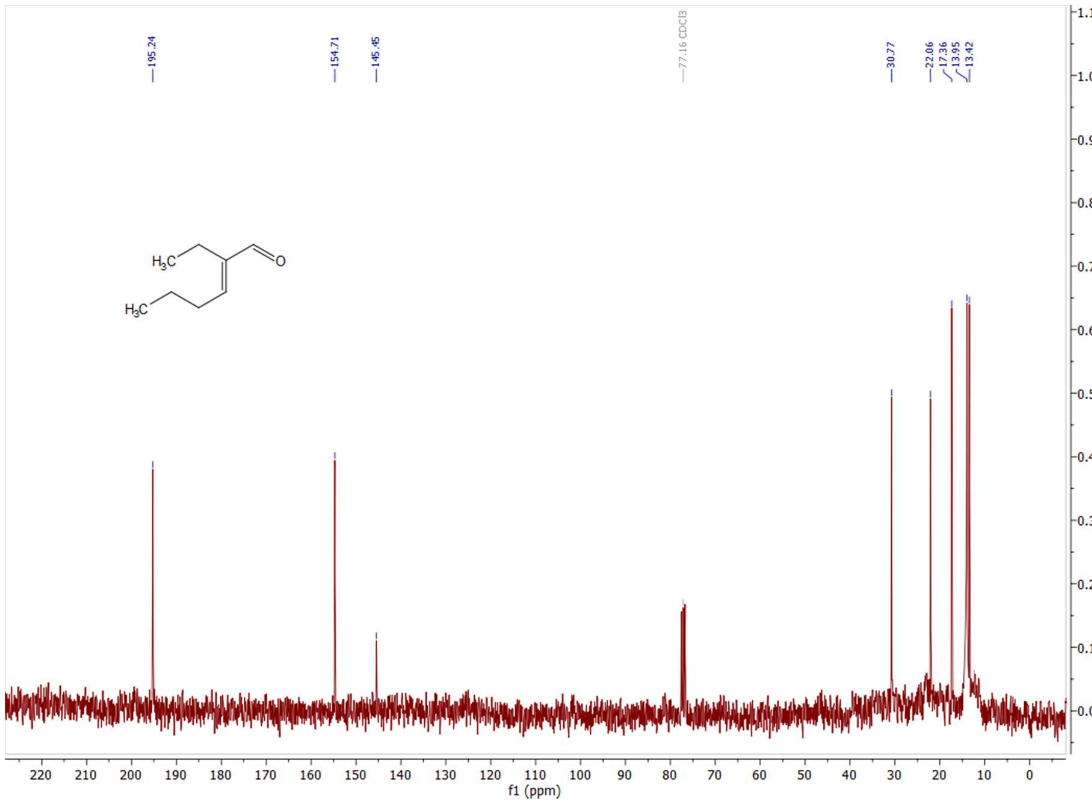

(*E*)-2-butyloct-2-enal (36m)

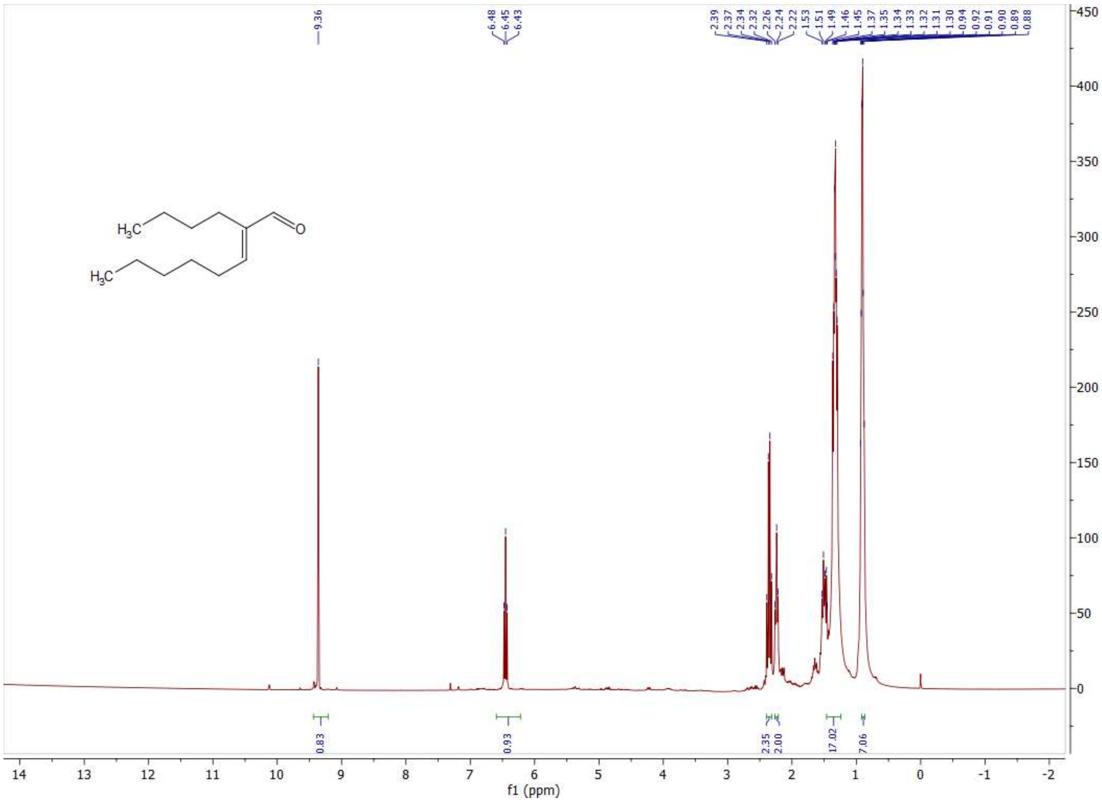

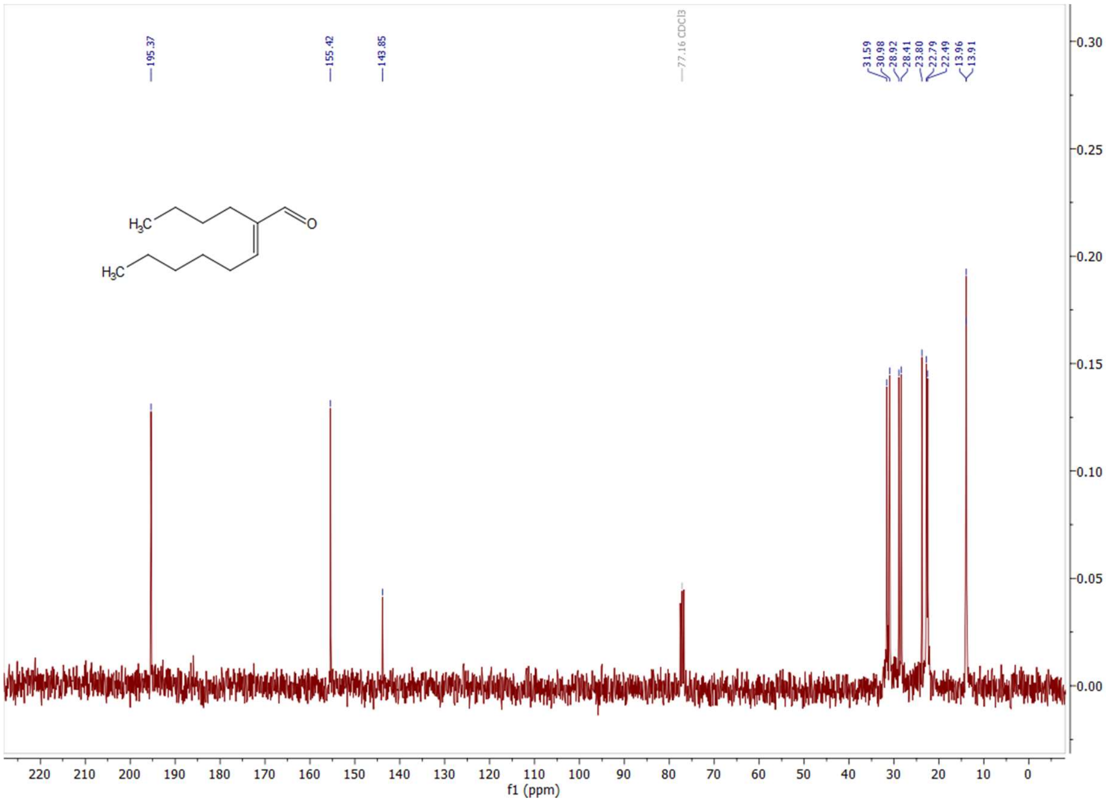

(*E*)-2-pentylnon-2-enal (37m)

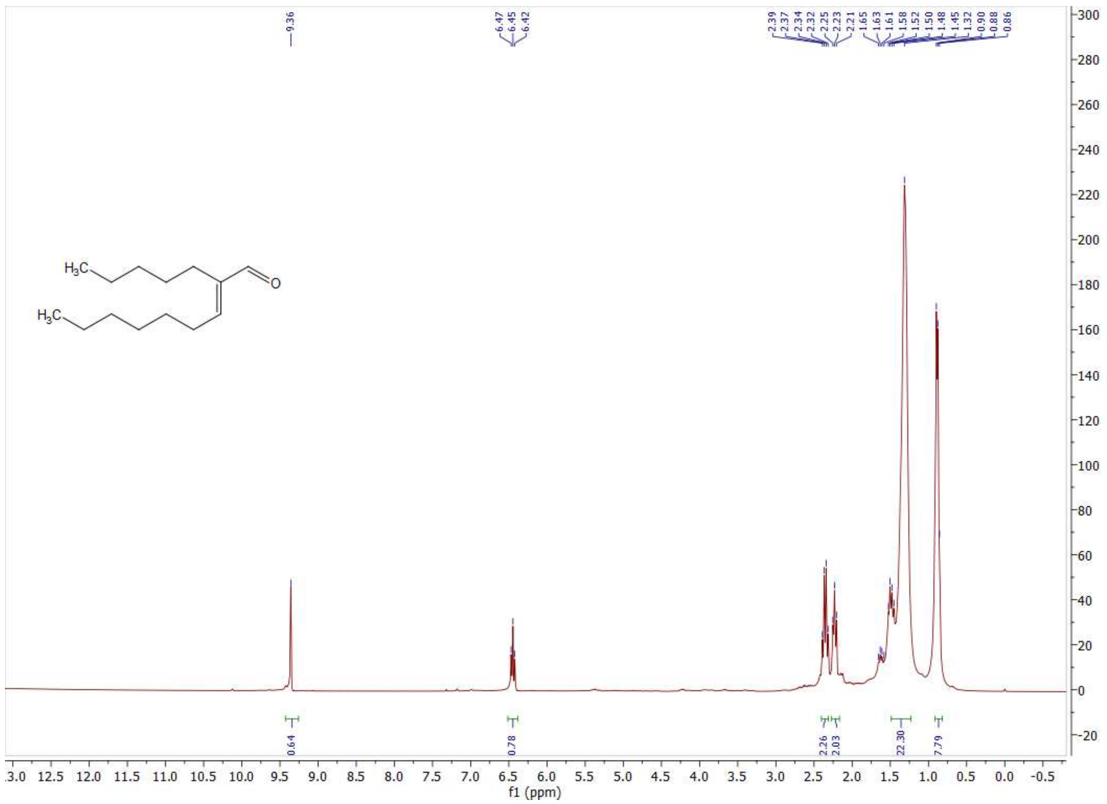

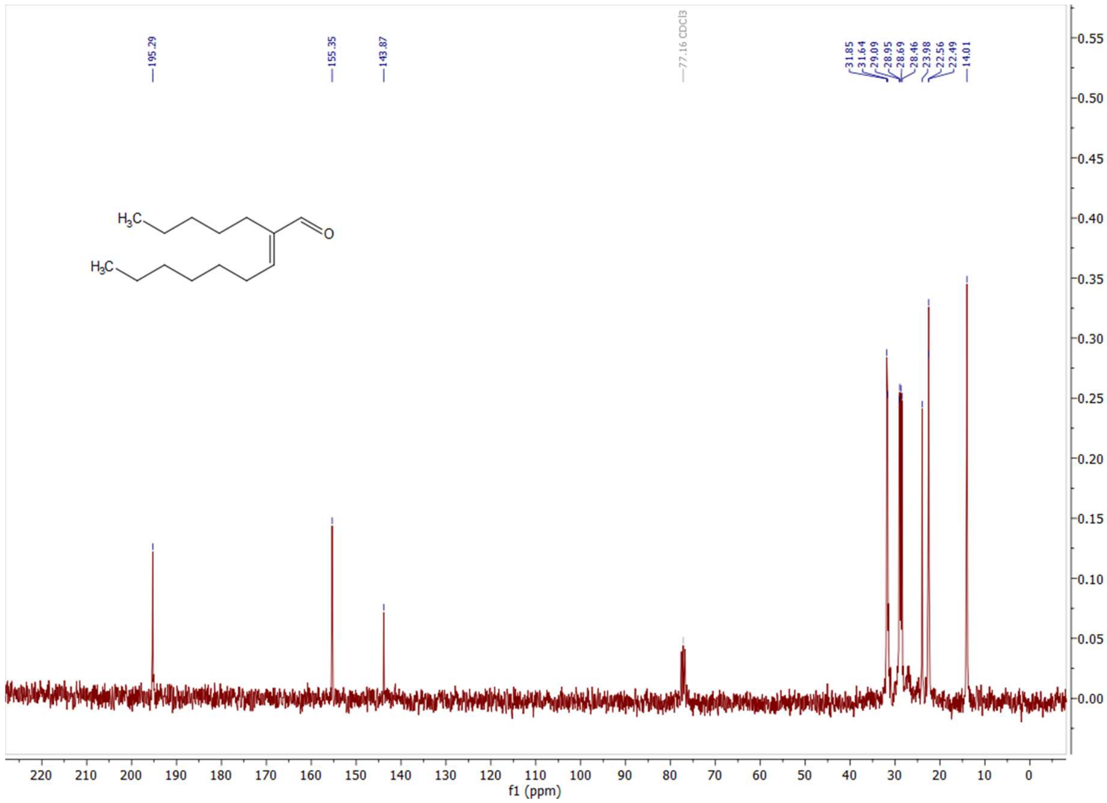

(*E*)-2-octyldodec-2-enal (38m)

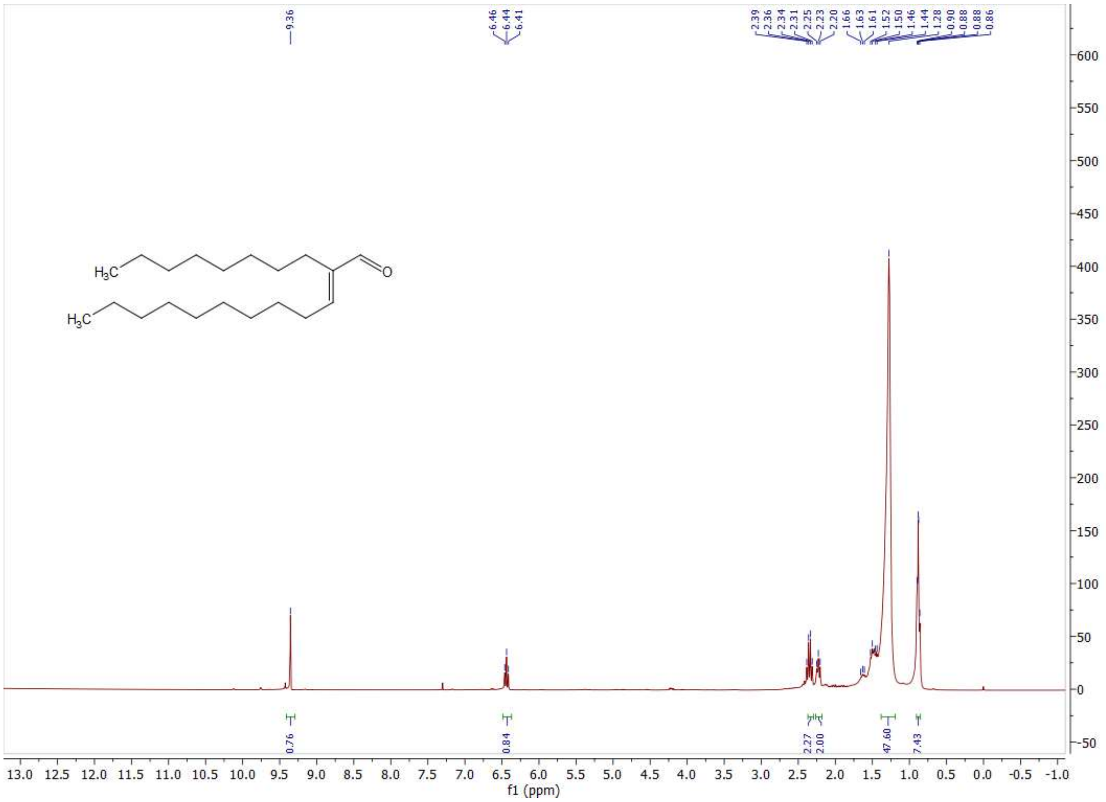

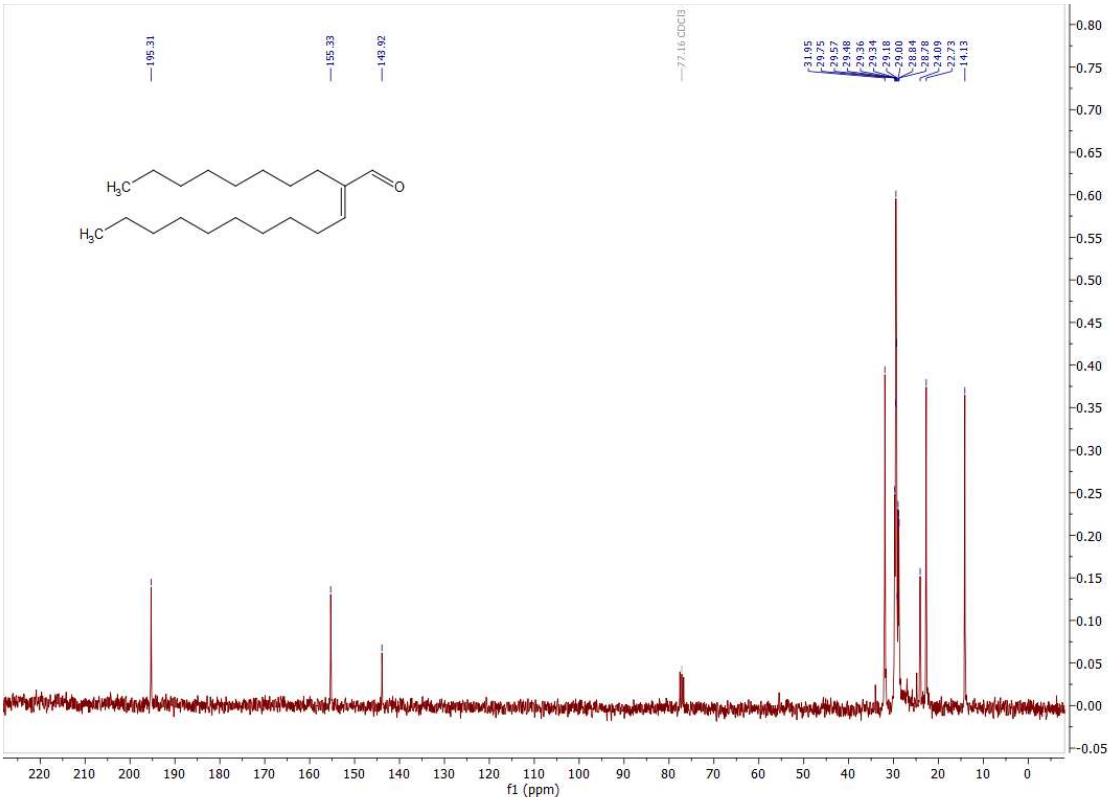

(*E*)-2-decyltetradec-2-enal (39m)

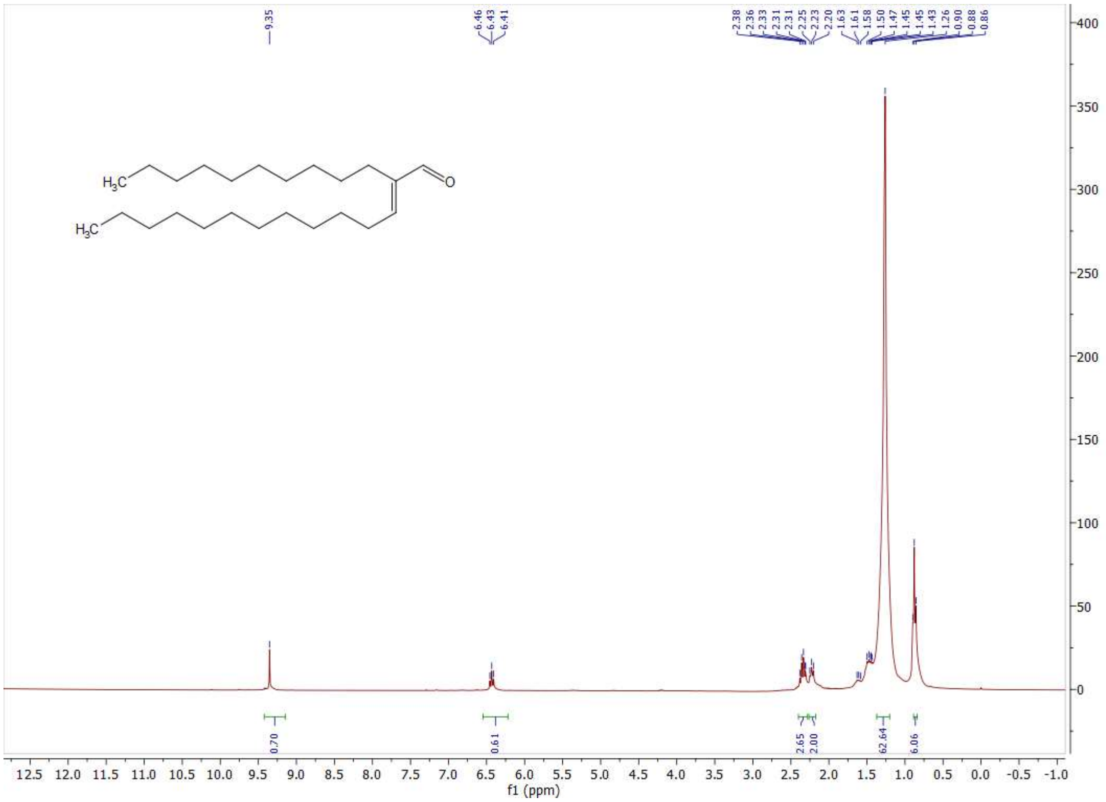

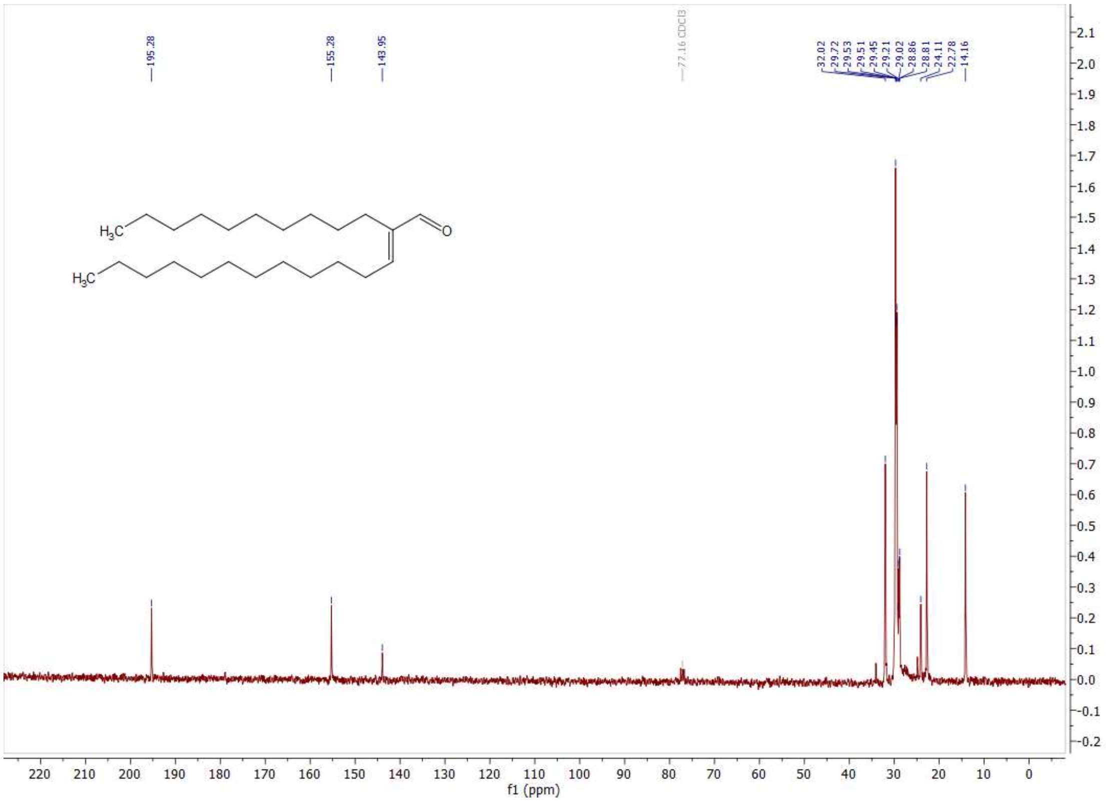

(*E*)-2-benzylideneheptanal (40m)

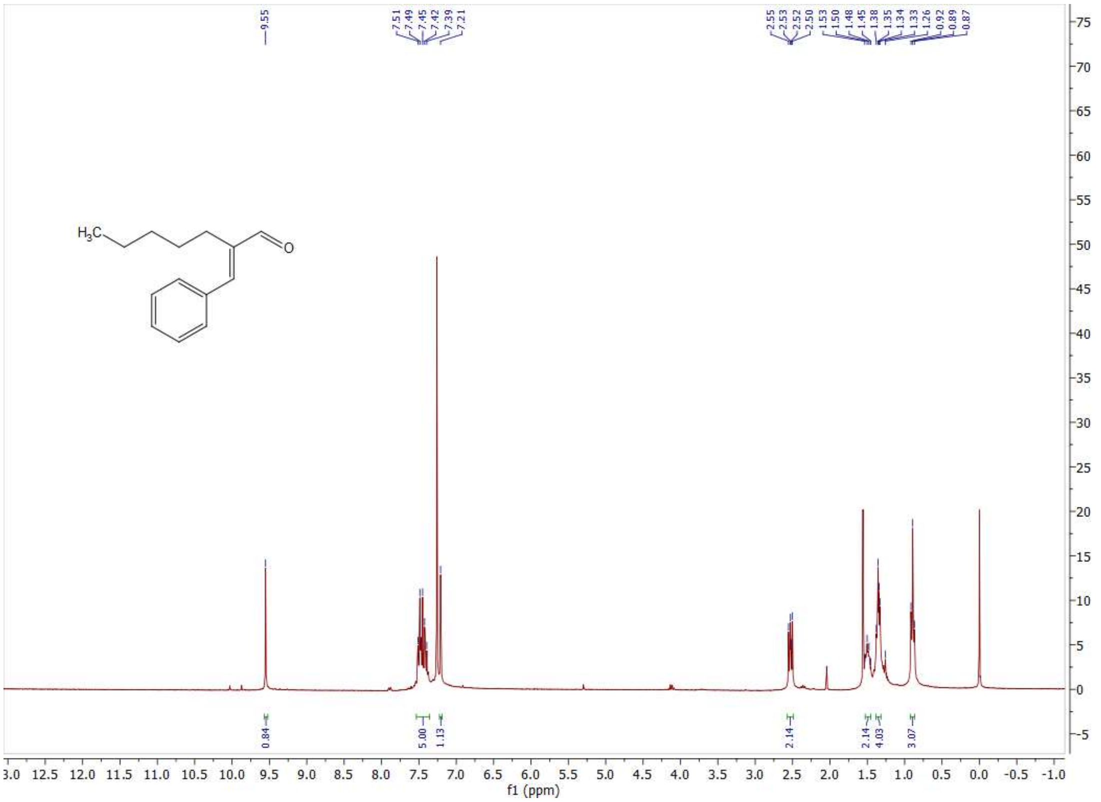

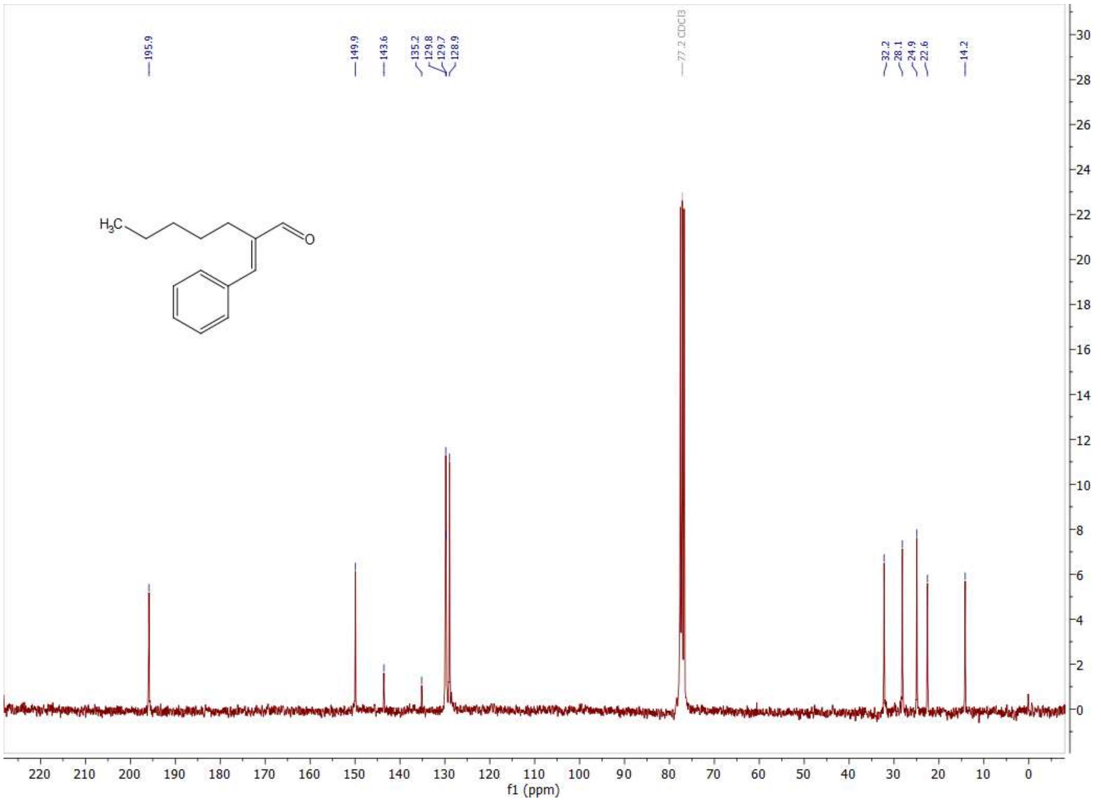

5,5-Dimethylhydantoin

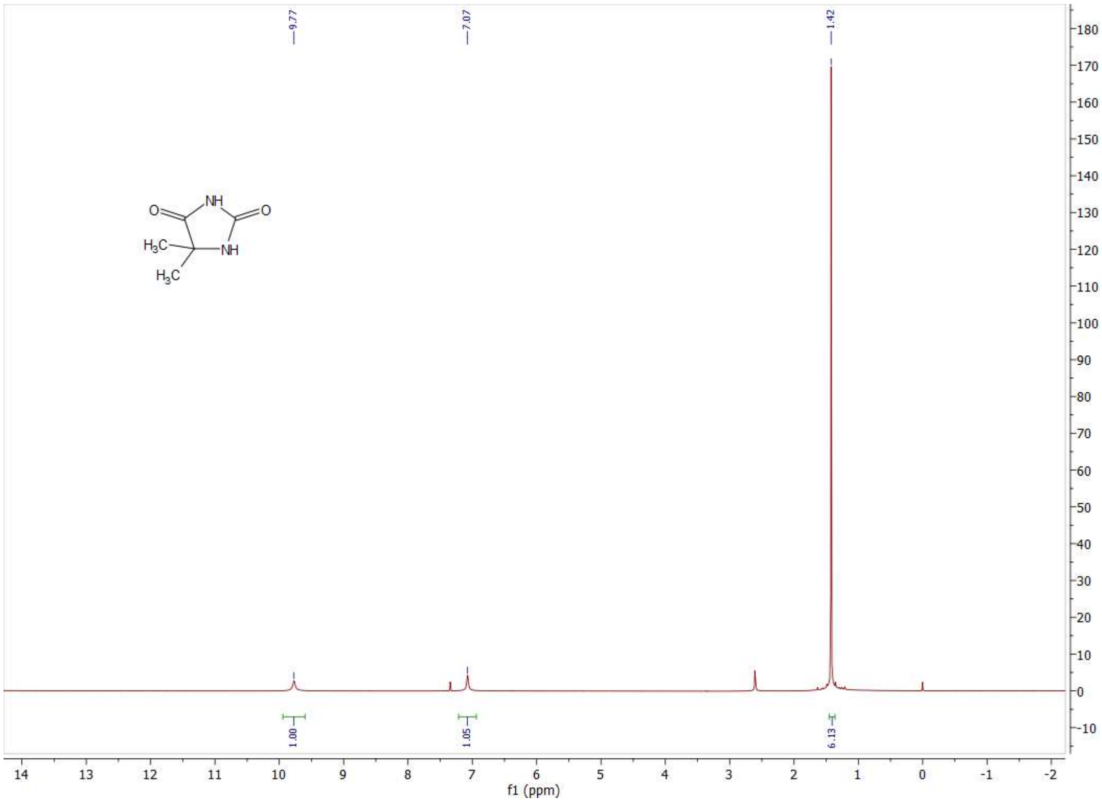

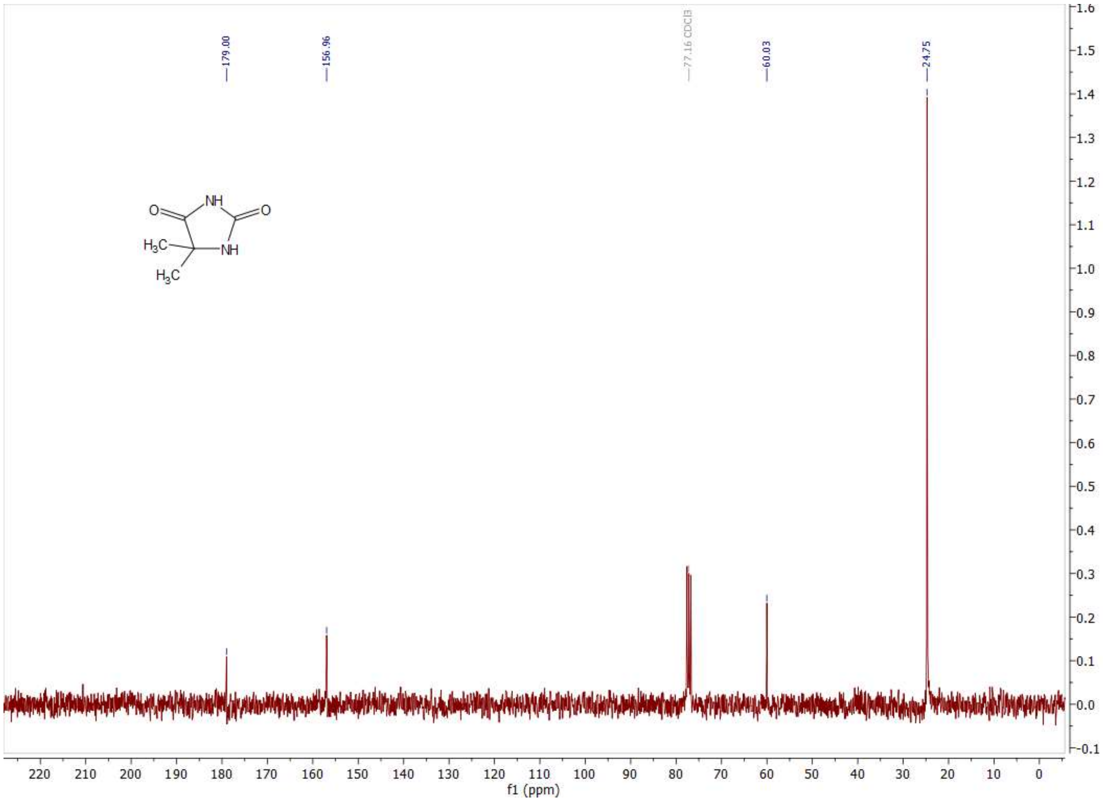

I. References

1. Chen, Z.; Wen, Y.; Fu, Y.; Chen, H.; Ye, M.; Luo, G., Graphene Oxide: An Efficient Acid Catalyst for the Construction of Esters from Acids and Alcohols. *Synlett* **2017,** 28, (08), 981-985.
2. Nguyen, T. V.; Lyons, D. J. M., A novel aromatic carbocation-based coupling reagent for esterification and amidation reactions. *Chem. Commun. (Cambridge, U. K.)* **2015,** 51, (15), 3131-3134.
3. Jia, J.; Jiang, Q.; Zhao, A.; Xu, B.; Liu, Q.; Luo, W.-P.; Guo, C.-C., Copper-Catalyzed O-Methylation of Carboxylic Acids Using DMSO as a Methyl Source. *Synthesis* **2016,** 48, (03), 421-428.
4. Shen, G.; Zhao, L.; Liu, W.; Huang, X.; Song, H.; Zhang, T., Convenient, metal-free *ipso*-nitration of arylboronic acids using nitric acid and trifluoroacetic acid. *Synth. Commun.* **2017,** 47, (1), 10-14.
5. Powell, A. B.; Stahl, S. S., Aerobic Oxidation of Diverse Primary Alcohols to Methyl Esters with a Readily Accessible Heterogeneous Pd/Bi/Te Catalyst. *Org. Lett.* **2013,** 15, (19), 5072-5075.
6. Cheung, C. W.; Buchwald, S. L., Mild and General Palladium-Catalyzed Synthesis of Methyl Aryl Ethers Enabled by the Use of a Palladacycle Precatalyst. *Org. Lett.* **2013,** 15, (15), 3998-4001.
7. Shao, C.; Lu, A.; Wang, X.; Zhou, B.; Guan, X.; Zhang, Y., Oxalic acid as the in situ carbon monoxide generator in palladium-catalyzed hydroxycarbonylation of arylhalides. *Org. Biomol. Chem.* **2017,** 15, (23), 5033-5040.
8. Offermann, D. A.; McKendrick, J. E.; Sejberg, J. J. P.; Mo, B.; Holdom, M. D.; Helm, B. A.; Leatherbarrow, R. J.; Beavil, A. J.; Sutton, B. J.; Spivey, A. C., Synthesis and Incorporation into Cyclic Peptides of Tolan Amino Acids and Their Hydrogenated Congeners: Construction of an Array of A–B-loop Mimetics of the Cε3 Domain of Human IgE. *J. Org. Chem.* **2012,** 77, (7), 3197-3214.
9. Dabral, S.; Mottweiler, J.; Rinesch, T.; Bolm, C., Base-catalysed cleavage of lignin β-O-4 model compounds in dimethyl carbonate. *Green Chem.* **2015,** 17, (11), 4908-4912.
10. Minakawa, M.; Baek, H.; Yamada, Y. M. A.; Han, J. W.; Uozumi, Y., Direct Dehydrative Esterification of Alcohols and Carboxylic Acids with a Macroporous Polymeric Acid Catalyst. *Org. Lett.* **2013,** 15, (22), 5798-5801.
11. Lai, L.; Li, A. N.; Zhou, J.; Guo, Y.; Lin, L.; Chen, W.; Wang, R., Mg(OMe)2 promoted allylic isomerization of γ-hydroxy-α,β-alkenoic esters to synthesize γ-ketone esters. *Org. Biomol. Chem.* **2017,** 15, (10), 2185-2190.
12. Li, G.; Zatolochnaya, O. V.; Wang, X.-J.; Rodríguez, S.; Qu, B.; Desrosiers, J.-N.; Mangunuru, H. P. R.; Biswas, S.; Rivalti, D.; Karyakarte, S. D.; Sieber, J. D.; Grinberg, N.; Wu, L.; Lee, H.; Haddad, N.; Fandrick, D. R.; Yee, N. K.; Song, J. J.; Senanayake, C. H., BABIPhos Family of Biaryl Dihydrobenzooxaphosphole Ligands for Asymmetric Hydrogenation. *Org. Lett.* **2018,** 20, (7), 1725-1729.
13. Dethe, D. H.; Erande, R. D.; Ranjan, A., Biomimetic Total Syntheses of Borreverine and Flinderole Alkaloids. *J. Org. Chem.* **2013,** 78, (20), 10106-10120.
14. Hutchby, M.; Houlden, C. E.; Haddow, M. F.; Tyler, S. N. G.; Lloyd-Jones, G. C.; Booker-Milburn, K. I., Switching Pathways: Room-Temperature Neutral Solvolysis and Substitution of Amides. *Angew. Chem., Int. Ed.* **2012,** 51, (2), 548-551.
15. Strazzolini, P.; Gambi, A.; G. Giumanini, A.; Vancik, H., The reaction between ethanedioyl (oxalyl) dihalides and Ag2C2O4: a route to Staudinger's elusive ethanedioic (oxalic) acid anhydride. *J. Chem. Soc., Perkin Trans. 1* **1998**, (16), 2553-2558.

16. Sun, H.-B.; Hua, R.; Yin, Y., ZrOCl2·8H2O: An Efficient, Cheap and Reusable Catalyst for the Esterification of Acrylic Acid and Other Carboxylic Acids with Equimolar Amounts of Alcohols. *Molecules* **2006,** 11, (4), 263-271.
17. Rohacova, J.; Marin, M. L.; Martinez-Romero, A.; O'Connor, J.-E.; Gomez-Lechon, M. J.; Donato, M. T.; Castell, J. V.; Miranda, M. A., Synthesis of new, UV-photoactive dansyl derivatives for flow cytometric studies on bile acid uptake. *Org. Biomol. Chem.* **2009,** 7, (23), 4973-4980.
18. Jin, C.; Wang, Y.; Sun, B.; Su, W., A Concise Synthesis of 25-Hydroxycholesterol from Hyodesoxycholic Acid. *J. Chem. Res.* **2018,** 42, (2), 96-99.
19. do Nascimento, P. G. G.; Lemos, T. L. G.; Almeida, M. C. S.; de Souza, J. M. O.; Bizerra, A. M. C.; Santiago, G. M. P.; da Costa, J. G. M.; Coutinho, H. D. M., Lithocholic acid and derivatives: Antibacterial activity. *Steroids* **2015,** 104, 8-15.
20. Maslov, M. A.; Morozova, N. G.; Solomatina, T. V.; Shaforostova, N. G.; Serebrennikova, G. A., Synthesis of amino analogues of cholic acid. *Russ. J. Bioorg. Chem.* **2011,** 37, (4), 507-515.
21. Suwada, M.; Fukuhara, T.; Hara, S., Selective mono-fluorination of diols via a cyclic acetal of N,N-diethyl-4-methoxybenzamide. *J. Fluorine Chem.* **2007,** 128, (10), 1121-1125.
22. Watanabe, S.; Fujita, T.; Sakamoto, M.; Kuramochi, T.; Kitazume, T., Reactions of monoesters of ethylene glycol with N,N-diethyl-1,1,2,3,3,3-hexafluoropropylamine. *J. Fluorine Chem.* **1987,** 36, (3), 361-372.
23. Jiang, Q.; Zhao, A.; Xu, B.; Jia, J.; Liu, X.; Guo, C., PIFA-Mediated Esterification Reaction of Alkynes with Alcohols via Oxidative Cleavage of Carbon Triple Bonds. *J. Org. Chem.* **2014,** 79, (6), 2709-2715.
24. Umeda, R.; Nishimura, T.; Kaiba, K.; Tanaka, T.; Takahashi, Y.; Nishiyama, Y., Rhenium complex-catalyzed acylative cleavage of ethers with acyl chlorides. *Tetrahedron* **2011,** 67, (38), 7217-7221.
25. Niculescu-Duvaz, I.; Elian, I.; Ionescu, M.; Tarnauceanu, E., New urethanic type nitrogen mustards derived from steroidic structures. *Journal fur Praktische Chemie* **1979,** 321, (3), 522-528.
26. Whittaker, A. M.; Dong, V. M., Nickel-Catalyzed Dehydrogenative Cross-Coupling: Direct Transformation of Aldehydes into Esters and Amides. *Angew. Chem., Int. Ed.* **2015,** 54, (4), 1312-1315.
27. Hosseini-Sarvari, M.; Sodagar, E., Esterification of free fatty acids (Biodiesel) using nano sulfated-titania as catalyst in solvent-free conditions. *C. R. Chim.* **2013,** 16, (3), 229-238.
28. Zígolo, M. A.; García Liñares, G.; Baldessari, A., New cholic acid derivatives: Biocatalytic synthesis and molecular docking study. *Steroids* **2016,** 107, 10-19.
29. Marples, B. A.; Stretton, R. J. Method of treating fungal infections using sterodial ester compounds. US5266566 (A), 1993-11-30, 1993.
30. Manta; Lazar, *Rev. Chem. Bukarest* **1958**.
31. Gianetti, T. L.; Annen, S. P.; Santiso-Quinones, G.; Reiher, M.; Driess, M.; Grützmacher, H., Nitrous Oxide as a Hydrogen Acceptor for the Dehydrogenative Coupling of Alcohols. *Angew. Chem., Int. Ed.* **2016,** 55, (5), 1854-1858.
32. Hatano, M.; Tabata, Y.; Yoshida, Y.; Toh, K.; Yamashita, K.; Ogura, Y.; Ishihara, K., Metal-free transesterification catalyzed by tetramethylammonium methyl carbonate. *Green Chem.* **2018,** 20, (6), 1193-1198.
33. Kaul, S.; Kumar, A.; Sain, B.; Gupta, A. K., A SIMPLE AND CONVENIENT ONE-POT SYNTHESIS OF FATTY ACID ESTERS FROM HINDERED ALCOHOLS

USING N,N-DIMETHYLCHLORO-SULFITEMETHANIMINIUM CHLORIDE AS DEHYDRATING AGENT. *Synth. Commun.* **2002,** 32, (18), 2885-2891.
34. Sasaki, T.; Eguchi, S.; Ryu, I. H.; Hirako, Y., Preparation of 1- and 2-adamantyldiazomethanes and their application for synthesis of some adamantane derivatives. *Tetrahedron Lett.* **1974,** 15, (23), 2011-2014.
35. Park, Y. J.; Arasu, M. V.; Al-Dhabi, N. A.; Lim, S. S.; Kim, Y. B.; Lee, S. W.; Park, S. U., Expression of Terpenoid Biosynthetic Genes and Accumulation of Chemical Constituents in *Valeriana fauriei*. *Molecules* **2016,** 21, (6), 691-706.
36. Jia, M.; Zhao, R.; Xu, B.; Yan, W.; Chu, F.; Gu, H.; Xie, T.; Xiang, H.; Ren, J.; Chen, D.; Wang, P.; Lei, H., Synthesis and biological activity evaluation of novel peroxo-bridged derivatives as potential anti-hepatitis B virus agents. *MedChemComm* **2017,** 8, (1), 148-151.
37. Koch, V.; Bräse, S., Pd-mediated cross-coupling of C-17 lithiated androst-16-en-3-ol – access to functionalized arylated steroid derivatives. *Org. Biomol. Chem.* **2017,** 15, (1), 92-95.
38. Watanabe, Y.; Sawada, K.; Hayashi, M., A green method for the self-aldol condensation of aldehydes using lysine. *Green Chem.* **2010,** 12, (3), 384-386.
39. Tomioka, T.; Sankranti, R.; Vaughan, T. G.; Maejima, T.; Yanase, T., An α-Diaminoboryl Carbanion Assisted Stereoselective Single-Pot Preparation of α,β-Disubstituted Acrylonitriles. *J. Org. Chem.* **2011,** 76, (19), 8053-8058.
40. Pérez-Sánchez, M.; de María, P. D., Synthesis of natural fragrance jasminaldehyde using silica-immobilized piperazine as organocatalyst. *Catal. Sci. Technol.* **2013,** 3, (10), 2732-2736.
41. Arias Ugarte, R.; Devarajan, D.; Mushinski, R. M.; Hudnall, T. W., Antimony(v) cations for the selective catalytic transformation of aldehydes into symmetric ethers, α,β-unsaturated aldehydes, and 1,3,5-trioxanes. *Dalton Trans.* **2016,** 45, (27), 11150-11161.
42. Abanda-Nkpwatt, D.; Schwab, W., Microbial Transformation of Aliphatic Aldehydes by Bacillus megaterium to 2,3-Dialkylacroleins. *J. Agric. Food Chem.* **2004,** 52, (19), 5939-5942.
43. Haibin, L.; Dejuan, K.; Bin, W.; Shihan, W.; Yongsheng, W., Synthesis and Evaluation of Anti-Inflammatory and Antitussive Activity of Hydantion Derivatives. *Lett. Drug Des. Discovery* **2012,** 9, (6), 638-642.
